# Phosphate-Based Self-Immolative Linkers for the Delivery of Amine-Containing Drugs

**DOI:** 10.3390/molecules26175160

**Published:** 2021-08-25

**Authors:** Mateja Đud, Markéta Tichotová, Eliška Procházková, Ondřej Baszczyňski

**Affiliations:** 1 Faculty of Science, Charles University, 128 43 Prague, Czech Republic; mateja.dud@natur.cuni.cz (M.Đ.); marketa.tichotova@uochb.cas.cz (M.T.); 2 Institute of Organic Chemistry and Biochemistry, The Czech Academy of Sciences, 166 10 Prague, Czech Republic

**Keywords:** ^31^P-NMR spectroscopy, amine-containing drugs, phosphate-based linkers, prodrugs, self-immolative linkers

## Abstract

Amine-containing drugs often show poor pharmacological properties, but these disadvantages can be overcome by using a prodrug approach involving self-immolative linkers. Accordingly, we designed l-lactate linkers as ideal candidates for amine delivery. Furthermore, we designed linkers bearing two different cargos (aniline and phenol) for preferential amine cargo release within 15 min. Since the linkers carrying secondary amine cargo showed high stability at physiological pH, we used our strategy to prepare phosphate-based prodrugs of the antibiotic Ciprofloxacin. Therefore, our study will facilitate the rational design of new and more effective drug delivery systems for amine-containing drugs.

## 1. Introduction

Drugs containing an amino group are key pharmaceutical agents, covering a broad spectrum of biological actions and displaying anti-inflammatory [1], anticancer [2,3], antimicrobial [4,5], and pain-relieving properties [6]. Currently, 542 drugs containing an amino group have already been approved for the EU market, according to the drug bank online (https://go.drugbank.com, accessed on 15 July 2021). This number does not include many other biologically active compounds—potential leads—or compounds from natural resources, such as alkaloids. However, amine drugs often show poor pharmacological properties, such as low aqueous solubility and poor membrane permeability due to ionization of the amino group [7] under physiological conditions. Nevertheless, amines are generally susceptible to derivatization. Thus, a prodrug strategy [8,9,10] can be used to overcome these drawbacks [7,11]. 

Prodrug strategies rely on a structural modification (masking) of the active pharmaceutical agent—a drug—with a suitable protecting group (promoiety) to modulate its pharmacokinetic properties. Such a change helps to facilitate drug delivery to the target site (e.g., tissues, cells, cell compartments, or organs). One of the most rapidly developing prodrug strategies consists of using self-immolative (SI) linkers [12,13] to control drug release [14]. 

SI linkers are covalent assemblies that couple an active compound (drug) to a protecting group. After external stimuli, either chemical or enzymatic, a cascade of spontaneous reactions [15] leads to linker fragmentation and consequently to drug release. The two main classes of SI linkers are (1) carbamates and (2) phosphate-based systems. Phosphorus-based SI linkers stand above the “classical” carbamate linkers because they make it possible to attach an additional substituent, which can help fine-tune the SI rate or provide a double cargo option.

Phosphorus-based SI linkers have been introduced as suitable drug-delivery systems for several drugs. A paradigmatic example of a phosphorus-based SI linker application is the methoxymethylphosphonic acid (MMPA) drug delivery vehicle for the oral delivery of propofol [16]. Other examples include pro-nucleotide prodrugs (ProTides) [17], which have been used to treat various viral infections, such as HIV [18], hepatitis B [19], or SARS-CoV-2 (COVID-19) [20]. Considering their success as drug-delivery systems, phosphate-based SI linkers may find broader applications in drug discovery and materials science through systematic studies. 

**Figure 1:**
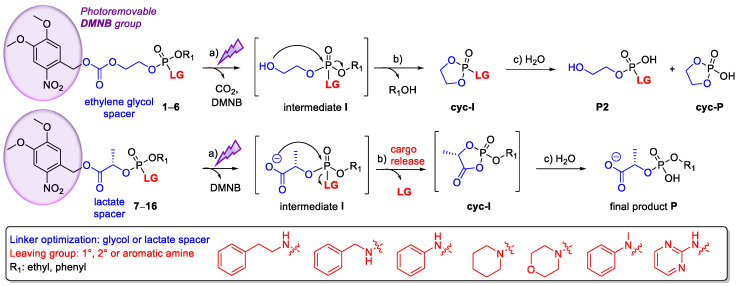
Self-immolation (SI) of **1**–**16**; (a) SI is initiated by UV light (365 nm), which cleaves a photosensitive DMNB group [21]; (b) the intermediate **I** then spontaneously cyclizes, releasing R_1_ (**4**–**6**) or LG (**7**–**16**); (c) hydrolysis of the lactate-based cyclic intermediate leads to the final product **P**, whereas glycol-based **cyc-I** hydrolyzes to the final product **P2** or the cyclic product **cyc-P**.

This study reports the development of new phosphate-based SI linkers designed to release amine-containing cargos (Figure 1). For this purpose, we searched for a suitable spacer responsible for SI. Although SI of ethylene glycol linkers **1**–**6** did not lead to amine release in a reasonable time, our lactate linkers **7**–**9** showed successful cargo release. After screening a wide range of linkers bearing various amines (**10**–**16**), representing model drugs, we prepared a prodrug of the FDA-approved drug Ciprofloxacin, which is a broad-spectrum fluoroquinolone antibiotic. 

## 2. Results

### 2.1. Ethylene Glycol Phosphate-Based Linkers

**Scheme 1:**
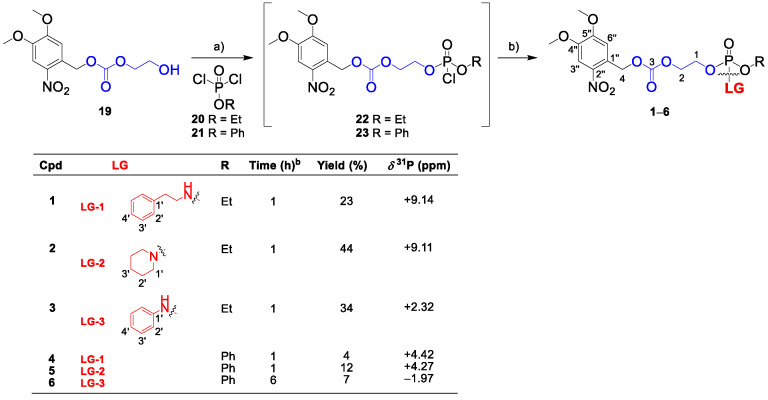
Linkers **1**–**6** bearing ethylene glycol spacer were synthesized under the following reaction conditions: (a) ethyl (**20**) or phenyl (**21**) dichlorophosphate (0.5 mmol), TEA (0.65 mmol), toluene, 25 °C, 16 h; (b) the corresponding amine (0.5 mmol), TEA (0.5 mmol), toluene, 25 °C, 1–6 h.

Ethylene glycol-based linkers provide stable cyclic intermediates traceable by NMR spectroscopy, which indicates that the cargo is released via SI [22]. For proof of concept, we prepared glycol-based linkers **1**–**6** (Scheme 1) bearing phenethylamine, piperidine, and aniline (LG-1, LG-2 and LG-3, respectively) as representatives of primary, secondary, and aromatic amines, respectively. 

Target linkers were synthesized in two consecutive phosphorylation steps, starting from commercially available phosphorodichloridates **20**–**21** (Scheme 1). Carbonate **19** was prepared in a reaction between ethylene glycol and dimethoxynitrobenzyl (DMNB) chloroformate in THF, using pyridine as the base [22]. The reaction of equimolar amounts of dichloridates **20**–**21** and DMNB carbonate **19** in the presence of triethylamine (TEA) in toluene gave intermediates **22** and **23**. This reaction was monitored by ^31^P-NMR spectra (signal at δ_P_ 4.7 ppm for **22** and δ_P_ 0.08 ppm for **23**, in CDCl_3_), and intermediates **22** and **23** were directly used for the second phosphorylation step. The reaction of **22** and **23** with one equivalent of the corresponding amine and TEA as a base afforded the final linkers **1**–**6**. The isolated yields in the series bearing ethoxy substituent (**1**–**3**) were moderate (23–44%), whereas compounds **4**–**6** were isolated in low yields (4–12%) despite multiple flash chromatography (silica gel and C18). 

The self-immolation of **1**–**6** was triggered photochemically (365 nm), and the reaction was monitored by ^31^P-NMR spectroscopy. Surprisingly, the successful photoactivation of **1**–**3** yielded intermediates (**1**–**3**)-**I,** with no further spectral change over several days, thus indicating that the cargo was not released (Figure S1 in Supplementary Materials). 

**Figure 2:**
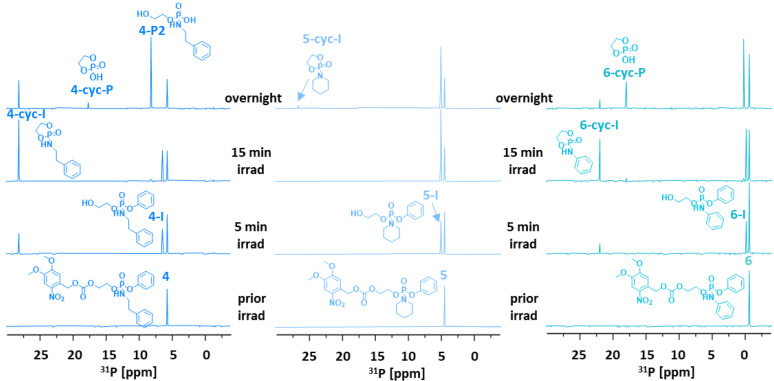
^31^P-NMR spectra of linkers **4**–**6** (5 mM in 50% CACO/DMSO) measured before and after irradiation with UV light (365 nm) at room temperature (25 °C), previously optimizing the reaction conditions (DMSO/cacodylate buffer (CACO), 0.1 M, pH = 7.4; 1/1, *v*/*v*) [24].

In contrast to **1**–**3**, linkers **4** and **6** afforded cyclic intermediates **4-cyc-I** and **6-cyc-I** within 5 min of irradiation (Figure 2). In compound **5**, bearing a secondary amine, only a trace of **5-cyc-I** was detected overnight. However, the downfield shifted ^31^P-NMR signals of cyclic intermediates **4-cyc-I, 5-cyc-I,** and **6-cyc-I** (δ_P_ 28.0, 26.5, 21.7 ppm, respectively) indicated that the amine cargo was still attached to the phosphorus and that phenol was released instead. 

^31^P-NMR spectra recorded overnight suggested three different scenarios: (1) preferential release of phenol as **4-cyc-I** and **4-P2** were detected; (2) formation of the stable intermediate **5-I**, which released phenol in several days; (3) sequential release of phenol in minutes (**6-cyc-I**) and aniline overnight (**6-cyc-P**). The formation of phospholane intermediates (**4-6**)**-cyc-I** could be useful for other applications, such as preparing functional biopolymers for controlled drug delivery systems [23], but linkers **1**–**6** did not release amine successfully. Therefore, we altered the spacer structure to find an effective system for releasing amine-containing cargos.

### 2.2. Lactate Phosphate-Based Linkers

**Scheme 2:**
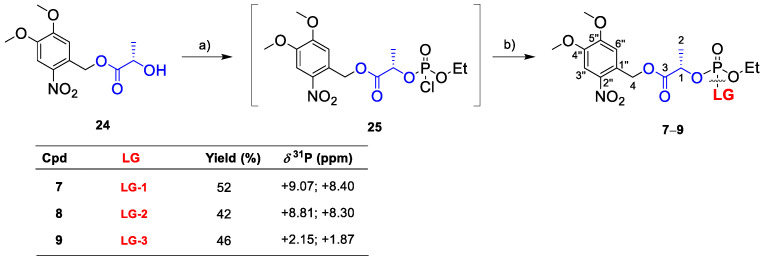
Lactate-based linkers **7**–**9** were synthesized under the following reaction conditions: (a) ethyl dichlorophosphate **20** (0.5 mmol), TEA (0.65 mmol), dry toluene, 25 °C, 16 h; (b) the corresponding amine (0.5 mmol), TEA (0.5 mmol), dry toluene, 25 °C, 1 h.

To stimulate amine cargo release, we altered the glycol spacer in **1**–**3** to an l-lactate spacer, thus preparing linkers **7**–**9** (Scheme 2). Despite promoting a slow release of phenolic compounds, [25] the lactate spacer could be suitable for amines. Compounds **7**–**9** were synthesized from DMNB ester **24** via acid-catalyzed esterification in refluxing toluene [25], and intermediate **25** was generated in a reaction of **24** with dichloridate **20** in toluene. The reaction was monitored by ^31^P-NMR, and a new pair of ^31^P-NMR signals at δ_P_ 4.6 ppm and δ_P_ 4.1 ppm (ca. 1:1 ratio), corresponding to two diastereoisomers of **25**, was observed due to the new stereogenic center on phosphorus. Intermediate **25** was directly used for amine phosphorylation, and the final products **7**–**9** were isolated with good yields (42–52%) as 1:1 mixtures of diastereoisomers, as shown by the two sets of NMR signals.

The SI of **7**–**9**, monitored by ^31^P-NMR, showed successful amine release, which provided the final product **P** in 15 min (Figure S2 in the Supplementary Materials). However, in **7** and **9**, we also detected a new ^31^P signal (δ_P_ −1.6 ppm) belonging to the undesired product of alternative decomposition (**7**-**X** and **9-X**). Interestingly, linker **8**, bearing a secondary amine as a cargo, did not form the undesired product **8**-**X** and followed the expected reaction course. 

Given the unexpected reactivity of **7** and **9** in the CACO/DMSO mixture (1/1, *v*/*v*), we optimized the solvent system. For this purpose, we performed irradiation experiments on **7**, in various solvent mixtures (Figure S3 in the Supplementary Materials), and we found that the formation of side product **7-X** can be suppressed by either decreasing the pH of the cacodylate buffer (to pH = 5) or changing the buffer itself. HEPES buffer or an unbuffered system can suppress the formation of **7-X**. Lastly, we selected the HEPES (pH 7.4)/DMSO system (1:1, *v*/*v*) for further SI investigation.

**Figure 3:**
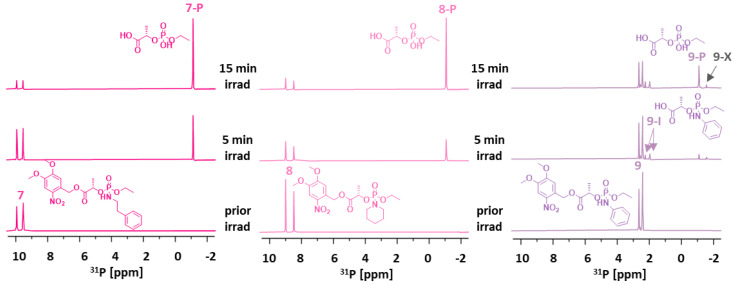
^31^P NMR spectra of linkers **7**–**9** (5 mM), measured before and after irradiation by UV light (365 nm) in a solvent mixture of 50% HEPES (pH = 7.4)/DMSO at room temperature (25 °C).

We monitored the SI of **7**–**9** in the HEPES/DMSO solvent mixture (Figure 3). In 5 min, we detected the final product **P** in all cases (δ_P_ −1.1 ppm). Linkers **7** and **8** did not provide any intermediate **7-I** and **8-I**, respectively, as they undergo fast cyclization, and photoactivation is a rate-limiting step. In turn, the SI of **9** was slow, and we did detect intermediate **9-I** (Figure 3, right) or traces of the undesired product **9-X** (δ_P_ −1.6 ppm). Considering the overall limited stability of **7** and **9**, we investigated the formation of the unknown side product **X** and performed stability tests in **7**–**9**. The results showed the limited stability of **7** and **9**, which were significantly decomposed within 7 days (Figures S6 and S7 in the Supplementary Materials, details therein).

### 2.3. Characterization of the Undesired Product **X**

**Figure 4:**
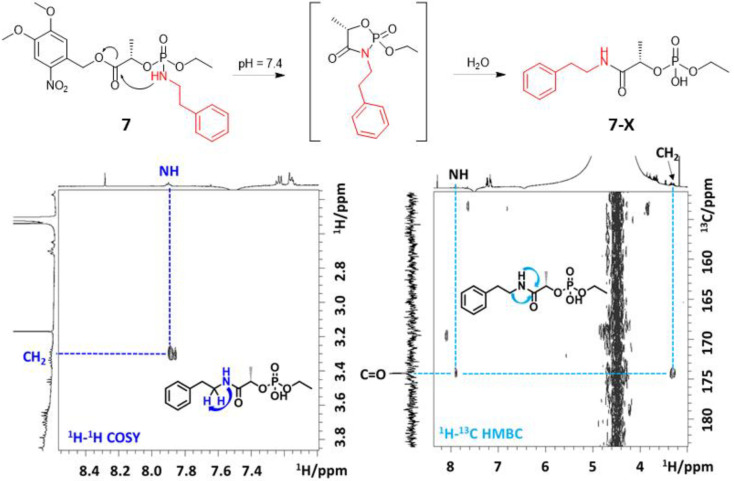
The proposed mechanism of intramolecular rearrangement: part of the COSY and HMBC spectra recorded after UV irradiation of **7** (5 mM solution in CACO (pH 7.4)/DMSO) show cross-peaks between lactate C=O and phenethylamine NH-CH_2_ groups, confirming the structure of **7**-**X**.

To identify the alternative decomposition pathway, we characterized the undesired product **X**. The significant upfield shift (δ_P_ < 0) suggested that the P-NH bond had been cleaved. In addition, one singlet ^31^P-NMR signal indicated the lack of a stereogenic center on the phosphorus atom. ^31^P-NMR chemical shifts of **7-X** and **9-X** differed slightly (δ_P_ −1.66 and −1.58 ppm, respectively), as found in the starting compounds **7** and **9** (differing in LGs). Combined, these findings demonstrate that **7-X** and **9-X** also differ in the amine moiety, which may be explained by the intramolecular rearrangement proposed in Figure 4. Additional 2D NMR experiments performed on linker **7** suggested the formation of carboxamide **7**-**X**. We found the key interaction between the phenethylamine alkyl chain and lactate carbonyl in the HMBC spectrum (Figure 4). The proposed structure of **7**-**X** was confirmed by HR-MS (Figure S64 in the Supplementary Materials).

Only the linkers containing the NH group in the phosphoramidate bond underwent the proposed intramolecular rearrangement (linker **8** without NH did not form **8-X**). Indeed, *N*-methylation of **9** yielded linker **10**, which did not form the undesired product **10-X** (Figure S4 in the Supplementary Materials). This intramolecular rearrangement has already been reported by the Mulliez group in 1985 [26].

### 2.4. Amine Screening—Application Scope 

**Scheme 3:**
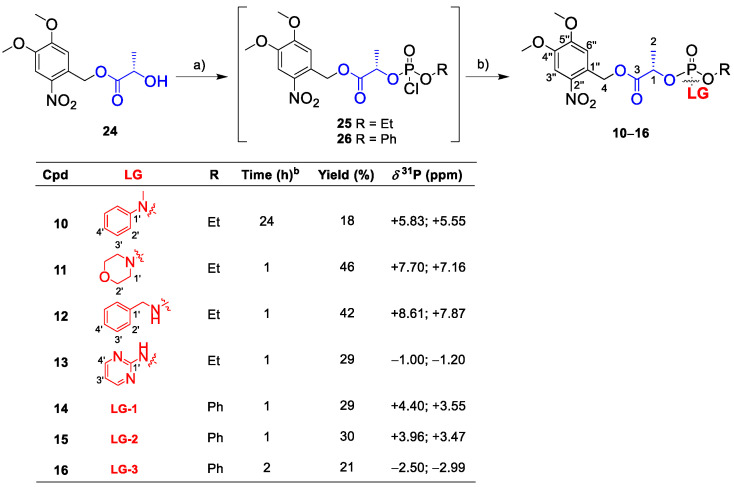
Lactate-based linkers **10**–**16** were synthesized under the following reaction conditions: (a) ethyl or phenyl dichlorophosphate **20**–**21** (0.5 mmol), TEA (0.65 mmol), dry toluene, 25 °C, 16 h; (b) the corresponding amine (0.5 mmol), TEA (0.5 mmol), dry toluene, 25 °C, 1–24 h.

Based on the successful SI observed in linkers **7**–**9**, we examined the synthetic scope and application feasibility of lactate linkers by altering the structure of the cargo. Accordingly, we prepared linkers **10**–**16** (Scheme 3). 

Linkers with aliphatic amines (morpholine and benzylamine) were synthesized as final products **11** and **12**, respectively. Aromatic amines provided only two linkers bearing *N*-methylaniline and 2-aminopyrimidine (**10** and **13**, respectively). Other heterocyclic amines, such as imidazole, indoline, 2-aminobenzothiazole, 1- and 2-aminobenzoimidazole, and 2-aminobenzoxazole, yielded complex reaction mixtures, as shown in ^31^P-NMR spectra (Figure S9 in the Supplementary Materials), which we were unable to separate. 

Since cargo release was faster in **4**–**6**, phenyl-lactyl phosphate analogs **14**–**16** were also prepared with a phenyl instead of an ethyl group attached to the phosphorus.

**Figure 5:**
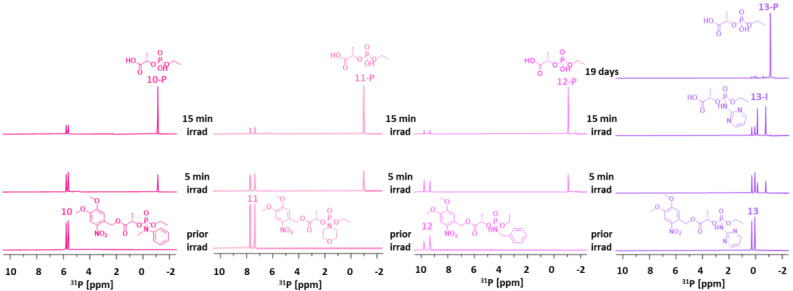
^31^P-NMR spectra of linkers **10**–**13**, measured before and after irradiation with UV light (365 nm) of 5 mM solutions in a solvent mixture of 50% HEPES (pH = 7.4)/DMSO at room temperature (25 °C).

**Figure 6:**
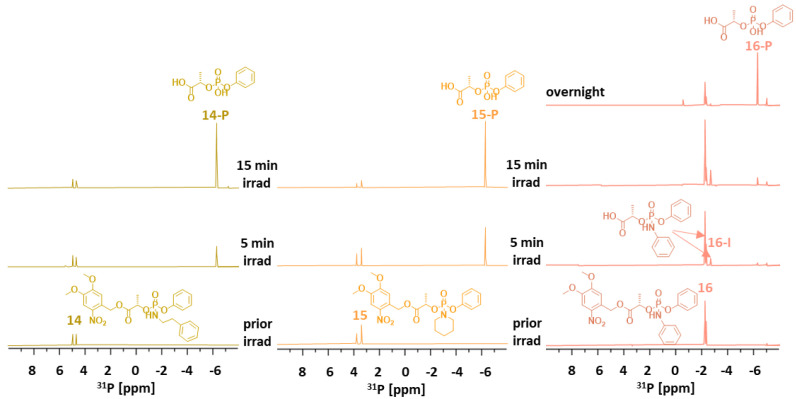
^31^P-NMR spectra of linkers **14**–**16**, measured before and after irradiation with UV light (365 nm) using 5 mM solutions in a solvent mixture of 50% HEPES (pH = 7.4)/DMSO at room temperature (25 °C).

Then, we subjected **10**–**16** to irradiation NMR experiments (Figure 5 and Figure 6). Compounds **10**–**12** afforded the final product **P** within 5 min, and product **P** was a major component in the reaction mixtures in 15 min. In contrast, 2-aminopyrimidine derivative **13** showed a slow formation of intermediate **13**-**I** without any further spectral change in 15 min. Lastly, the pyrimidine cargo was fully released from **13**-**I** within 19 days. 

Linkers **14**–**16** released their amine cargos slightly faster than their ethyl counterparts **7**–**9**. Linkers **14**–**16** afforded the final product **P**, which emerged as one singlet ^31^P signal at δ_P −_6 ppm (Figure 6). Although **14** and **15** released the corresponding amines within 5 min, linker **16** did so overnight. 

### 2.5. Application

**Figure 7:**
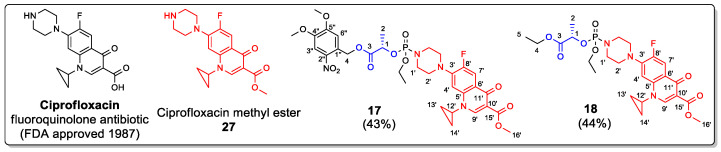
Chemical structures of the FDA-approved fluoroquinolone antibiotic Ciprofloxacin, its methyl ester **27**, and the prodrugs activated by light (**17**) or by an enzyme (**18**) prepared in this work.

Ultimately, we prepared two model prodrugs of Ciprofloxacin (Figure 7), which is a known fluoroquinolone antibiotic containing a secondary amino group, to demonstrate the applicability of lactate phosphate-based linkers. To avoid side reactions during the synthesis of **17** and **18**, we protected the carboxylic group of Ciprofloxacin by methylation, which should not decrease the antibiotic activity as reported previously [27]. Then, Ciprofloxacin methyl ester **27** was phosphorylated, following the procedure that had been used for model linkers **7**–**9** (Scheme 2). Two-step phosphorylation starting from DMNB ester **24** and ethyl dichlorophosphate **20** afforded photoactivable compound **17**. In addition to **17**, we also prepared its enzymatically activable analog **18**. Both compounds were obtained in moderate isolated yields (43–44%) as ca. 1:1 mixtures of diastereoisomers, as confirmed by the presence of two sets of NMR signals.

**Figure 8:**
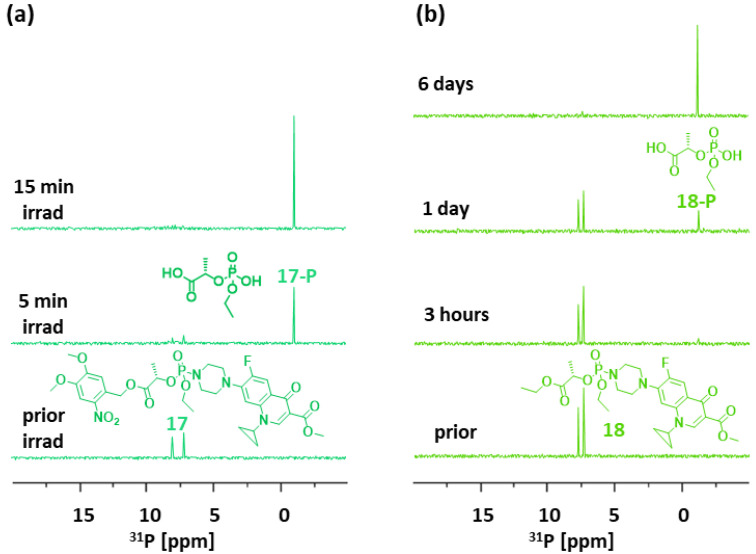
^31^P-NMR spectra of Ciprofloxacin prodrugs **17** (**a**) and **18** (**b**) recorded in 50% HEPES (pH = 7.4)/DMSO, 25 °C, with linker **17** bearing a photoactivable DMNB group, before and after irradiation by UV light (365 nm), and linker **18**, where SI is activated enzymatically by lipase, before and after lipase addition.

The photoactivation of linker **17** resulted in Ciprofloxacin release in 5 min, which was supported by the formation of product **17**-**P** (Figure 8a). Compound **18** was activated by a lipase from *Candida Antarctica*. We detected **18**-**P** after 3 h, with ca. 30% cargo release in 24 h. Most Ciprofloxacin (97%) was released in 6 days (Figure 8b). Enzymatic cargo release was relatively slow, which was presumably due to the substrate specificity of the lipase that was used in the experiment. 

Furthermore, we performed a biological screening of **18** and **27**, which showed that Ciprofloxacin methylation at the carboxylic moiety inhibits the antibiotic activity of this fluoroquinolone (Table S1 in the Supplementary Materials), despite previous reports stating otherwise [27]. Nevertheless, we believe that phosphate-based linkers may be used to design secondary amine drug delivery systems.

## 3. Discussion

Our study demonstrates that a universal spacer for delivering all types of amines will unlikely ever be designed given the sensitivity of the phosphorus atom to substitution. An ethylene glycol spacer, which was previously identified as the best linker for phenolic cargo delivery [22], proved inefficient in delivering amines, as shown in **1**–**6**. An alteration in the electron density of phosphorus caused by oxygen substitution (from phenol—previous work [22]) for nitrogen (from amine) could explain the inefficiency of **1**–**6**. Although installing phenol (**4**–**6**) as the second cargo slightly accelerated the SI, when compared to the ethyl analogs **1**–**3**, phenol was preferentially released instead of the amine cargos. Surprisingly, the l-lactate spacer was the most suitable for aliphatic amines, releasing the cargo in 15 min (**7**, **8**, **11**, **12**, **14**, **15**). In contrast, linkers with aromatic amine cargos (aniline (**9** and **16**), *N*-methylaniline (**10**), and 2-aminopyrimidine (**13**)) released their cargos more slowly than linkers bearing aliphatic amines, as indicated by the formation of higher amounts of intermediate **I**.

Slower SI, especially in **13** (**13**-**I**), could be partly explained by the low p*K*_a_ of 2-aminopyrimidine (p*K*_a_ 3.54 [28]). Although there is no clear correlation between the p*K*_a_ of amine and the SI rate, p*K*_a_ plays a key role in amine release [29]. *N*-protonation in phosphoramidates facilitates the nucleophilic attack of water (a carboxylate group in our case) to the phosphorus atom. Imbach [30] has shown that phosphoramidates consisting of low p*K*_a_ amines are stable, whereas those containing amines with a higher p*K*_a_ (more than 9) show the fastest hydrolysis, which has also been described by Wagner [31]. Nevertheless, differences in cargo release rate could not be easily explained by the various p*K*_a_ of amines. Based on Mayr’s extensive studies of amine behavior in solution, the amine leaving group can be affected by attributes other than p*K*_a_, such as nucleophilicity [32], hydration energy (amine stabilization by solvation in water), polarity, and solvent pH, in addition to structural (cyclic vs. acyclic) or sterical effects. Therefore, predicting the optimal spacer for a specific cargo is a difficult task, and designing purposeful drug delivery systems requires studying structure-activity relationships in detail.

## 4. Materials and Methods

Unless otherwise indicated, all chemicals were purchased from commercial suppliers (Sigma Aldrich, Merck, EU; Fluorochem, UK; Acros Organics, Thermo Fisher Scientific, EU) and used without further purification. All reactions sensitive to air or moisture were performed under an inert atmosphere of argon in dry solvents. Thin layer chromatography (TLC) was performed on TLC aluminium sheets (silica-gel 60 F_254_; Merck, EU) and visualized by UV fluorescence. The reaction was monitored by TLC and/or ^31^P-NMR spectroscopy in CDCl_3_. Flash-column chromatography was performed on a Compact (ECOM s.r.o., EU) chromatography system using silica-gel or C18 silica-gel 230–400 mesh, 60 Å (Merck KGaA, EU). 

NMR spectroscopy. NMR spectra were recorded on a Bruker Avance III spectrometer operating at 400 MHz for ^1^H and 101 MHz for ^13^C equipped with a probe with an ATM module (5 mm BBFO BB-19F/1H/D Z-GRD). For NMR signal assignment, standard Bruker pulse sequences were used for 1D (^1^H, ^13^C-APT, ^31^P, ^19^F) and 2D (COSY, ROESY, HSQC, HMBC) NMR experiments at a corrected temperature of 25 °C. NMR spectra coupled with UV irradiation were recorded on a Bruker Avance III spectrometer with a broad-band cryo probe with an ATM module (5 mm CPBBO BB-1H/19F/15N/D Z-GRD) operating at 500 MHz for ^1^H and 125.7 MHz for ^13^C. All NMR data were interpreted using Topspin 3.5. For reference, the following solvent signals were used: DMSO-*d_6_*: 2.50 (^1^H) and 39.7 (^13^C) ppm or CDCl_3_: 7.28 (^1^H) and 77.0 (^13^C) ppm. The ^31^P-NMR spectra were referenced to H_3_PO_4_ with 0 ppm.

For NMR experiments with in situ irradiation, a light emitting diode (LED; Thorlabs, EU) was used at 365 nm. The light was guided into the spectrometer, directly into the NMR tube via a multimode silica optical fiber with 1 mm diameter, 0.39 NA, and a high amount of OH (Thorlabs, EU). 

Mass spectrometry. Mass spectra were measured on a LTQ. Orbitrap XL (Thermo Fisher Scientific, EU) using electrospray ionization (ESI).

All products were viscous oils, semi-solids or non-crystalline solids. The reaction conditions were not optimized for the highest possible yields.

***4,5-dimethoxy-2-nitrobenzyl (2-hydroxyethyl) carbonate* (19)** was prepared according to a literature procedure [22], using 1.0 g of 4,5-dimethoxy-2-nitrobenzyl chloroformate and obtained (0.98 g) as a yellow solid (90%).

***l-4,5-dimethoxy-2-nitrobenzyl 2-hydroxypropanoate* (24)** was prepared according to a literature procedure [25], using 2.13 g of 4,5-dimethoxy-2-nitrobenzyl alcohol and obtained (0.94 g) as a light pink solid (33%). 

**General Procedure for the One-Pot Synthesis of Phosphate-Based Linkers.** For each experiment, a DMNB-containing photoarm **19** or **24** (0.5 mmol, 1.0 eq.) was dissolved in 2.5 mL of dry toluene under argon at 25 °C, adding dry TEA (90.6 μL, 0.65 mmol, 1.3 eq.) followed by the corresponding dichlorophosphate (0.5 mmol, 1.0 eq.). The reaction mixture was stirred at 25 °C for 16 h, and the formation of the intermediates was confirmed by ^31^P-NMR (**22**: δ_P_ 4.7 ppm; **23**: δ_P_ 0.08 ppm; **25**: δ_P_ 4.6 and 4.1 ppm; **26**: δ_P_ −0.08 and −0.17 ppm) before phosphorylating the amines. The corresponding amine (0.5 mmol, 1.0 eq.) was added, followed by dry TEA (69.7 μL, 0.5 mmol, 1.0 eq.). The reaction mixture was stirred at room temperature until completing the reaction (monitored by ^31^P-NMR). After evaporating the solvent, pure products were isolated by Flash silica gel chromatography, which was followed by reverse-phase chromatography, as described for each compound.

***4,5-dimethoxy-2-nitrobenzyl (2-((ethoxy(phenethylamino)phosphoryl)oxy)ethyl) carbonate* (1)** was prepared from **19** (150.5 mg, 0.5 mmol, 1.0 eq.) and **20** (63.2 μL, 0.5 mmol, 1.0 eq.) according to the general procedure and purified by Flash silica gel chromatography using a gradient (CH_2_Cl_2_/MeOH 100:0→90:10, *v*/*v*), followed by reverse-phase chromatography using a gradient (H_2_O/MeCN 100:0→50:50, *v*/*v*), which afforded **1** as a dense yellow oil (58.6 mg, 23%). **^1^H-NMR** (400 MHz, CDCl_3_, 25 °C) δ 7.74 (s, 1H, 3″), 7.30 (m, 2H, 3′), 7.25–7.20 (m, 1H, 4′), 7.20 (m, 2H, 2′), 7.07 (s, 1H, 6″), 5.60 (s, 1H, 4a), 5.60 (s, 1H, 4b), 4.40 (m, 2H, 2), 4.25–4.10 (m, 2H, 1), 4.10–3.99 (m, 2H, O**CH_2_**CH_3_), 3.98 and 3.97 (-s, 6H, 4″-O**CH_3_**, 5″-O**CH_3_**), 3.25–3.16 (m, 2H, NH**CH_2_**), 2.80 (m, 1H, **CH_2_**Ph), 1.32 (td, 3H, *J*_CH3-CH2_ = 7.1 Hz, *J*_CH3-P_ = 0.8 Hz, OCH_2_**CH_3_**). **^13^C-NMR** (101 MHz, CDCl_3_, 25 °C) δ 154.59 (3), 153.52 and 148.38 (4″, 5″), 139.68 (2″), 138.53 (1′), 128.88 (2′), 128.64 (3′), 126.59 (4′), 126.52 (1″), 109.96 (6″), 108.20 (3″), 67.08 (d, *J*_2-P_ = 7.0 Hz, 2), 66.55 (4), 63.57 (d, *J*_1-P_ = 5.2 Hz, 1), 62.66 (d, *J*_CH2-P_ = 5.7 Hz, O**CH_2_**CH_3_), 56.55, and 56.44 (4″-O**CH_3_**, 5″-O**CH_3_**), 42.65 (NH**CH_2_**), 37.85 (d, *J*_CH2-P_ = 6.2 Hz, **CH_2_**Ph), 16.19 (d, *J*_CH3-P_ = 6.9 Hz, OCH_2_**CH_3_**). **^31^P-NMR** (162 MHz, CDCl_3_, 25 °C) δ 9.1 ppm. **HR-MS** (ESI) calculated for C_22_H_30_O_10_N_2_P 513.16326, found [M + H]^+^ 513.16284.

***4,5-dimethoxy-2-nitrobenzyl (2-((ethoxy(piperidin-1-yl)phosphoryl)oxy)ethyl) carbonate* (2)** was prepared from **19** (150.5 mg, 0.5 mmol, 1.0 eq.) and **20** (63.2 μL, 0.5 mmol, 1.0 eq.) according to the general procedure and purified by Flash silica gel chromatography using a gradient (CH_2_Cl_2_/MeOH 100:0→90:10, *v*/*v*), followed by reverse-phase chromatography using a gradient (H_2_O/MeCN 100:0→50:50, *v*/*v*), which afforded **2** as a dense yellow oil (103.7 mg, 0.22 mmol, 44%). **^1^H-NMR** (400 MHz, CDCl_3_, 25 °C) δ 7.74 (s, 1H, 3″), 7.09 (s, 1H, 6″), 5.62–5.60 (m, 2H, 4), 4.46–4.35 (m, 2H, 2), 4.28–4.12 (m, 2H, 1), 4.09–3.98 (m, 2H, O**CH_2_**CH_3_), 4.00 (s, 3H, 5″-O**CH_3_**), 3.97 (s, 3H, 4″-O**CH_3_**), 3.14–3.05 (m, 4H, 1′), 1.62–1.47 (m, 6H, 2′, 3′), 1.31 (td, *J*_CH3-CH2_ = 7.1 Hz, *J*_CH3-P_ = 0.8 Hz, OCH_2_**CH_3_**). **^13^C-NMR** (101 MHz, CDCl_3_, 25 °C) δ 154.58 (3), 153.73 (5″), 148.35 (4″), 139.65 (2″), 126.62 (1″), 109.89 (6″), 108.18 (3″), 67.14 (d, *J*_2-P_ = 7.4 Hz, 2), 66.47 (4), 63.38 (d, *J*_1-P_ = 5.2 Hz, 1), 62.39 (d, *J*_CH2-P_ = 5.9 Hz, O**CH_2_**CH_3_), 56.55 (5″-O**CH_3_**), 56.43 (4″-O**CH_3_**), 45.36 (d, *J*_1′-P_ = 2.3 Hz, 1′), 26.02 (d, *J*_2′-P_ = 5.1 Hz, 2′), 24.39 (d, *J*_3′-P_ = 1.5 Hz, 3′), 16.17 (d, *J*_CH3-CH2_ = 7.0 Hz, OCH_2_**CH_3_**). **^31^P-NMR** (162 MHz, CDCl_3_, 25 °C) δ 9.10 ppm. **HR-MS** (ESI) calculated for C_19_H_30_O_10_N_2_P 477.16326, found [M + H]^+^ 477.16340.

***4,5-dimethoxy-2-nitrobenzyl (2-((ethoxy(phenylamino)phosphoryl)oxy)ethyl) carbonate* (3)** was prepared from **19** (150.5 mg, 0.5 mmol, 1.0 eq.) and **20** (63.2 μL, 0.5 mmol, 1.0 eq.) according to the general procedure and purified by Flash silica gel chromatography using a gradient (CH_2_Cl_2_/MeOH 100:0→90:10, *v*/*v*), followed by two reverse-phase chromatographies using a gradient (H_2_O/MeCN 100:0→50:50, *v*/*v*), which afforded **3** as a dense yellow oil (82.8 mg, 34%). **^1^H-NMR** (400 MHz, CDCl_3_, 25 °C) δ 7.75 (s, 1H, 3″), 7.24 (m, 2H, 3′), 7.06 (s, 1H, 6″), 7.00 (m, 2H, 2′), 6.95 (m, 1H, 4′), 5.75 (d, *J*_NH-P_ = 9.0 Hz, NH), 5.56 (m, 2H, 4), 4.42 (m, 2H, 2), 4.41–4.25 (m, 2H, 1), 4.25–4.08 (m, 2H, O**CH_2_**CH_3_), 3.98–3.97 (m, 6H, 4″-O**CH_3_**, 5″-O**CH_3_**), 1.33 (td, *J*_CH3-CH2_ = 7.1 Hz, *J*_CH3-P_ = 0.8 Hz, OCH_2_**CH_3_**). **^13^C-NMR** (101 MHz, CDCl_3_, 25 °C) δ 154.49 (3), 153.76 and 148.34 (4″, 5″), 139.61 (2″), 139.16 (1′), 129.35 (3′), 126.58 (1″), 121.97 (4′), 117.45 (d, *J*_2′-P_ = 7.2 Hz, 2′), 109.82 (6″), 108.18 (3″), 66.78 (d, *J*_2-P_ = 7.3 Hz, 2), 66.55 (4), 64.00 (d, *J*_1-P_ = 4.6 Hz, 1), 63.31 (d, *J*_CH2-P_ = 5.0 Hz, O**CH_2_**CH_3_), 56.57 and 56.43 (4″-O**CH_3_**, 5″-O**CH_3_**), 16.07 (d, *J*_CH3-P_ = 7.0 Hz, OCH_2_**CH_3_**). **^31^P-NMR** (162 MHz, CDCl_3_, 25 °C) δ 2.31 ppm. **HR-MS** (ESI) calculated for C_20_H_26_O_10_N_2_P 485.13196, found [M + H]^+^ 485.13189.

***4,5-dimethoxy-2-nitrobenzyl (2-(((phenethylamino)(phenoxy)phosphoryl)oxy)ethyl) carbonate*(4)** was prepared from **19** (150.5 mg, 0.5 mmol, 1.0 eq.) and **21** (74.7 μL, 0.5 mmol, 1.0 eq.) according to the general procedure and purified by Flash silica gel chromatography using a gradient (hexane/EtOAc 100:0→0:100, *v*/*v*), followed by reverse-phase chromatography using a gradient (H_2_O/MeCN 100:0→0:100, *v*/*v*) and preparative HPLC chromatography using a gradient (H_2_O/MeCN 95:5 -> 20:80, *v*/*v*), which afforded **4** as a dense yellow oil (10.2 mg, 4%). **^1^H-NMR** (400 MHz, CDCl_3_, 25 °C) δ 7.74 (s, 1H, 3″), 7.35–7.12 (m, 10H, 2′, 3′, 4′, 2‴, 3‴, 4‴), 7.06 (s, 1H, 6″), 5.60 (s, 1H, 4a), 5.60 (s, 1H, 4b), 4.47–4.37 (m, 2H, 2), 4.35–4.21 (m, 2H, 1), 3.97 (s, 3H, 4″-O**CH_3_**), 3.94 (s, 3H, 5″-O**CH_3_**), 3.33–3.24 (m, 2H, NH**CH_2_**), 2.93–2.82 (m, 1H, NH), 2.77 (t, 2H, *J*_CH2Ph-CH2NH_ = 6.8 Hz, **CH_2_**Ph). **^13^C-NMR** (101 MHz, CDCl_3_, 25 °C) δ 154.55 (3), 153.73 (5″), 150.78 (d, *J*_1‴-P_ = 6.8 Hz, 1‴), 148.37 (4″), 139.65 (2″), 138.27 (1′), 129.69 (3′ or 3‴), 128.87 (2′), 128.67 (3′ or 3‴), 126.65 (4′), 126.50 (1″), 124.87 (4‴), 120.13 (d, *J*_2‴-P_ = 4.8 Hz, 2‴), 109.94 (6″), 108.19 (3″), 66.87 (d, *J*_2-P_ = 7.4 Hz, 2), 66.58 (4), 64.18 (d, *J*_1-P_ = 5.2 Hz, 1), 56.54 (5″-O**CH_3_**), 56.43 (4″-O**CH_3_**), 42.78 (NH**CH_2_**), 37.69 (d, *J*_CH2-P_ = 6.0 Hz, **CH_2_**Ph). **^31^P-NMR** (162 MHz, CDCl_3_, 25 °C) δ 4.42 ppm. **HR-MS** (ESI) calculated for C_26_H_30_O_10_N_2_P 561.16326, found [M + H]^+^ 561.16318.

***4,5-dimethoxy-2-nitrobenzyl (2-(((piperidin-1-yl)(phenoxy)phosphoryl)oxy)ethyl) carbonate*(5)** was prepared from **19** (150.5 mg, 0.5 mmol, 1.0 eq.) and **21** (74.7 μL, 0.5 mmol, 1.0 eq.) according to the general procedure and purified by Flash silica gel chromatography using a gradient (hexane/EtOAc 100:0→0:100, *v*/*v*), followed by reverse-phase chromatography using a gradient (H_2_O/MeCN 100:0→0:100, *v*/*v*) and preparative HPLC chromatography using a gradient (H_2_O/MeCN 95:5→0:100, *v*/*v*), which afforded **5** as a dense yellow oil (31.9 mg, 12%). **^1^H-NMR** (400 MHz, CDCl_3_, 25 °C) δ 7.75 (s, 1H, 3″), 7.32 (m, 2H, 3‴), 7.22 (m, 2H, 2‴), 7.14 (m, 1H, 4‴), 7.09 (s, 1H, 6″), 5.62 (s, 1H, 4a), 5.62 (s, 1H, 4b), 4.47–4.43 (m, 2H, 2), 4.35–4.28 (m, 2H, 1), 3.99 (s, 3H, 5″-O**CH_3_**), 3.98 (s, 3H, 4″-O**CH_3_**), 3.20–3.12 (m, 4H, 1′), 1.60–1.51 (m, 2H, 3′), 1.51–1.44 (m, 4H, 2′). **^13^C-NMR** (101 MHz, CDCl_3_, 25 °C) δ 154.57 (3), 153.75 (5″), 150.98 (d, *J*_1‴-P_ = 6.8 Hz, 1‴), 148.36 (4″), 139.64 (2″), 129.60 (3‴), 126.61 (1″), 124.62 (4″), 120.07 (d, *J*_2‴-P_ = 5.2 Hz, 2‴), 109.90 (6″), 108.18 (3″), 66.96 (d, *J*_2-P_ = 7.3 Hz, 2), 66.53 (4), 63.98 (d, *J*_1-P_ = 5.3 Hz, 1), 56.55 and 56.44 (4″-O**CH_3_**, 5″-O**CH_3_**), 45.49(d, *J*_1′-P_ = 2.3 Hz, 1′), 25.82 (d, *J*_2′-P_ = 4.7 Hz, 2′), 25.26 (3′). **^31^P-NMR** (162 MHz, CDCl_3_, 25 °C) δ 4.27 ppm. **HR-MS** (ESI) calculated for C_23_H_30_O_10_N_2_P 525.16326, found [M + H]^+^ 525.16278.

***4,5-dimethoxy-2-nitrobenzyl (2-(((phenylamino)(phenoxy)phosphoryl)oxy)ethyl) carbonate*(6)** was prepared from **19** (150.5 mg, 0.5 mmol, 1.0 eq.) and **21** (74.7 μL, 0.5 mmol, 1.0 eq.) according to the general procedure and purified by Flash silica gel chromatography using a gradient (hexane/EtOAc 100:0→0:100, *v*/*v*), followed by reverse-phase chromatography using a gradient (H_2_O/MeCN 100:0→0:100, *v*/*v*) and preparative HPLC chromatography using a gradient (H_2_O/MeCN 95:5→0:100, *v*/*v*), which afforded **6** as a dense yellow oil (18.1 mg, 7%). **^1^H-NMR** (400 MHz, CDCl_3_, 25 °C) δ 7.74 (s, 1H, 3″), 7.28–7.22 (m, 4H, 3′, 3‴), 7.17–7.11 (m, 3H, 2‴, 4‴), 7.07 (m, 2H, 2′), 7.05 (s, 1H, 6″), 6.99 (m, 1H, 4′), 6.25 (d, *J*_NH-P_ = 9.8 Hz, NH), 5.62–5.52 (m, 2H, 4), 4.49–4.33 (m, 4H, 1, 2), 3.97 (s, 3H, 4″-O**CH_3_**), 3.93 (s, 3H, 5″-O**CH_3_**). **^13^C-NMR** (101 MHz, CDCl_3_, 25 °C) δ 154.47 (3), 153.77 (5″), 150.15 (d, *J*_1‴-P_ = 6.1 Hz, 1‴), 148.32 (4″), 139.56 (2″), 138.79 (1′), 129.70 (3‴), 129.39 (3′), 126.58 (1″), 125.30 (4‴), 122.33 (4′), 120.38 (d, *J*_2‴-P_ = 4.8 Hz, 2‴), 117.87 (d, *J*_2′-P_ = 7.4 Hz, 2′), 109.77 (6″), 108.16 (3″), 66.61 (d, *J*_2-P_ = 7.0 Hz, 2), 66.59 (4), 64.53 (d, *J*_1-P_ = 4.6 Hz, 1), 56.53 (5″-O**CH_3_**), 56.42 (4″-O**CH_3_**). **^31^P-NMR** (162 MHz, CDCl_3_, 25 °C) δ −1.97 ppm. **HR-MS** (ESI) calculated for C_24_H_26_O_10_N_2_P 533.13196, found [M + H]^+^ 533.13188.

***4,5-dimethoxy-2-nitrobenzyl (2S)-2-((ethoxy(phenethylamino)phosphoryl)oxy)propanoate* (7)** was prepared from **24** (142.6 mg, 0.5 mmol, 1.0 eq.) and **20** (63.2 μL, 0.5 mmol, 1.0 eq.) according to the general procedure and purified by Flash silica gel chromatography using a gradient (hexane/EtOAc 50:50→0:100, *v*/*v*), followed by reverse-phase chromatography using a gradient (H_2_O/MeOH 100:0→0:100, *v*/*v*), which afforded **7** as a dense yellow oil (129.3 mg, 52%). **^1^H-NMR** (400 MHz, CDCl_3_, 25 °C, 2 diastereoisomers in ca. 1:1 ratio) δ 7.73 and 7.72 (s, 2H, 3″), 7.33–7.13 (m, 10H, 2′, 3′, 4′), 7.07 and 7.03 (s, 2H, 6″), 5.66–5.53 (m, 4H, 4), 5.02–4.88 (m, 2H, 1), 4.14–3.94 (m, 4H, O**CH_2_**CH_3_), 4.00 and 3.98 (s, 6H, 5″-O**CH_3_**), 3.96 and 3.96 (s, 6H, 4″-O**CH_3_**), 3.26–3.16 (m, 4H, NH**CH_2_**), 2.83–2.67 (m, 6H, **CH_2_**Ph, NH), 1.61 (d, 3H, *J*_2-1_ = 6.8 Hz), 1.56 (d, 3H, *J*_2-1_ = 6.9 Hz, 2), 1.32 (td, 3H, *J*_CH3-CH2_ = 7.0 Hz, *J*_CH3-P_ = 0.8 Hz, OCH_2_**CH_3_**), 1.28 (td, 3H, *J*_CH3-CH2_ = 7.2 Hz, *J*_CH3-P_ = 0.7 Hz, OCH_2_**CH_3_**). **^13^C-NMR** (101 MHz, CDCl_3_, 25 °C) δ 170.68–170.47 (m, 3), 153.65 and 153.63 (5″), 148.40 and 148.37 (4″), 139.84 (2″), 138.55 and 138.50 (1′), 128.86 and 128.83 (2′), 128.65 and 128.58 (3′), 126.53 and 126.43 (4′), 126.32 and 126.29 (1″), 110.47 and 110.35 (6″), 108.26 and 108.23 (3″), 70.57 (d, *J*_1-P_ = 7.6 Hz, 1), 70.52 (d, *J*_1-P_ = 7.6 Hz, 1), 64.09 and 64.06 (4), 62.93 (d, *J*_CH2-P_ = 5.7 Hz, O**CH_2_**CH_3_), 62.79 (d, *J*_CH2-P_ = 5.7 Hz, O**CH_2_**CH_3_), 56.68 and 56.62 (4″-O**CH_3_**), 56.42 (5″-O**CH_3_**), 42.65 and 42.54 (NH**CH_2_**), 37.88–37.70 (m, **CH_2_**Ph), 19.42 (d, *J*_2-P_ = 5.5 Hz, 2), 19.41 (d, *J*_2-P_ = 5.3 Hz, 2), 16.18 (d, *J*_CH3-P_ = 7.0 Hz, OCH_2_**CH_3_**), 16.13 (d, *J*_CH3-P_ = 7.0 Hz, OCH_2_**CH_3_**). **^31^P-NMR** (162 MHz, CDCl_3_, 25 °C) δ 9.07 and 8.39 ppm. **HR-MS** (ESI) calculated for C_22_H_29_O_9_N_2_NaP 519.15029, found [M + Na]^+^ 519.14986.

***4,5-dimethoxy-2-nitrobenzyl (2S)-2-((ethoxy(piperidin-1-yl)phosphoryl)oxy)propanoate* (8)** was prepared from **24** (142.6 mg, 0.5 mmol, 1.0 eq.) and **20** (63.2 μL, 0.5 mmol, 1.0 eq.) according to the general procedure and purified by Flash silica gel chromatography using a gradient (CH_2_Cl_2_/MeOH 100:0→80:20, *v*/*v*), followed by reverse-phase chromatography using a gradient (H_2_O/MeCN 100:0→0:100, *v*/*v*), which afforded **8** as a dense yellow oil (97.0 mg, 42%). **^1^H-NMR** (400 MHz, CDCl_3_, 25 °C, 2 diastereoisomers in ca. 1:1 ratio) δ 7.74 and 7.73 (s, 2H, 3″), 7.10 and 7.09 (s, 2H, 6″), 5.64 (dd, 1H, *J*_Gem_ = 14.8 Hz, *J*_4a-6″_ = 0.6 Hz, 4a), 5.63 (dd, 1H, *J*_Gem_ = 14.8 Hz, *J*_4a-6″_ = 0.6 Hz, 4a), 5.59 (dd, 1H, *J*_Gem_ = 14.8 Hz, *J*_4b-6″_ = 0.6 Hz, 4b), 5.58 (dd, 1H, *J*_Gem_ = 14.8 Hz, *J*_4b-6″_ = 0.6 Hz, 4b), 5.00–4.88 (m, 1H, 1), 4.12–3.99 (m, 2H, O**CH_2_**CH_3_), 4.02 and 4.02 (s, 6H, 5″-O**CH_3_**), 3.97 and 3.97 (s, 6H, 4″-O**CH_3_**), 3.19–3.01 (m, 4H, 1′), 1.61 (d, *J*_2-P_ = 6.9 Hz, 3H, 2), 1.59 (d, *J*_2-P_ = 6.9 Hz, 3H, 2), 1.57–1.44 (m, 10H, 2′, 3′), 1.33 (td, 3H, *J*_CH3-CH2_ = 7.1 Hz, *J*_CH3-P_ = 0.8 Hz, OCH_2_**CH_3_**), 1.28 (td, 3H, *J*_CH3-CH2_ = 7.1 Hz, *J*_CH3-P_ = 0.8 Hz, OCH_2_**CH_3_**). **^13^C-NMR** (101 MHz, CDCl_3_, 25 °C) δ 170.71 (d, *J*_3-P_ = 4.4 Hz, 3), 170.51 (d, *J*_3-P_ = 5.7 Hz, 3), 153.70 and 153.68 (5″), 148.28 (4″), 139.74 (2″), 126.66 and 126.60 (1″), 110.37 and 110.24 (6″), 108.17 (3″), 70.33 (d, *J*_1-P_ = 5.1 Hz, 1), 70.18 (d, *J*_1-P_ = 5.2 Hz, 1), 63.92 and 63.89 (4), 62.60 (d, *J*_CH2-P_ = 5.9 Hz, O**CH_2_**CH_3_), 62.42 (d, *J*_CH2-P_ = 5.8 Hz, O**CH_2_**CH_3_), 56.66 and 56.61 and 56.39–56.36 (m) (4″-O**CH_3_**, 5″-O**CH_3_**), 45.34 and 45.32 (1′), 25.96 (d, *J*_2′-P_ = 4.7 Hz, 2′), 25.84 (d, *J*_2′-P_ = 5.4 Hz, 2′), 24.33 (d, *J*_3′-P_ = 1.5 Hz, 3′), 24.29 (d, *J*_3′-P_ = 1.5 Hz, 3′), 19.45 (d, *J*_2-P_ = 4.8 Hz, 2), 19.41 (d, *J*_2-P_ = 5.2 Hz, 2), 16.17–16.01 (m, OCH_2_**CH_3_**). **^31^P-NMR** (162 MHz, CDCl_3_, 25 °C) δ 8.81 and 8.30 ppm. **HR-MS** (ESI) calculated for C_19_H_29_O_9_N_2_NaP 483.15029, found [M + Na]^+^ 483.14977.

***4,5-dimethoxy-2-nitrobenzyl (2S)-2-((ethoxy(phenylamino)phosphoryl)oxy)propanoate* (9)** was prepared from **24** (142.6 mg, 0.5 mmol, 1.0 eq.) and **20** (63.2 μL, 0.5 mmol, 1.0 eq.) according to the general procedure and purified by Flash silica gel chromatography using a gradient (hexane/EtOAc 100:0→0:100, *v*/*v*), followed by reverse-phase chromatography using a gradient (H_2_O/MeCN 100:0→0:100, *v*/*v*), which afforded **9** as a dense yellow oil (108.2 mg, 46%). **^1^H-NMR** (400 MHz, CDCl_3_, 25 °C, 2 diastereoisomers in ca. 1:1 ratio) δ 7.73 and 7.64 (s, H, 3″), 7.22 and 7.17 (m, 4H, 3′), 7.08 and 6.94 (s, 2H, 6″), 7.06–6.96 (m, 4H, 2′), 6.96–6.89 (m, 2H, 4′), 6.35 (d, 1H, *J*_NH-P_ = 9.3 Hz, NH), 6.22 (d, 1H, *J*_NH-P_ = 8.7 Hz, NH), 5.65 (dd, 1H, *J*_Gem_ = 14.9 Hz, *J*_4a-6″_ = 0.6 Hz, 4a), 5.57 (dd, 1H, *J*_Gem_ = 14.9 Hz, *J*_4b-6″_ = 0.5 Hz, 4b), 5.56–5.41 (m, 2H, 4a, 4b), 5.12 and 5.06 (m, 2H, 1), 4.29–4.07 (m, 4H, O**CH_2_**CH_3_), 3.97–3.95 (m, 6H, 4″-O-**CH_3_**), 3.95 and 3.90 (s, 6H, 5″-O**CH_3_**), 1.67 (d, 3H, *J*_2-1_ = 6.9 Hz, 2), 1.52 (d, 3H, *J*_2-1_ = 6.9 Hz, 2), 1.36–1.26 (m, 6H, OCH_2_**CH_3_**). **^13^C-NMR** (101 MHz, CDCl_3_, 25 °C) δ 170.13 (d, *J*_3-P_ = 4.6 Hz, 3), 170.10 (d, *J*_3-P_ = 5.4 Hz, 3), 153.64 and 153.55 (5″), 148.28 and 148.26 (4″), 139.69 and 139.67 (2″), 139.20 (d, *J*_1′-P_ = 4.7 Hz, 1′), 129.19 and 129.10 (3′), 126.40 and 126.16 (1″), 121.88 and 121.84 (4′), 117.63 (d, *J*_2′-P_ = 7.7 Hz, 2′), 117.55 (d, *J*_2′-P_ = 7.3 Hz, 2′), 110.30 and 110.17 (6″), 108.15 and 108.11 (3″), 71.25 (d, *J*_1-P_ = 4.6 Hz, 1), 70.92 (d, *J*_1-P_ = 4.7 Hz, 1), 64.12 and 64.09 (4), 63.46 (d, *J*_CH2-P_ = 5.4 Hz, O**CH_2_**CH_3_), 63.36 (d, *J*_CH2-P_ = 5.6 Hz, O**CH_2_**CH_3_), 56.53, 56.40, 56.35, and 56.33 (4″-O**CH_3_**, 5″-O**CH_3_**), 19.30 (d, *J*_2-P_ = 5.1 Hz, 2), 19.12 (d, *J*_2-P_ = 6.0 Hz, 2),15.98 (d, *J*_CH3-P_ = 7.3 Hz, OCH_2_**CH_3_**), 15.95 (d, *J*_CH3-P_ = 7.3 Hz, OCH_2_**CH_3_**). **^31^P-NMR** (162 MHz, CDCl_3_, 25 °C) δ 2.14 and 1.86 ppm. **HR-MS** (ESI) calculated for C_20_H_25_O_9_N_2_NaP 491.11899, found [M + Na]^+^ 491.11862.

***4,5-dimethoxy-2-nitrobenzyl (2S)-2-((ethoxy(N-methylphenyl)phosphoryl)oxy)propanoate* (10)** was prepared from **24** (142.6 mg, 0.5 mmol, 1.0 eq.) and **20** (63.2 μL, 0.5 mmol, 1.0 eq.) according to the general procedure and purified by Flash silica gel chromatography using a gradient (hexane/EtOAc 100:0→0:100, *v*/*v*), followed by two reverse-phase chromatographies using a gradient (H_2_O/MeCN 100:0 -> 50:50, *v*/*v*), which afforded **10** as a dense yellow oil (44.5 mg, 18%). **^1^H-NMR** (400 MHz, CDCl_3_, 25 °C, 2 diastereoisomers in ca. 1:1 ratio) δ 7.74 and 7.73 (s, 2H, 3″), 7.33–7.30 (m, 2H, 2′), 7.30–7.22 (m, 6H, 2′, 3′), 7.14–7.04 (m, 2H, 4′), 7.10 and 7.00 (s, 2H, 6″), 5.65–5.62 and 5.56–5.54 (m, 4H, 4), 5.11–4.96 (m, 2H, 1), 4.23–4.00 (m, 4H, O**CH_2_**CH_3_), 3.99 and 3.98 and 3.98 (s, 9H, 4″-O**CH_3_**, 5″-O**CH_3_**), 3.91 (s, 3H, 5″-O**CH_3_**), 3.22 (d, 3H, *J*_CH3-P_ = 9.0 Hz, N**CH_3_**), 3.17 (d, 3H, *J*_CH3-P_ = 9.1 Hz, N**CH_3_**), 1.65 (d, 3H, *J*_2-1_ = 7.0 Hz, 2), 1.51 (d, 3H, *J*_2-1_ = 7.0 Hz, 2), 1.30 (td, 3H, *J*_CH3-CH2_ = 7.0 Hz, *J*_CH3-P_ = 0.8 Hz, OCH_2_**CH_3_**), 1.28 (dt, 3H, *J*_CH3-CH2_ = 7.0 Hz, *J*_CH3-P_ = 0.8 Hz, OCH_2_**CH_3_**). **^13^C-NMR** (101 MHz, CDCl_3_, 25 °C) δ 170.4* and 170.2* (3), 153.8* (5″), 148.3* (4″), 143.9* (1′), 139.8* and 139.7* (2″), 128.97 and 128.85 (3′), 126.67 and 126.65 (1″), 124.27 and 124.19 (4′), 123.13 (d, *J*_2′-P_ = 4.6 Hz, 2′), 122.69 (d, *J*_2′-P_ = 3.9 Hz, 2′), 110.33 and 110.11 (6″), 108.21 and 108.15 (3″), 71.15 (d, *J*_1-P_ = 5.4 Hz, 1), 70.90 (d, *J*_1-P_ = 4.9 Hz, 1), 64.08 and 64.04 (4), 63.34 (d, *J*_CH2-P_ = 5.7 Hz, O**CH_2_**CH_3_), 62.96 (d, *J*_CH2-P_ = 5.6 Hz, O**CH_2_**CH_3_), 56.69 and 56.58 and 56.49–56.33 (m, 4″-O**CH_3_**, 5″-O**CH_3_**), 37.35–36.95 (m, N**CH_3_**), 19.37 (d, *J*_2-P_ = 4.6 Hz, 2), 19.16 (d, *J*_2-P_ = 5.4 Hz, 2), 15.99 (d, *J*_CH3-P_ = 6.7 Hz, OCH_2_**CH_3_**). * The ^13^C chemical shift was extracted from HMBC. **^31^P-NMR** (162 MHz, CDCl_3_, 25 °C) δ 5.82 and 5.55 ppm. **HR-MS** (ESI) calculated for C_21_H_27_O_9_N_2_NaP 505.13464, found [M + Na]^+^ 505.13472. 

***4,5-dimethoxy-2-nitrobenzyl (2S)-2-((ethoxy(morpholin-1-yl)phosphoryl)oxy)propanoate* (11)** was prepared from **24** (142.6 mg, 0.5 mmol, 1.0 eq.) and **20** (63.2 μL, 0.5 mmol, 1.0 eq.) according to the general procedure and purified by Flash silica gel chromatography using a gradient (hexane/EtOAc 100:0→0:100, *v*/*v*), followed by two reverse-phase chromatographies using a gradient (H_2_O/MeCN 100:0→60:40, *v*/*v*), which afforded **11** as a dense yellow oil (80.0 mg, 35%). **^1^H-NMR** (400 MHz, CDCl_3_, 25 °C, 2 diastereoisomers in ca. 1:1 ratio) δ 7.72–7.70 (m, 2H, 3″), 7.04 and 7.02 (s, 2H, 6″), 5.64–5.51 (m, 4H, 4), 5.00–4.87 (m, 2H, 1), 4.13–4.01 (m, 4H, O**CH_2_**CH_3_), 3.99 and 3.99 (s, 6H, 5″-O**CH_3_**), 3.96–3.94 (m, 6H, 4″-O**CH_3_**), 3.64 and 3.59 (m, 8H, 2′), 3.19–3.04 (m, 8H, 1′), 1.59 (dd, 6H, *J*_2-1_ = 6.9 Hz, *J*_2-P_ = 1.0 Hz, 2), 1.32 (td, 3H, *J*_CH3-CH2_ = 7.1 Hz, *J*_CH3-P_ = 0.9 Hz, OCH_2_**CH_3_**), 1.28 (td, 3H, *J*_CH3-CH2_ = 7.1 Hz, *J*_CH3-P_ = 0.8 Hz, OCH_2_**CH_3_**). **^13^C-NMR** (101 MHz, CDCl_3_, 25 °C) δ 170.41 (d, *J*_3-P_ = 4.6 Hz, 3), 170.27 (d, *J*_3-P_ = 4.9 Hz, 3), 153.55 and 153.52 (5″), 148.37 and 148.33 (4″), 139.85 and 139.82 (2″), 126.21 and 126.08 (1″), 110.53 and 110.49 (6″), 108.20 and 108.15 (3″), 70.49 (d, *J*_1-P_ = 5.0 Hz, 1), 70.40 (d, *J*_1-P_ = 5.3 Hz, 1), 66.83 (d, *J*_2′-P_ = 5.8 Hz, 2′), 66.73 (d, *J*_2′-P_ = 6.1 Hz, 2′), 64.02 and 64.00 (4), 62.95 (d, *J*_CH2-P_ = 6.1 Hz, O**CH_2_**CH_3_), 62.74 (d, *J*_CH2-P_ = 6.1 Hz, O**CH_2_**CH_3_), 56.56 and 56.52 (5″-O**CH_3_**), 56.35–56.30 (m, 4″-O**CH_3_**), 44.49–44.43 (m, 1′), 19.34 (d, *J*_2-P_ = 4.7 Hz, 2), 19.33 (d, *J*_2-P_ = 5.5 Hz, 2), 16.03 (d, *J*_CH3-P_ = 7.0 Hz, OCH_2_**CH_3_**), 15.98 (d, *J*_CH3-P_ = 6.7 Hz, OCH_2_**CH_3_**). **^31^P-NMR** (162 MHz, CDCl_3_, 25 °C) δ 7.68 and 7.13 ppm. **HR-MS** (ESI) calculated for C_18_H_28_O_10_N_2_P 463.14761, found [M + H]^+^ 463.14720.

***4,5-dimethoxy-2-nitrobenzyl (2S)-2-((ethoxy(benzylamino)phosphoryl)oxy)propanoate* (12)** was prepared from **24** (142.6 mg, 0.5 mmol, 1.0 eq.) and **20** (63.2 μL, 0.5 mmol, 1.0 eq.) according to the general procedure and purified by Flash silica gel chromatography using a gradient (hexane/EtOAc 50:50→0:100, *v*/*v*), followed by reverse-phase chromatography using a gradient (H_2_O/MeOH 100:0→0:100, *v*/*v*), which afforded **12** as a dense yellow oil (101 mg, 42%). **^1^H-NMR** (400 MHz, CDCl_3_, 25 °C, 2 diastereoisomers in ca. 1:1 ratio) δ 7.73 and 7.71 (s, 2H, 3″), 7.36–7.22 (m, 10H, 2′, 3′, 4′), 7.07 and 7.02 (s, 2H, 6″), 5.63 (d, 1H, *J*_Gem_ = 14.6 Hz, 4a), 5.58 (d, *J*_Gem_ = 14.6 Hz, 1H, 4b), 5.56 (d, 1H, *J*_Gem_ = 14.7 Hz, 4a), 5.51 (d, 1H, *J*_Gem_ = 14.7 Hz, 4b), 5.04 and 4.97 (m, 2H, 1), 4.17–4.05 (m, 8H, NH**CH_2,_** O**CH_2_**CH_3_), 4.00 and 3.98 (s, 6H, 5″-O**CH_3_**), 3.96 and 3.96 (s, 6H, 4″-O**CH_3_**), 3.12 (m, 2H, NH), 1.63 (d, 3H, *J*_2-1_ = 6.9 Hz, 2), 1.54 (d, 3H, *J*_2-1_ = 6.9 Hz, 2), 1.32 (tm, *J*_CH3-CH2_= 7.1 Hz, 3H, OCH_2_**CH_3_**), 1.29 (tm, *J*_CH3-CH2_= 7.1 Hz, 3H, OCH_2_**CH_3_**). **^13^C-NMR** (101 MHz, CDCl_3_, 25 °C) δ 170.69–170.57 (m, 3), 153.66 and 153.61 (5″), 148.35 and 148.32 (4″), 139.80 (2″), 13942–139.30 (m, 1′), 128.56 and 128.48 (3′), 127.41, 127.30, and 127.21 (2′, 4′), 126.43 and 126.23 (1″), 110.46 and 110.37 (6″), 108.19 (3″), 70.67 (d, *J*_1-P_ = 4.7 Hz, 1), 70.60 (d, *J*_1-P_ = 5.1 Hz, 1), 64.07 and 64.04 (4), 63.06 (d, *J*_CH2-P_ = 5.4 Hz, O**CH_2_**CH_3_), 62.91 (d, *J*_CH2-P_ = 5.4 Hz, O**CH_2_**CH_3_), 56.63 and 56.55 (5″-O**CH_3_**), 56.37 (4″-O**CH_3_**), 45.23 (d, *J*_CH2-P_ = 6.2 Hz, NH**CH_2_**), 19.34 (d, *J*_2-P_ = 5.4 Hz, 2), 19.28 (d, *J*_2-P_ = 5.9 Hz, 2), 16.08 (d, *J*_CH3-P_ = 7.4 Hz, OCH_2_**CH_3_**), 16.04 (d, *J*_CH3-P_ = 6.9 Hz, OCH_2_**CH_3_**). **^31^P-NMR** (162 MHz, CDCl_3_, 25 °C) δ 8.61 and 7.87 ppm. **HR-MS** (ESI) calculated for C_21_H_27_O_9_N_2_NaP 505.13464, found [M + Na]^+^ 505.13456.

***4,5-dimethoxy-2-nitrobenzyl (2S)-2-((ethoxy(pyrimidin-2-ylamino)phosphoryl)oxy)propanoate* (13)** was prepared from **24** (142.6 mg, 0.5 mmol, 1.0 eq.) and **20** (63.2 μL, 0.5 mmol, 1.0 eq.) according to the general procedure and purified by Flash silica gel chromatography using a gradient (CH_2_Cl_2_/MeOH 100:0→90:10, *v*/*v*), followed by reverse-phase chromatography using a gradient (H_2_O/MeCN 100:0→0:100, *v*/*v*), which afforded **13** as a dense yellow oil (67.0 mg, 29%). **^1^H-NMR** (400 MHz, CDCl_3_, 25 °C, 2 diastereoisomers in ca. 1:1 ratio) δ 8.53 (d, 1H, *J*_2′-P_ = 4.9 Hz, 2′), 8.47 (d, 1H, *J*_2′-P_ = 4.9 Hz, 2′), 7.73 and 7.71 (s, 2H, 3″), 7.13 and 7.07 (s, 2H, 6″), 6.88 (t, 2H, *J*_3′-P_ = 4.9 Hz, 3′), 6.85 (t, 2H, *J*_3′-P_ = 4.9 Hz, 3′), 5.64 (dd, 1H, *J*_Gem_ = 15.0 Hz, *J*_4a-6″_ = 0.5 Hz, 4a), 5.59 (dd, 1H, *J*_Gem_ = 15.0 Hz, *J*_4b-6″_ = 0.5 Hz, 4b), 5.56 (dd, 1H, *J*_Gem_ = 14.9 Hz, *J*_4a-6″_ = 0.5 Hz, 4a), 5.51 (dd, 1H, *J*_Gem_ = 14.9 Hz, *J*_4b-6″_ = 0.5 Hz, 4b), 5.43–5.29 (m, 2H, 1), 4.39–4.18 (m, 4H, O**CH_2_**CH_3_), 4.02 and 3.98 (s, 6H, 5″-O-**CH_3_**), 3.96 and 3.96 (s, 6H, 4″-O**CH_3_**), 1.66 (dd, 3H, *J*_2-1_ = 6.9 Hz, *J*_2-P_ = 0.4 Hz, 2), 1.59 (dd, 3H, *J*_2-1_ = 7.0 Hz, *J*_2-P_ = 0.5 Hz, 2), 1.35 (td, 3H, *J*_CH3-CH2_= 7.1 Hz, *J* _CH3-P_ = 0.9 Hz, OCH_2_**CH_3_**), 1.34 (td, 3H, *J*_CH3-CH2_= 7.1 Hz, *J* _CH3-P_ = 0.9 Hz, OCH_2_**CH_3_**). **^13^C-NMR** (101 MHz, CDCl_3_, 25 °C) δ 170.73 (d, *J*_3-P_ = 3.9 Hz, 3), 170.40 (d, *J*_3-P_ = 4.6 Hz, 3), 159.22 (d, *J*_1′-P_ = 6.2 Hz, 1′), 159.11 (d, *J*_1′-P_ = 5.9 Hz, 1′), 158.38 (d, *J*_2′-P_ = 1.5 Hz, 2′), 158.30 (d, *J*_2′-P_ = 1.5 Hz, 2′), 153.81 and 153.65 (5″), 148.28 and 148.22 (4″), 139.67 and 139.56 (2″), 126.70 and 126.27 (1″), 114.56 and 114.51 (3′), 110.28 and 110.11 (6″), 108.12 and 108.11 (3″), 72.29 (d, *J*_1-P_ = 5.4 Hz, 1), 72.19 (d, *J*_1-P_ = 5.1 Hz, 1), 64.06 and 64.03 (4), 63.94 (d, *J*_CH2-P_ = 5.5 Hz, O**CH_2_**CH_3_), 56.76 and 56.59 (5″-O**CH_3_**), 56.39–56.32 (m, 4″-O**CH_3_**), 19.37 (d, *J*_2-P_ = 5.9 Hz, 2), 19.05 (d, *J*_2-P_ = 6.7 Hz, 2), 16.07 (d, *J*_CH3-P_ = 7.1 Hz, OCH_2_**CH_3_**), 16.03 (d, *J*_CH3-P_ = 7.1 Hz, OCH_2_**CH_3_**). **^31^P-NMR** (162 MHz, CDCl_3_, 25 °C) δ −0.87 ppm. **HR-MS** (ESI) calculated for C_18_H_24_O_9_N_4_P 471.12754, found [M + H]^+^ 471.12723.

***4,5-dimethoxy-2-nitrobenzyl (2S)-2-((phenoxy(phenethylamino)phosphoryl)oxy)propanoate*(14)** was prepared from **24** (142.6 mg, 0.5 mmol, 1.0 eq.) and **21** (74.7 μL, 0.5 mmol, 1.0 eq.) according to the general procedure and purified by Flash silica gel chromatography using a gradient (hexane/EtOAc 100:0→0:100, *v*/*v*), followed by reverse-phase chromatography using a gradient (H_2_O/MeCN 100:0→50:50, *v*/*v*), which afforded **14** as a dense yellow oil (79.4 mg, 29%). **^1^H-NMR** (400 MHz, CDCl_3_, 25 °C, 2 diastereoisomers in ca. 1:0.8 ratio) δ 7.74 and 7.71 (s, 2H, 3″), 7.38–7.11 (m, 20H, 2′, 3′, 4′, 2‴, 3‴, 4‴), 7.04 and 7.02 (s, 2H, 6″), 5.63 (dd, *J*_Gem_ = 14.8 Hz, *J*_4a-6″_ = 0.5 Hz, 1H, 4a), 5.63 (dd, 1H, *J*_Gem_ = 14.7 Hz, *J*_4a-6″_ = 0.5 Hz, 4a), 5.58 (dd, 1H, *J*_Gem_ = 14.7 Hz, *J*_4b-6″_ = 0.5 Hz, 4b), 5.55 (dd, 1H, *J*_Gem_ = 14.8 Hz, *J*_4b-6″_ = 0.5 Hz, 4b), 5.11–5.01 (m, 2H, 1), 3.98 (s, 3H, 4″-O**CH_3_**), 3.97 (s, 3H, 5″-O**CH_3_**), 3.96 (s, 3H, 4″-O**CH_3_**), 3.89 (s, 3H, 5″-O**CH_3_**), 3.39–3.23 (m, 4H, NH**CH_2_**), 2.96–2.84 (m, 2H, NH), 2.80–2.74 (m, 4H, **CH_2_**Ph), 1.64 (d, 3H, *J*_2-1_ = 6.9 Hz, 2), 1.57 (dd, 3H, *J*_2-1_ = 7.0 Hz, *J*_2-P_ = 0.3 Hz, 2). **^13^C-NMR** (101 MHz, CDCl_3_, 25 °C) δ 170.37 (d, *J*_3-P_ = 4.5 Hz, 3), 170.15 (d, *J*_3-P_ = 4.3 Hz, 3), 153.72 and 153.71 (5″), 150.8* (1‴), 148.44 and 148.32 (4″), 139.87 and 139.77 (2″), 138.29 and 138.18 (1′), 129.69 and 129.62 (3′), 128.86 and 128.84 (2′), 128.71 and 128.63 (3‴), 126.71 and 126.60 (4‴), 126.45 and 126.30 (1″), 124.97 and 124.92 (4′), 124.97 and 124.92 (2′), 120.27 (d, *J*_2‴-P_ = 4.9 Hz, 2‴), 120.11 (d, *J*_2‴-P_ = 5.1 Hz, 2‴), 110.40 and 110.35 (6″), 108.28 and 108.16 (3″), 71.27 (d, *J*_1-P_ = 4.9 Hz, 1), 71.25 (d, *J*_1-P_ = 5.3 Hz, 1), 64.22 and 64.12 (4), 56.64, 56.56, 56.44, and 56.41 (4″-O**CH_3_**, 5″-O**CH_3_**), 42.77 and 42.66 (NH**CH_2_**), 37.64 (d, *J*_CH2-P_ = 5.9 Hz, **CH_2_**Ph), 37.61 (d, *J*_CH2-P_ = 6.1 Hz, **CH_2_**Ph), 19.44 (d, *J*_2-P_ = 5.5 Hz, 2) 19.21 (d, *J*_2-P_ = 5.8 Hz, 2). * The ^13^C chemical shift was extracted from HMBC. **^31^P-NMR** (162 MHz, CDCl_3_, 25 °C) δ 4.40 and 3.55 ppm. **HR-MS** (ESI) calculated for C_26_H_29_O_9_N_2_NaP 567.15029, found [M + Na]^+^ 567.14944.

***4,5-dimethoxy-2-nitrobenzyl (2S)-2-((phenoxy(piperidin-1-yl)phosphoryl)oxy)propanoate*(15)** was prepared from **24** (142.6 mg, 0.5 mmol, 1.0 eq.) and **21** (74.7 μL, 0.5 mmol, 1.0 eq.) according to the general procedure and purified by Flash silica gel chromatography using a gradient (CH_2_Cl_2_/MeOH 100:0→90:10, *v*/*v*), followed by two reverse-phase chromatographies using a gradient (H_2_O/MeCN 100:0→60:40, *v*/*v*), which afforded **15** as a dense yellow oil (75.5 mg, 30%). **^1^H-NMR** (400 MHz, CDCl_3_, 25 °C, 2 diastereoisomers in ca. 1:0.7 ratio) δ 7.75 and 7.72 (s, 2H, 3″), 7.34 and 7.27 (m, 4H, 3‴), 7.23 and 7.18 (m, 4H, 2‴), 7.17–7.10 (m, 2H, 4‴), 7.09 and 7.05 (s, 2H, 6″), 5.67–5.55 (m, 4H, 4), 5.12–5.01 (m, 2H, 1), 4.00 (s, 3H, 5″-O**CH_3_**), 3.98 (s, 3H, 4″-O**CH_3_**), 3.96 (s, 3H, 4″-O**CH_3_**), 3.88 (s, 3H, 5″-O**CH_3_**), 3.24–3.12 (m, 8H, 1′), 1.66 (d, 3H, *J*_2-1_ = 6.9 Hz, 2), 1.58 (d, 3H, *J*_2-1_ = 6.9 Hz, 2), 1.59–1.52 (m, 4H, 3′), 1.52–1.40 (m, 8H, 2′). **^13^C-NMR** (101 MHz, CDCl_3_, 25 °C) δ 170.32 (d, *J*_3-P_ = 4.7 Hz, 3), 170.27 (d, *J*_3-P_ = 4.6 Hz, 3), 153.77 and 153.75 (5″), 150.94 (d, *J*_1‴-P_ = 6.9 Hz, 1‴), 150.86 (d, *J*_1‴-P_ = 6.6 Hz, 1‴), 148.36 and 148.25 (4″), 139.78 and 139.68 (2″), 129.60 and 129.53 (3‴), 126.66 and 126.59 (1″), 124.71 (d, *J*_4‴-P_ = 1.5 Hz, 4‴), 124.65 (d, *J*_4‴-P_ = 1.5 Hz, 4‴), 120.17 (d, *J*_2‴-P_ = 4.6 Hz, 2‴), 120.02 (d, *J*_2‴-P_ = 5.4 Hz, 2‴), 110.28 and 110.26 (6″), 108.23 and 108.12 (3″), 71.06 (d, *J*_1‴-P_ = 5.4 Hz, 1‴), 70.86 (d, *J*_1‴-P_ = 5.3 Hz, 1‴), 64.07 and 64.01 (4), 56.68 and 56.58 (5″-O**CH_3_**), 56.44 and 56.40 (4″-O**CH_3_**), 45.51 (d, *J*_1′-P_ = 2.3 Hz, 1′), 45.50 (d, *J*_1′-P_ = 2.2 Hz, 1′), 25.79 (d, *J*_2′-P_ = 4.6 Hz, 2′), 25.68 (d, *J*_2′-P_ = 4.7 Hz, 2′), 24.24–24.20 (m, 3′), 19.48 (d, *J*_2-P_ = 5.4 Hz, 2), 19.26 (d, *J*_2-P_ = 5.4 Hz, 2). **^31^P-NMR** (162 MHz, CDCl_3_, 25 °C) δ 3.96 and 3.47 ppm. **HR-MS** (ESI) calculated for C_23_H_30_O_9_N_2_P 509.16834, found [M + H]^+^ 509.16852.

***4,5-dimethoxy-2-nitrobenzyl (2S)-2-((phenoxy(phenylamino)phosphoryl)oxy)propanoate*(16)** was prepared from **24** (142.6 mg, 0.5 mmol, 1.0 eq.) and **21** (74.7 μL, 0.5 mmol, 1.0 eq.) according to the general procedure and purified by Flash silica gel chromatography using a gradient (hexane/EtOAc 100:0→0:100, *v*/*v*), followed by two reverse-phase chromatographies using a gradient (H_2_O/MeCN 100:0→60:40, *v*/*v*), which afforded **16** as a dense yellow oil (53.5 mg, 21%). **^1^H-NMR** (400 MHz, CDCl_3_, 25 °C, 2 diastereoisomers in ca. 2:1 ratio) δ 7.73 and 7.71 (s, 2H, 3″), 7.32–6.96 (m, 20H, 2′, 3′, 4′, 2‴, 3‴, 4‴), 5.91 (d, 1H, *J*_NH-P_ = 9.8 Hz, NH), 5.86 (d, 1H, *J*_NH-P_ = 9.1 Hz, NH), 5.67 (dd, 1H, *J*_Gem_ = 14.8 Hz, *J*_4a-6″_ = 0.6 Hz, 4a), 5.58 (dd, 1H, *J*_Gem_ = 14.8 Hz, *J*_4b-6″_ = 0.6 Hz, 4b), 5.55 (dd, *J*_Gem_ = 14.5 Hz, *J*_4a-6″_ = 0.6 Hz, 4a), 5.51 (dd, *J*_Gem_ = 14.5 Hz, *J*_4b-6″_ = 0.6 Hz, 4b), 5.26–5.14 (m, 2H, 1), 3.97–3.96 (m, 6H, 4″-O**CH_3_**), 3.90 and 3.87 (s, 6H, 5″-O**CH_3_**), 1.67 (dd, 3H, *J*_2-1_ = 6.9 Hz, *J*_2-P_ = 0.5 Hz, 2), 1.60 (dd, 3H, *J*_2-1_ = 6.9 Hz, *J*_2-P_ = 0.3 Hz, 2). **^13^C-NMR** (101 MHz, CDCl_3_, 25 °C) δ 170.03 (d, *J*_3-P_ = 5.0 Hz, 3), 169.75 (d, *J*_3-P_ = 4.7 Hz, 3), 153.73 and 153.65 (5″), 150.29 (d, *J*_1‴-P_ = 6.8 Hz, 1‴), 150.12 (d, *J*_1‴-P_ = 6.5 Hz, 1‴), 148.42 and 148.34 (4″), 139.84 and 139.75 (2″), 138.63 and 138.58 (1′), 129.72 and 129.66 (3‴), 129.40 and 129.29 (3′), 126.37 and 126.06 (1″), 125.40–125.31 (4‴), 122.57 and 122.52 (4′), 120.43 (d, *J*_2‴-P_ = 4.6 Hz, 2‴), 120.28 (d, *J*_2‴-P_ = 4.7 Hz, 2‴), 118.24 (d, *J*_2′-P_ = 7.3 Hz, 2′), 118.07 (d, *J*_2′-P_ = 7.5 Hz, 2′), 110.39 and 110.33 (6″), 108.23 and 108.18 (3″), 72.00 (d, *J*_1-P_ = 4.9 Hz, 1), 71.75 (d, *J*_1-P_ = 4.8 Hz, 1), 64.37 and 64.30 (4), 56.54 and 56.51 (5″-O**CH_3_**), 56.45–56.39 (4″-O**CH_3_**), 19.23 (d, *J*_2-P_ = 5.8 Hz, 2). **^31^P-NMR** (162 MHz, CDCl_3_, 25 °C) δ −2.50 and −2.99 ppm. **HR-MS** (ESI) calculated for C_24_H_26_O_9_N_2_P 517.13704, found [M + H]^+^ 517.13695.

***Methyl-1-cyclopropyl-7-(4-(((1-((4,5-dimethoxy-2-nitrobenzyl)oxy)-1-oxopropan-2-yl)oxy)(ethoxy)phosphoryl)piperazin-1-yl)-6-fluoro-4-oxo-1,4-dihydroquinoline-3-carboxylate* (17).** To a solution of **24** (71.3 mg, 0.25 mmol, 1 eq.) in 1.5 mL of dry toluene, **20** (31.6 μL, 0.25 mmol, 1.0 eq.), and TEA (45.4 μL, 0.325 mmol, 1.3 eq.) were added, and the reaction mixture was stirred at 25 °C for 16 h. The formation of the intermediate **25** was confirmed by ^31^P NMR (δ_P_ 4.6 and 4.1 ppm) before adding amine 27 (86.3 mg, 0.25 mmol, 1.0 eq.) and TEA (34.9 μL, 0.25 mmol, 1.0 eq.). The reaction mixture was stirred at room temperature until completion (1 h). After solvent evaporation, the crude residue was purified by Flash silica gel chromatography using a gradient (hexane/EtOAc/MeOH 50:50:0→0:100:0→0:50:50 *v/v/v*), which afforded **17** as a white solid (78.1 mg, 43%). **^1^H-NMR** (400 MHz, CDCl_3_, 25 °C, 2 diastereoisomers in ca. 1:1 ratio) δ 8.53 and 8.53 (s, 2H, 9′), 8.00 (d, 1H, *J*_7′-F_ = 13.2 Hz, 7′), 8.00 (d, 1H, *J*_7′-F_ = 13.1 Hz, 7′), 7.72 and 7.67 (s, 2H, 3″), 7.27 (d, 1H, *J*_4′-F_ = 7.2 Hz, 4′), 7.26 (d, 1H, *J*_4′-F_ = 7.1 Hz, 4′), 7.05 and 7.02 (s, 2H, 6″), 5.66–5.53 (m, 4H, 4), 5.06–4.92 (m, 2H, 1), 4.19–4.06 (m, 4H, O**CH_2_**CH_3_), 4.01 and 4.00 (s, 6H, 5″-O**CH_3_**), 3.97 and 3.93 (s, 6H, 4″-O**CH_3_**), 3.91–3.90 (m, 6H, 16′), 3.50–3.30 (m, 10H, 1′, 12′), 3.26–3.08 (m, 8H, 2′), 1.65–1.60 (m, 6H, 2), 1.43–1.28 (m, 10H, OCH_2_**CH_3_**, 13′ or 14′), 1.18–1.11 (m, 4H, 13′ or 14′). **^13^C-NMR** (101 MHz, CDCl_3_, 25 °C) δ 173.04 (d, *J*_11′-F_ = 2.1 Hz, 11′), 170.00 (d, *J*_11′-F_ = 2.2 Hz, 11′), 170.50 (d, *J*_3-P_ = 4.6 Hz, 3), 170.43 (d, *J*_3-P_ = 5.4 Hz, 3), 166.40 and 166.35 (15′), 153.38 (d, *J*_8′-F_ = 248.6 Hz, 8′), 153.63 (5″), 148.58 and 148.55 (4″), 148.42 (9′), 144.55 (d, *J*_3′-F_ = 10.5 Hz, 3′), 144.49 (d, *J*_3′-F_ = 10.6 Hz, 3′), 140.06 (2″), 138.01 (d, *J*_5′-F_ = 1.5 Hz, 5′), 137.99 (d, *J*_5′-F_ = 1.5 Hz, 5′), 126.09 and 125.97 (1″), 123.36 (d, *J*_6′-F_ = 7.3 Hz, 6′), 123.28 (d, *J*_6′-F_ = 6.9 Hz, 6′), 113.40 (d, *J*_7′-F_ = 23.5 Hz, 7′), 113.30 (d, *J*_7′-F_ = 22.9 Hz, 7′), 110.94 and 110.85 (6″), 110.12 and 110.07 (10′), 108.30 and 108.26 (3″), 105.13 (d, *J*_4′-F_ = 9.5 Hz, 4′), 105.12 (d, *J*_4′-F_ = 9.1 Hz, 4′), 70.69 (d, *J*_1-F_ = 4.8 Hz, 1), 70.65 (d, *J*_1-F_ = 5.6 Hz, 1), 64.21 and 64.18 (4), 63.21 (d, *J*_CH2-P_ = 6.0 Hz, O**CH_2_**CH_3_), 63.00 (d, *J*_CH2-P_ = 6.0 Hz, O**CH_2_**CH_3_), 56.68 and 56.63 (5″-O**CH_3_**), 56.45 and 56.44 (4″-O**CH_3_**), 52.07 and 52.06 (16′), 50.26 and 50.09 (m, 2′), 44.40 and 44.38 (1′), 34.56 and 34.55 (12′), 19.50 (d, *J*_2-P_ = 5.2 Hz, 2), 16.18 (d, *J*_CH3-P_ = 7.0 Hz, OCH_2_**CH_3_**), 16.14 (d, *J*_CH3-P_ = 7.0 Hz, OCH_2_**CH_3_**), 8.15 (m, 13′, 14′). **^31^P-NMR** (162 MHz, CDCl_3_, 25 °C) δ 7.87 and 7.17 ppm. **HR-MS** (ESI) calculated for C_32_H_39_O_12_N_4_FP 721.22806, found [M + H]^+^ 721.22796. 

***Methyl-1-cyclopropyl-7-(4-(ethoxy((1-ethoxy-1-oxopropan-2-yl)oxy)phosphoryl)piperazin-1-yl)-6-fluoro-4-oxo-1,4-dihydroquinoline-3-carboxylate* (18).** To a solution of (-)-Ethyl L-Lactate (28.7 μL, 0.25 mmol, 1 eq.) in 1.5 mL of dry toluene, **20** (31.6 μL, 0.25 mmol, 1.0 eq.) and TEA (45.4 μL, 0.325 mmol, 1.3 eq.) were added, and the reaction mixture was stirred at 25 °C for 16 h. The formation of the intermediate was confirmed by ^31^P-NMR (δ_P_ 4.7 and 3.7 ppm) before adding amine **27** (86.3 mg, 0.25 mmol, 1.0 eq.) and TEA (34.9 μL, 0.25 mmol, 1.0 eq.). The reaction mixture was stirred at 25 °C until completion (1 h). After evaporating the solvent, the crude residue was purified by Flash silica gel chromatography using a gradient (hexane/EtOAc 50:50→0:100, *v*/*v*), followed by reverse-phase chromatography using a gradient (H_2_O/MeCN 95:5→50:50, *v*/*v*), which afforded 18 as white solid (61.1 mg, 44%). **^1^H-NMR** (400 MHz, CDCl_3_, 25 °C, 2 diastereoisomers in ca. 1:1 ratio) δ 8.60–8.57 (m, 2H, 9′), 8.08 (d, 1H, *J*_7′-F_ = 13.2 Hz, 7′), 8.07 (d, 1H, *J*_7′-F_ = 13.2 Hz, 7′), 7.31–7.27 (m, 2H, 4′), 4.95–4.83 (m, 2H, 1), 4.30–4.08 (m, 8H, 4, O**CH_2_**CH_3_), 3.95–3.93 (m, 6H, 16′), 3.53–3.36 (m, 10H, 1′, 12′), 3.28–3.20 (m, 8H, 2′), 1.60 (d, 3H, *J*_2-P_ = 7.0 Hz, 2), 1.57 (d, 3H, *J*_2-P_ = 6.9 Hz, 2), 1.40–1.29 (m, 16H, 13′ or 14′, 5, OCH_2_**CH_3_**), 1.19–1.13 (m, 4H, 13′ or 14′). **^13^C-NMR** (101 MHz, CDCl_3_, 25 °C) δ 173.17–173.02 (m, 11′), 171.00 (d, *J*_3-P_ = 4.5 Hz, 3), 170.91 (d, *J*_3-P_ = 5.4 Hz, 3), 166.57–166.49 (m, 15′), 153.44 (d, *J*_8′-F_ = 249.1 Hz, 8′), 148.45 (9′), 144.76–144.55 (m, 3′), 138.02 (5′), 123.63–123.24 (m, 6′), 113.50 (d, *J*_7′-F_ = 23.1 Hz, 7′), 113.47 (d, *J*_7′-F_ = 22.8 Hz, 7′), 110.32–110.05 (m, 10′), 105.05–104.96 (m, 4′), 70.78 (d, *J*_1-P_ = 5.4 Hz, 1), 63.06 (d, *J*_CH2-P_ = 6.1 Hz, OCH_2_CH_3_), 62.94 (d, *J*_CH2-P_ = 6.2 Hz, O**CH_2_**CH_3_), 61.46 and 61.41 (4), 52.14 (16′), 50.41–50.14 (m, 2′), 44.43–44.34 (m, 1′), 34.54 (12′), 19.54–19.36 (2), 16.20 (d, *J*_CH3-P_ = 6.9 Hz, OCH_2_**CH_3_**), 16.17 (d, *J*_CH3-P_ = 6.6 Hz, OCH_2_**CH_3_**), 14.21 and 14.15 (5), 8.18 (m, 13′, 14′). **^31^P-NMR** (162 MHz, CDCl_3_, 25 °C) δ 7.78 and 6.98 ppm. **HR-MS** (ESI) calculated for C_25_H_34_O_8_N_3_FP 554.20621, found [M + H]^+^ 554.20614.

***Methyl-1-cyclopropyl-6-fluoro-4-oxo-7-(piperazin-1-yl)-1,4-dihydroquinoline-3-carboxylate* (27).** To a suspension of Ciprofloxacin (1.0 g, 3.0 mmol, 1 eq.) in 30 mL of dry methanol, cooled to 0 °C, SOCl_2_ was added dropwise, and the resulting solution was refluxed for 16 h [33]. After evaporating the solvent, the crude residue was dissolved in CH_2_Cl_2_ and washed with a saturated solution of K_2_CO_3_, and the organic layer was dried over Na_2_SO_4_. After filtration and solvent removal, the crude residue was purified by Flash silica gel chromatography using a gradient (CH_2_Cl_2_/MeOH 100:0→70:30, *v*/*v*), to obtain methyl ester **27** as a white solid (0.78 g, 76%). 

*Characterization of intermediates (**I**), products (**P**) obtained after irradiation with UV light, and undesired products (**X**)*

**1-I**. **^13^C-NMR** (126 MHz, 50% CACO/DMSO, 25 °C) δ 140.73 (1′), 130.43 (2′ or 3′), 130.07 (3′ or 2′), 127.93 (4′), 68.73 (d, *J*_2-P_ = 5.8 Hz, 2), 64.25 (d, *J*_1-P_ = 5.4 Hz, 1), 61.94 (d, *J*_CH2-P_ = 7.9 Hz, O**CH_2_**CH_3_), 43.81 (NH-**CH_2_**), 38.71 (d, *J*_CH2-P_ = 6.1 Hz, **CH_2_**Ph), 17.25 (d, *J*_CH3-P_ = 6.5 Hz, OCH_2_**CH_3_**). **^31^P-NMR** (202 MHz, 50% CACO/DMSO-*d*_6_, 25 °C) δ 10.83 ppm. **HR-MS** (ESI-) calculated for C_12_H_19_O_4_NP 272.10572, found [M−H]^−^ 272.10551. 

**2-I. ^13^C-NMR** (126 MHz, 50% CACO/DMSO-*d*_6_, 25 °C) δ 68.79 (d, *J*_2-P_ = 5.5 Hz, 2), 64.34 (d, *J*_1-P_ = 5.6 Hz, 1), 61.92 (d, *J*_CH2-P_ = 7.6 Hz, O**CH_2_**CH_3_), 46.33 (d, *J*_1′-P_ = 2.1 Hz, 1′), 26.97 (d, *J*_2′-P_ = 4.7 Hz, 2′), 25.15 (3′), 17.25 (d, *J*_CH3-P_ = 6.6 Hz, OCH_2_**CH_3_**). **^31^P-NMR** (202 MHz, 50% CACO/DMSO-*d*_6_, 25 °C) δ 9.81 ppm. **HR-MS** (ESI−) calculated for C_9_H_19_O_4_NP 236.10572, found [M−H]^−^ 236.10585.

**3-I. ^13^C-NMR** (126 MHz, 50% CACO/DMSO-*d*_6_, 25 °C) δ 141.11 (1′), 131.01 (3′), 123.48 (4′), 119.19 (d, *J*_2′-P_ = 7.6 Hz, 2′), 69.46 (d, *J*_2-P_ = 5.9 Hz, 2), 65.11 (d, *J*_1-P_ = 5.6 Hz, 1), 61.82 (d, *J*_CH2-P_ = 7.9 Hz, O**CH_2_**CH_3_), 17.19 (d, *J*_CH3-P_ = 6.9 Hz, OCH_2_**CH_3_**). **^31^P-NMR** (202 MHz, 50% CACO/DMSO-*d*_6_, 25 °C) δ 3.62 ppm. **HR-MS** (ESI−) calculated for C_10_H_15_O_4_NP 244.07442, found [M−H]^−^ 244.07419.

**4-I. ^13^C-NMR** (126 MHz, 50% HEPES/DMSO-*d*_6_, 25 °C) δ 151.73 (d, *J*_1‴-P_ = 6.8 Hz, 1‴), 140.51 (1′), 131.54 (3‴), 130.40 (2′), 130.08 (3′), 127.98 (4′), 126.77 (4‴), 121.67 (d, *J*_2‴-P_ = 4.6 Hz, 2‴), 69.41 (d, *J*_2-P_ = 5.9 Hz, 2), 61.85 (d, *J*_1-P_ = 7.8 Hz, 1), 44.04 (NH**CH_2_**), 38.58 (d, *J*_CH2-P_ = 6.1 Hz, **CH_2_**Ph). **^31^P-NMR** (202 MHz, 50% HEPES/DMSO-*d*_6_, 25 °C) δ 6.32 ppm. **HR-MS** (ESI) calculated for C_16_H_20_O_4_NNaP 344.10222, found [M + Na]^+^ 344.10212. 

**4-cyc-I. ^13^C-NMR** (126 MHz, 50% CACO/DMSO-*d*_6_, 25 °C) δ 140.45 (1′), 130.41 (2′), 130.08 (3′), 127.98 (4′), 67.74−67.69 (m, 2, 1), 43.71 (NH**CH_2_**), 38.67 (d, *J*_CH2-P_ = 5.2 Hz, **CH_2_**Ph). **^31^P-NMR** (202 MHz, 50% CACO/DMSO-*d*_6_, 25 °C) δ 28.06 ppm. **HR-MS** (ESI−) calculated for C_10_H_13_O_3_NP 226.06385, found [M−H]^−^ 226.06358.

**4-P2. ^13^C-NMR** (126 MHz, 50% CACO/DMSO-*d*_6_, 25 °C) δ 141.56 (1′), 130.38 (2′), 130.03 (3′), 127.69 (4′), 66.76 (d, *J*_2-P_ = 5.3 Hz, 2), 62.90 (d, *J*_1-P_ = 7.3 Hz, 1), 44.60 (NH**CH_2_**), 39.2* (**CH_2_**Ph). * The ^13^C chemical shift was extracted from HMBC. **^31^P-NMR** (202 MHz, 50% CACO/DMSO-*d*_6_, 25 °C) δ 8.05 ppm. **HR-MS** (ESI−) calculated for C_10_H_15_O_4_NP 244.07442, found [M−H]^−^ 244.07457.

**5-I. ^13^C-NMR** (126 MHz, 50% CACO/DMSO-*d*_6_, 25 °C) δ 151.72 (d, *J*_1‴-P_ = 6.8 Hz, 1‴), 131.58 (3‴), 126.83 (4‴), 121.64 (d, *J*_2‴-P_ = 5.1 Hz, 2‴), 64.48 (d, *J*_2-P_ = 6.0 Hz, 2), 61.84 (d, *J*_1-P_ = 7.9 Hz, 1), 46.53 (d, *J*_1′-P_ = 1.9 Hz, 1′), 26.77 (d, *J*_2′-P_ = 4.5 Hz, 2′), 25.01 (3′). **^31^P-NMR** (202 MHz, 50% CACO/DMSO-*d*_6_, 25 °C) δ 5.10 ppm. **HR-MS** (ESI) calculated for C_13_H_21_O_4_NP 286.12027, found [M + H]^+^ 286.12039. 

**5-cyc-I. ^13^C-NMR** (126 MHz, 50% CACO/DMSO-*d*_6_, 25 °C) δ 67.87−67.83 (m, 2, 1), 46.54 (d, *J*_1′-P_ = 3.1 Hz, 1′), 27.03 (d, *J*_2′-P_ = 3.6 Hz, 2′), 25.07 (3′). **^31^P-NMR** (202 MHz, 50% CACO/DMSO-*d*_6_, 25 °C) δ 26.48 ppm. **HR-MS** (ESI) calculated for C_7_H_15_O_3_NP 192.07841, found [M + H]^+^ 192.07829.

**6-I. ^13^C-NMR** (126 MHz, 50% CACO/DMSO-*d*_6_, 25 °C) δ 151.39 (d, *J*_1‴-P_ = 6.7 Hz, 1‴), 140.63 (1′), 131.66 (3‴), 131.10 (3′), 127.18 (4‴), 123.92 (4′), 121.55 (d, *J*_2‴-P_ = 4.7 Hz, 2‴), 119.50 (d, *J*_2′-P_ = 7.7 Hz, 2′), 70.19 (d, *J*_2-P_ = 5.8 Hz, 2), 61.76 (d, *J*_1-P_ = 7.6 Hz, 1). **^31^P-NMR** (202 MHz, 50% CACO/DMSO-*d*_6_, 25 °C) δ −0.94 ppm.

**6-cyc-I. ^13^C-NMR** (126 MHz, 50% CACO/DMSO-*d*_6_, 25 °C) δ 140.23 (1′), 131.15 (3′), 124.73 (4′), 120.98 (d, *J*_2′-P_ = 7.3 Hz, 2′), 68.02−67.95 (m, 2, 1). **^31^P-NMR** (202 MHz, 50% CACO/DMSO-*d*_6_, 25 °C) δ 21.66 ppm.

**9-I.HR-MS** (ESI−) calculated for C_11_H_15_O_5_NP 272.06933, found [M−H]^−^ 272.06926. 

**13-I. ^13^C-NMR** (126 MHz, 50% CACO/DMSO-*d*_6_, 25 °C) δ 177.54 (d, *J*_3-P_ = 4.5 Hz, 3), 160.21–160.10 (m, 2′), 160.05–159.93 (m, 1′), 116.69 and 116.67 (3′), 76.31 (d, *J*_1-P_ = 6.3 Hz, 1), 76.20 (d, *J*_1-P_ = 6.1 Hz, 1), 65.89 (d, *J*_CH2-P_ = 6.1 Hz, O**CH_2_**CH_3_), 65.87 (d, *J*_CH2-P_ = 6.2 Hz, O**CH_2_**CH_3_), 21.29 (d, *J*_2-P_ = 5.7 Hz, 2), 21.08 (d, *J*_2-P_ = 5.5 Hz, 2), 17.14 (d, *J*_CH3-P_ = 7.3 Hz, OCH_2_**CH_3_**), 17.10 (d, *J*_CH3-P_ = 6.7 Hz, OCH_2_**CH_3_**). **^31^P-NMR** (202 MHz, 50% CACO/DMSO-*d*_6_, 25 °C) δ −0.13 and −0.75 ppm. **HR-MS** (ESI−) calculated for C_9_H_13_O_5_N_3_P 274.05983, found [M − H]^−^ 274.05995.

**16-I. ^13^C-NMR** (126 MHz, 50% CACO/DMSO-*d*_6_, 25 °C) δ 153.93 (d, *J*_3-P_ = 6.8 Hz, 3), 151.68–151.46 (m, 1‴), 140.89 (1′). 140.79 (1′), 131.55 (3‴ or 3′), 131.53 (3‴ or 3′), 131.03 (3′ or 3‴), 130.98 (3′ or 3‴), 127.03 (4‴), 123.66 (4′), 123.63 (4′), 121.69–121.60 (m 2‴), 119.36 (d, *J*_2′-P_ = 8.0 Hz, 2′), 76.35 (d, *J*_1-P_ = 5.9 Hz, 1), 21.31 (d, *J*_2-P_ = 4.8 Hz, 2). **^31^P-NMR** (202 MHz, 50% CACO/DMSO-*d*_6_, 25 °C) δ −2.21 and −2.63 ppm. **HR-MS** (ESI−) calculated for C_15_H_15_O_5_NP 320.06933, found [M − H]^−^ 320.06956.

**7-P** (= **8-P**, **9-P**, **10-P**, **11-P**, **12-P**, **13-P**, **17-P**, **10-P**, **11-P**, **12-P**, **13-P**, **17-P**, **18-P**). **^13^C-NMR** (126 MHz, 50% CACO/DMSO-*d*_6_, 25 °C) δ 179.92 (d, *J*_3-P_ = 6.5 Hz, 3), 73.60 (d, *J*_1-P_ = 5.6 Hz, 1), 62.56 (d, *J*_CH2-P_ = 5.5 Hz, O**CH_2_**CH_3_), 21.60 (d, *J*_2-P_ = 3.1 Hz, 2), 17.52 (d, *J*_CH3-P_ = 7.4 Hz, OCH_2_**CH_3_**). **^31^P-NMR** (202 MHz, 50% CACO/DMSO-*d*_6_, 25 °C) *δ* −1.09 ppm. **HR-MS** (ESI−) calculated for C_5_H_10_O_6_P 197.02205, found [M − H]^−^ 197.02187.

**14-P1. ^13^C-NMR** (126 MHz, 50% CACO/DMSO-*d*_6_, 25 °C) δ 179.52 (d, *J*_3-P_ = 7.1 Hz, 3), 153.92 (d, *J*_1‴-P_ = 6.9 Hz, 1‴), 130.95 (3‴), 124.87 (4‴), 121.76 (d, *J*_2‴-P_ = 4.6 Hz, 2‴), 74.33 (d, *J*_1-P_ = 6.1 Hz, 1), 21.51 (d, *J*_2-P_ = 2.9 Hz, 2). **^31^P-NMR** (202 MHz, 50% CACO/DMSO-*d*_6_, 25 °C) δ −6.24 ppm. **HR-MS** (ESI−) calculated for C_9_H_10_O_6_NP 245.02205, found [M−H]^−^ 245.02184.

**7-X. ^13^C-NMR** (126 MHz, 50% CACO/DMSO-*d*_6_, 25 °C) δ 175.44 (d, *J*_3-P_ = 6.5 Hz, 3), 140.41 (1′), 130.32 (2′), 130.07 (3′), 127.96 (4′), 72.75 (d, *J*_1-P_ = 5.5 Hz, 1), 62.90–62.78 (m, O**CH_2_**CH_3_), 41.61 (NH-**CH_2_**), 36.02 (**CH_2_**Ph), 21.10–21.01 (m, 2), 17.61–17.45 (m, OCH_2_**CH_3_**). **^31^P-NMR** (202 MHz, 50% CACO/DMSO-*d*_6_, 25 °C) δ −1.76 ppm. **HR-MS** (ESI−) calculated for C_13_H_19_O_5_NP 300.10063, found [M − H]^−^ 300.10069.

**9-X. ^13^C-NMR** (126 MHz, 50% CACO/DMSO-*d*_6_, 25 °C) δ 173.93 (d, *J*_3-P_ = 6.2 Hz, 3), 138.54 (1′), 130.60 (3′), 126.60 (4′), 122.38 (2′), 73.05 (d, *J*_1-P_ = 5.5 Hz, 1), 62.94 (d, *J*_CH2-P_ = 5.8 Hz, O**CH_2_**CH_3_), 20.93 (d, *J*_2-P_ = 3.3 Hz, 2), 17.52 (d, *J*_CH3-P_ = 7.2 Hz, OCH_2_**CH_3_**). **^31^P-NMR** (202 MHz, 50% CACO/DMSO-*d*_6_, 25 °C) δ −1.57 ppm. **HR-MS** (ESI−) calculated for C_11_H_15_O_5_NP 272.06933, found [M−H]^−^ 272.06943.

## 5. Conclusions

In summary, we designed and synthesized phosphate-based SI linkers for amine- containing drug delivery. We found that the lactate spacer can release amines effectively within 15 min; moreover, it can release two cargos sequentially—the first amine cargo within minutes and the second phenolic cargo overnight. Surprisingly, this is exactly the opposite release order that we found when using an ethylene glycol SI spacer, whereby phenol is released preferentially [22]. Interestingly, the linkers bearing primary amines lack stability at physiological pH (pH = 7.4) due to an intramolecular rearrangement caused by the nucleophilic attack of NH nitrogen from LG on the carbonyl group of lactate. This alternative decomposition, which yields the undesired product **X**, can be suppressed by changing the buffer (e.g., HEPES instead of Cacodylate buffer, pH = 7.4), by decreasing the buffer pH to mildly acidic (pH = 5), or by *N*-methylation of phosphoramidate nitrogen. In turn, derivatives bearing secondary amines are stable in a range of pH 5–7.4. As such, our prodrug approach is the most suitable for the delivery of secondary amines. Further applicability was demonstrated by phosphorylation of the antibiotic Ciprofloxacin, whose phototriggerable and enzyme-triggerable prodrugs released Ciprofloxacin successfully. Overall, our results establish an experimental paradigm for the smart design of new self-immolative systems for the targeted delivery of various amine-containing drugs and their enhanced cellular uptake and activity, thus broadening the applications of prodrug technology. Moreover, phospholane amidates could lead to the design of new synthetic approaches in phosphorus chemistry. 

## Data Availability

Not applicable.

## Supplementary Materials

The Supplementary Materials are available online.

## Sample Availability

Samples of the compounds are not available from the authors.
