# Supplementary material for: Phosphate-Based Self-Immolative Linkers for the Delivery of Amine-Containing Drugs
Source: Molecules. 2021 Aug 25;26(17):5160. doi: 10.3390/molecules26175160 (PMC8434617; doi:10.3390/molecules26175160)
Supplement: Supplementary file 1 [file molecules-26-05160-s001.zip › molecules-1331912-supplementary.pdf]

Phosphate-based Self-Immolative Linkers for the Delivery of Amine-Containing Drugs

Mateja Đud,[a] Markéta Tichotová, [a,b] Eliška Procházková [b,]* and Ondřej Baszczyňski [a,b]*

[a] *Faculty of Science, Charles University, Prague 128 43, Czech Republic*
[b] *Institute of Organic Chemistry and Biochemistry, The Czech Academy of Sciences, Prague 166 10, Czech Republic*

Supporting Information

Table of Contents

1 NMR spectroscopy S3

1.1 *Irradiation NMR experiments* S3

1.2 Stability tests S5

2 Synthesis S10

3 Compound characterization: NMR spectra S13

4 Compound characterization: MS spectra S40

5 Antibiotic activity of ciprofloxacin, and its derivatives **18** and **27** S58

1 NMR spectroscopy

1.1 *Irradiation NMR experiments*

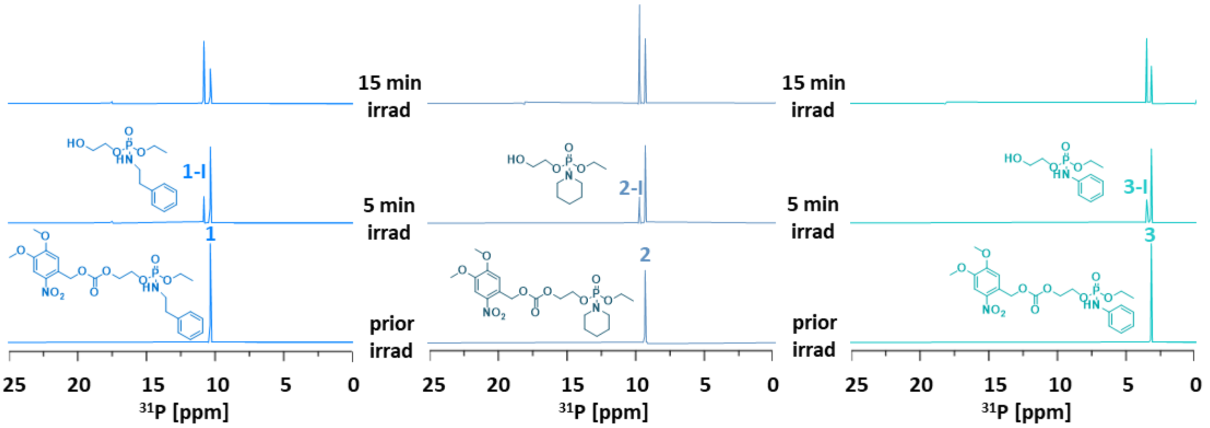

Figure S1. A series of ^{31}P NMR spectra of ethylene glycol-based linkers **1**–**3** (5 mM solutions in 50% CACO (pH = 7.4)/DMSO), measured before and after irradiation by UV light (365 nm) at room temperature (25 °C). After 15 minutes of irradiation only intermediate **I** was formed, and the cargo was not released within a minute-scale.

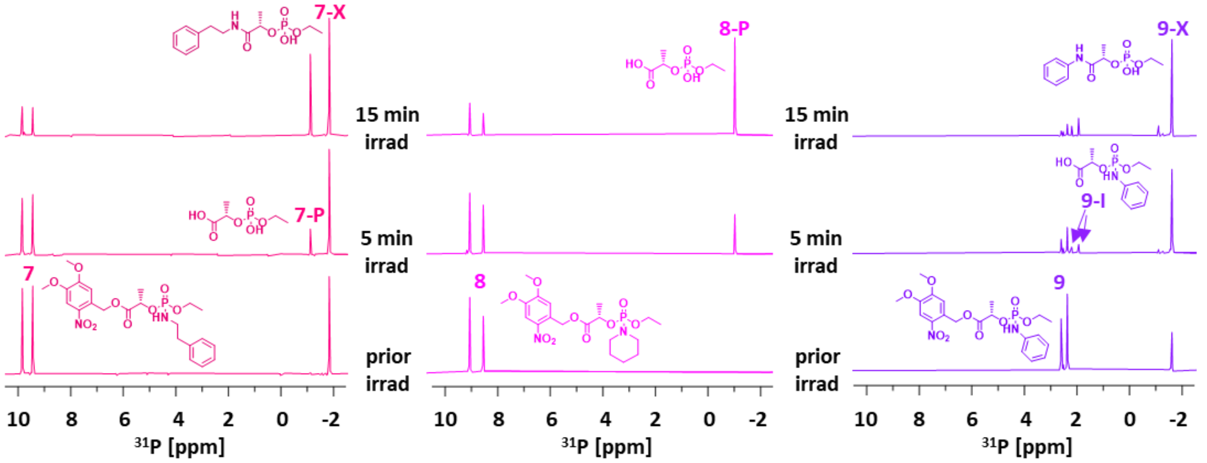

Figure S2. A series of ^{31}P NMR spectra of compounds **7**–**9** (5 mM in 50% CACO (pH = 7.4)/DMSO, 25 °C) measured before and after irradiation by UV light (365 nm). Product of the intramolecular rearrangement of **7** and **9**, carboxamides **7-X** and **9-X**, emerges even prior to irradiation, indicating the limited stability of linkers **7** and **9** in this solvent system.

As we observed decomposition of linkers **7** and **9** in 50% CACO/DMSO solvent system, we searched for a new environment which will be suitable for our further work. For this purpose, we used linker **7** bearing phenethylamine as a primary amine cargo and monitored the SI in several evironments (Figure S3). First, we changed only the pH of CACO buffer in the CACO/DMSO mixture from 7.4 to 5. Surprisingly, decreasing the pH prevented the alternative decomposition as it did not yield **7-X**. However, to keep the physiological pH, we selected other three buffers (HEPES, PBS, TRIS) at pH 7.4. HEPES did not allow formation of **7-X** and was chosen as the best solvent system for further NMR

experiments. We also compared buffered mixtures with unbuffered system (D_2O), which also showed self-immolation and formation only of the SI product **7-P**.

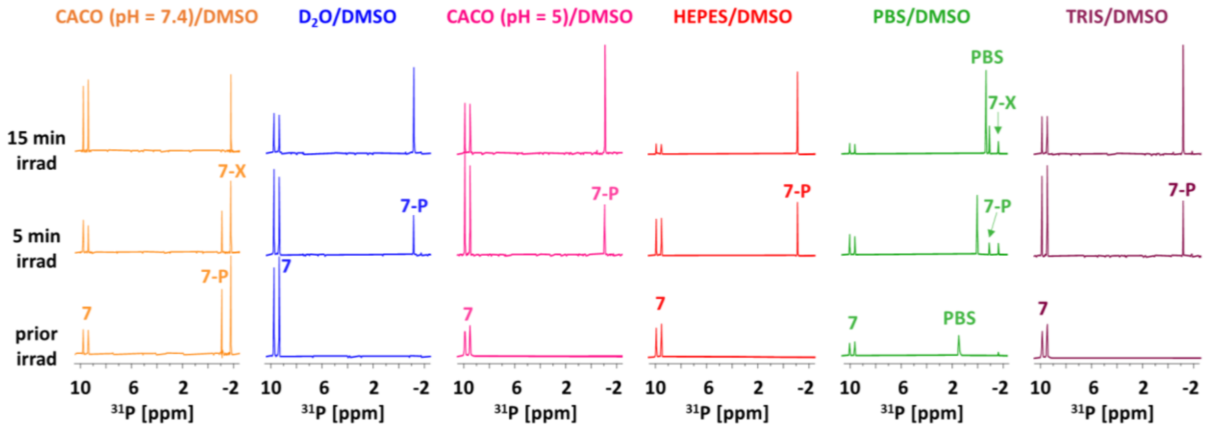

Figure S3. Optimization of the reaction conditions for the unstable linkers bearing primary amines. Series of ^{31}P spectra of compound **7** (5 mM) before and after irradiation by UV light (365 nm) measured in different solvent mixtures at room temperature (25 °C).

According to the results we have got up to now, NH group from the primary amine cargo initiates the intramolecular rearrangement. Therefore, we prepared *N*-methylated derivative of **9** (**10**) and performed the same irradiation experiments in 50% CACO/DMSO solvent system (Figure S4). While in **9** we detected a significant amount of **9-X** even prior to UV irradiation, *N*-methylated linker **10** obediently yielded only the final product **10-P** got from self-immolation, and no **10-X** was detected.

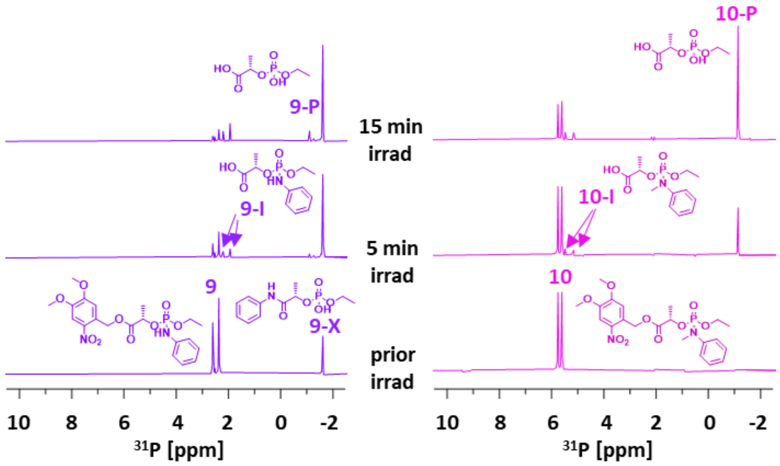

Figure S4. The effect of *N*-methylation on stability of the linker. ^{31}P NMR spectra of **9** and **10** in CACO (pH = 7.4)/DMSO-d_6 before and after UV irradiation (365 nm) at room temperature.

1.2 Stability tests

Limited stability of **7** and **9** and the formation of the side product **X** led us to investigate stability of **7**–**9**. Stability of linkers **7**–**9** was studied in a 1:1 mixture of HEPES (0.1 M)/DMSO-d_6, using ^{31}P NMR spectroscopy at 25 °C, at pH 7.4 and 5. As we have observed earlier (Figure S3), the stability of the compounds depends also on the nature of the buffer. Therefore, in order to stay consistent in terms of buffer, we had to acidify HEPES solution to pH 5, bellow its buffer range (6.8–8.2).

Firstly, we tested the stability of **7**–**9** at pH 7.4. Derivative **8** bearing the secondary amine cargo was stable in HEPES/DMSO mixture and did not provide **8-X** (Figure S5). In contrast, compounds **7** and **9** containing free NH showed slow decomposition to the undesired product **7-X** and **9-X**, respectively. In 7 days, **X** was the major component in both cases. The linker bearing aliphatic amine **7** showed a higher stability (93% of **7** remains intact during the first 24 hours, and 47% decomposes after 7 days) than **9** with aromatic amine (86% of **9** remains intact during first 24 hours, and 57% decomposes after seven days) (Figures S6a, S7a and S8). On the contrary, all three linkers were stable under the acidic conditions (pH = 5) and no **X** was detected (Figures S5b–7b).

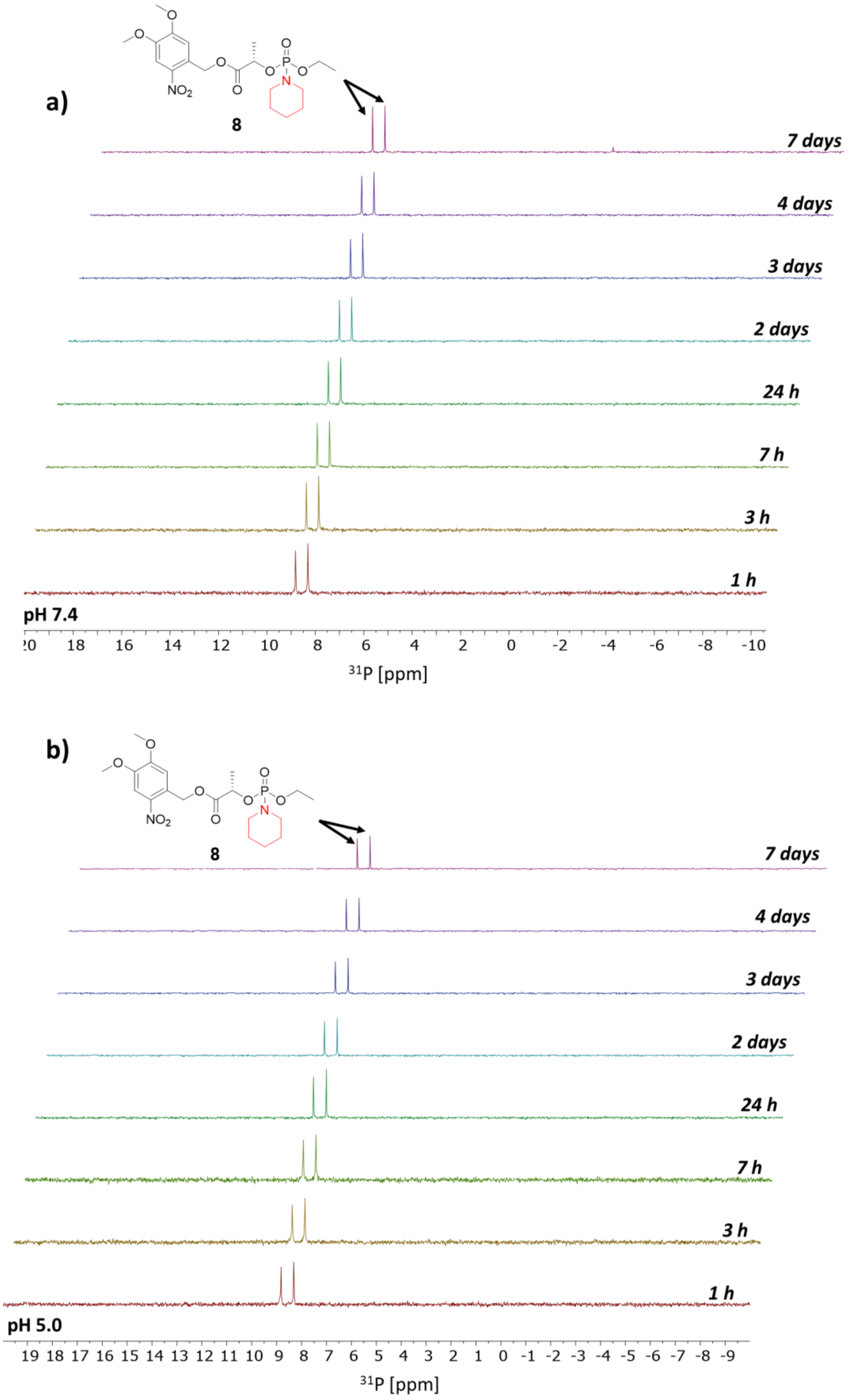

Figure S5. A series of ^{31}P NMR spectra measured in HEPES (0.1 M)/DMSO-d_6 at a) pH 7.4 and b) pH 5 used for monitoring chemical stability of **8**.

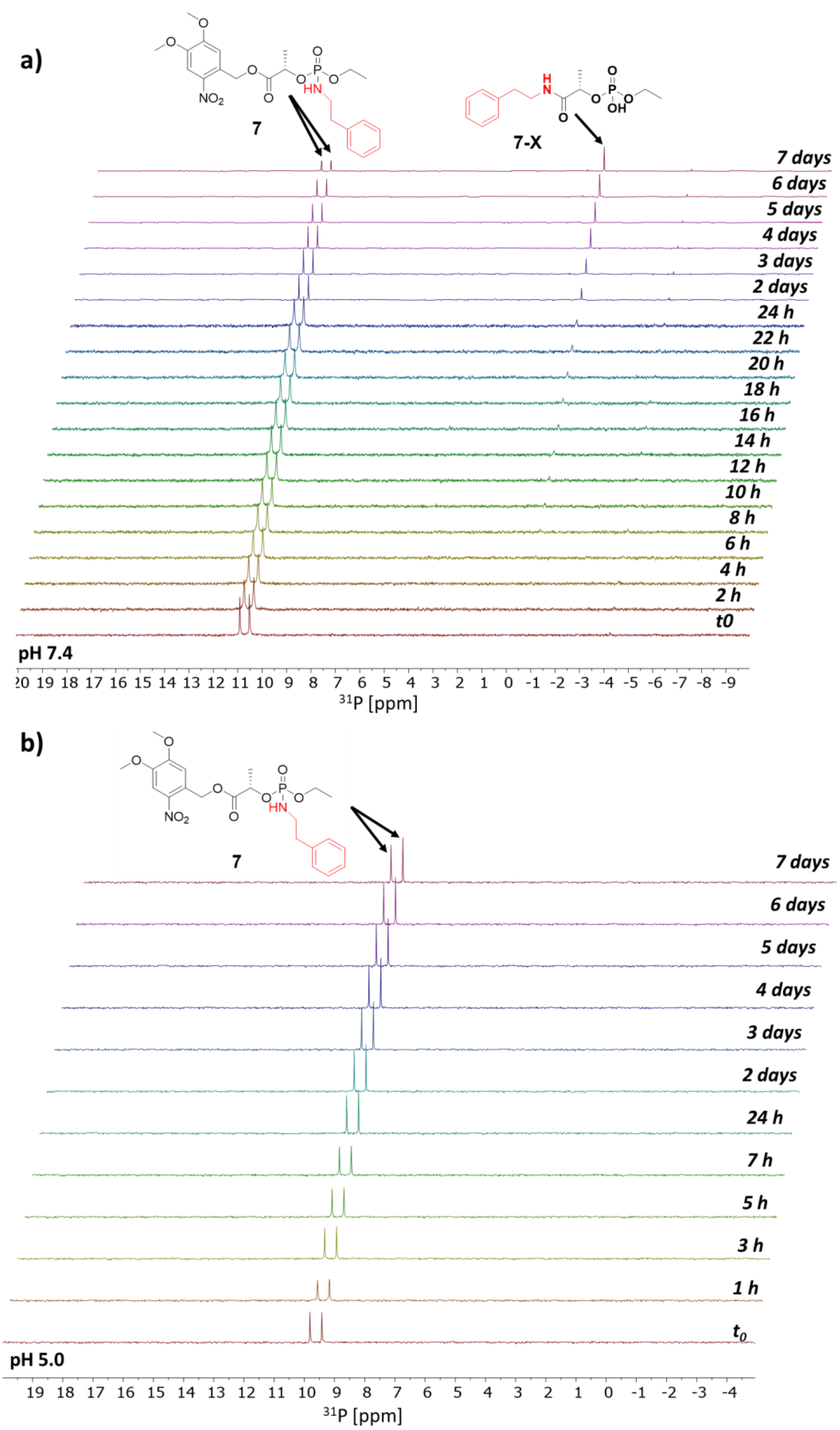

Figure S6. A series of ^{31}P NMR spectra measured in HEPES (0.1 M)/DMSO-d_6 at a) pH 7.4 and b) pH 5 used for monitoring chemical stability of **7**.

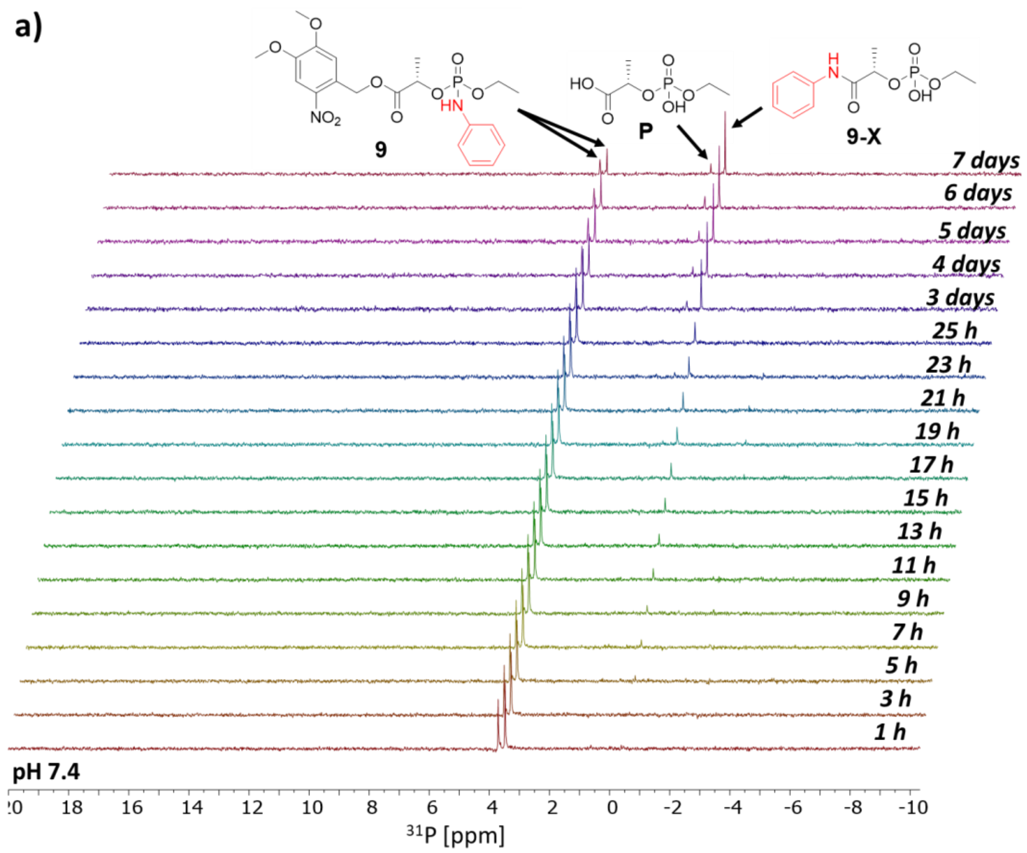

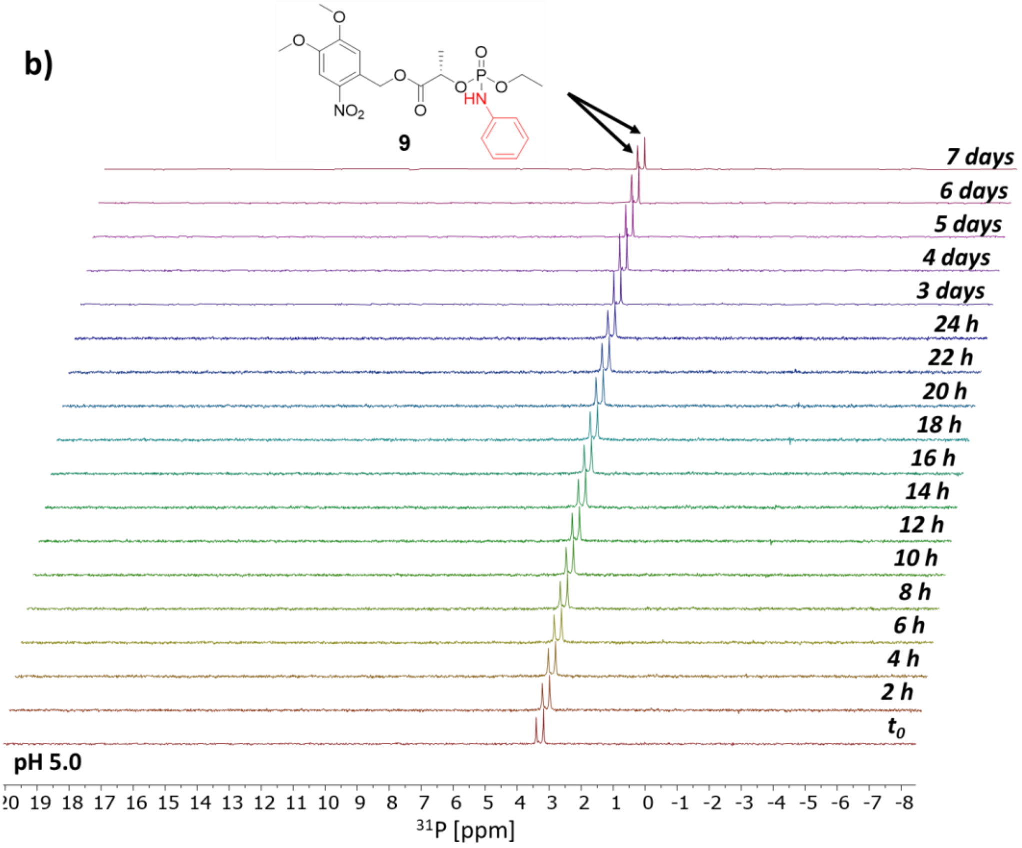

Figure S7. A series of ^{31}P NMR spectra measured in HEPES (0.1 M)/DMSO-d_6 at a) pH 7.4 and b) pH 5 used for monitoring chemical stability of **9**.

a)

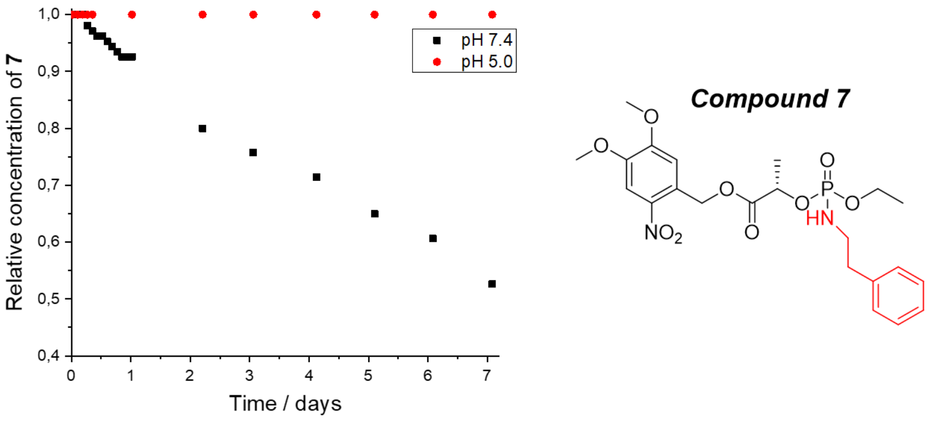

b)

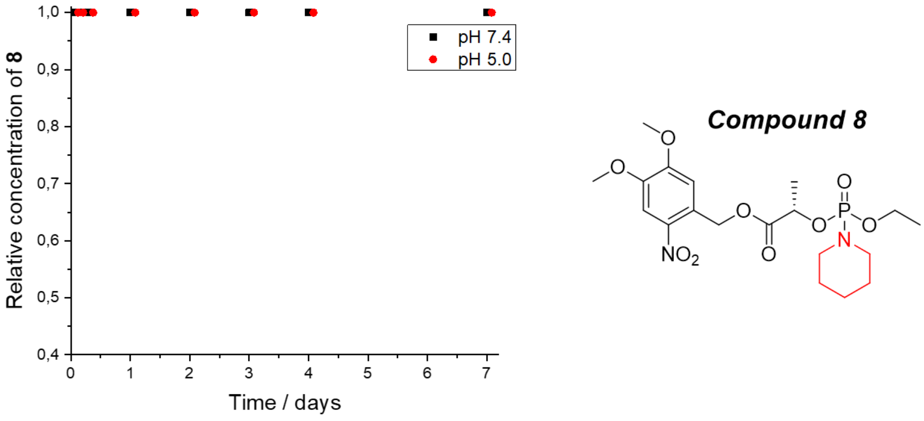

c)

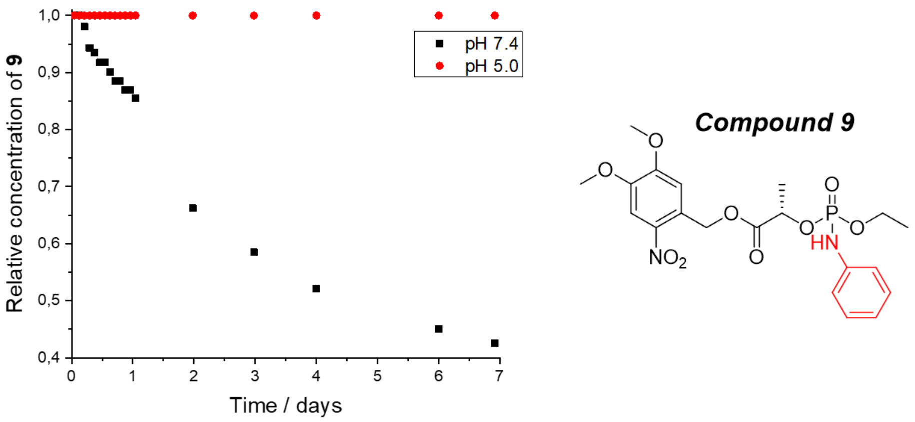

Figure S8. Concentration profiles of linkers **7**–**9** (a-c) extracted from ^{31}P NMR spectra shown above.

2 Synthesis

Synthesis of linkers

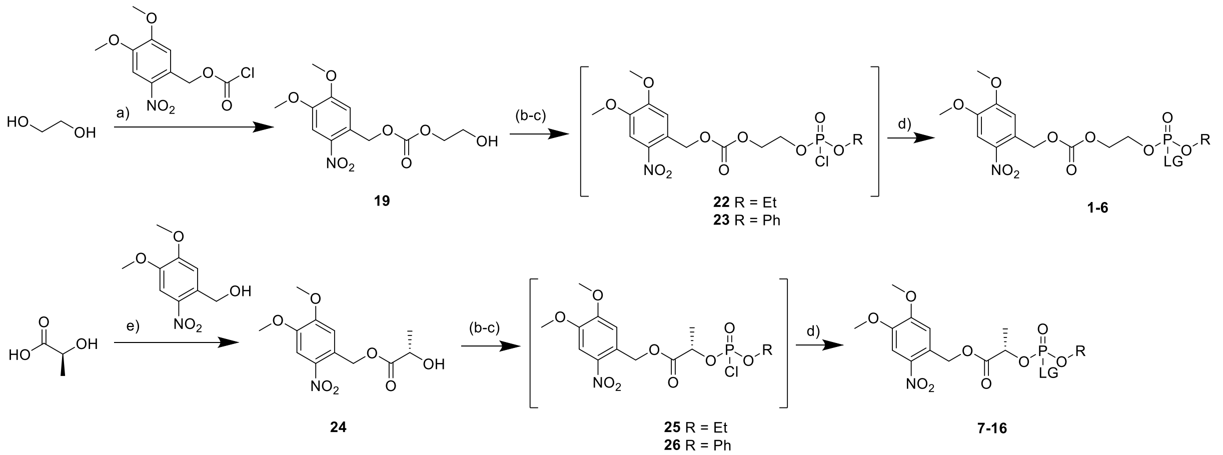

Scheme S1. *Reaction conditions*: a) pyridine, THF, 0 °C to 25 °C, 16 h; b) ethyl dichlorophosphate **20**, TEA (1.3 eq.), toluene, 25 °C, 16 h; c) phenyl dichlorophosphate **21**, TEA (1.3 eq.), toluene, 25 °C, 16 h; d) the corresponding amine, TEA (1.0 eq.), toluene, 25 °C, until the completion of the reaction (1–24 h); e) *p*-TSA.xH_2O, toluene, reflux, 16 h.

Synthesis of model prodrugs

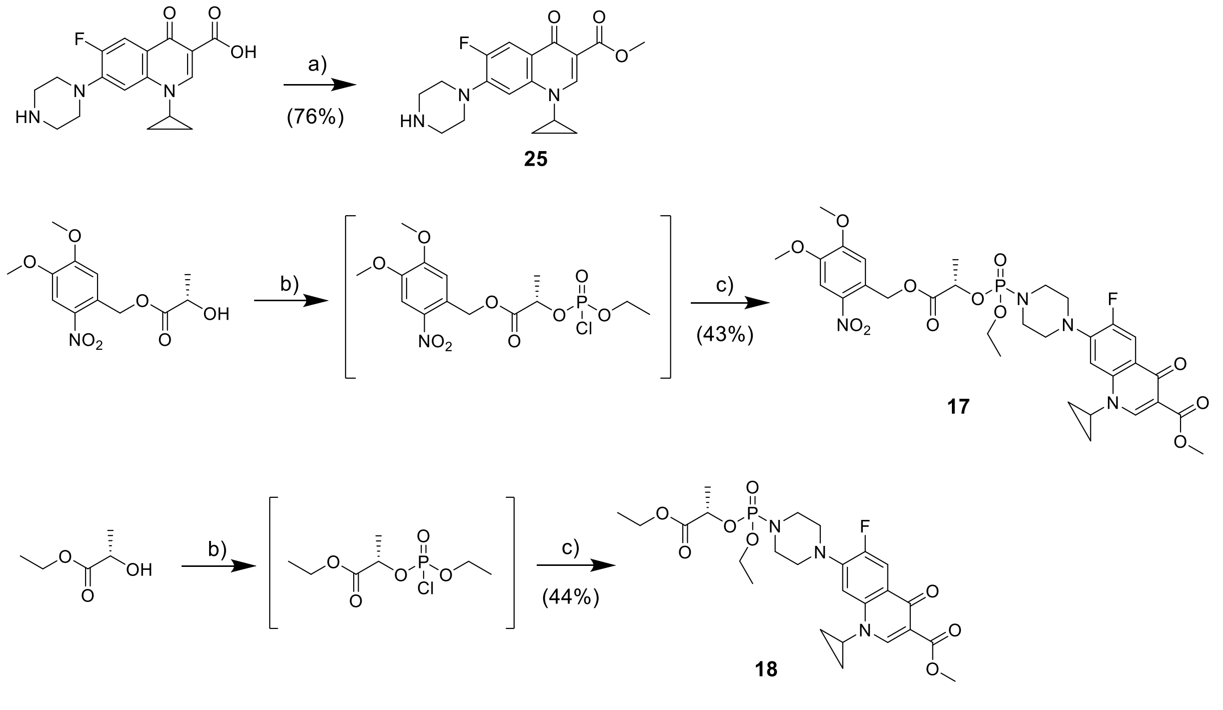

Scheme S2. *Reaction conditions*: a) $SOCl_2$, MeOH, 0 °C to reflux, 16 h; b) ethyl dichlorophosphate **20**, TEA (1.3 eq.), toluene, 25 °C, 16 h; c) **25**, TEA (1.0 eq.), toluene, 25 °C, 1 h.

Amine screening

We used the general procedure for the synthesis of the lactate-based linkers **7**–**9** for further amine screening (Scheme S3). ^{31}P NMR spectroscopy in $CDCl_3$ was used for reaction monitoring, as it provides qualitative insight into the reaction progress. The final linker should be obtained as a mixture of two diastereoisomers, therefore the appearance of a pair of signals in the spectra should be the indicator of its formation. A pair of ^{31}P NMR signals with high intensity was found after the addition of the corresponding amine to a solution of **25** in the case of 2-aminopyrimidine (Figure S9a), imidazole (Figure S9b) and 2-aminobenzimidazole (Figure S9f). This made us assume that the desired linker was being formed as the main product, so flash chromatography was performed. Isolation of the product by flash chromatography on silica-gel (hexane – ethyl acetate gradient) was successful only in the case of 2-aminopyrimidine (linker **13**), while the products of imidazole and 2-aminobenzimidazole probably decomposed during the isolation (chromatography). Change of the base from TEA to NMI in the reaction of 2-aminobenzimidazole with **25** resulted in the formation of higher amounts of the side products, thus we did not optimize this reaction further.

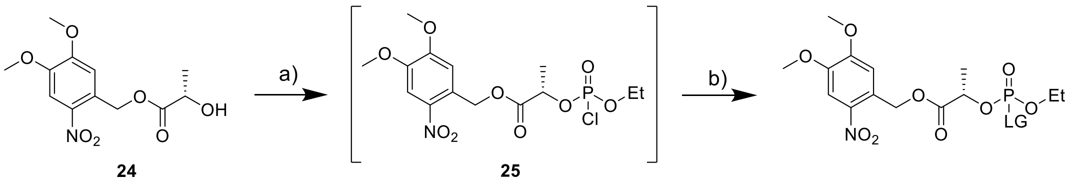

Scheme S3. *Reaction conditions*: a) ethyl dichlorophosphate **20**, TEA (1.3 eq.), toluene, 25 °C, 16 h; b) the corresponding heterocyclic amine, TEA or NMI (1.0 eq.), toluene, 25 °C, 1–24 h.

In the case of indoline and 2-aminobenzimidazole, we also tried the opposite approach – starting by phosphorylation of amine, followed by addition of **24** (Scheme S4). While 2-aminobenzimidazole didn't react with **20** under our non-optimized conditions, the reaction of indoline with **20** resulted in the formation of intermediate, which didn't further react with **24**.

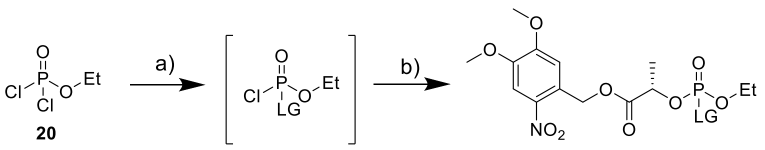

Scheme S4. *Reaction conditions*: a) the corresponding heterocyclic amine, TEA (1.3 eq.), toluene, 25 °C, 16 h; b) **24** (1.0 eq.), TEA (1.0 eq.), toluene, 25 °C, 16 h.

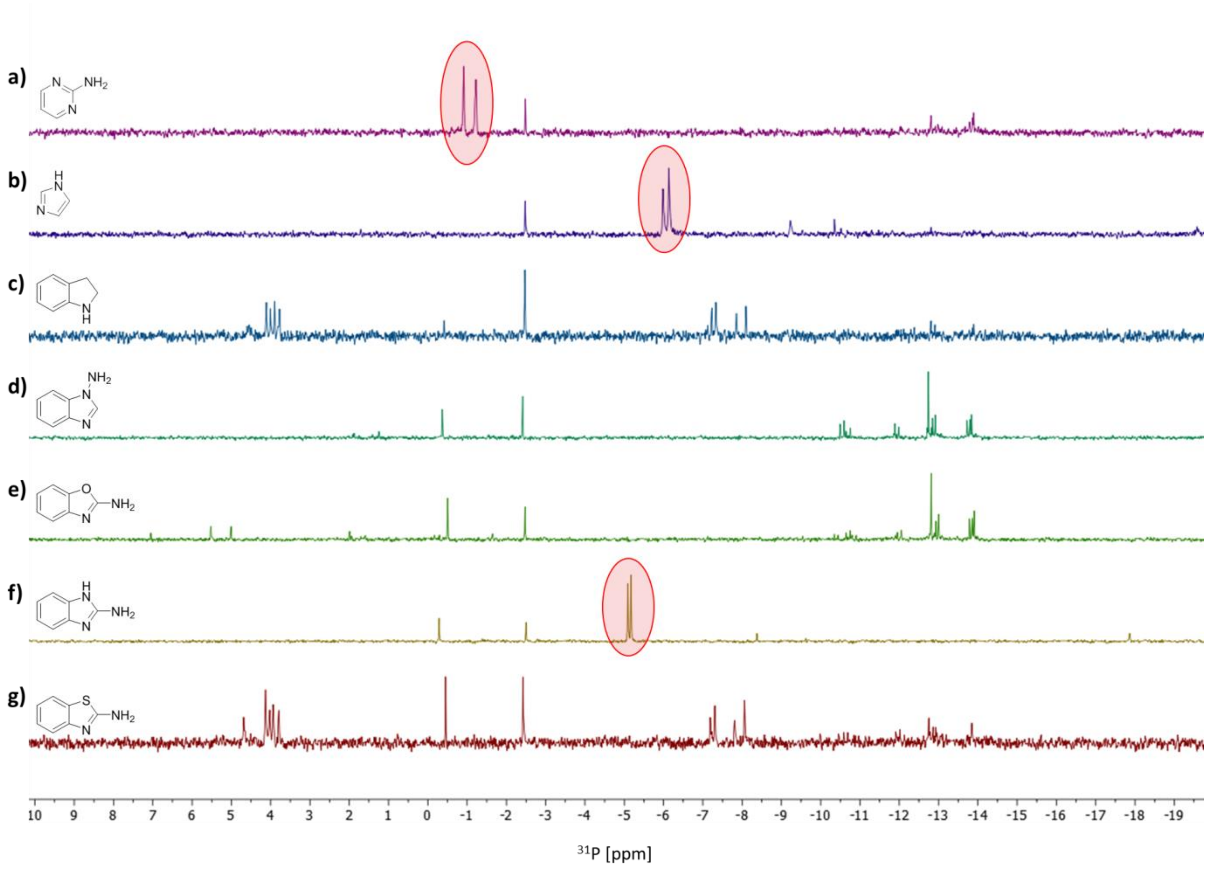

Figure S9. ^{31}P NMR spectra of the crude reaction mixtures after phosphorylation (using procedure shown on Scheme S3) of heterocyclic amines measured in $CDCl_3$.

3 Compound characterization: NMR spectra

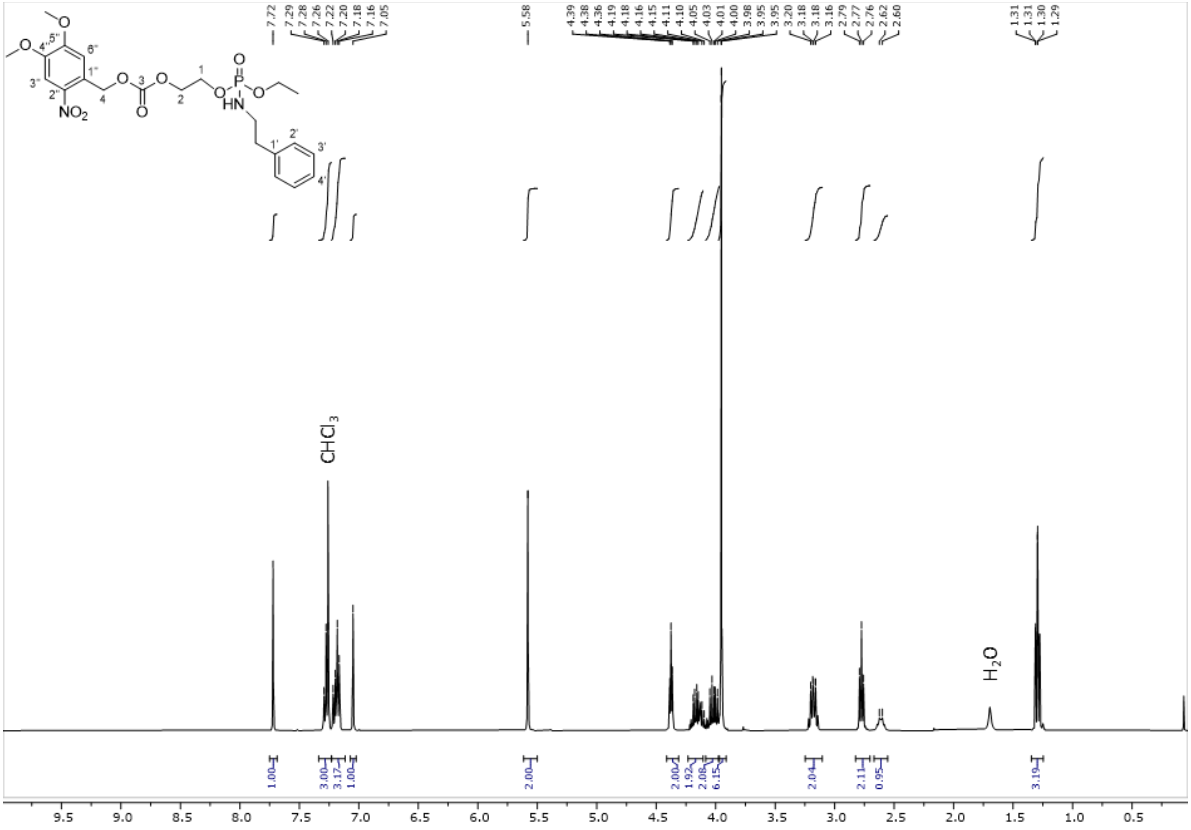

Figure S10. ^{1}H spectrum (ppm) of **1** in $CDCl_3$ measured at 25 °C.

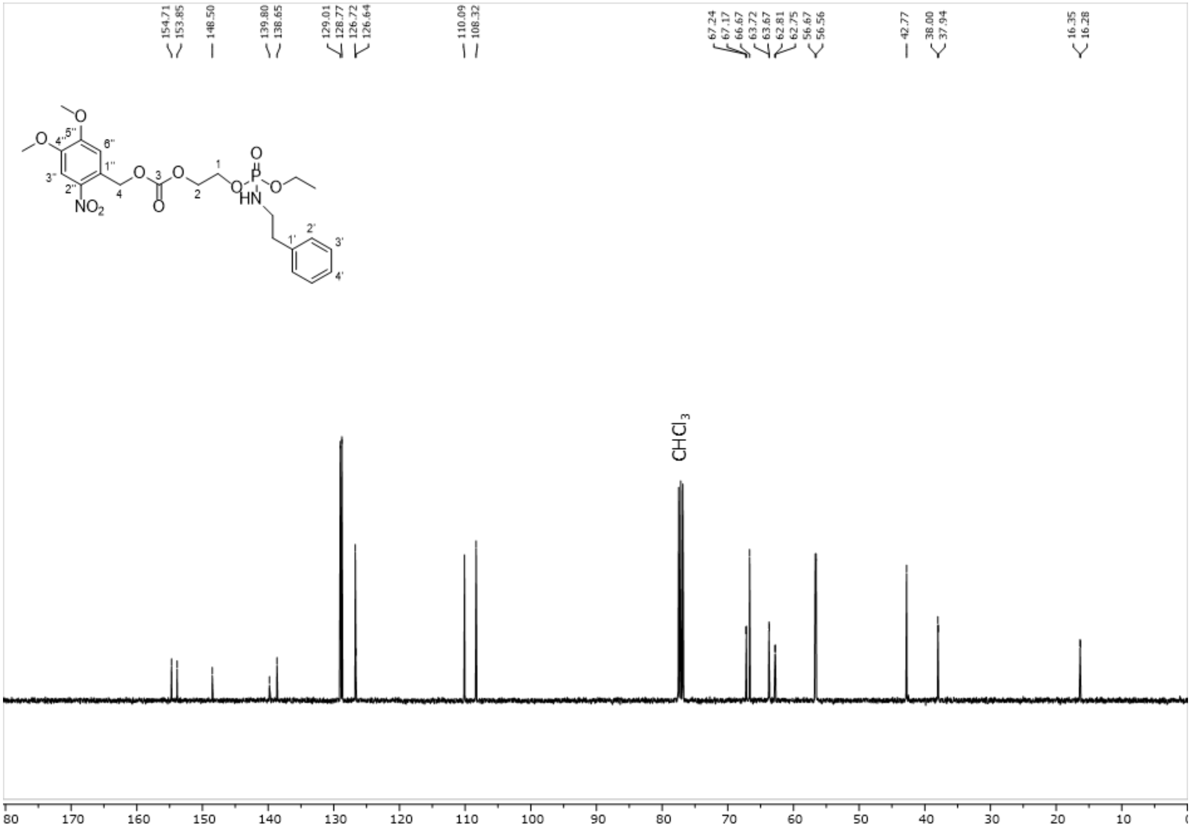

Figure S11. ^{13}C spectrum of **1** in $CDCl_3$ measured at 25 °C.

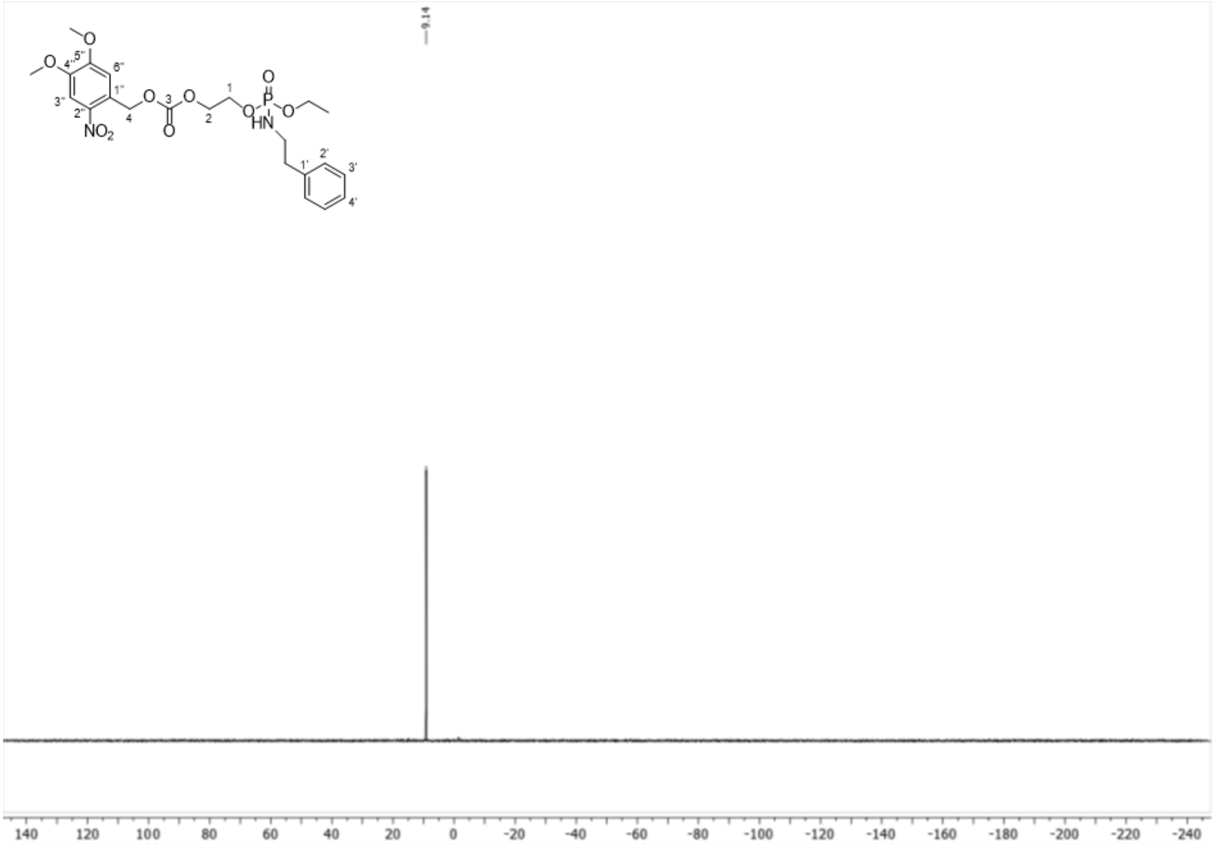

Figure S12. ^{31}P spectrum (ppm) of **1** in $CDCl_3$ measured at 25 °C.

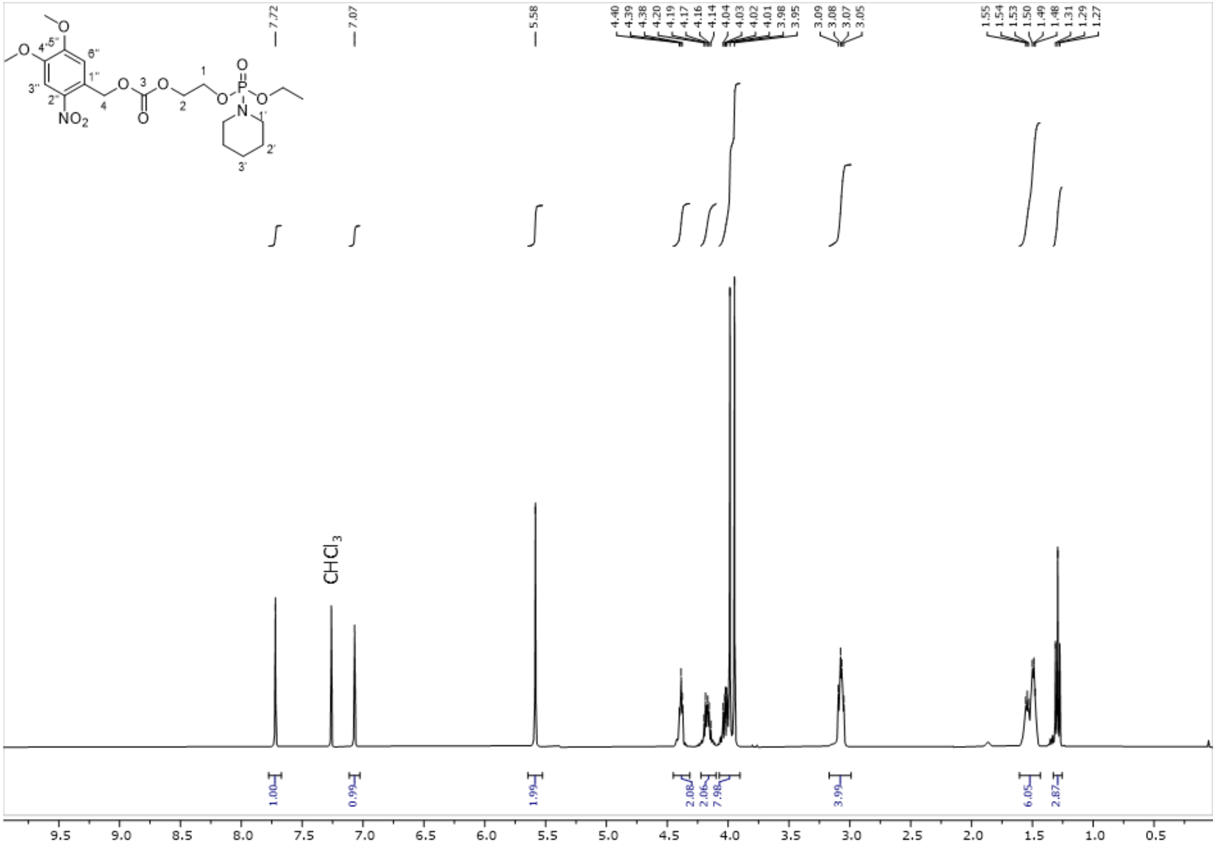

Figure S13. ^{1}H spectrum (ppm) of **2** in $CDCl_3$ measured at 25 °C.

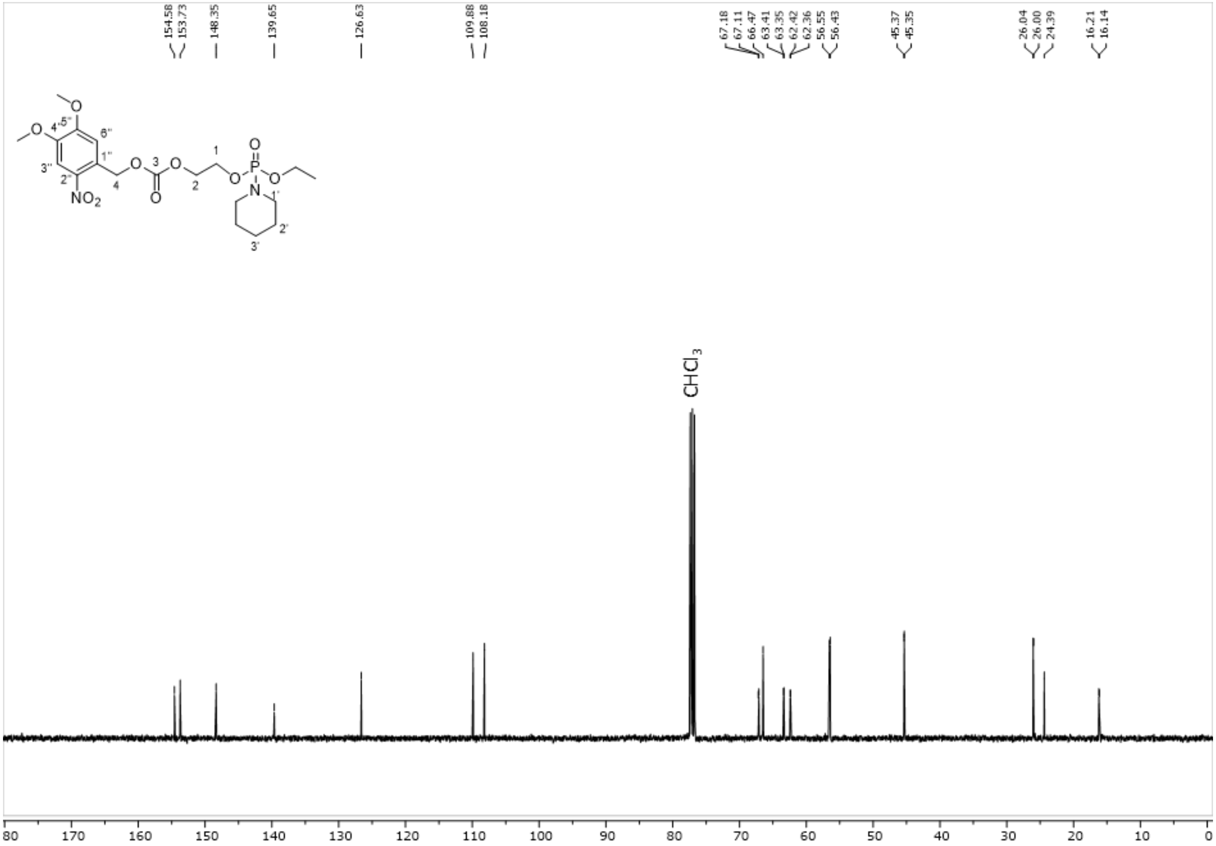

Figure S14. ^{13}C spectrum (ppm) of **2** in $CDCl_3$ measured at 25 °C.

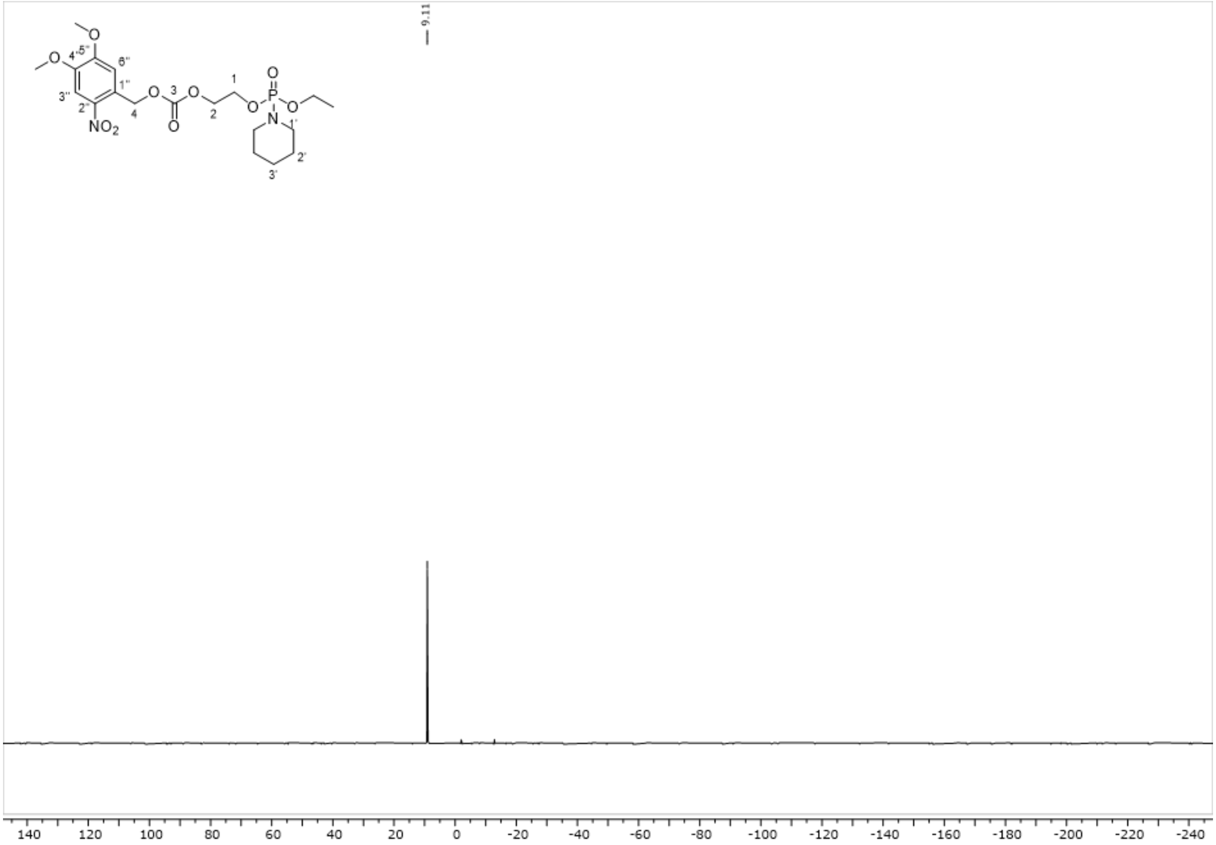

Figure S15. ^{31}P spectrum (ppm) of **2** in $CDCl_3$ measured at 25 °C.

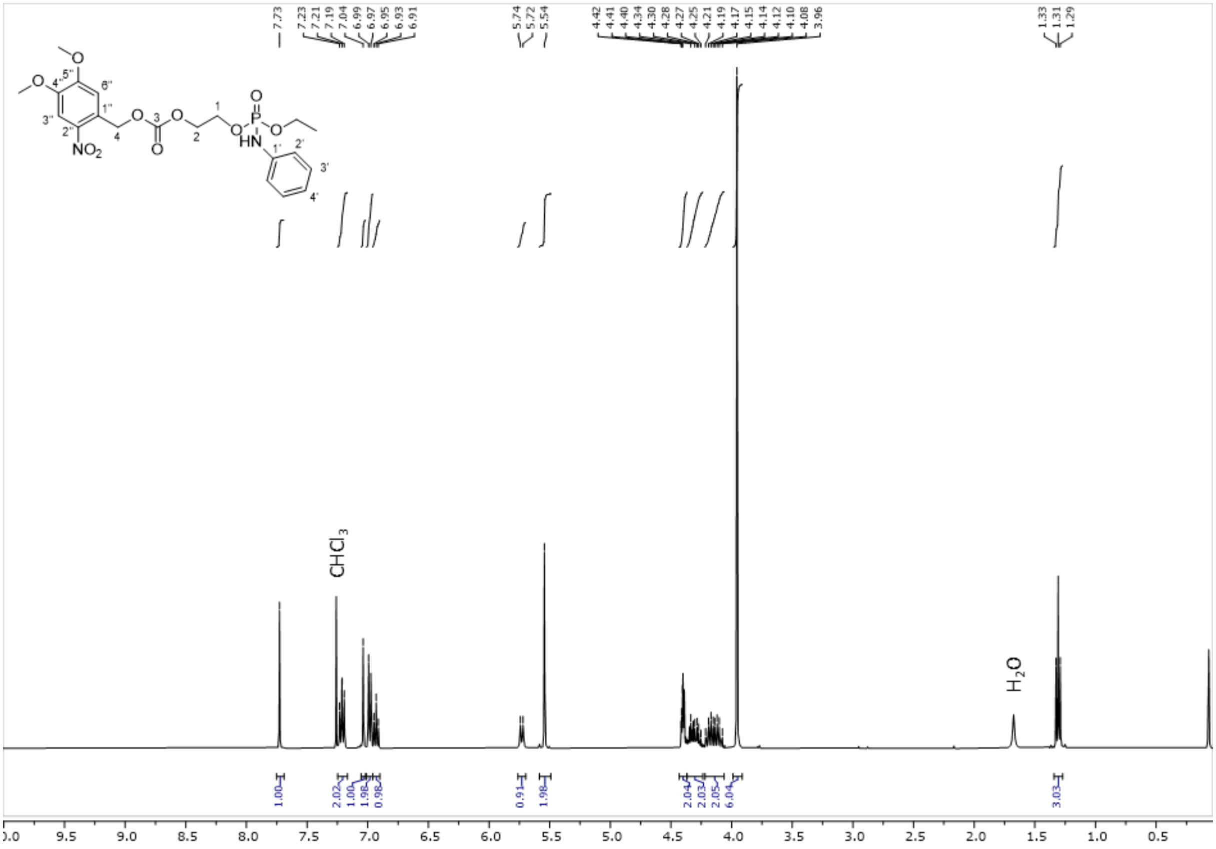

Figure S16. ^{1}H spectrum (ppm) of **3** in $CDCl_3$ measured at 25 °C.

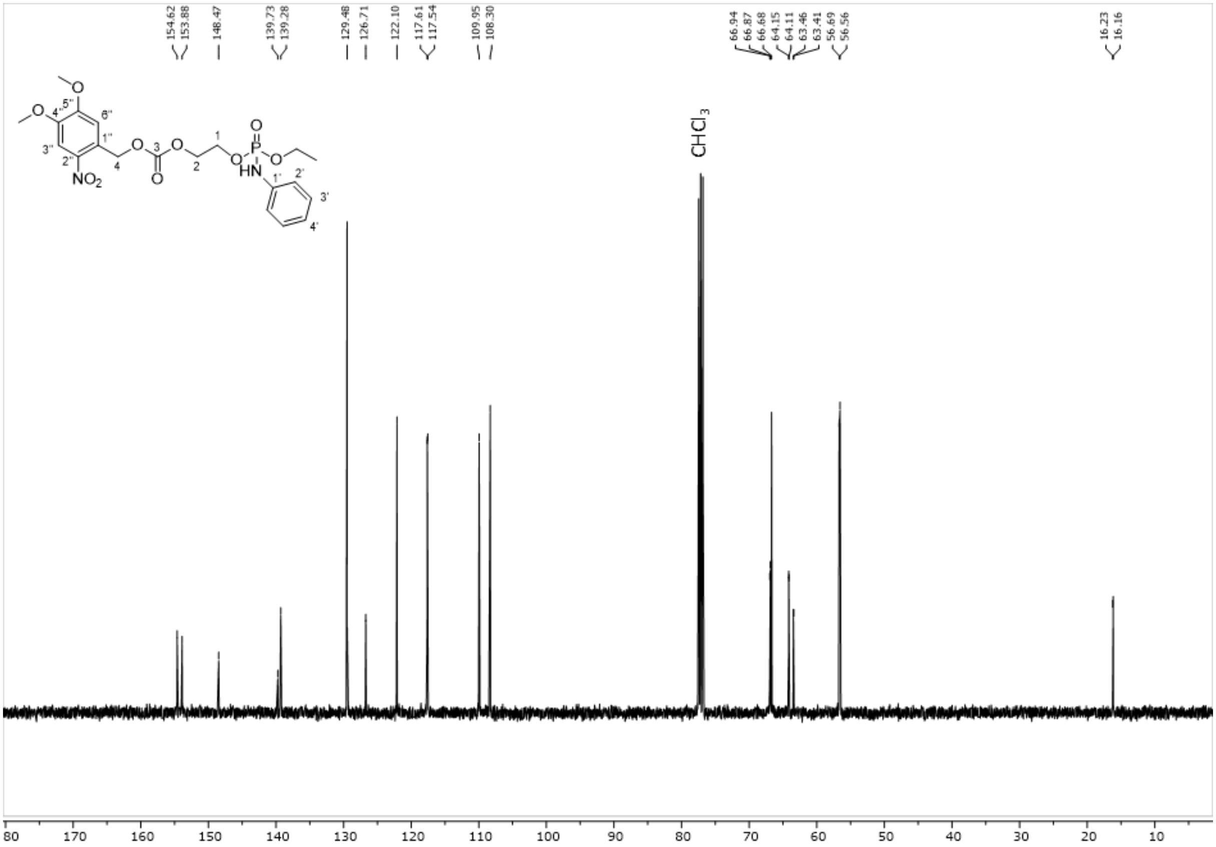

Figure S17. ^{13}C spectrum (ppm) of **3** in $CDCl_3$ measured at 25 °C.

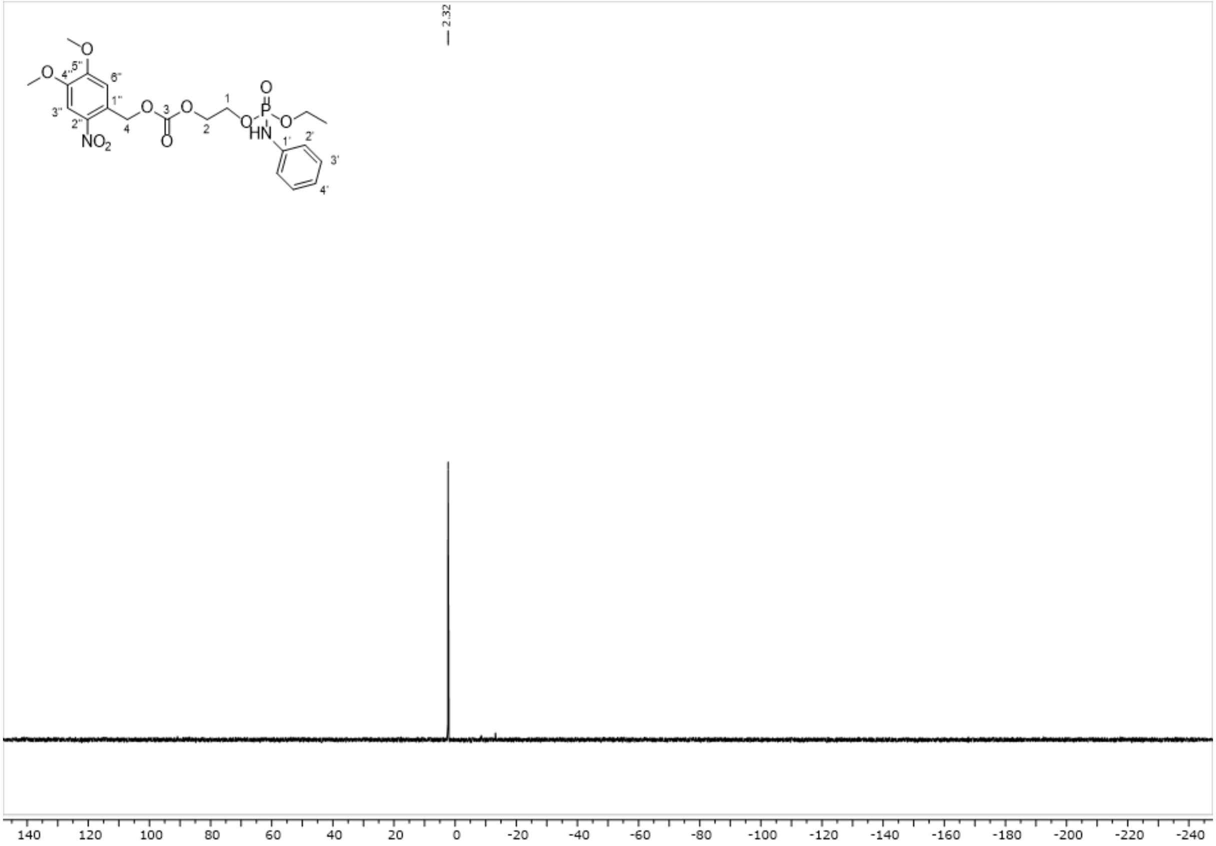

Figure S18. ^{31}P spectrum (ppm) of **3** in $CDCl_3$ measured at 25 °C.

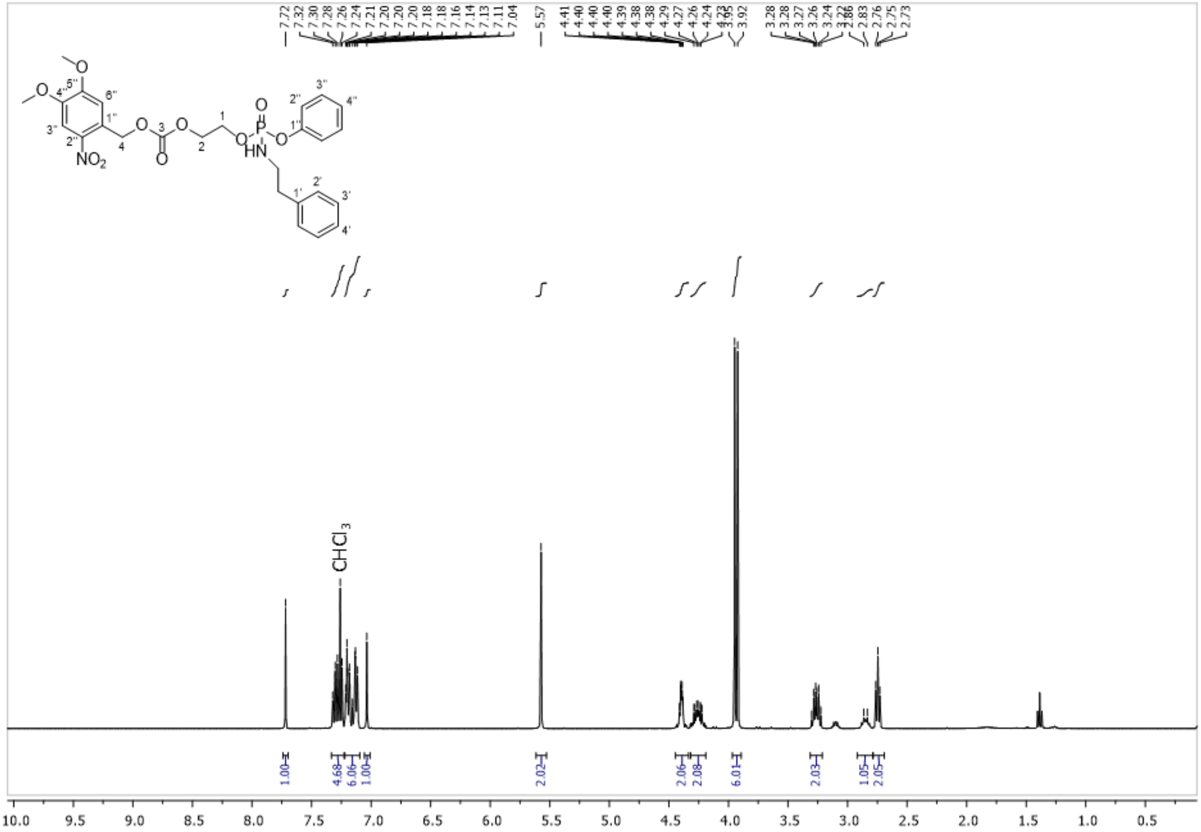

Figure S19. ^{1}H spectrum (ppm) of **4** in $CDCl_3$ measured at 25 °C.

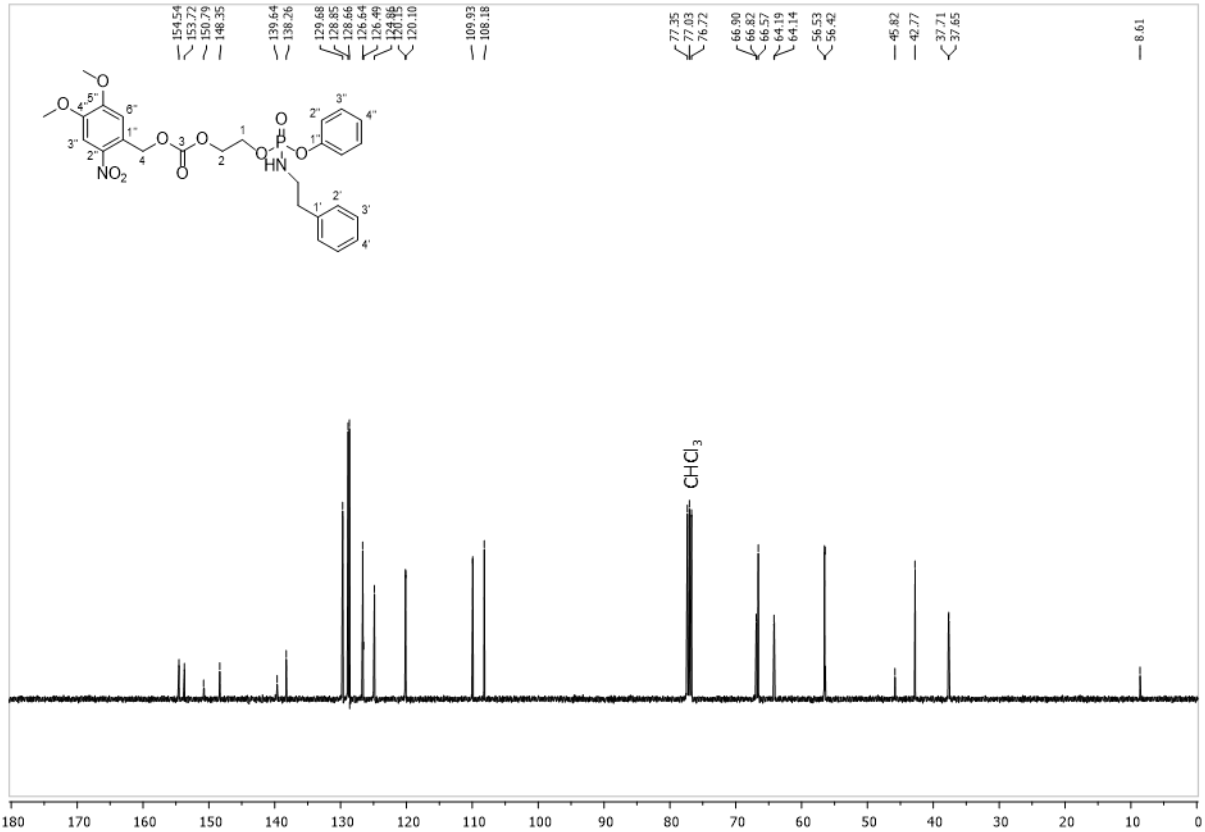

Figure S20. ^{13}C spectrum (ppm) of **4** in $CDCl_3$ measured at 25 °C.

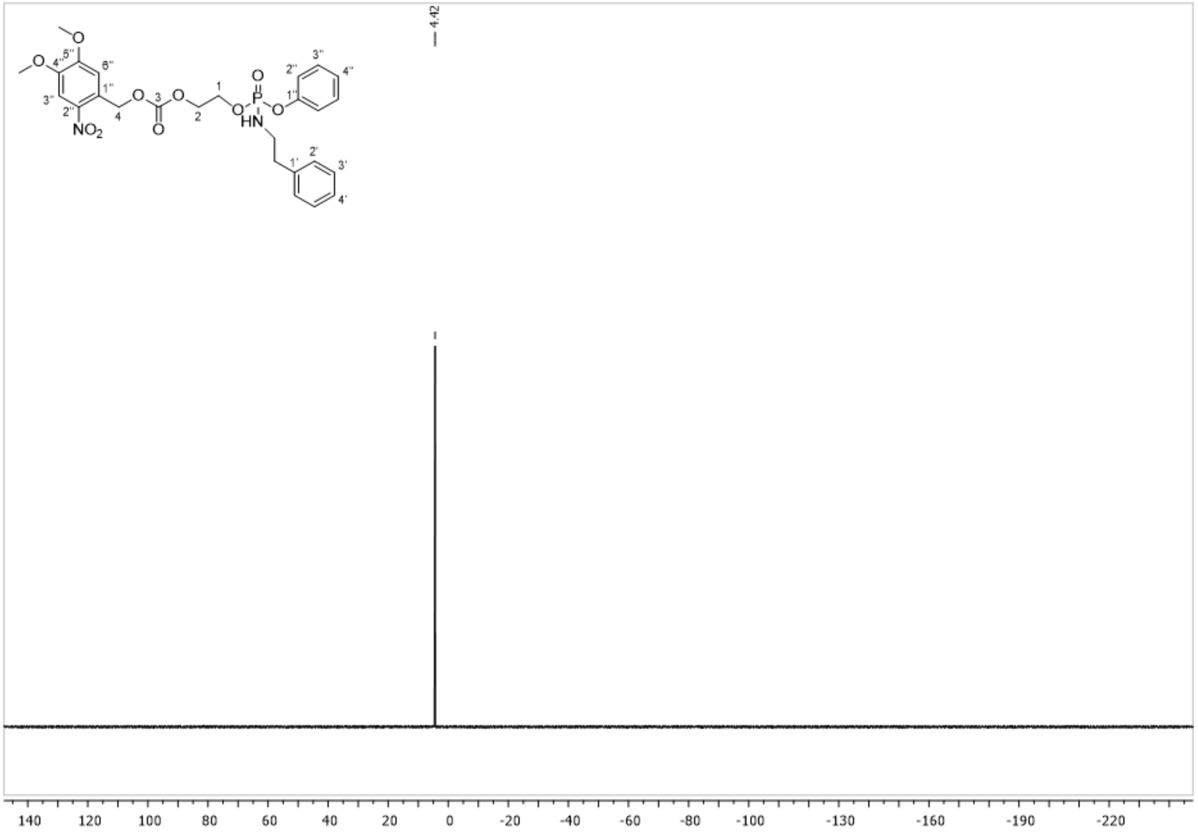

Figure S21. ^{31}P spectrum (ppm) of **4** in $CDCl_3$ measured at 25 °C.

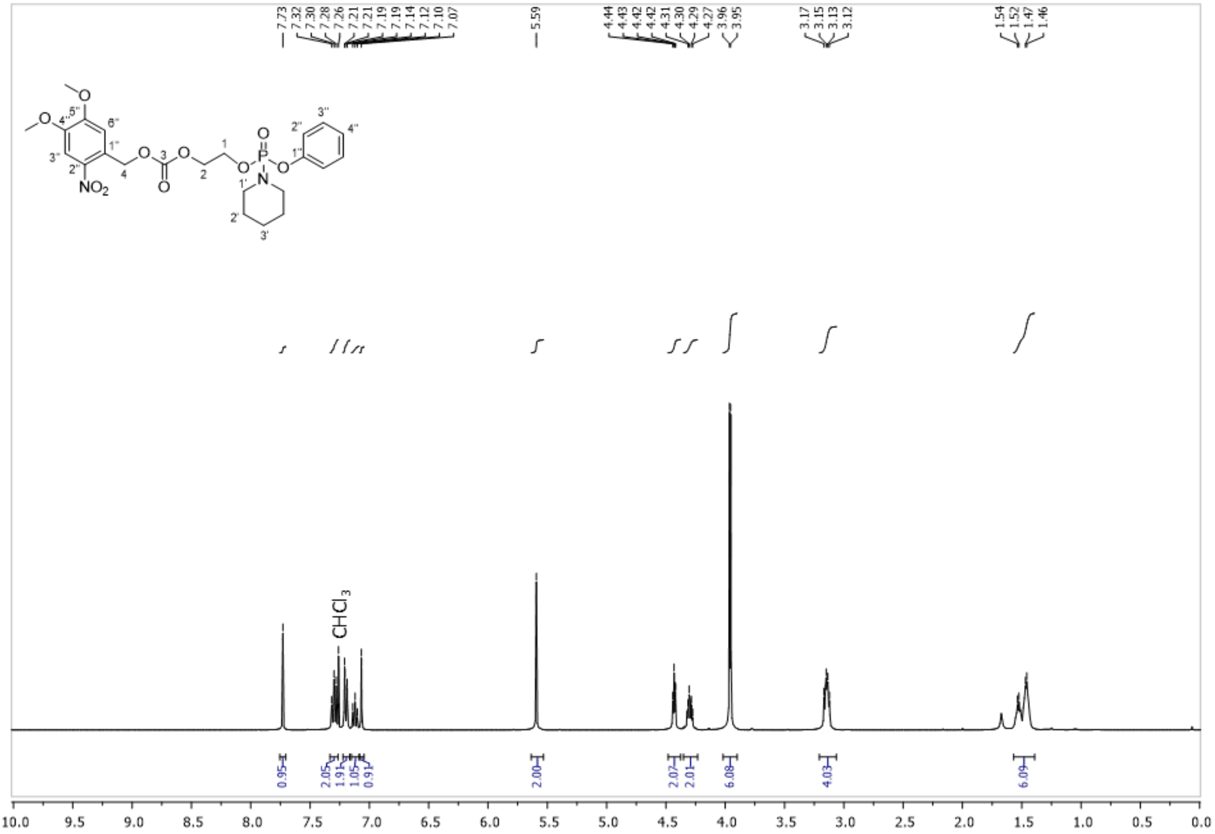

Figure S22. ^{1}H spectrum (ppm) of **5** in $CDCl_3$ measured at 25 °C.

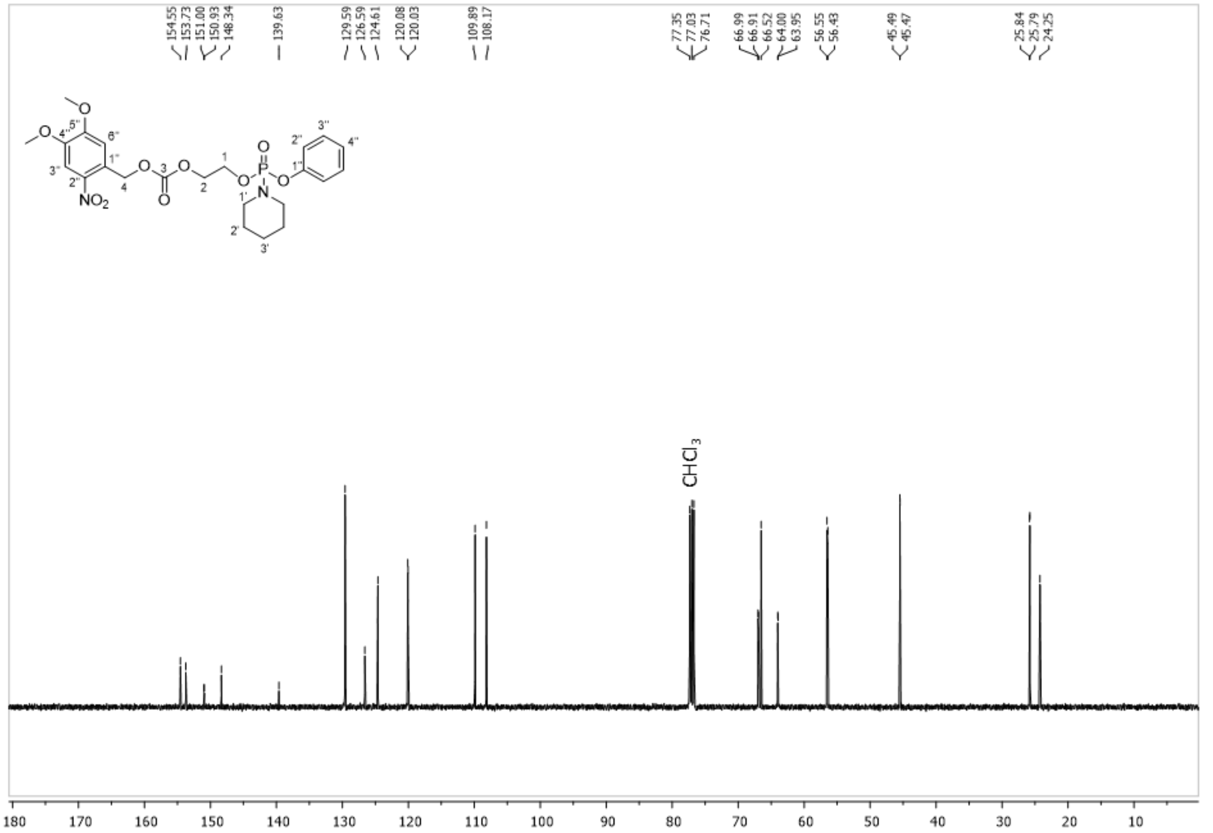

Figure S23. ^{13}C spectrum (ppm) of **5** in $CDCl_3$ measured at 25 °C.

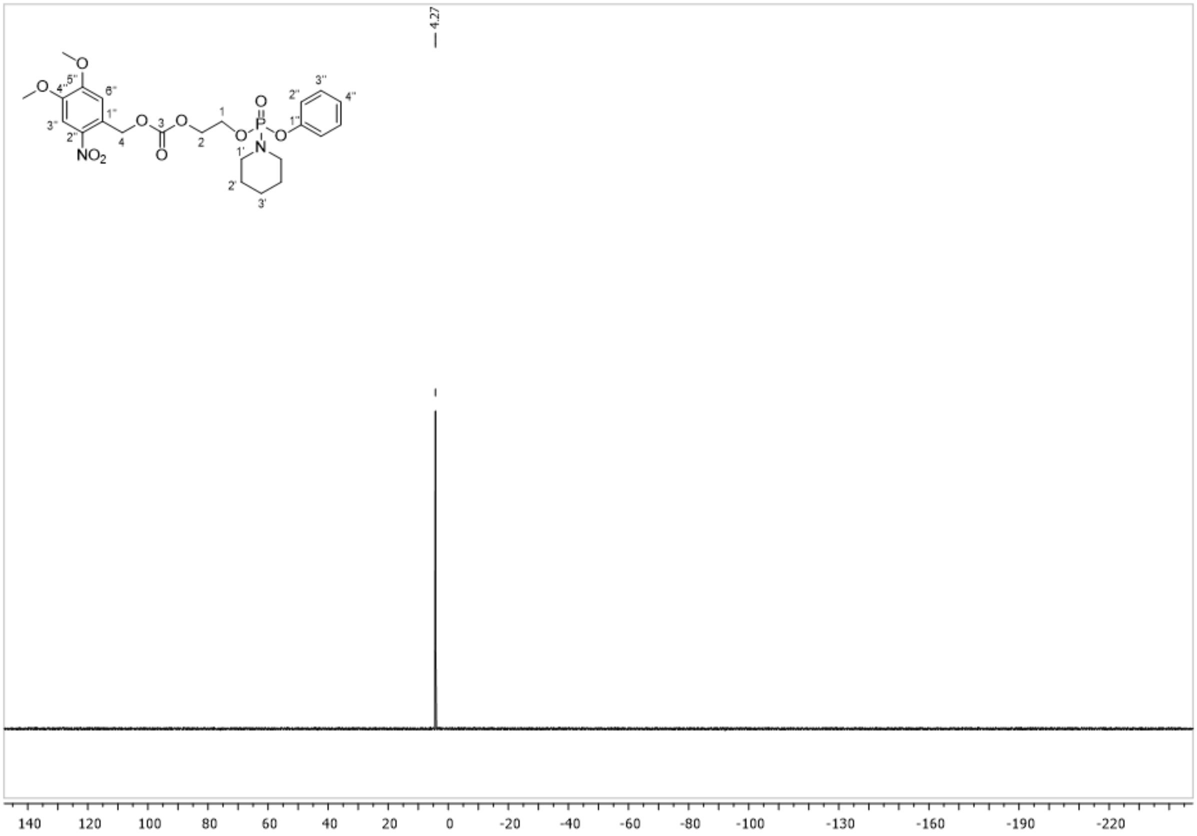

Figure S24. ^{31}P spectrum (ppm) of **5** in $CDCl_3$ measured at 25 °C.

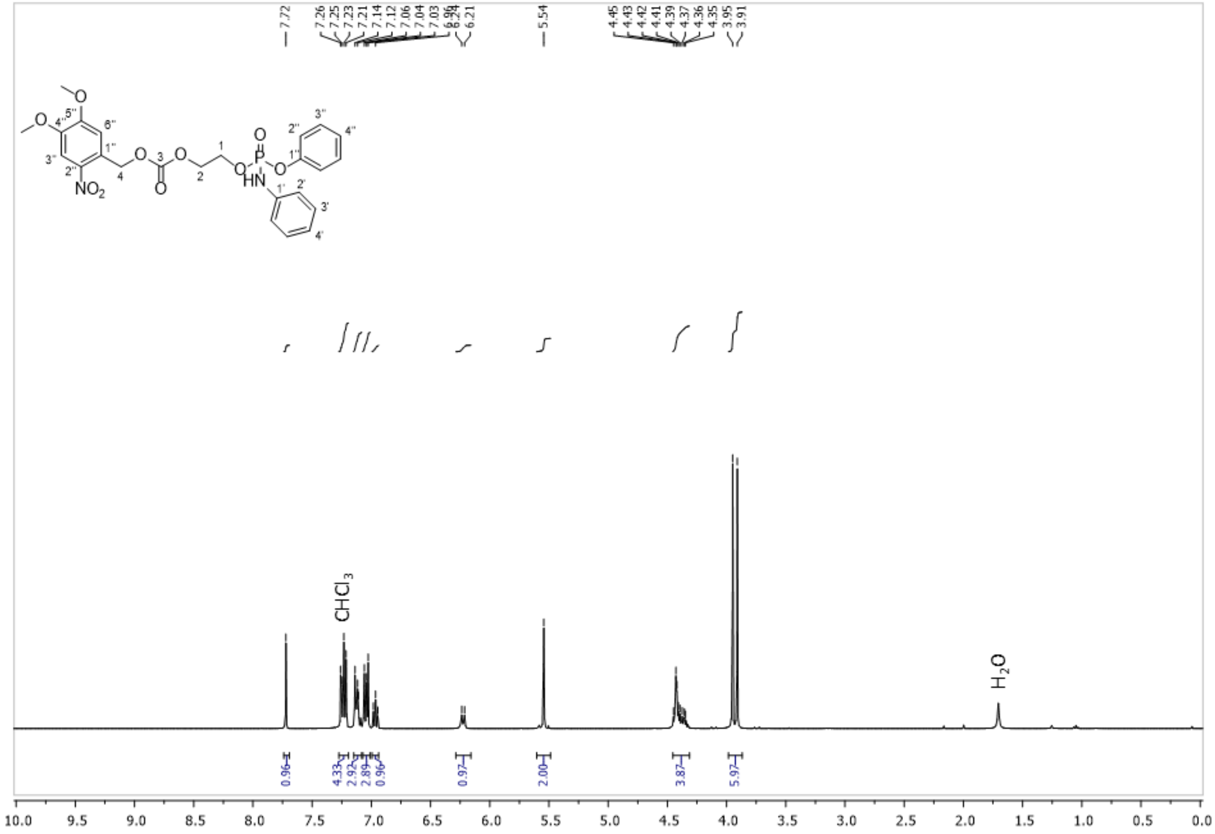

Figure S25. ^{1}H spectrum (ppm) of **6** in $CDCl_3$ measured at 25 °C.

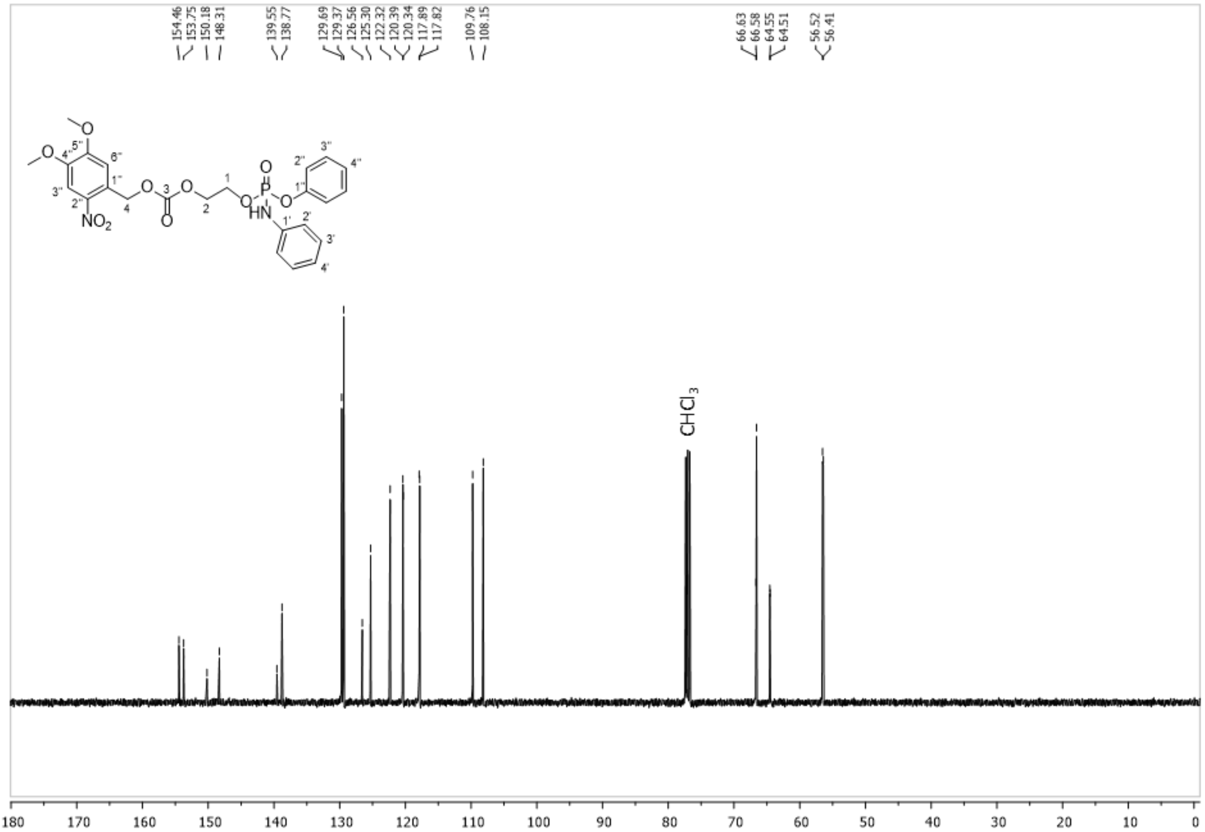

Figure S26. ^{13}C spectrum (ppm) of **6** in $CDCl_3$ measured at 25 °C.

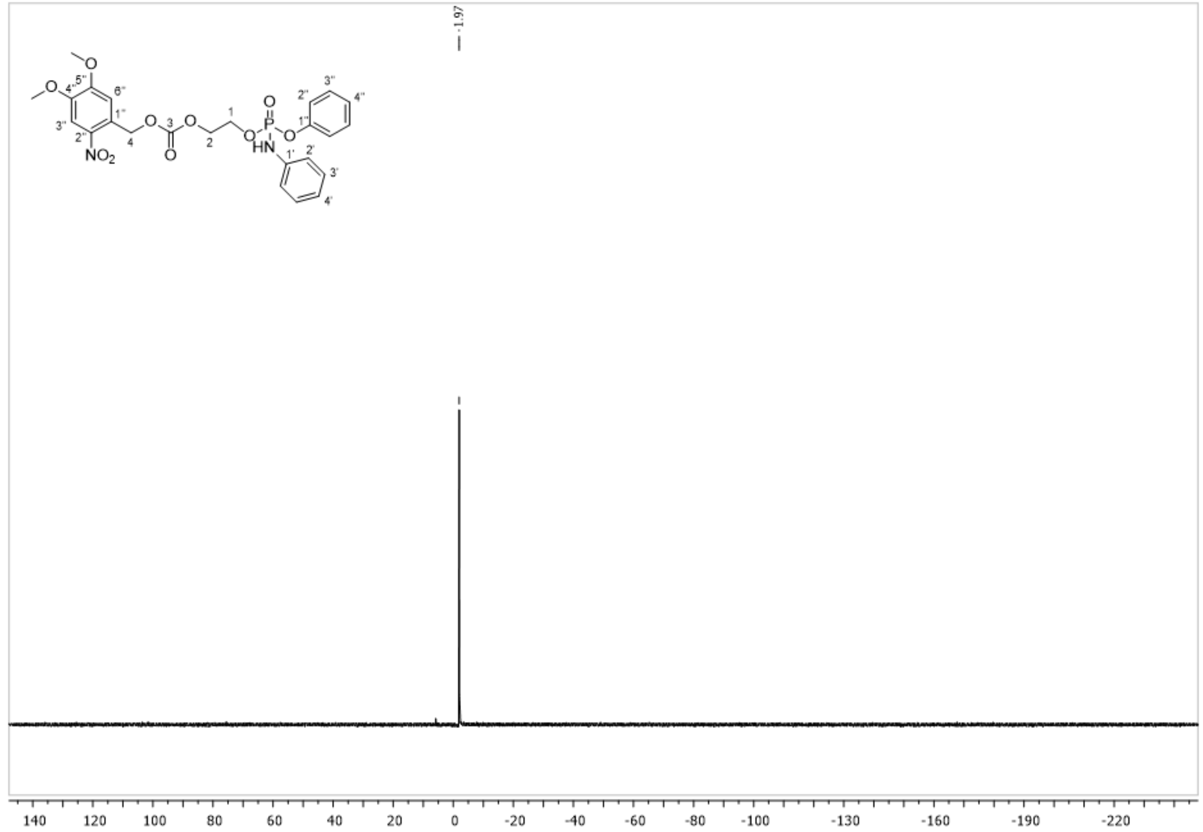

Figure S27. ^{31}P spectrum (ppm) of **6** in $CDCl_3$ measured at 25 °C.

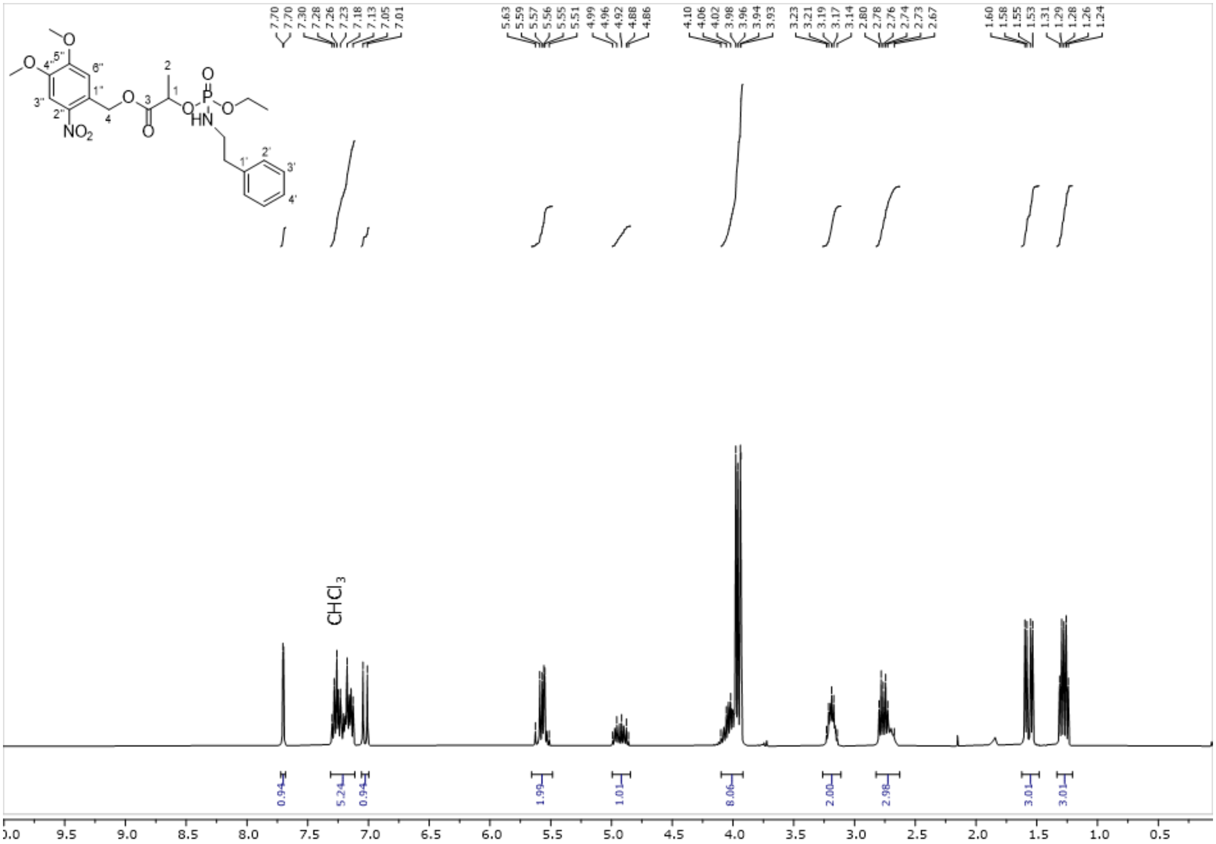

Figure S28. ^{1}H spectrum (ppm) of **7** in $CDCl_3$ measured at 25 °C.

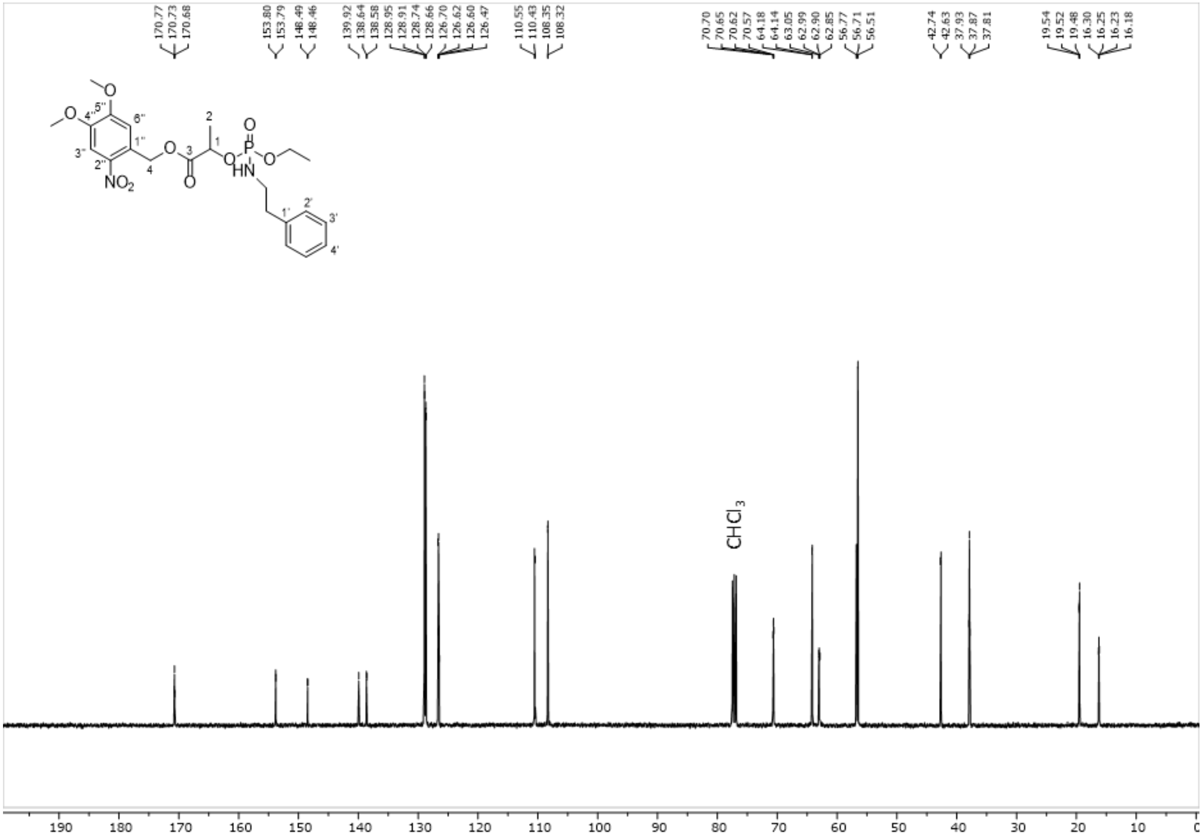

Figure S29. ^{13}C spectrum (ppm) of **7** in $CDCl_3$ measured at 25 °C.

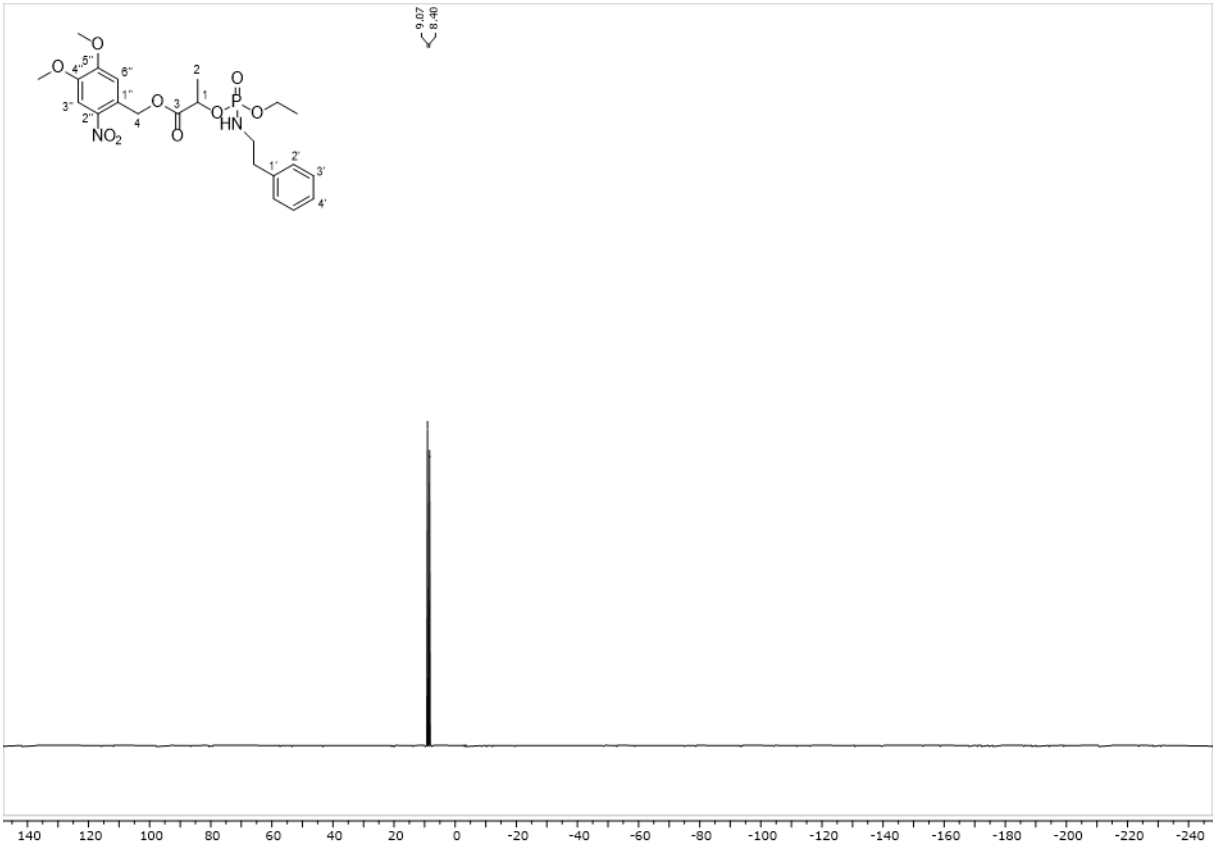

Figure S30. ^{31}P spectrum (ppm) of **7** in $CDCl_3$ measured at 25 °C.

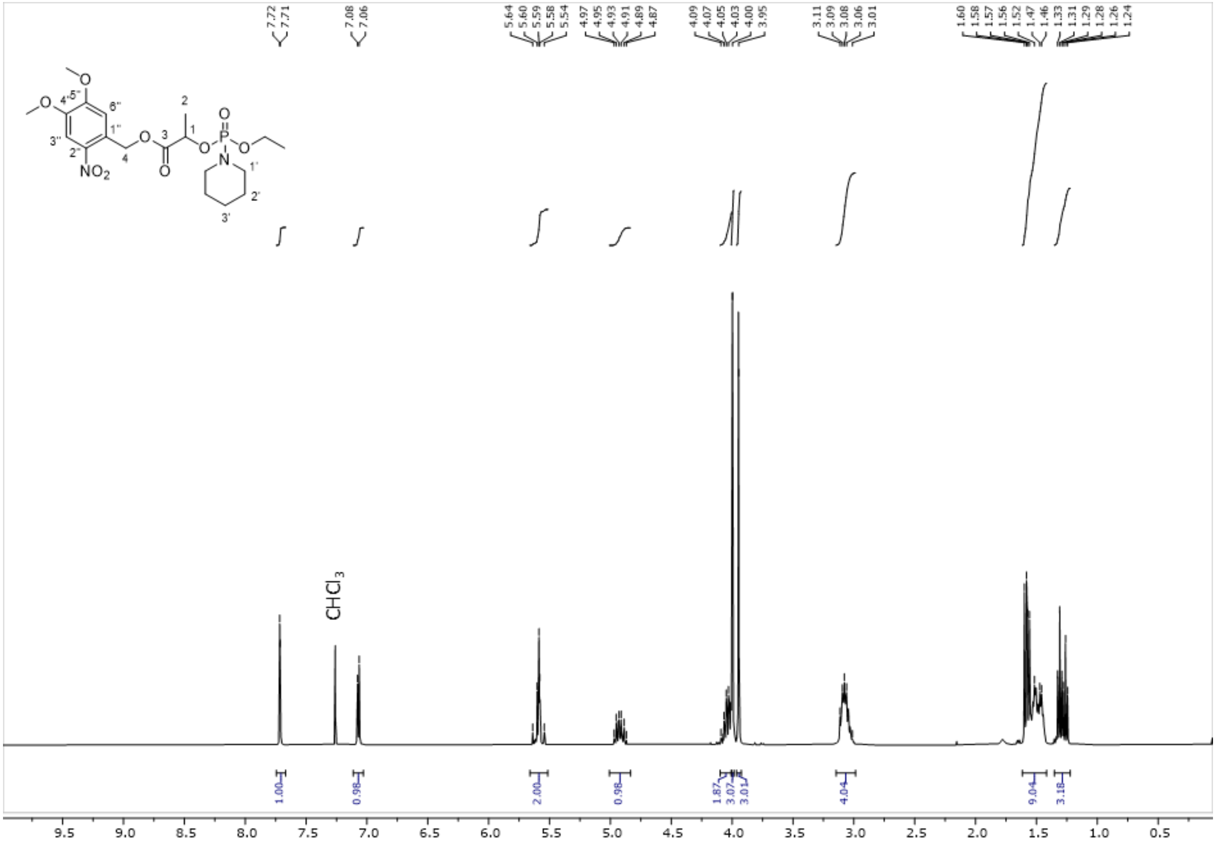

Figure S31. ^{1}H spectrum (ppm) of **8** in $CDCl_3$ measured at 25 °C.

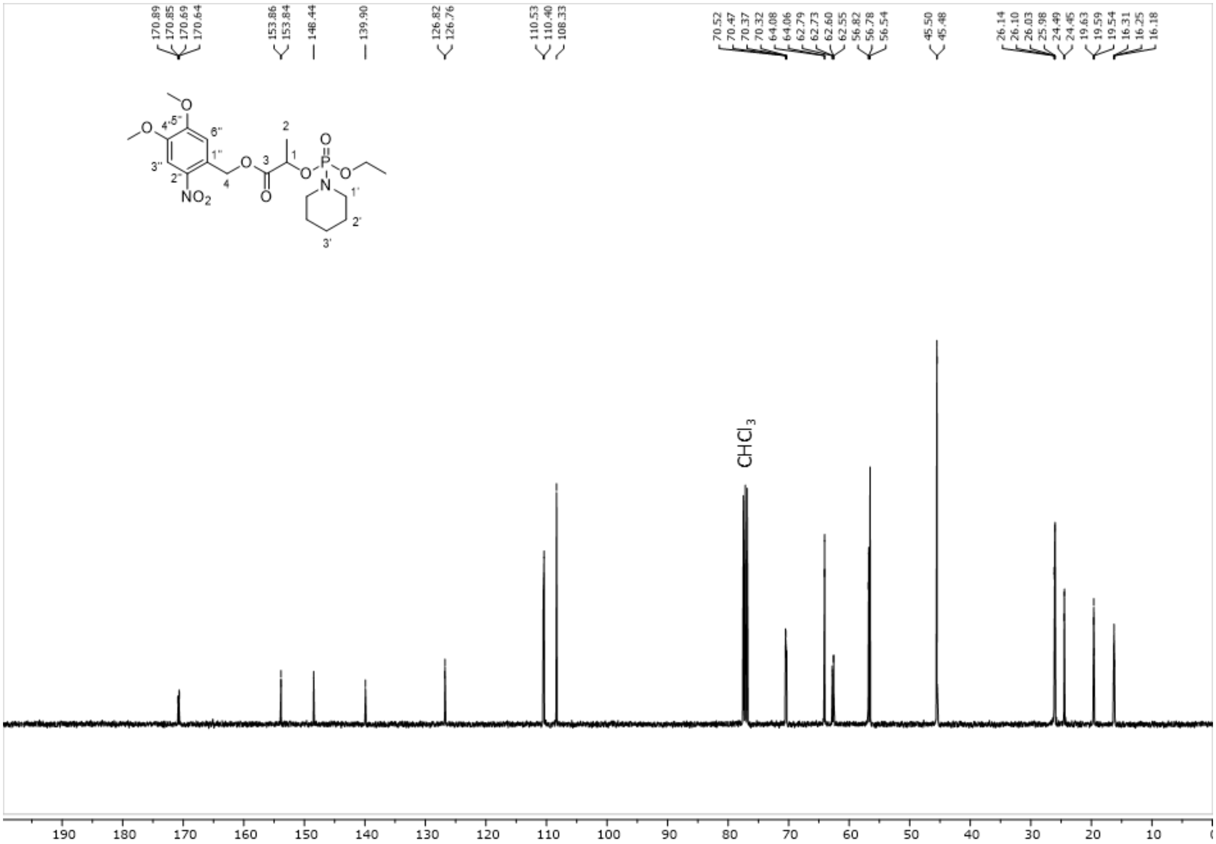

Figure S32. ^{13}C spectrum (ppm) of **8** in $CDCl_3$ measured at 25 °C.

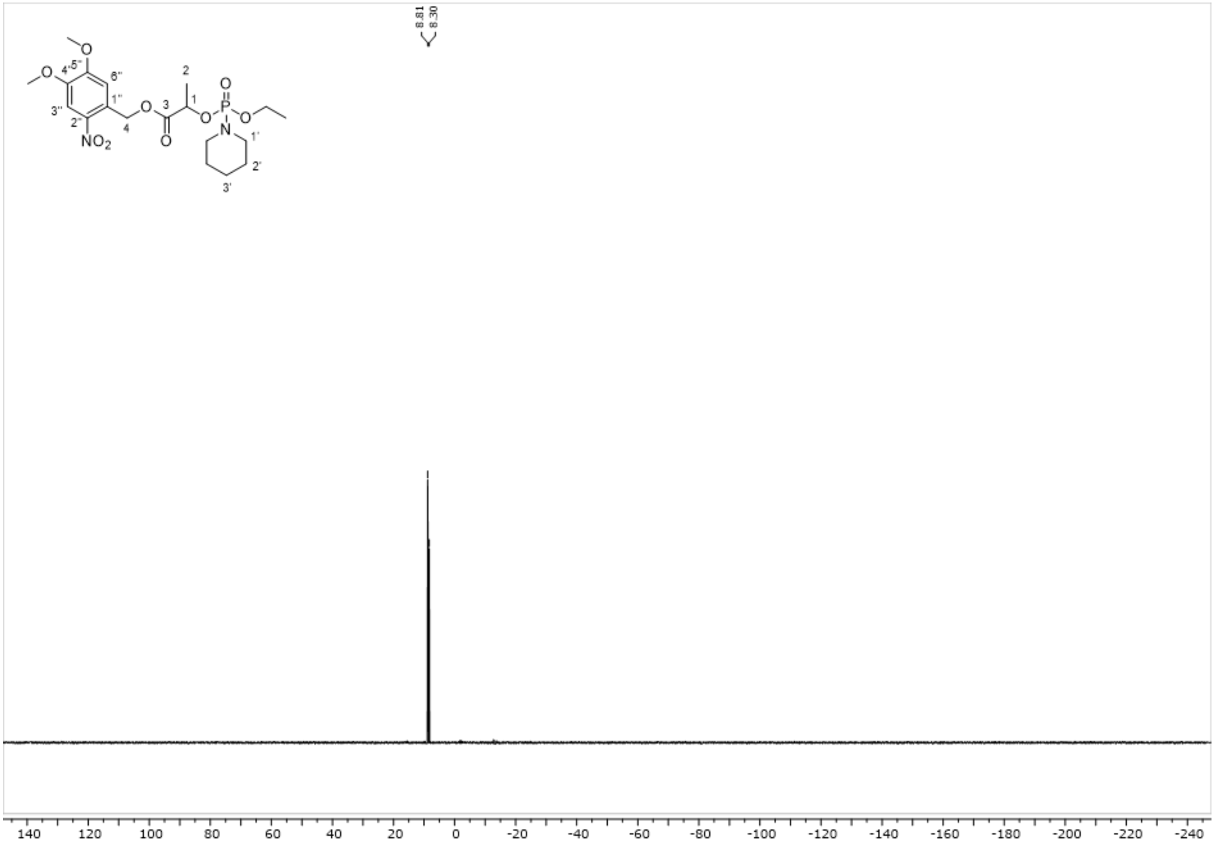

Figure S33. ^{31}P spectrum (ppm) of **8** in $CDCl_3$ measured at 25 °C.

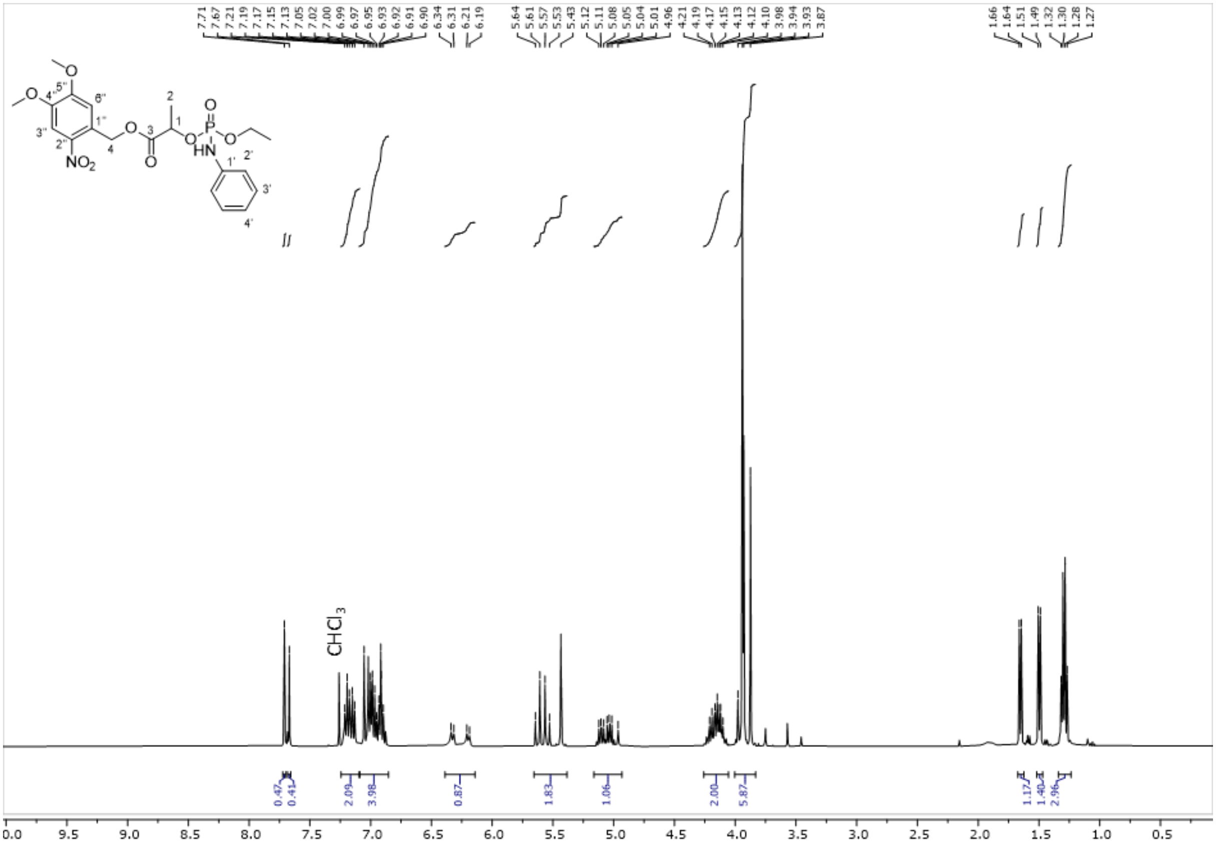

Figure S34. ^{1}H spectrum (ppm) of **9** in $CDCl_3$ measured at 25 °C.

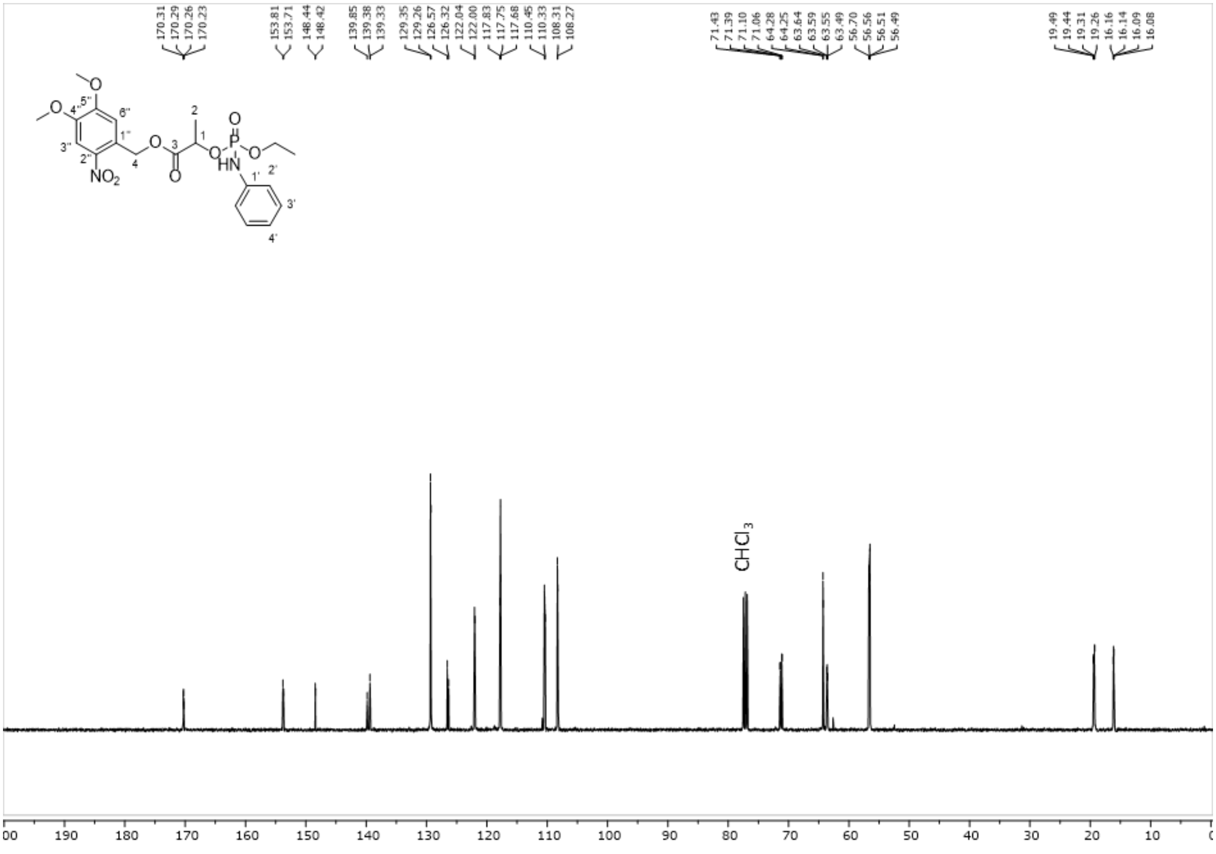

Figure S35. ^{13}C spectrum (ppm) of **9** in $CDCl_3$ measured at 25 °C.

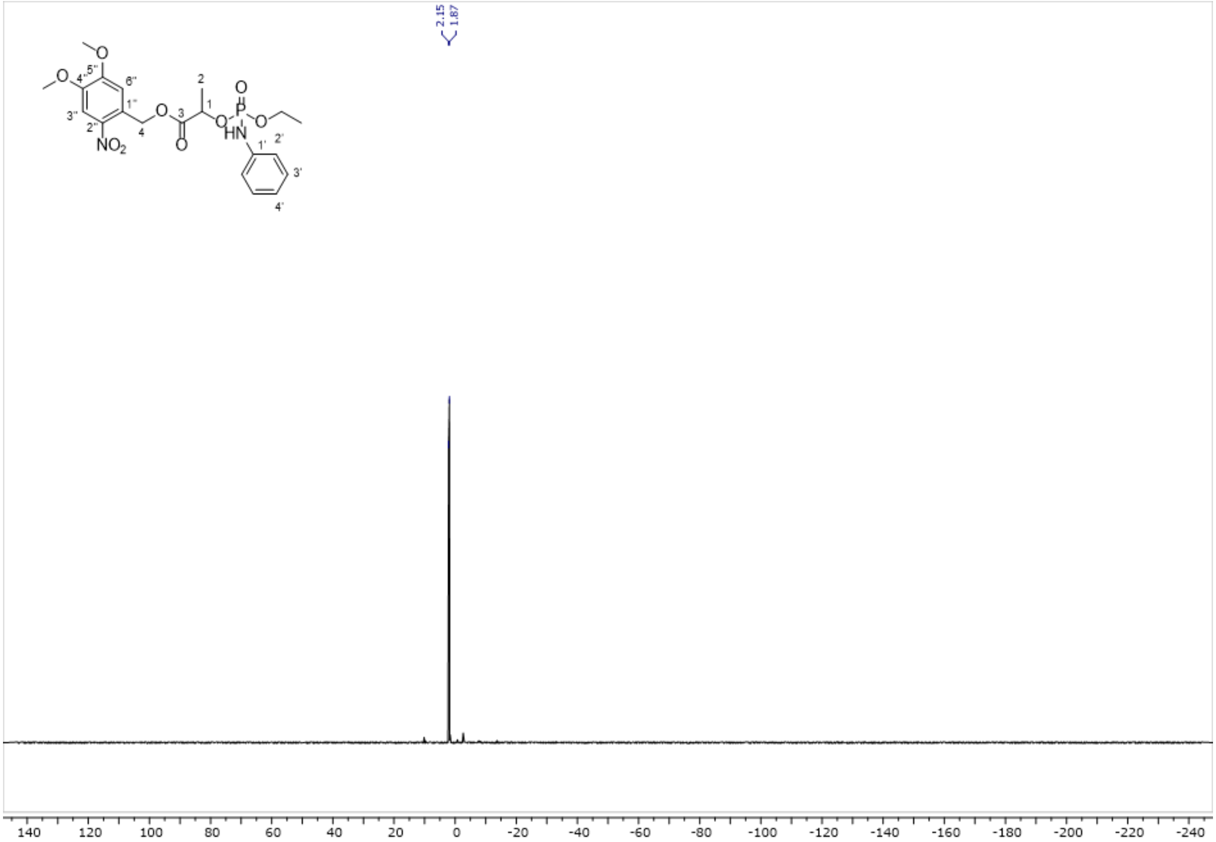

Figure S36. ^{31}P spectrum (ppm) of **9** in $CDCl_3$ measured at 25 °C.

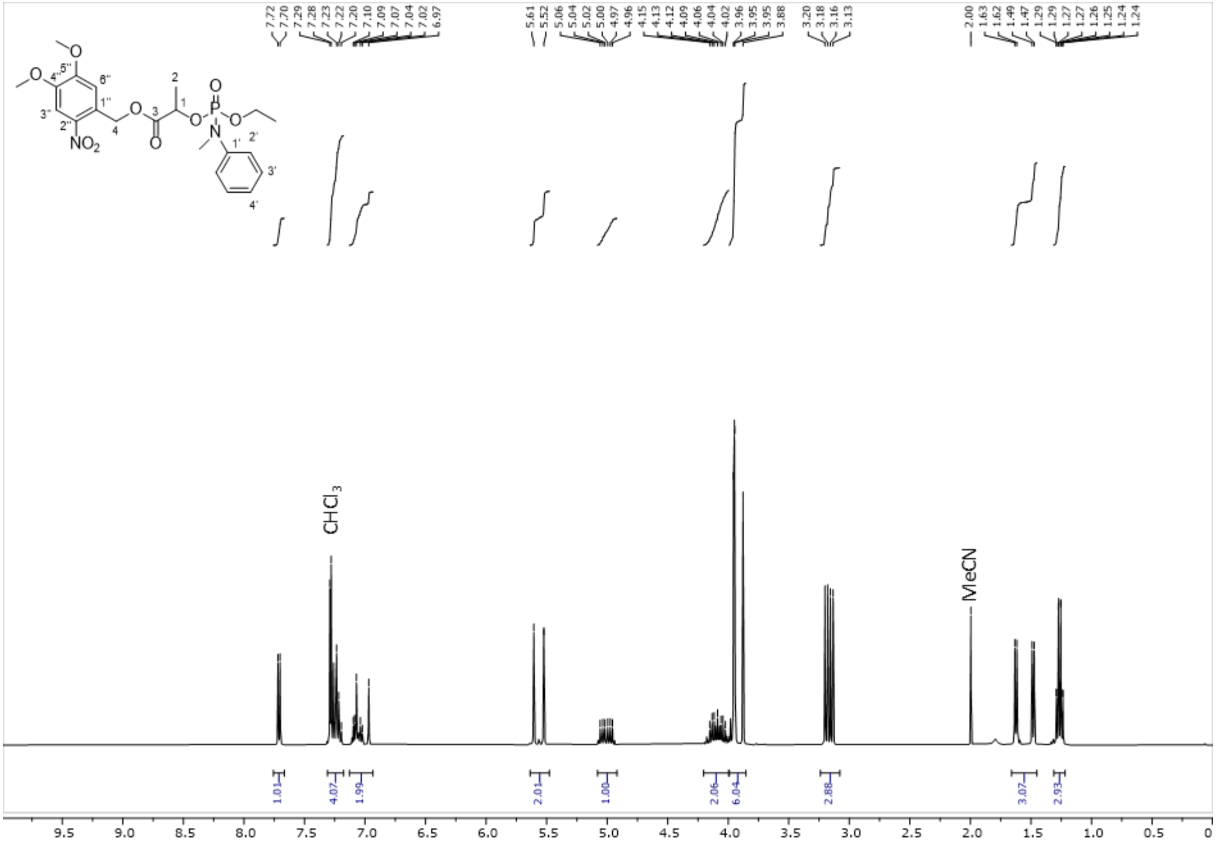

Figure S37. ^{1}H spectrum (ppm) of **10** in $CDCl_3$ measured at 25 °C.

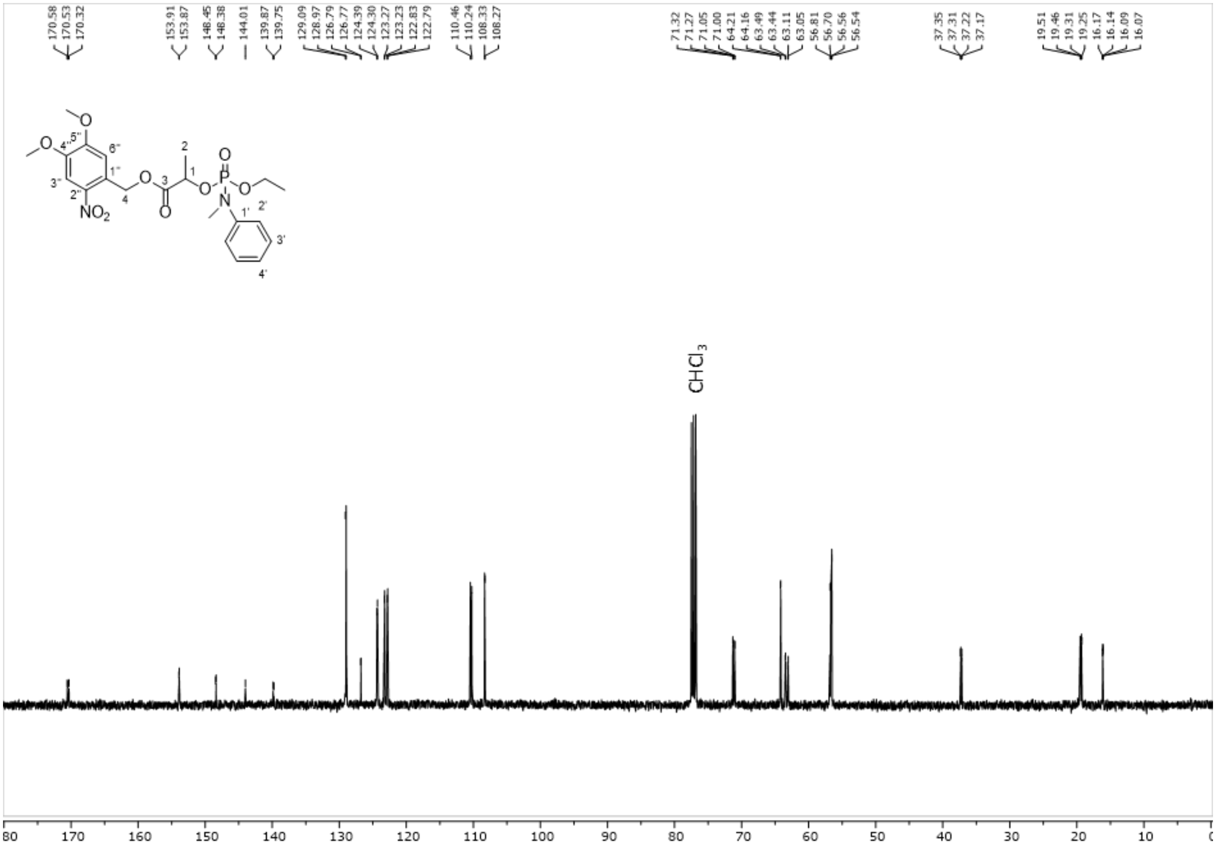

Figure S38. ^{13}C spectrum (ppm) of **10** in $CDCl_3$ measured at 25 °C.

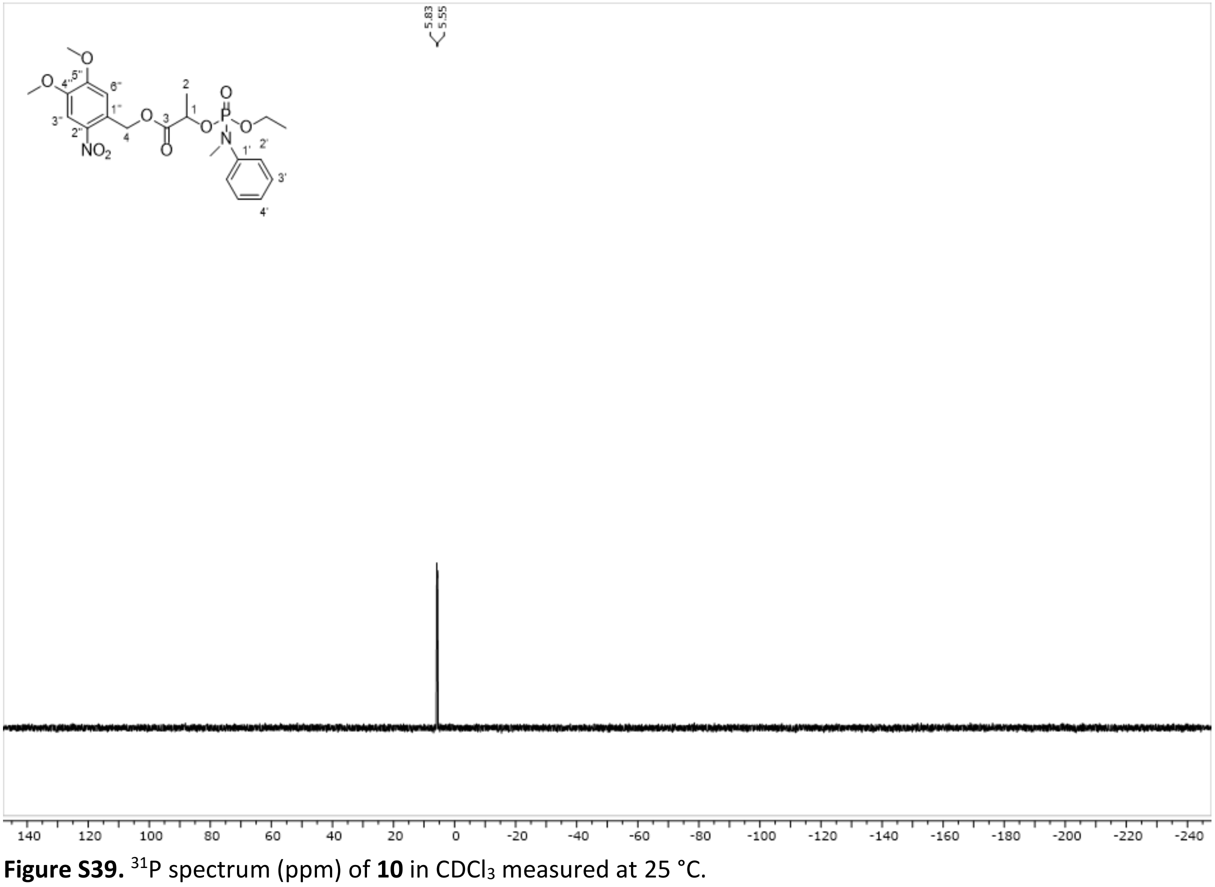

Figure S39. ^{31}P spectrum (ppm) of **10** in $CDCl_3$ measured at 25 °C.

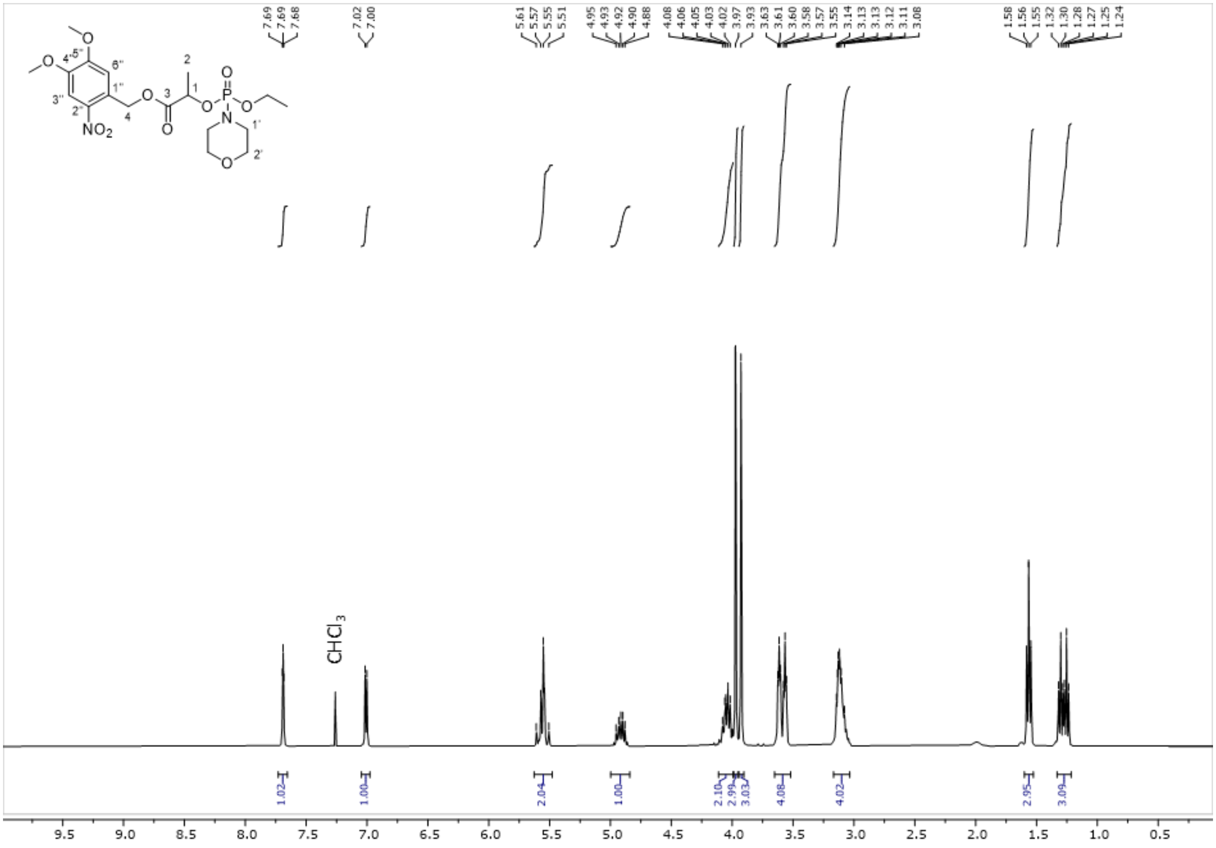

Figure S40. ^{1}H spectrum (ppm) of **11** in $CDCl_3$ measured at 25 °C.

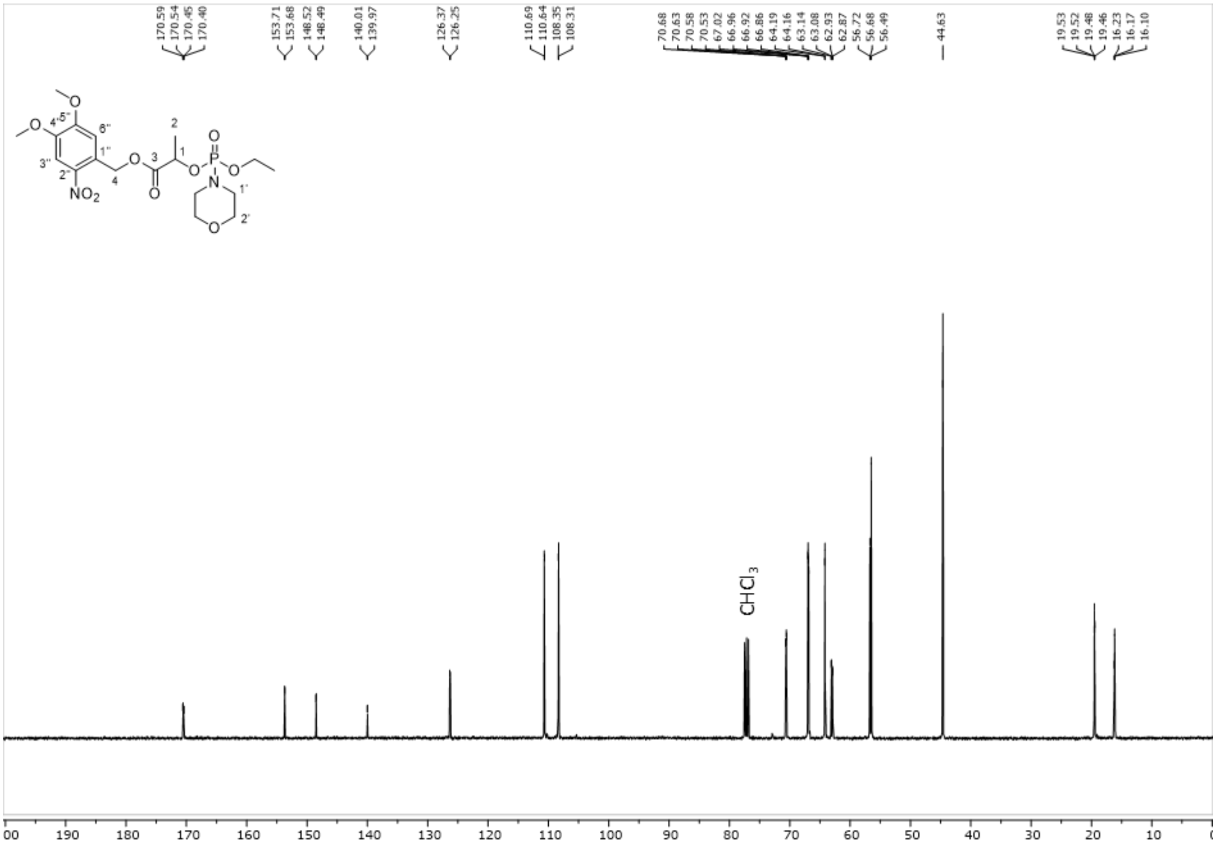

Figure S41. ^{13}C spectrum (ppm) of **11** in $CDCl_3$ measured at 25 °C.

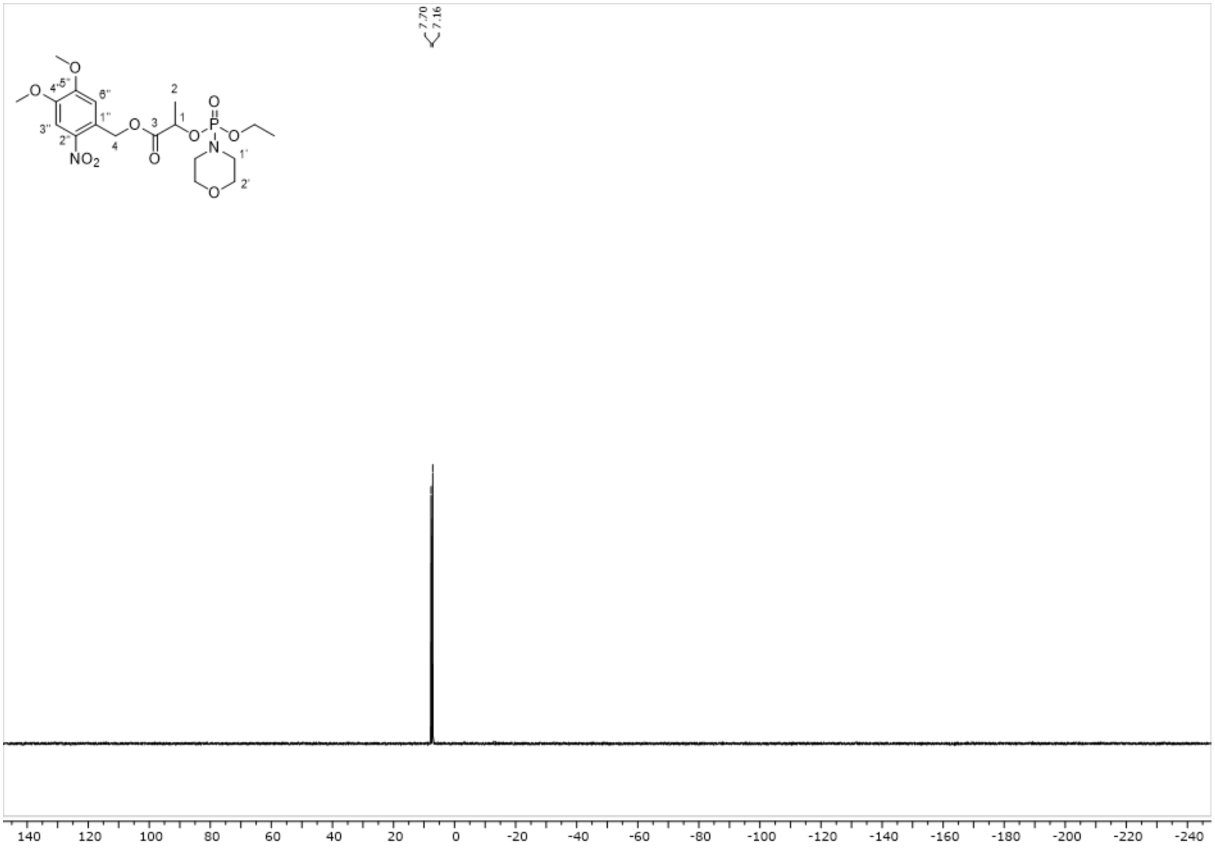

Figure S42. ^{31}P spectrum (ppm) of **11** in $CDCl_3$ measured at 25 °C.

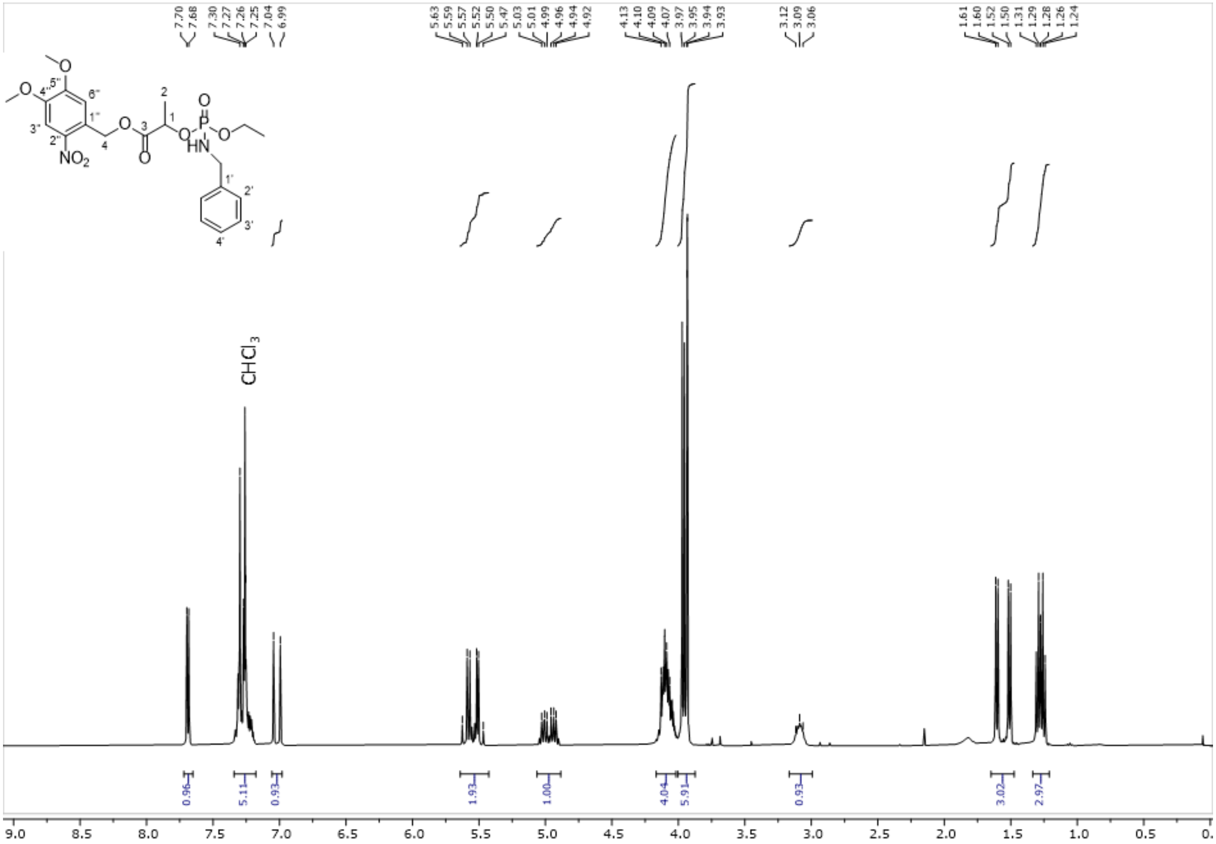

Figure S43. ^{1}H spectrum (ppm) of **12** in $CDCl_3$ measured at 25 °C.

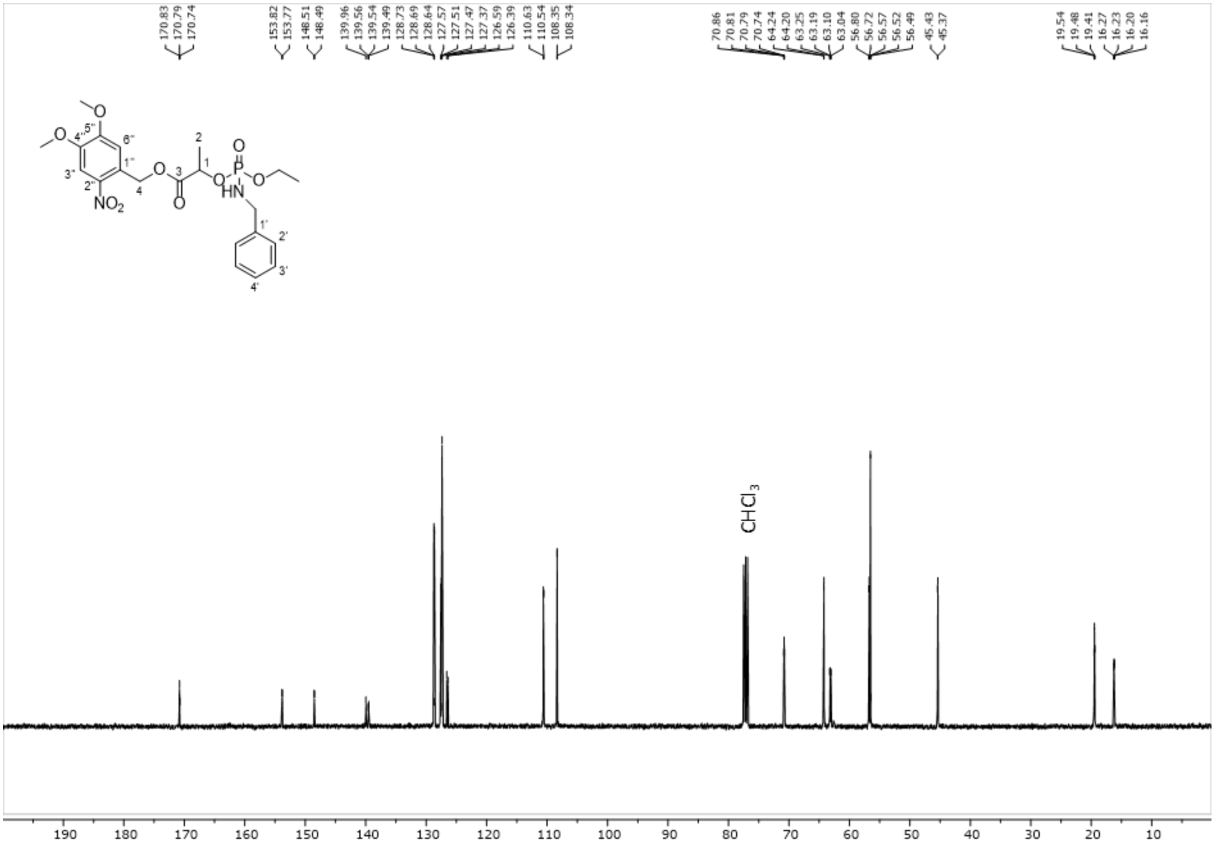

Figure S44. ^{13}C spectrum (ppm) of **12** in $CDCl_3$ measured at 25 °C.

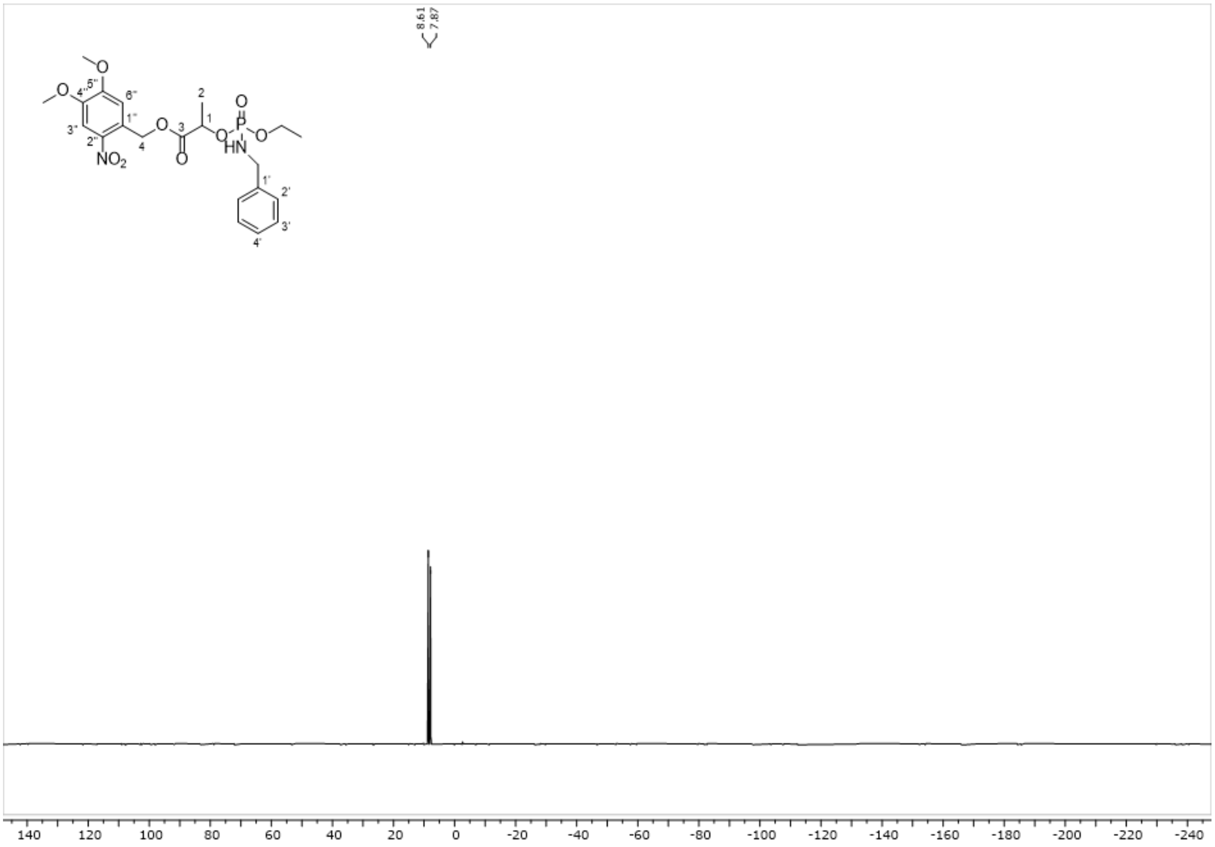

Figure S45. ^{31}P spectrum (ppm) of **12** in $CDCl_3$ measured at 25 °C.

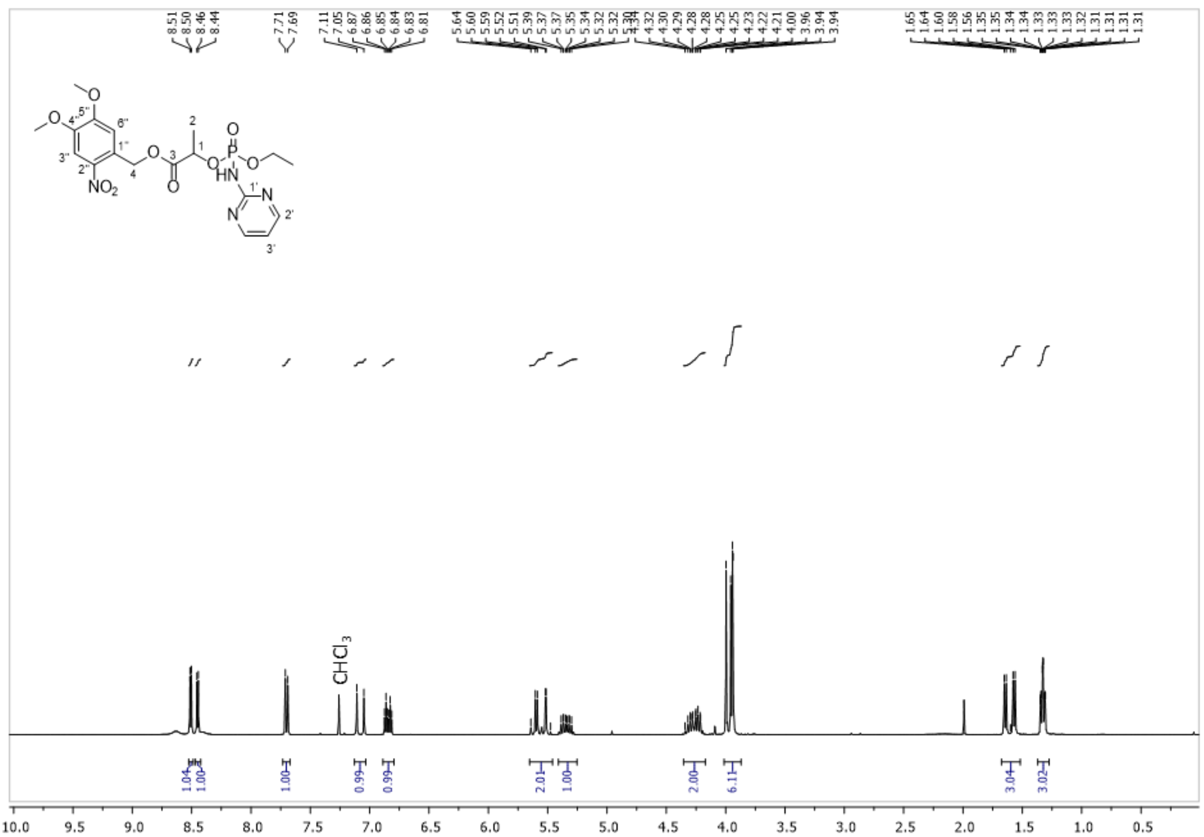

Figure S46. ^{1}H spectrum (ppm) of **13** in $CDCl_3$ measured at 25 °C.

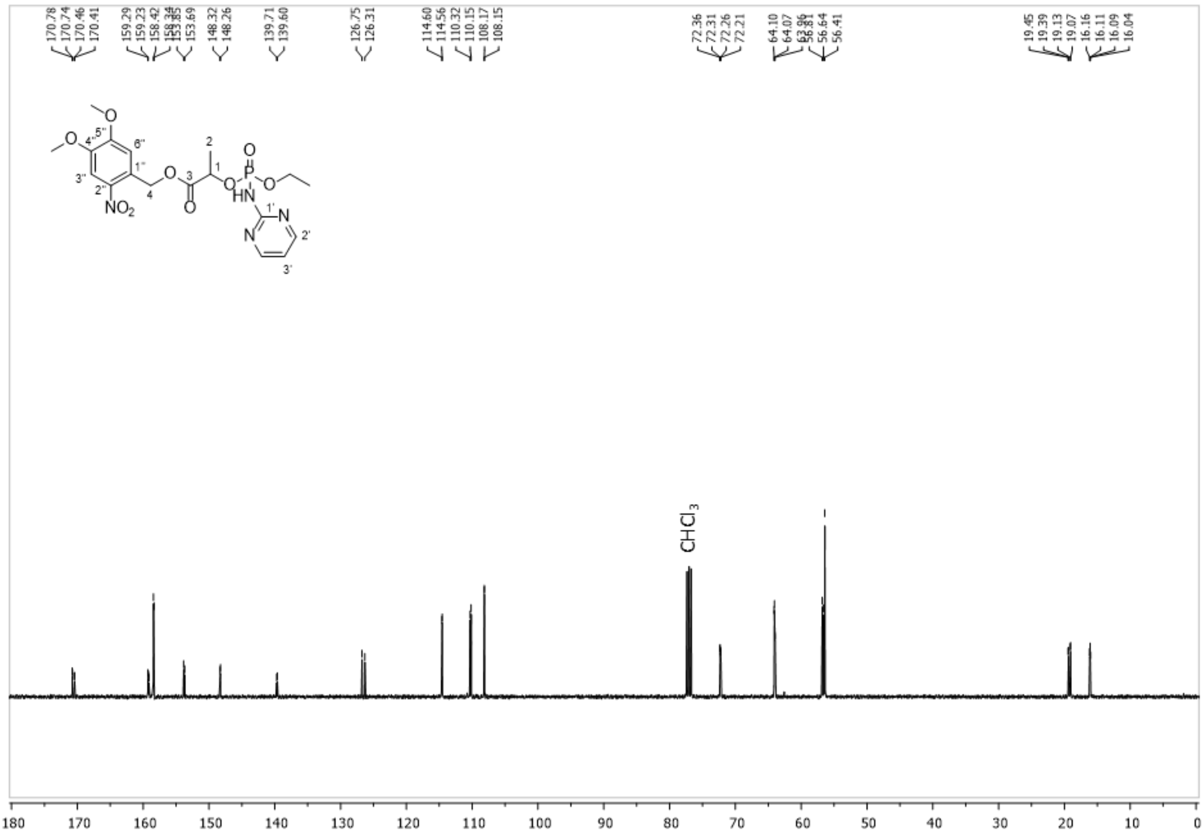

Figure S47. ^{13}C spectrum (ppm) of **13** in $CDCl_3$ measured at 25 °C.

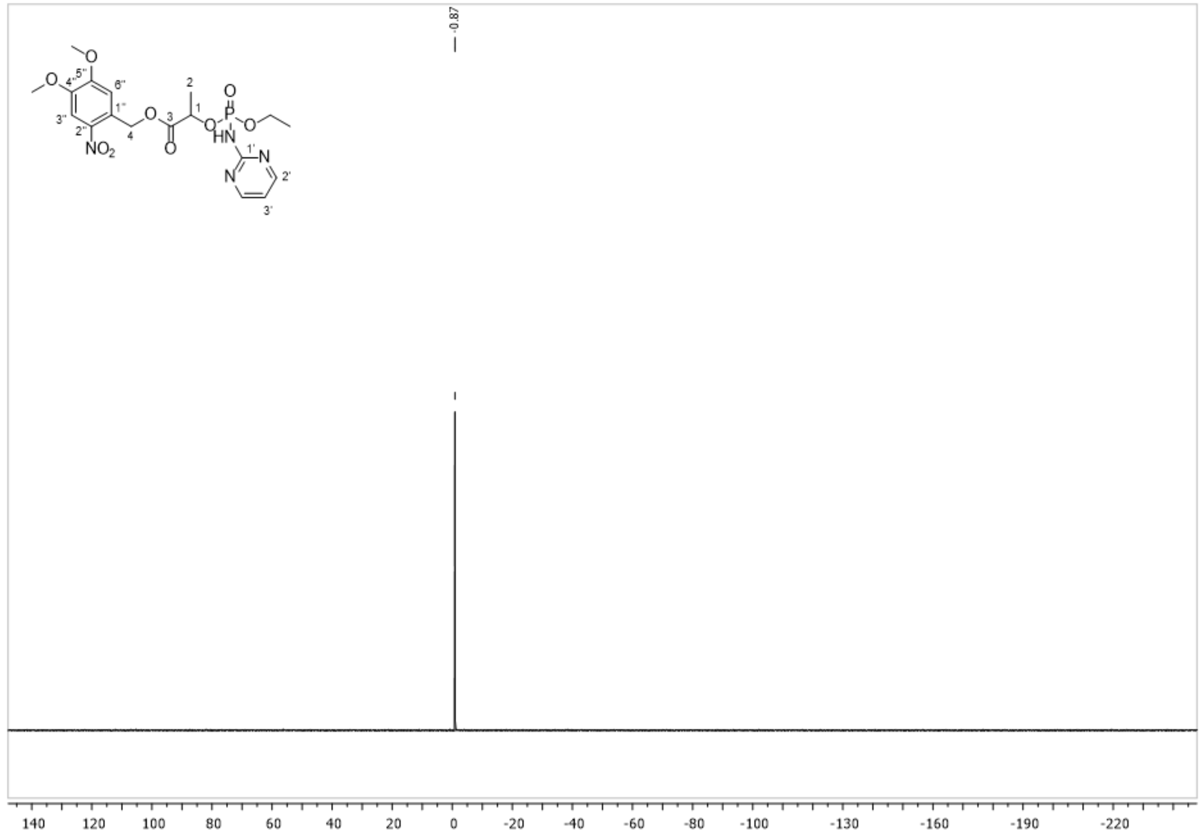

Figure S48. ^{31}P spectrum (ppm) of **13** in $CDCl_3$ measured at 25 °C.

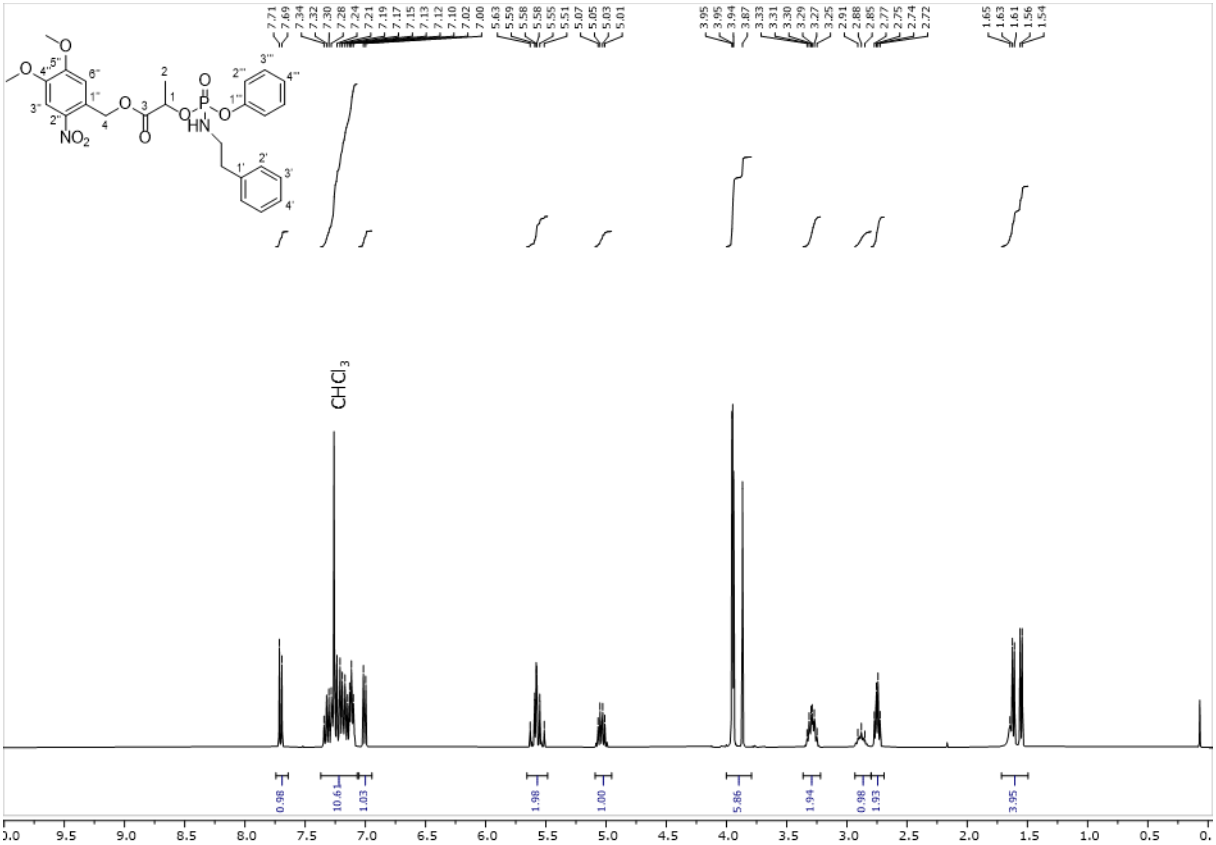

Figure S49. ^{1}H spectrum (ppm) of **14** in $CDCl_3$ measured at 25 °C.

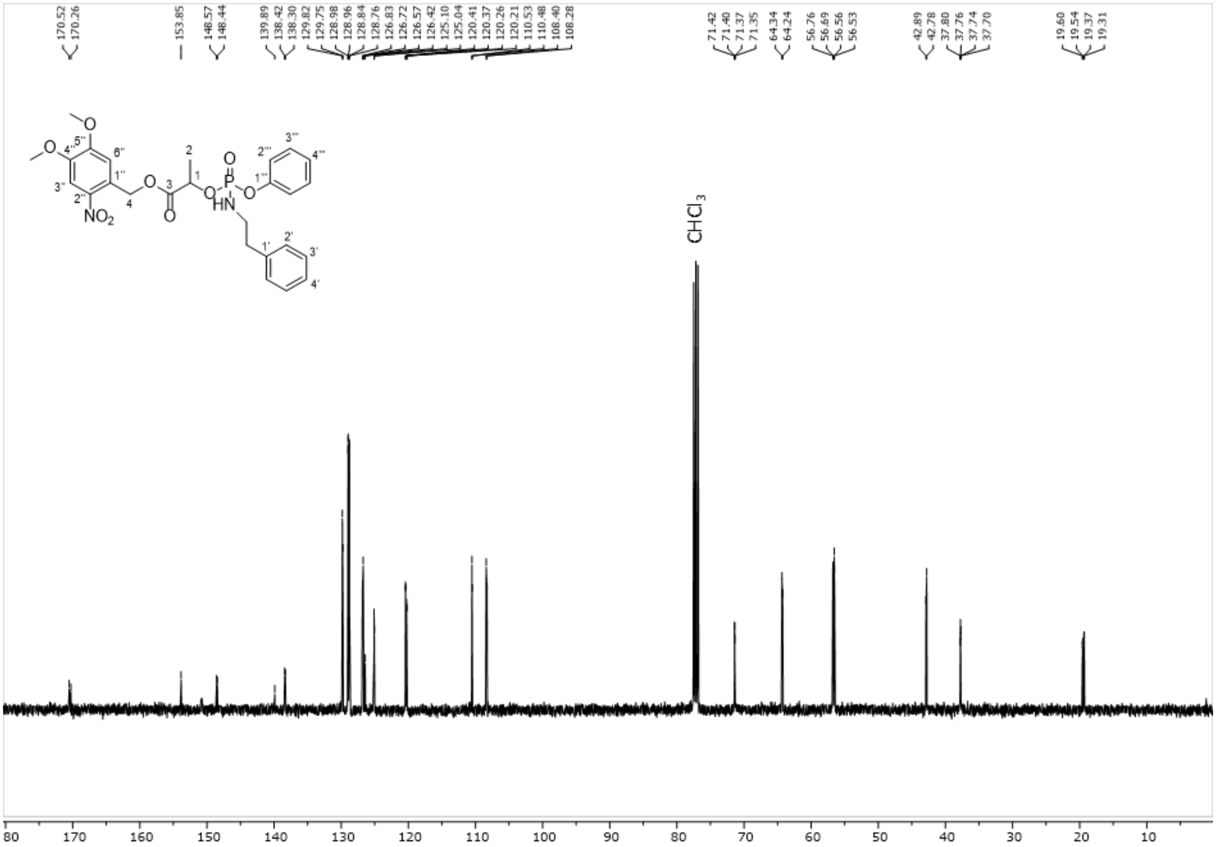

Figure S50. ^{13}C spectrum (ppm) of **14** in $CDCl_3$ measured at 25 °C.

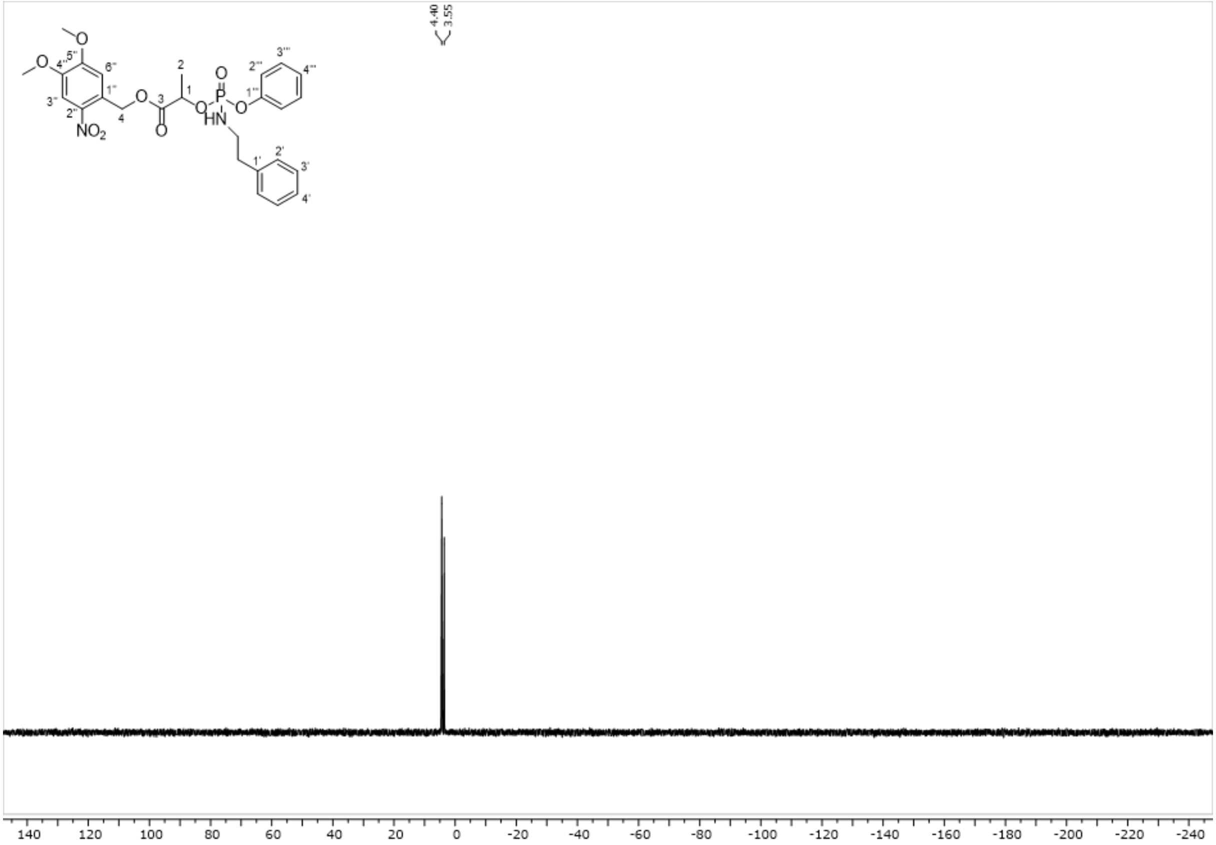

Figure S51. ^{31}P spectrum (ppm) of **14** in $CDCl_3$ measured at 25 °C.

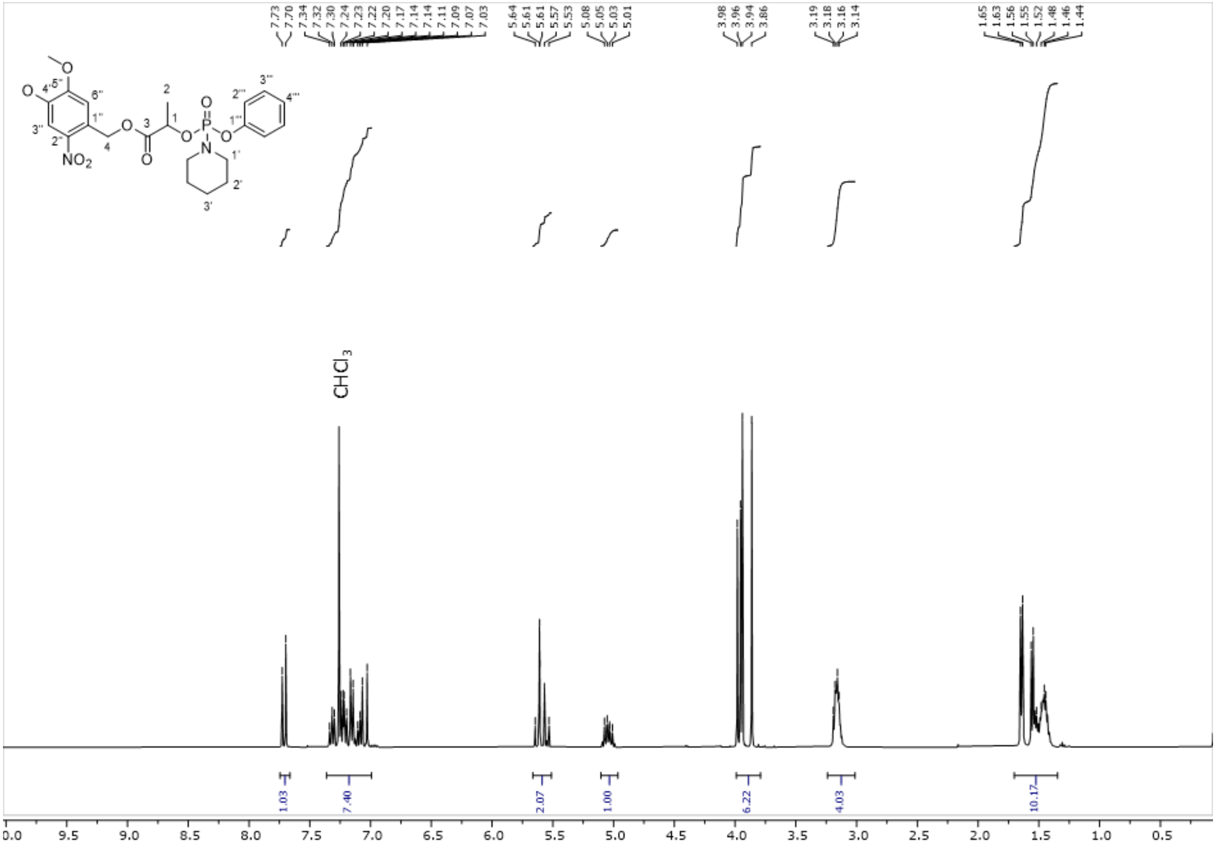

Figure S52. ^{1}H spectrum (ppm) of **15** in $CDCl_3$ measured at 25 °C.

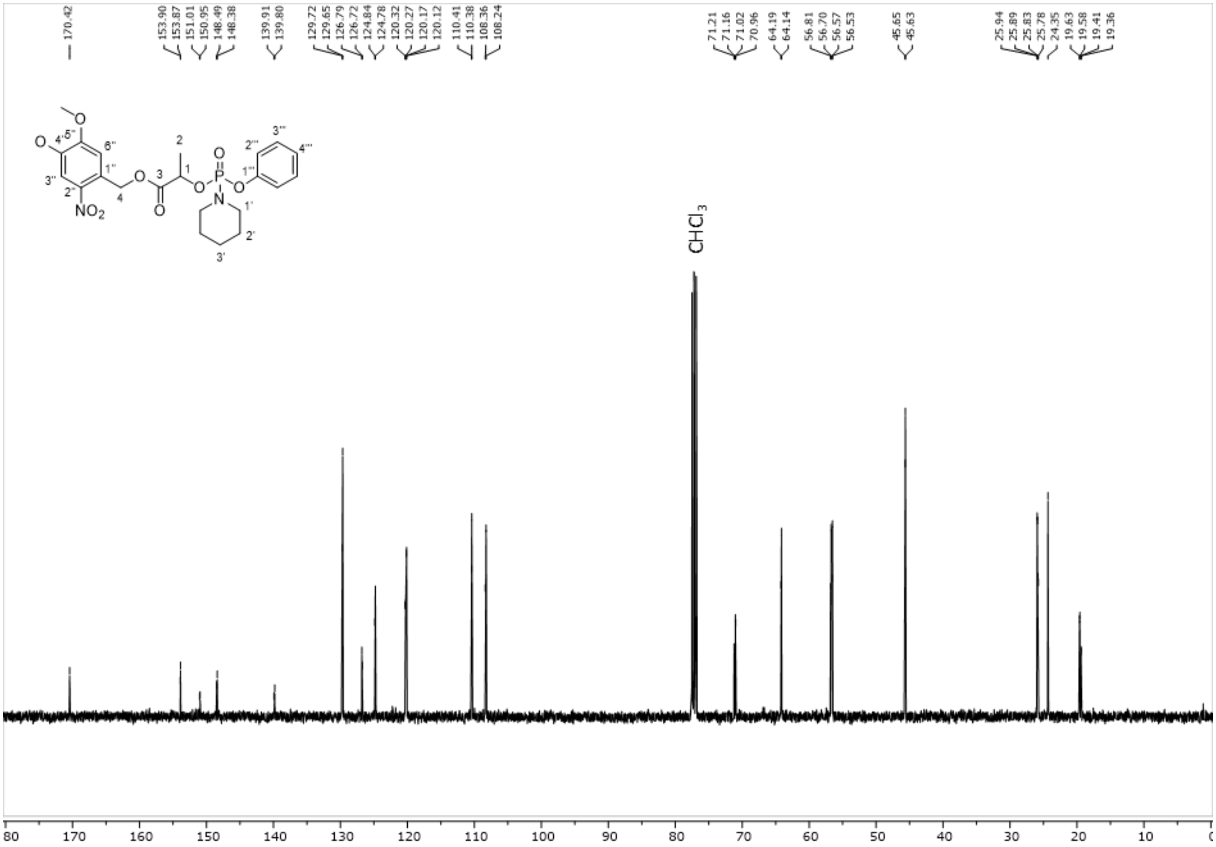

Figure S53. ^{13}C spectrum (ppm) of **15** in $CDCl_3$ measured at 25 °C.

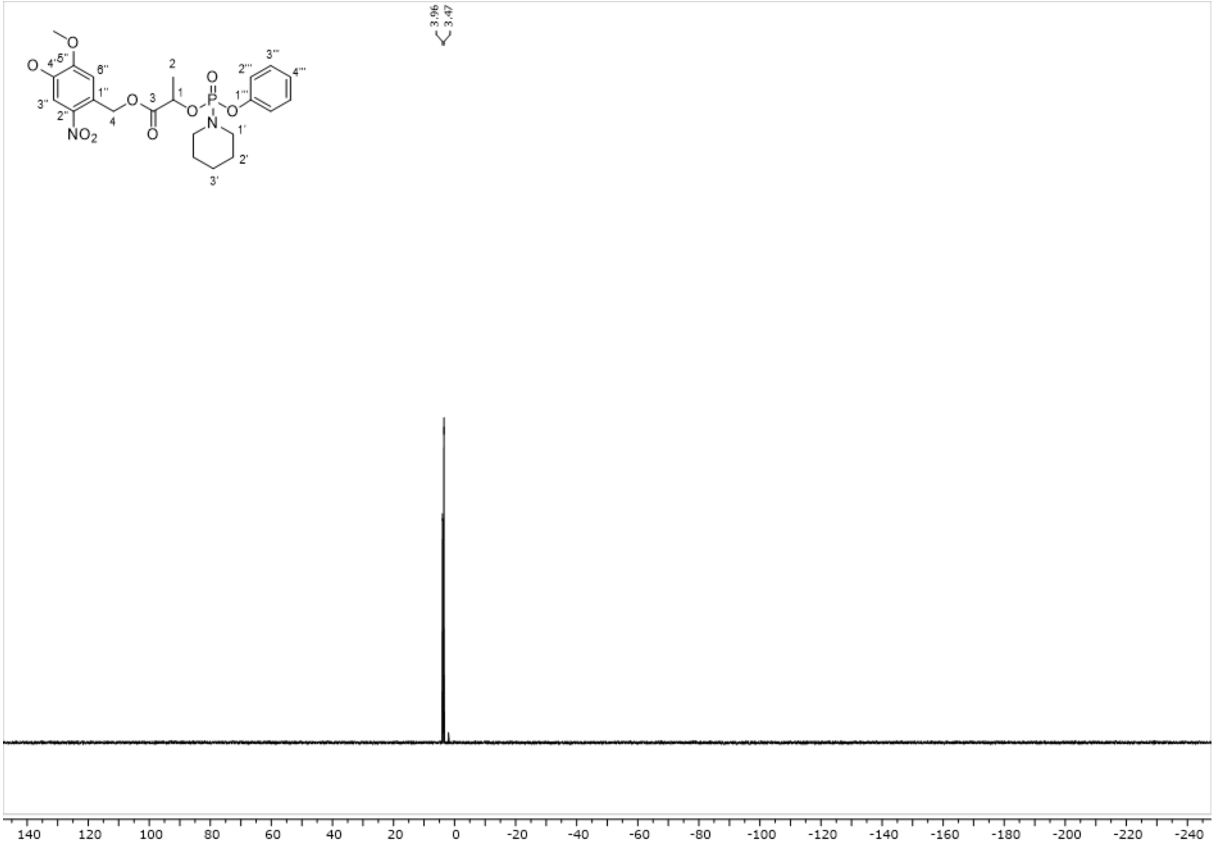

Figure S54. ^{31}P spectrum (ppm) of **15** in $CDCl_3$ measured at 25 °C.

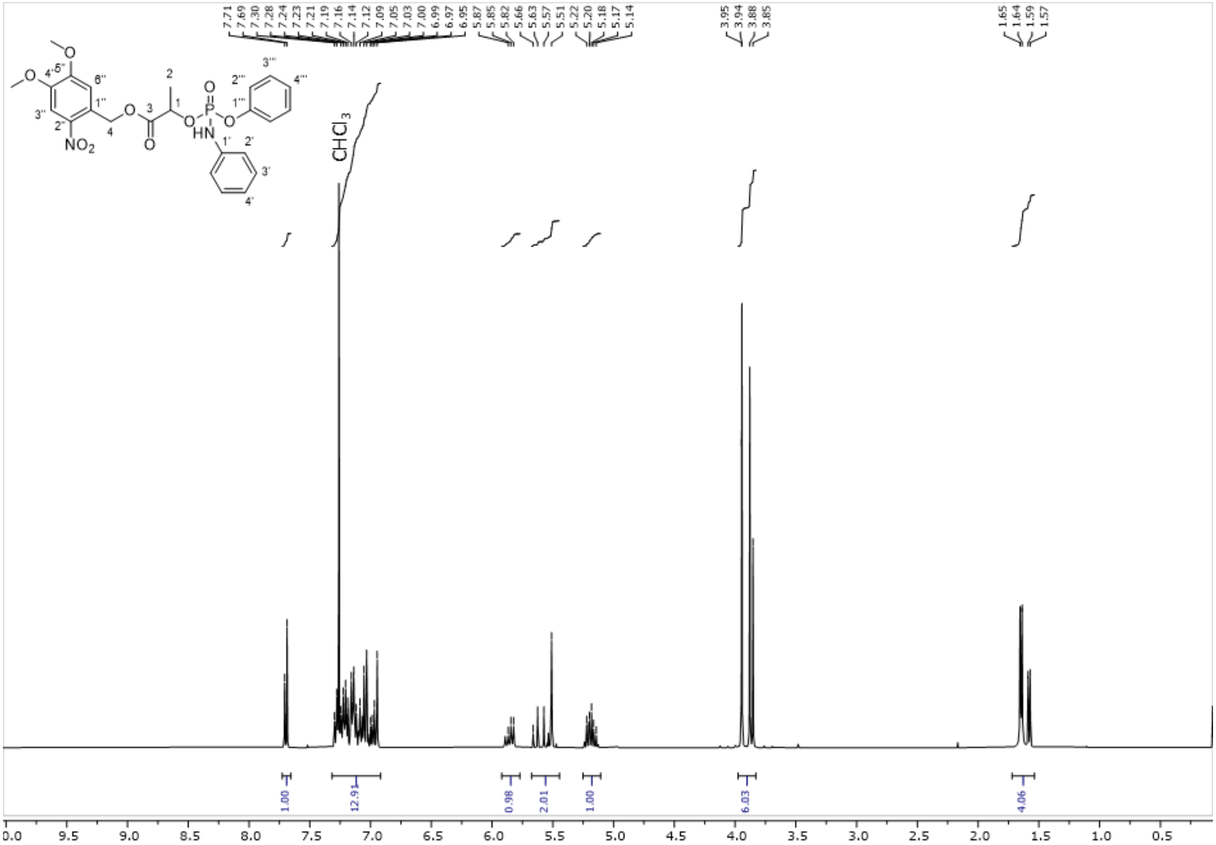

Figure S55. ^{1}H spectrum (ppm) of **16** in $CDCl_3$ measured at 25 °C.

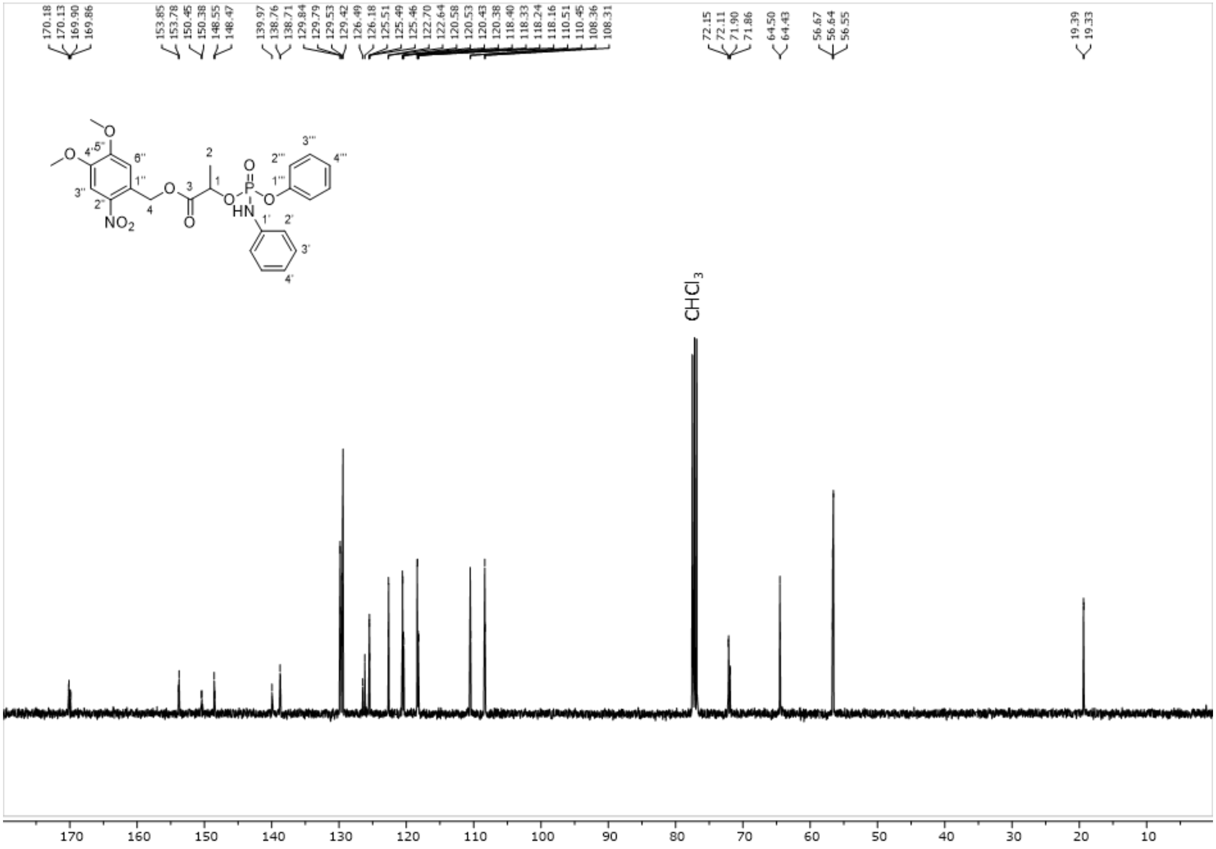

Figure S56. ^{13}C spectrum (ppm) of **16** in $CDCl_3$ measured at 25 °C.

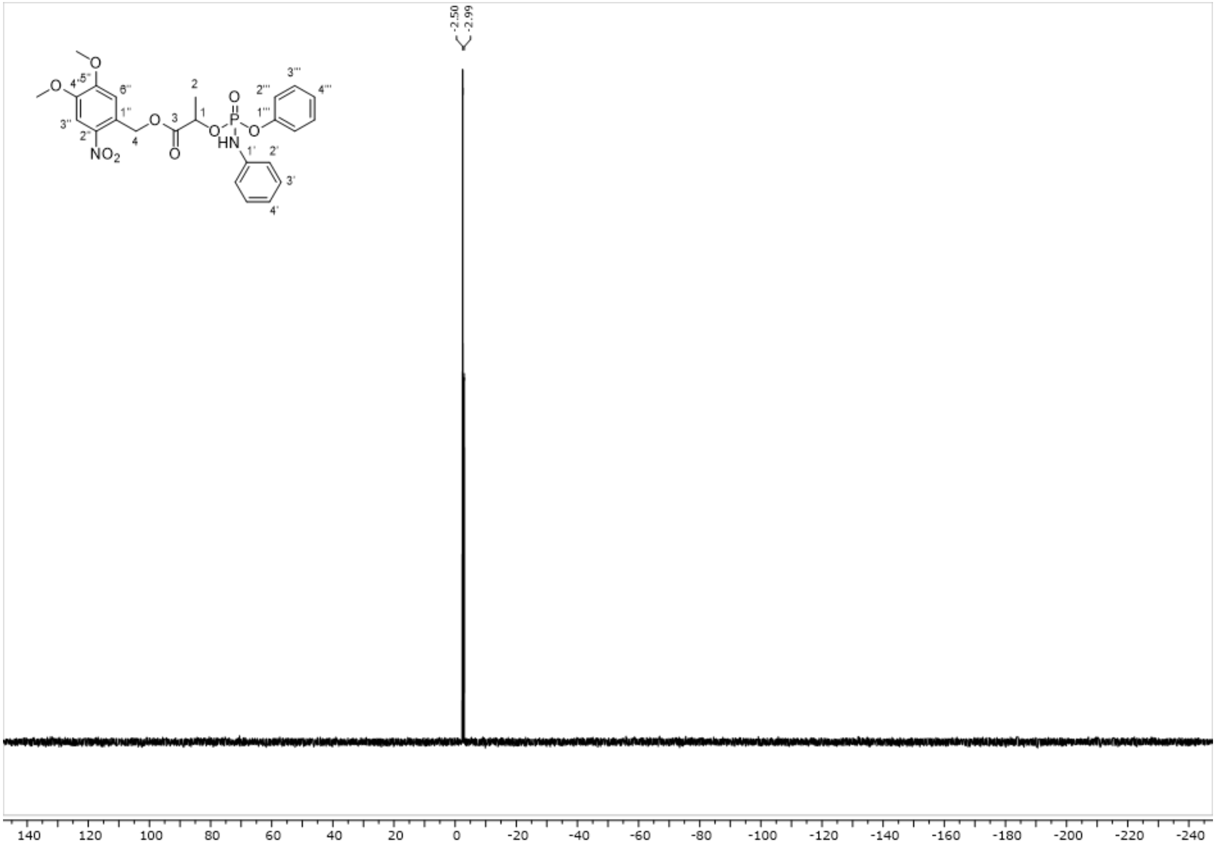

Figure S57. ^{31}P spectrum (ppm) of **16** in $CDCl_3$ measured at 25 °C.

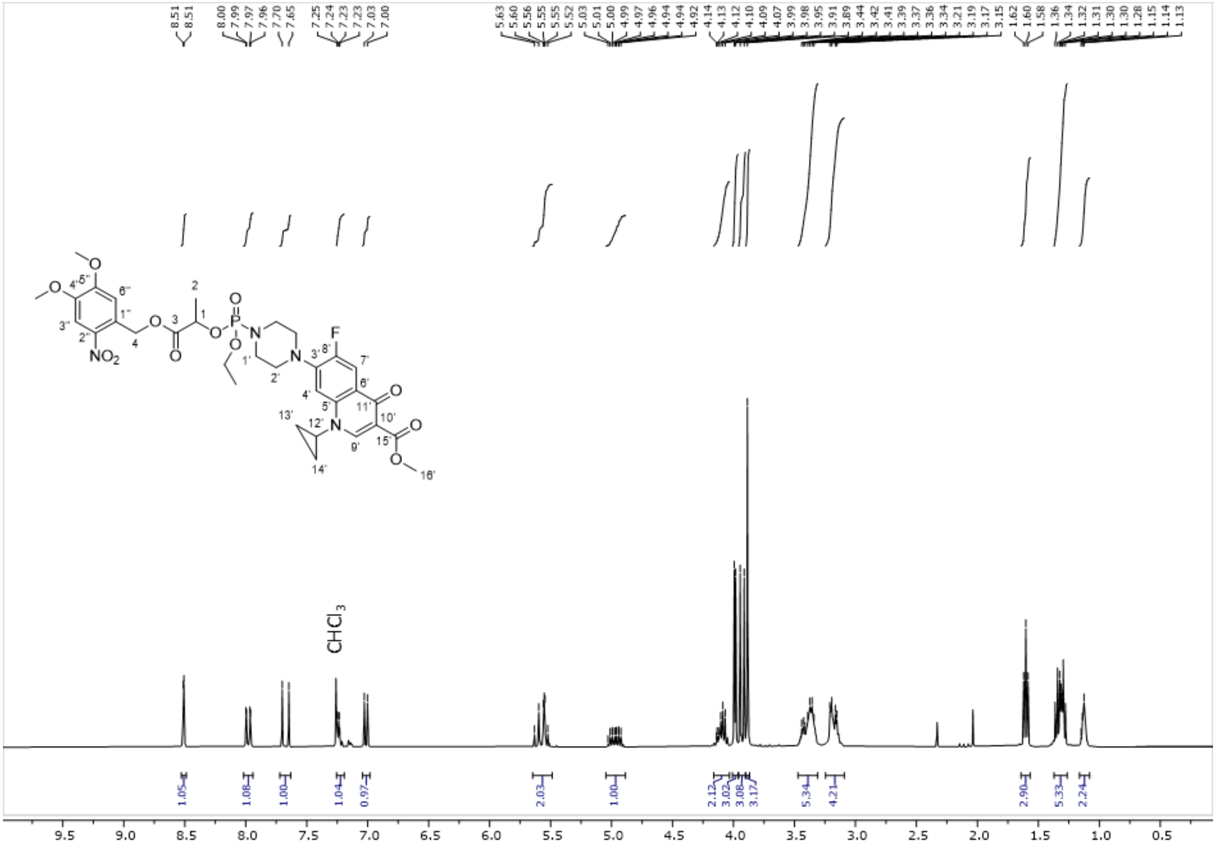

Figure S58. ^{1}H spectrum (ppm) of **17** in $CDCl_3$ measured at 25 °C.

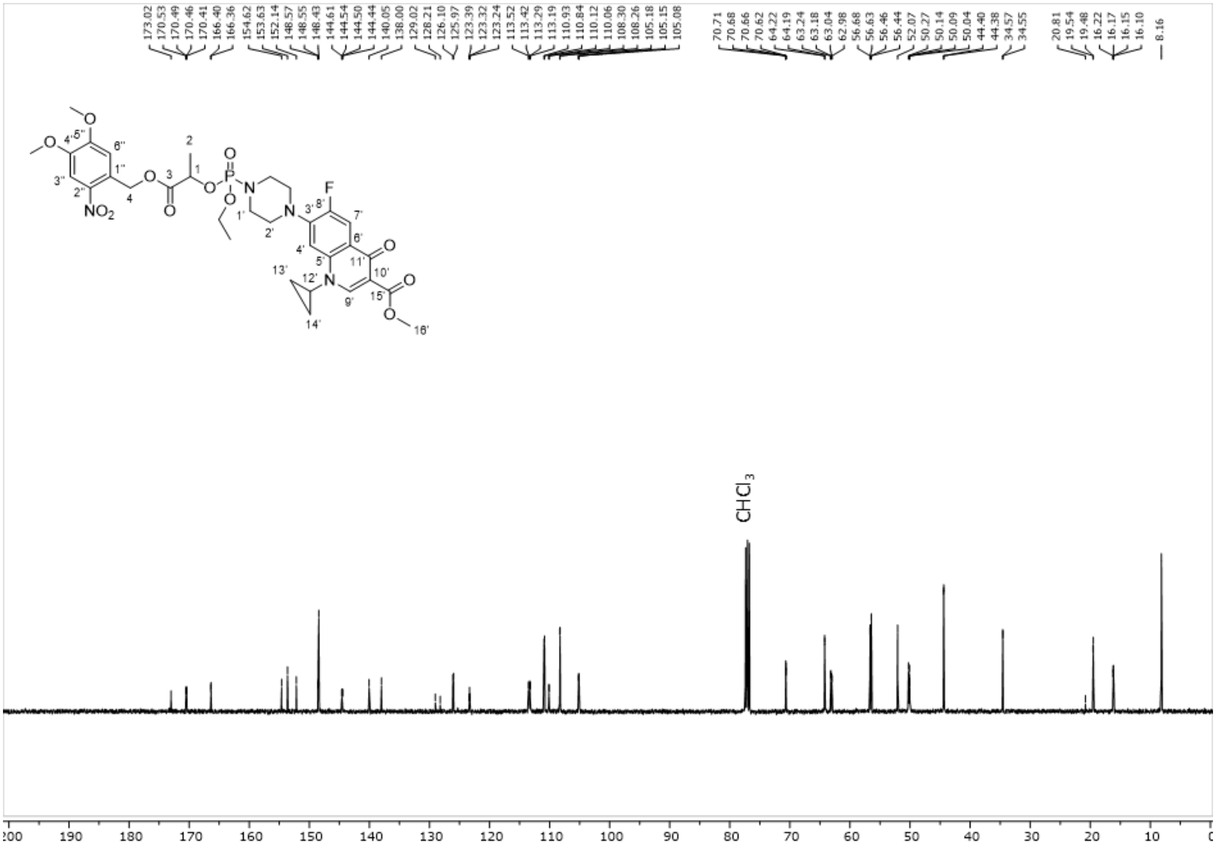

Figure S59. ^{13}C spectrum (ppm) of **17** in $CDCl_3$ measured at 25 °C.

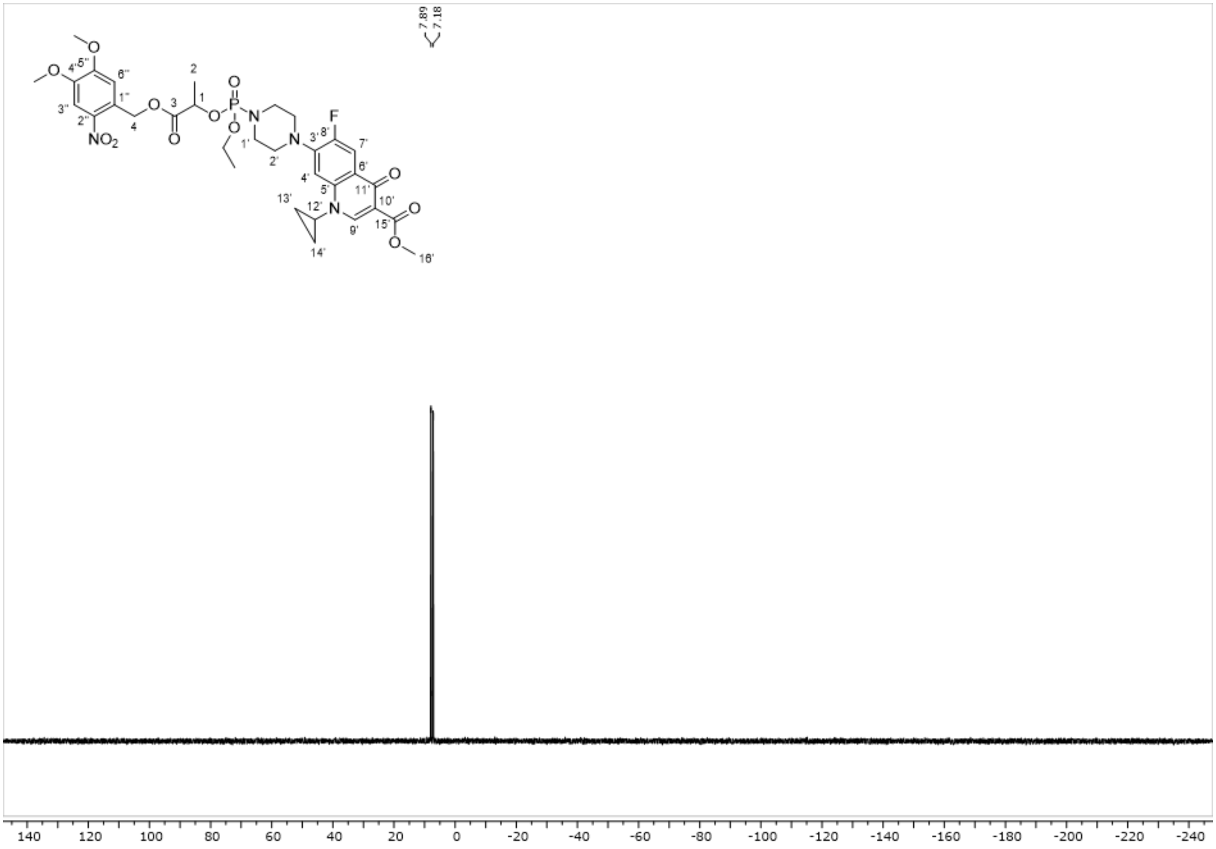

Figure S60. ^{31}P spectrum (ppm) of **17** in $CDCl_3$ measured at 25 °C.

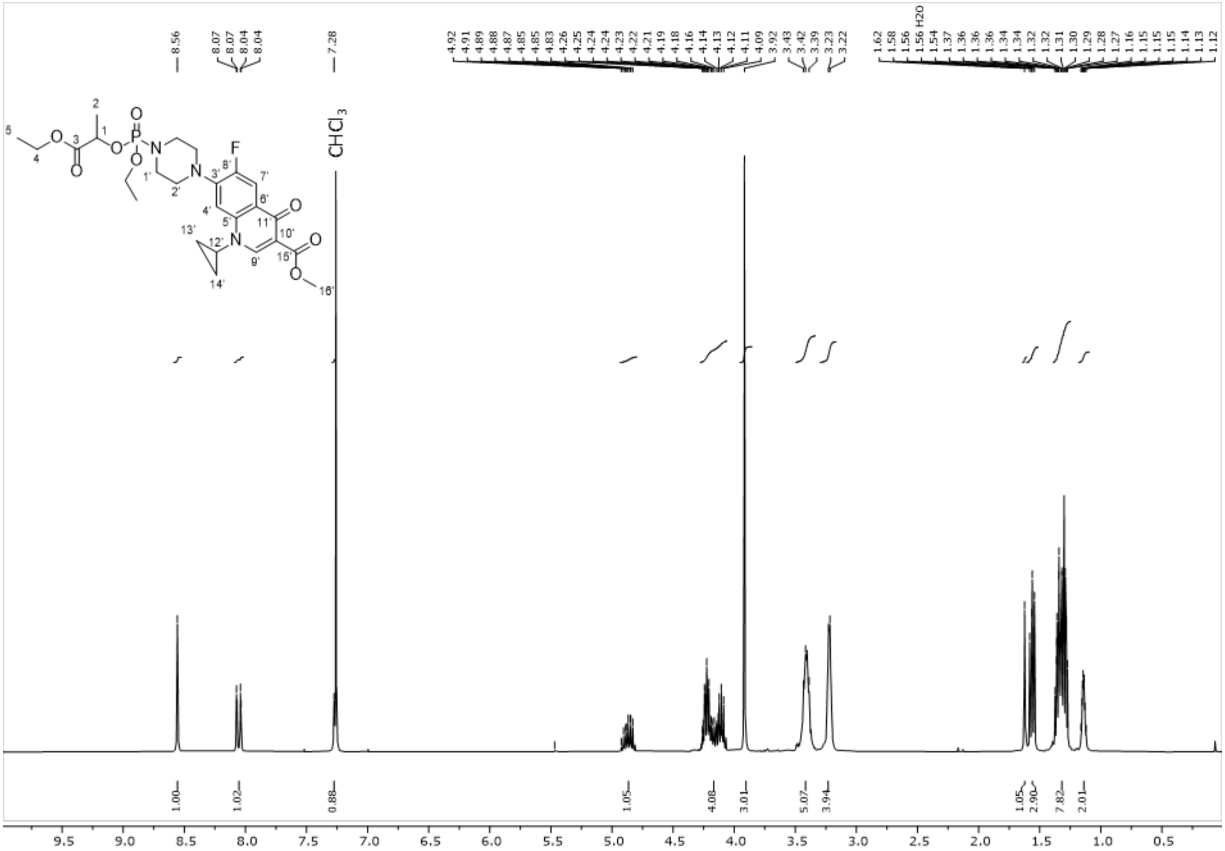

Figure S61. ^{1}H spectrum (ppm) of **18** in $CDCl_3$ measured at 25 °C.

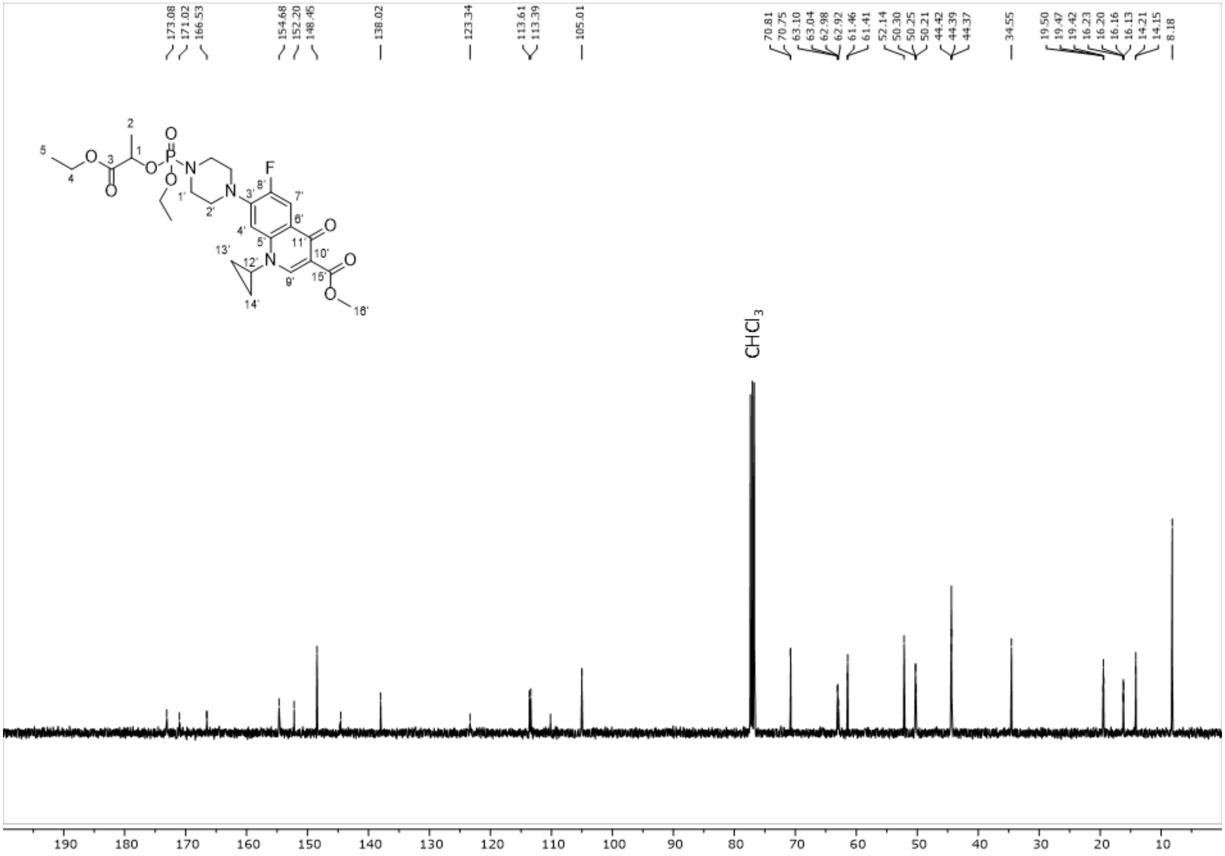

Figure S62. ^{13}C spectrum (ppm) of **18** in $CDCl_3$ measured at 25 °C.

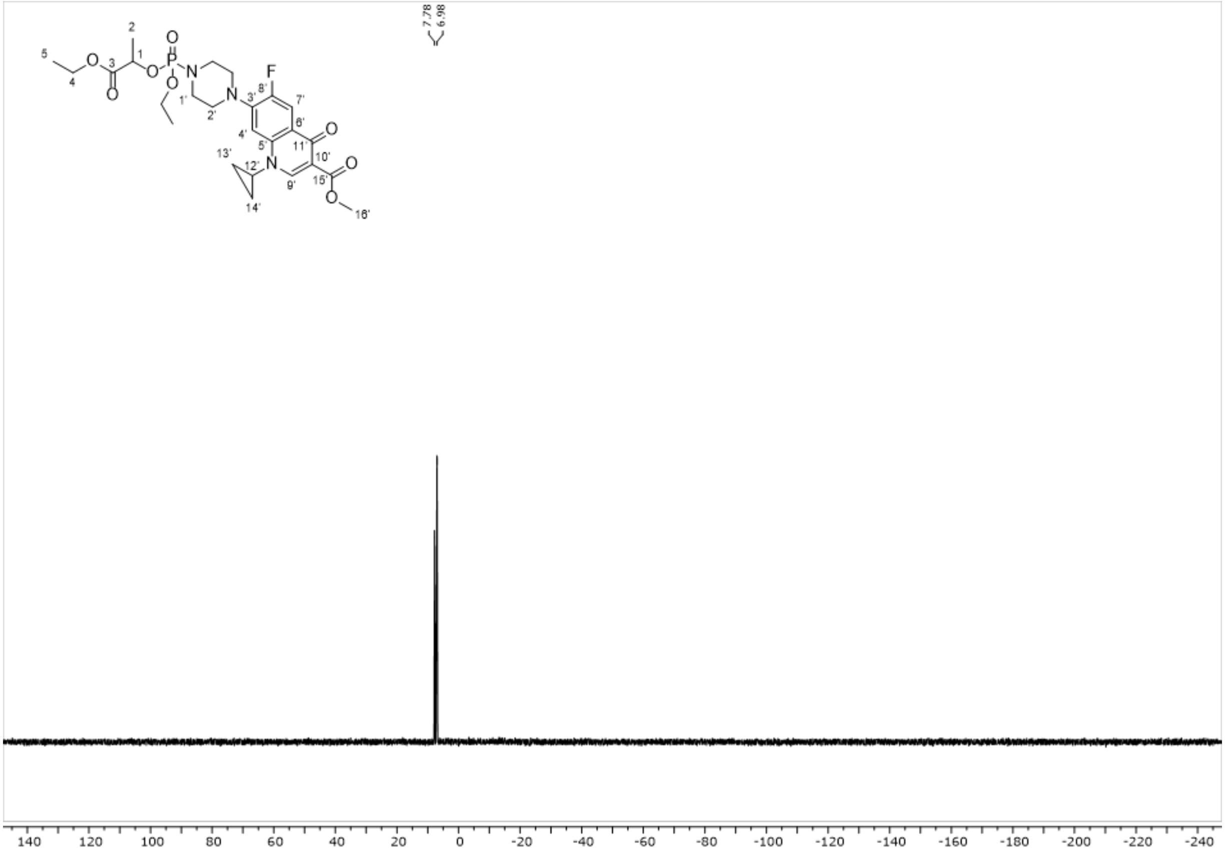

Figure S63. ^{31}P spectrum (ppm) of **18** in $CDCl_3$ measured at 25 °C.

4 Compound characterization: MS spectra

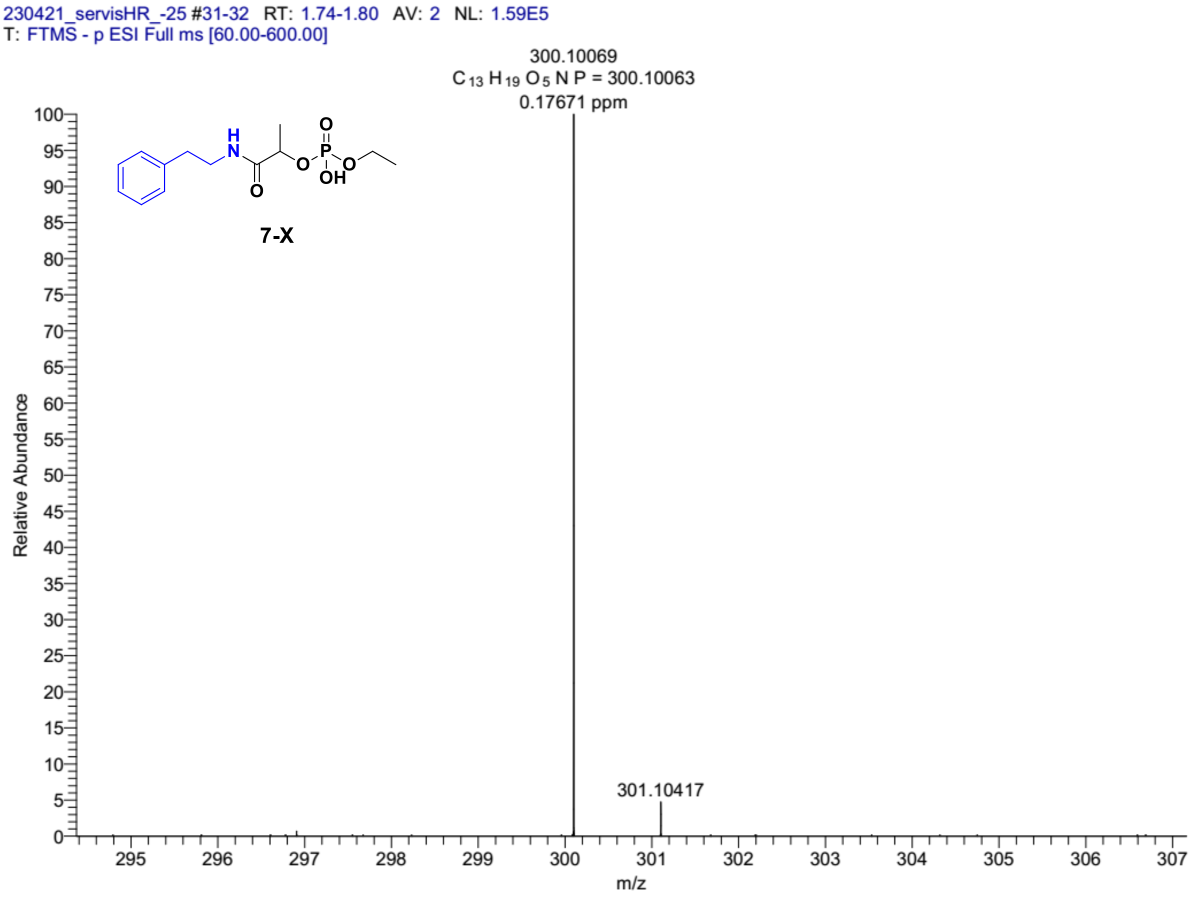

Figure S64. HR-MS spectrum of **7** measured in CACO (pH = 7.4)/DMSO-d_6, 25 °C after irradiation by UV light (365 nm) clearly confirms mass of the **7-X**. Molecular ion in the negative mode (M^-) corresponds to the calculated for the carboxamide **7-X**, formed by intramolecular rearrangement under basic conditions.

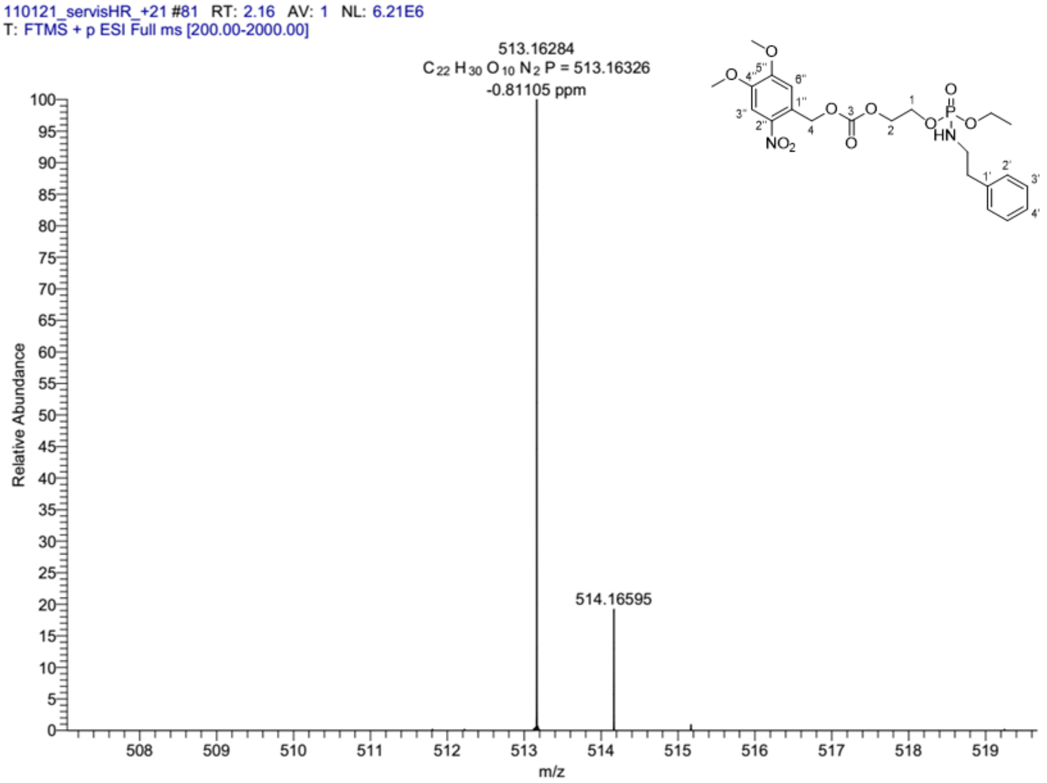

Figure S65. HR-MS spectrum of compound **1**.

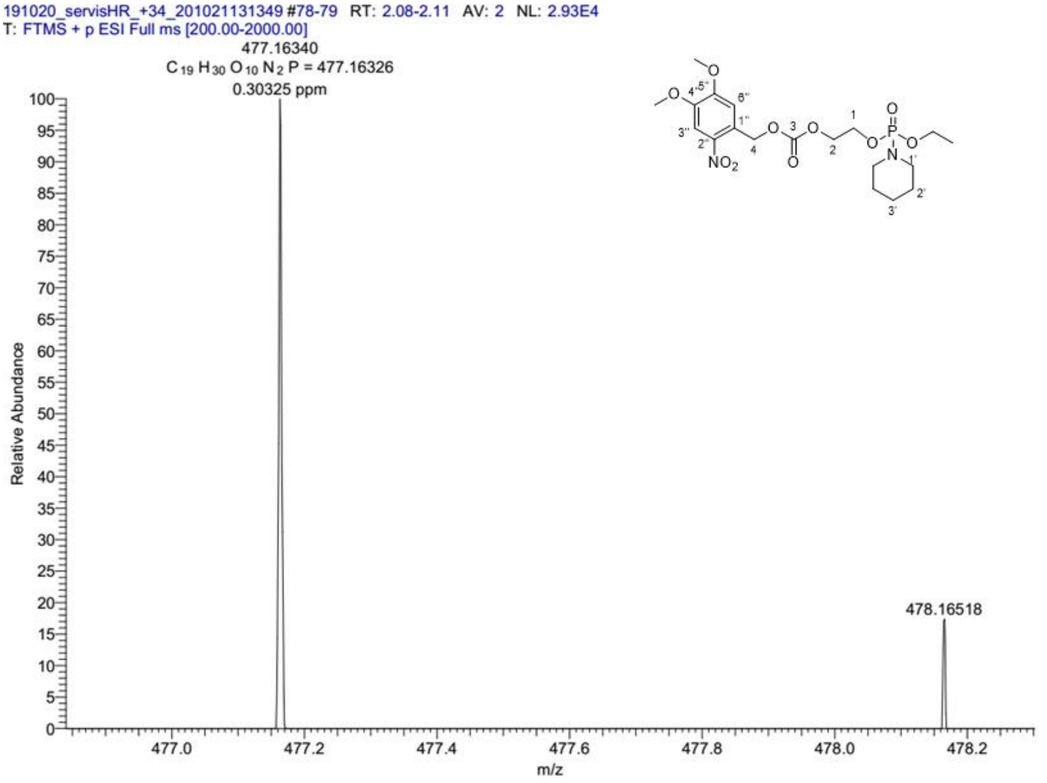

Figure S66. HR-MS spectrum of compound **2**.

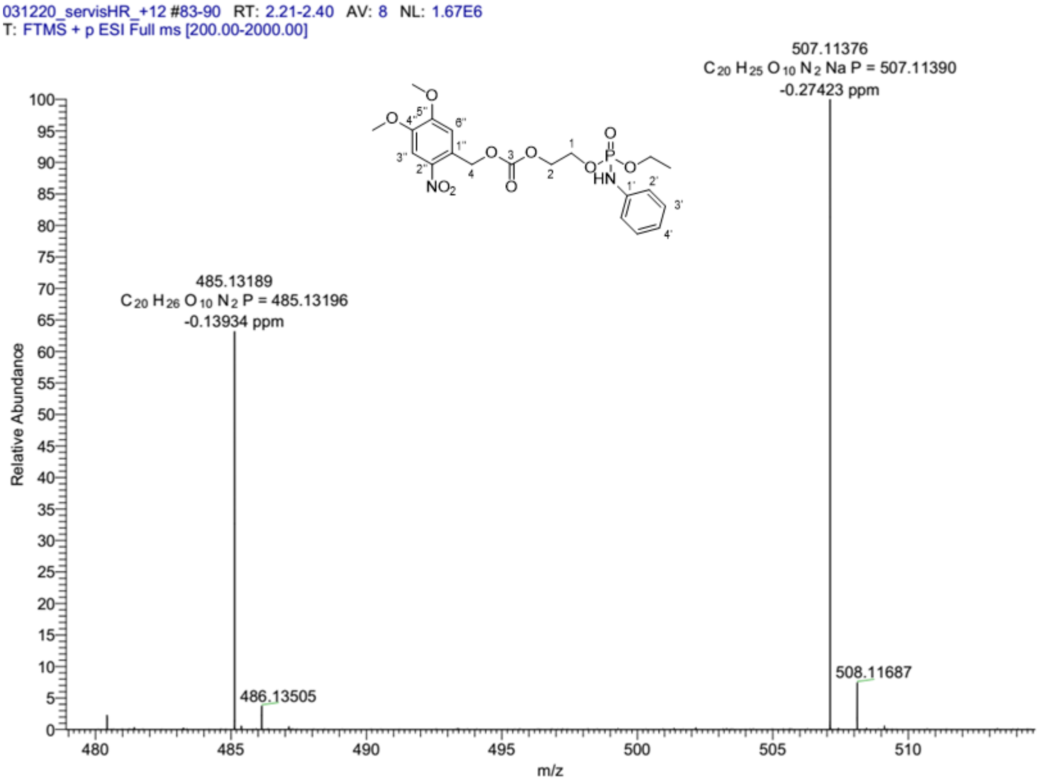

Figure S67. HR-MS spectrum of compound **3**.

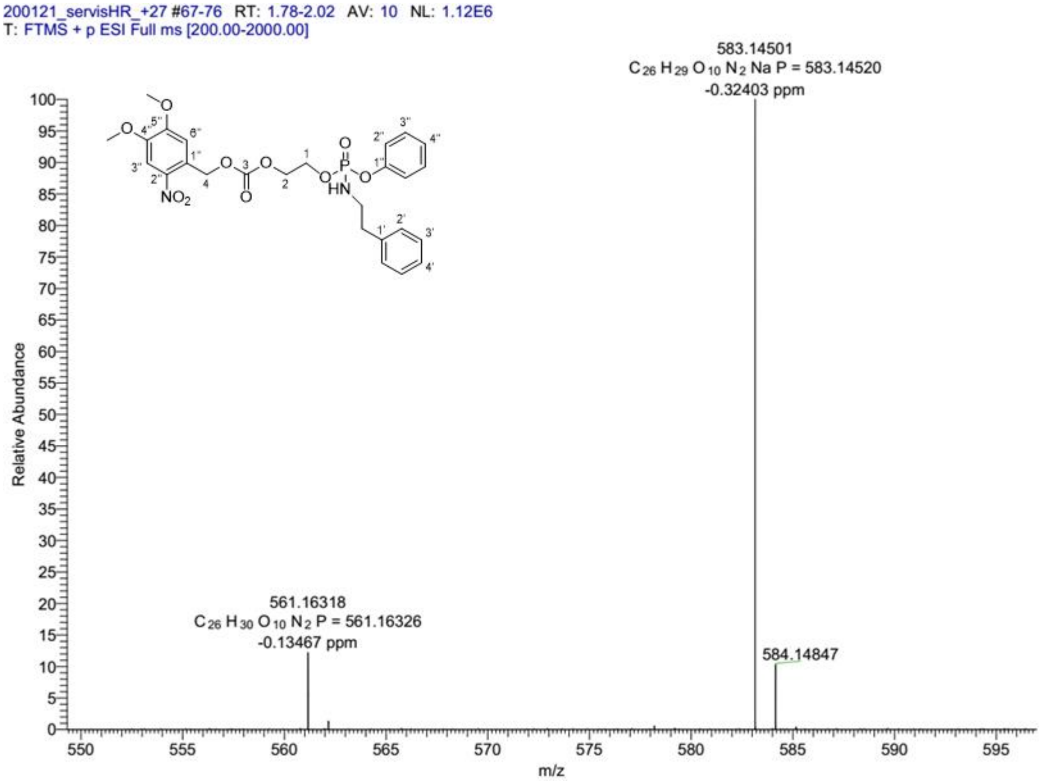

Figure S68. HR-MS spectrum of compound **4**.

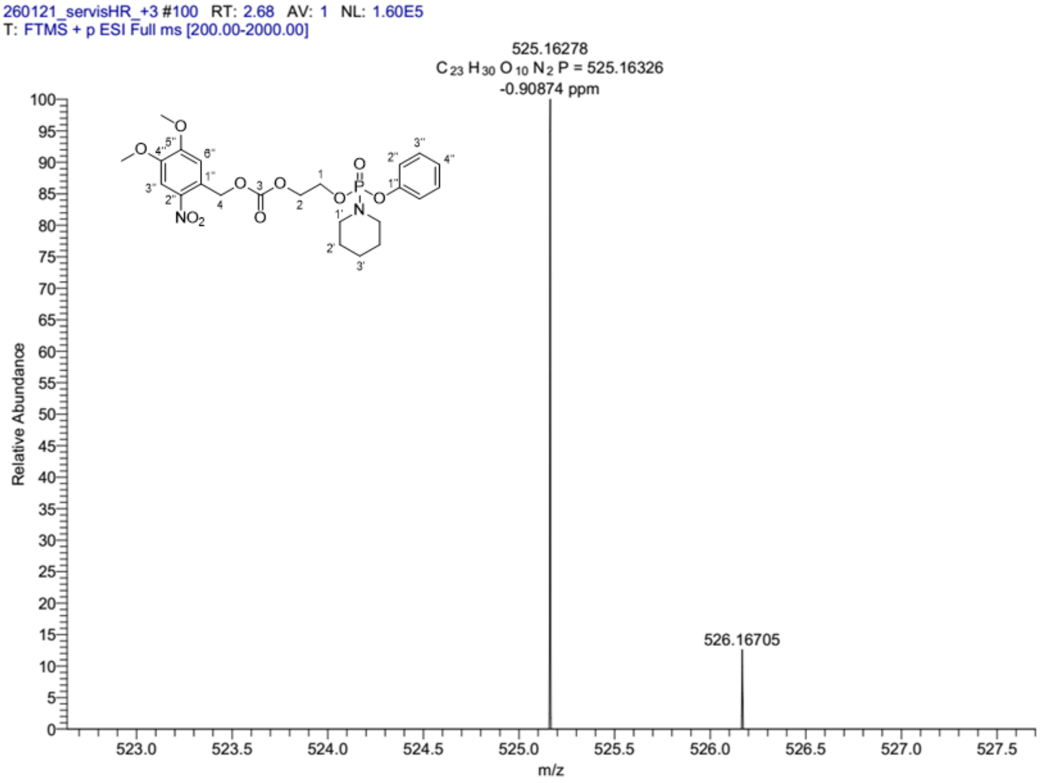

Figure S69. HR-MS spectrum of compound **5**.

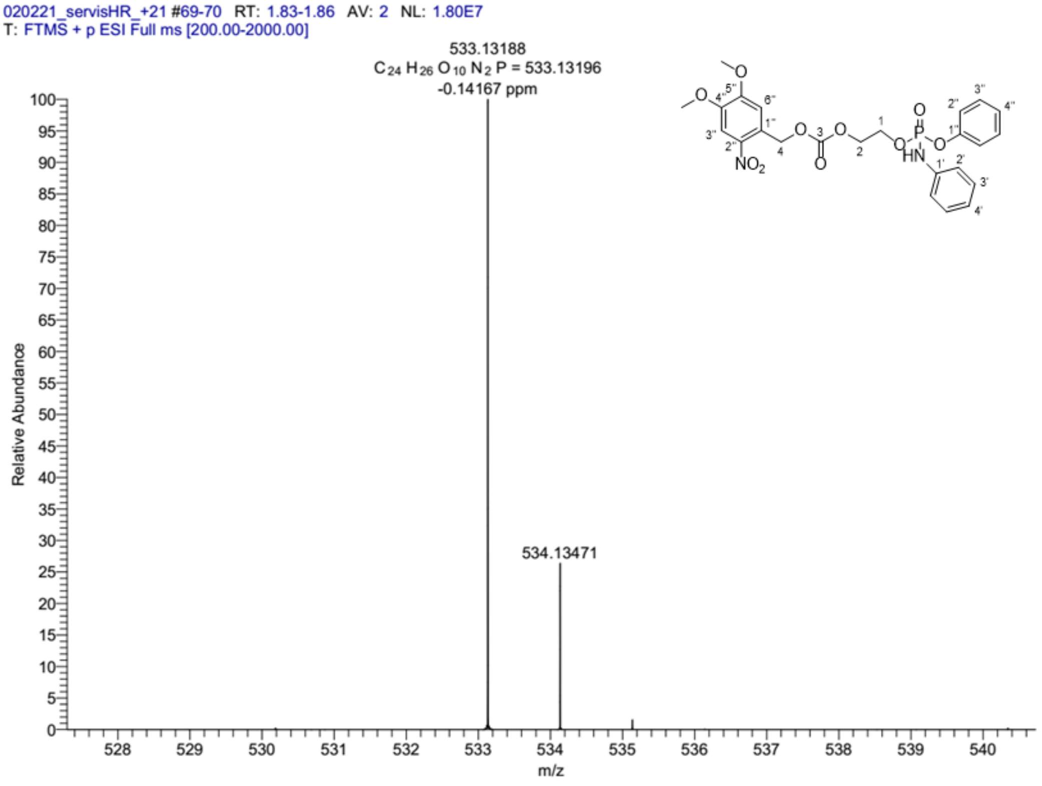

Figure S70. HR-MS spectrum of compound **6**.

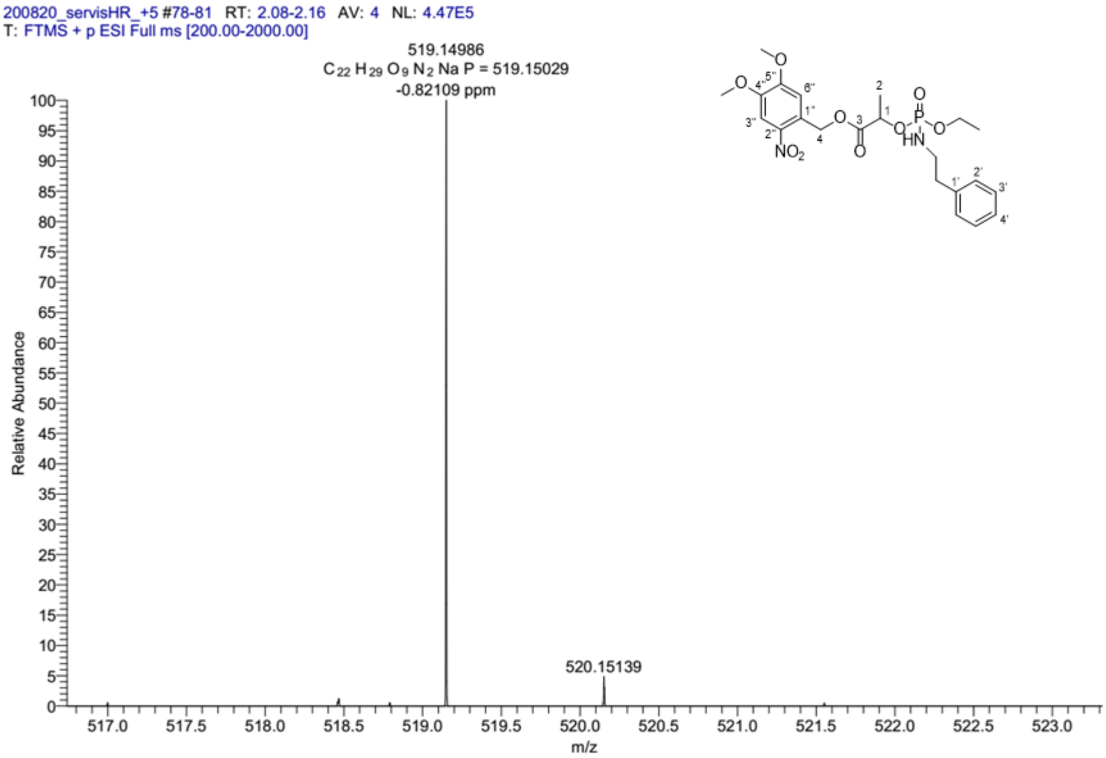

Figure S71. HR-MS spectrum of compound **7**.

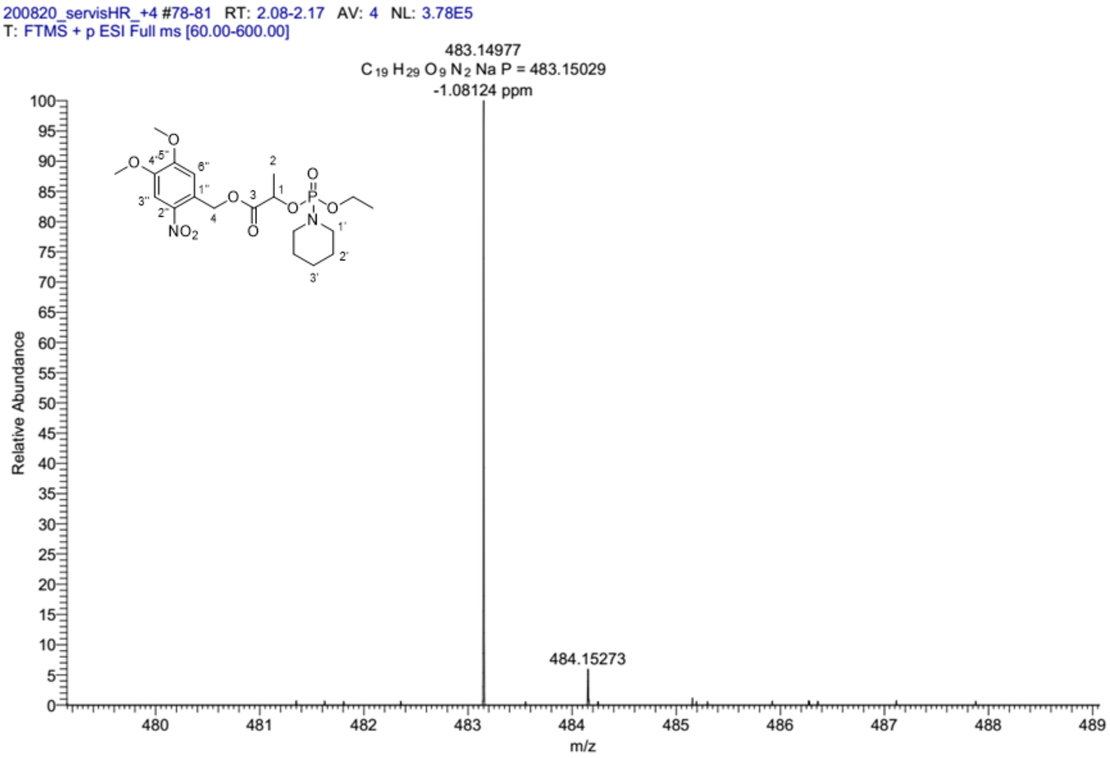

Figure S72. HR-MS spectrum of compound **8**.

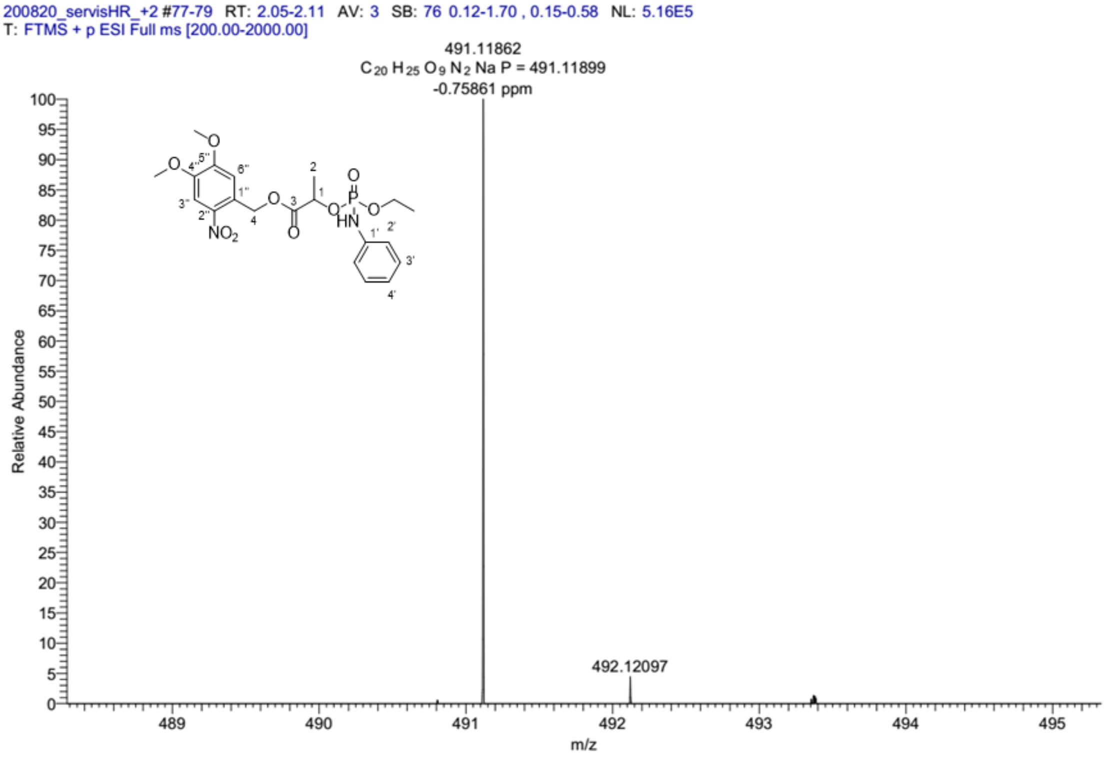

Figure S73. HR-MS spectrum of compound **9**.

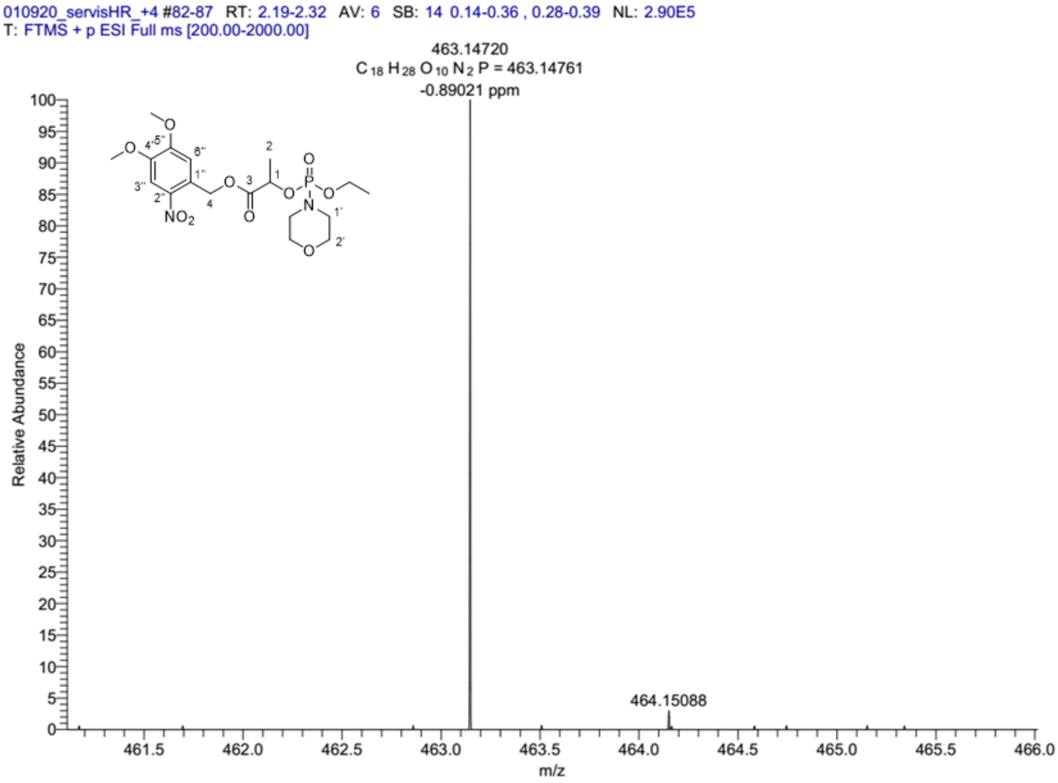

Figure S74. HR-MS spectrum of compound **10**.

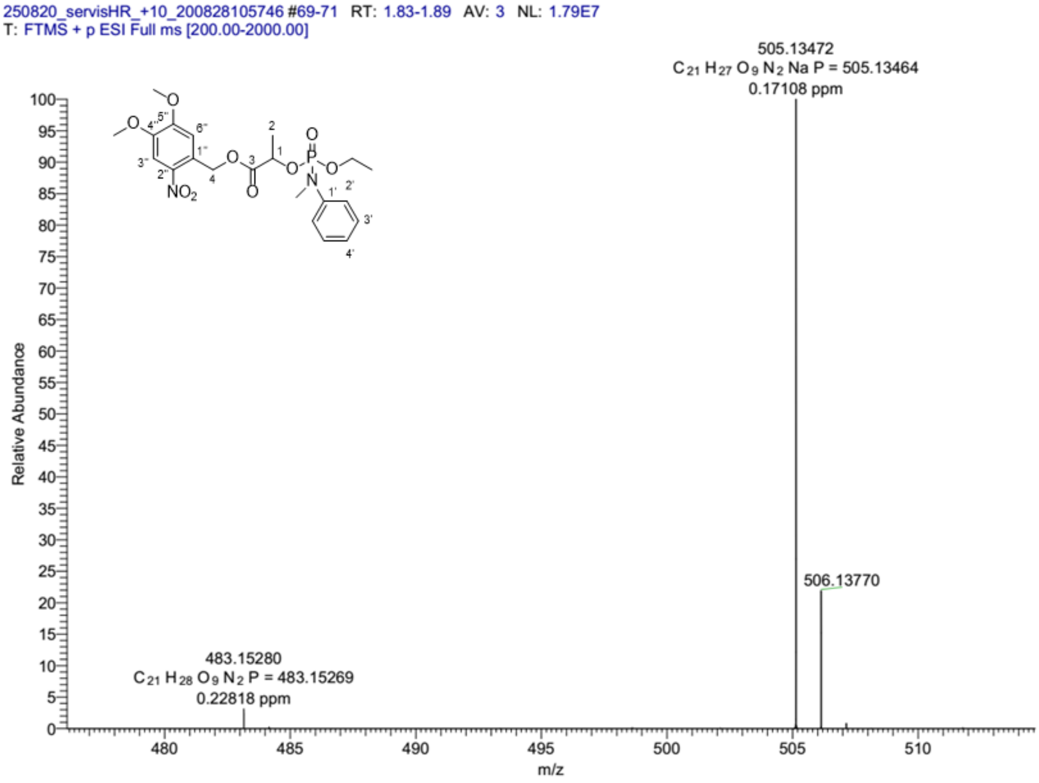

Figure S75. HR-MS spectrum of compound **11**.

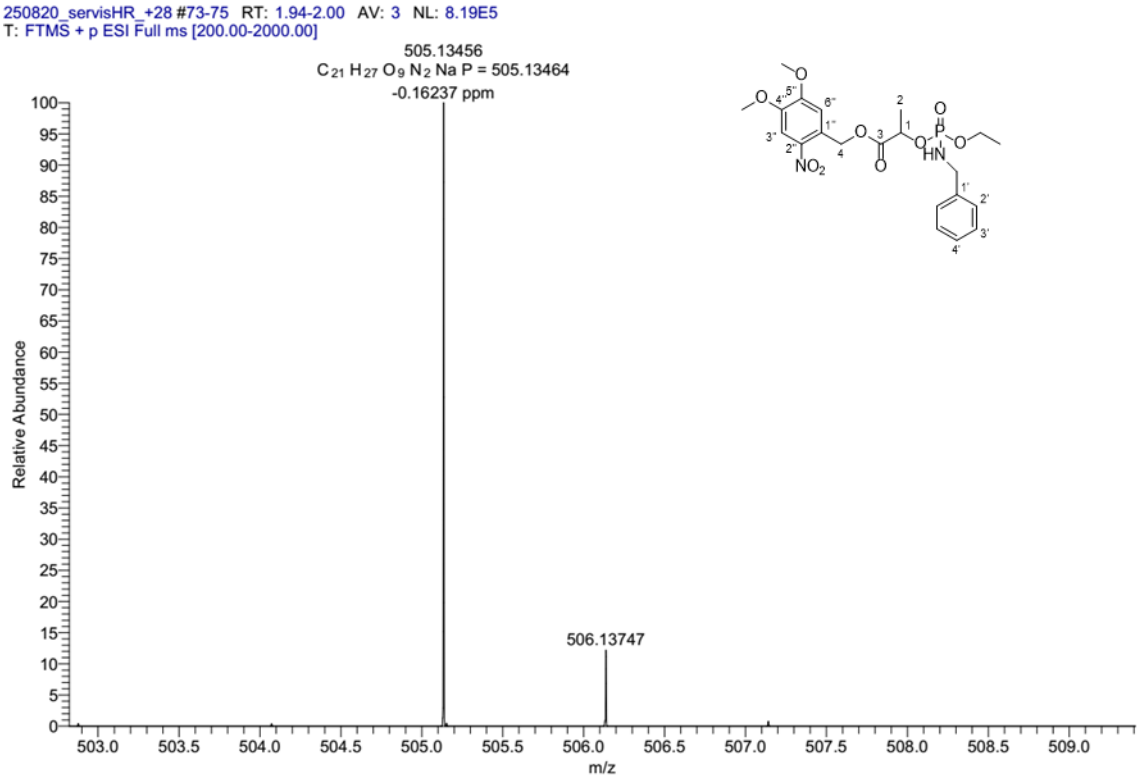

Figure S76. HR-MS spectrum of compound **12**.

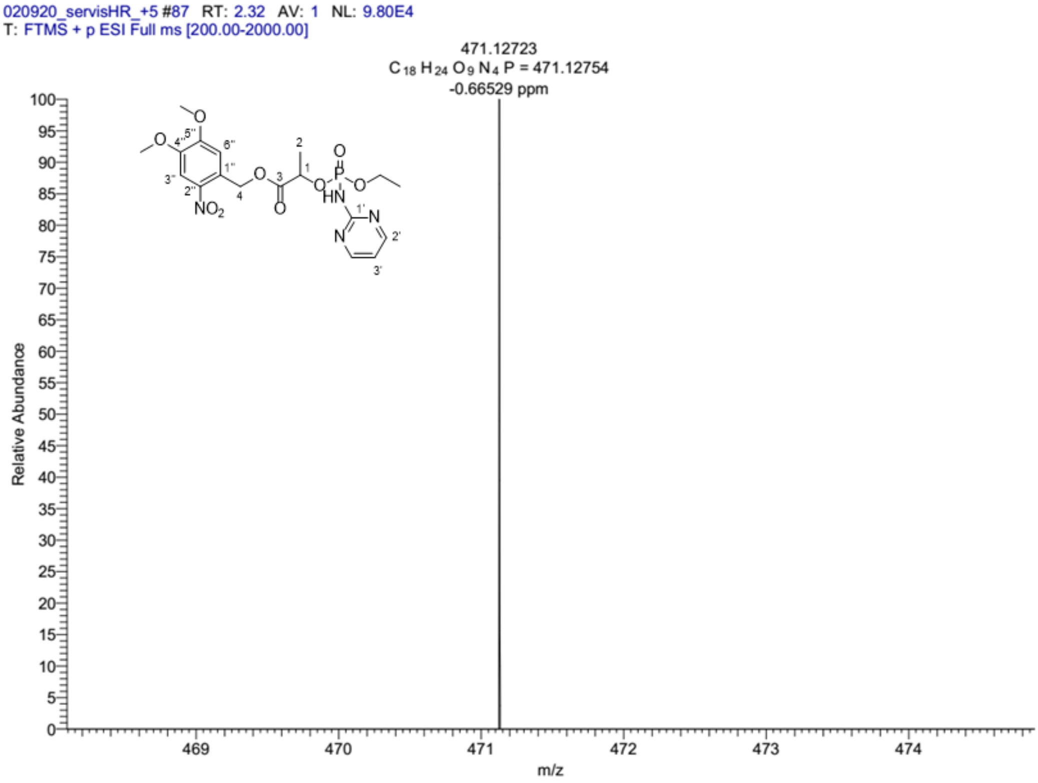

Figure S77. HR-MS spectrum of compound **13**.

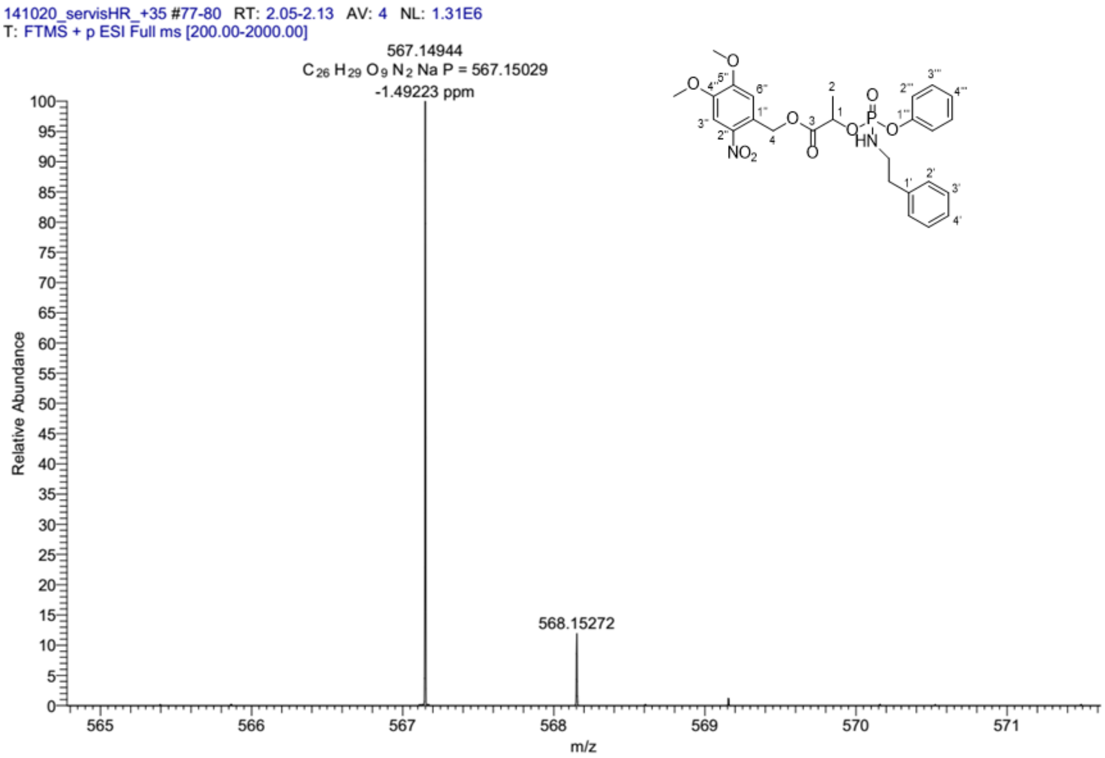

Figure S78. HR-MS spectrum of compound **14**.

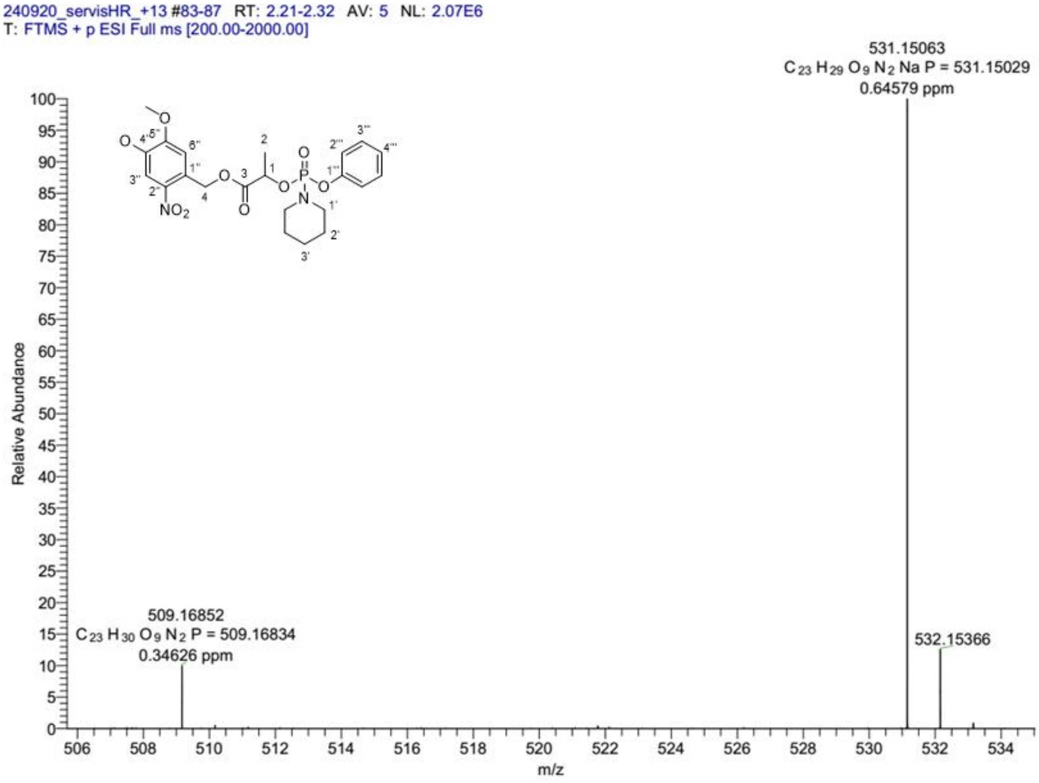

Figure S79. HR-MS spectrum of compound **15**.

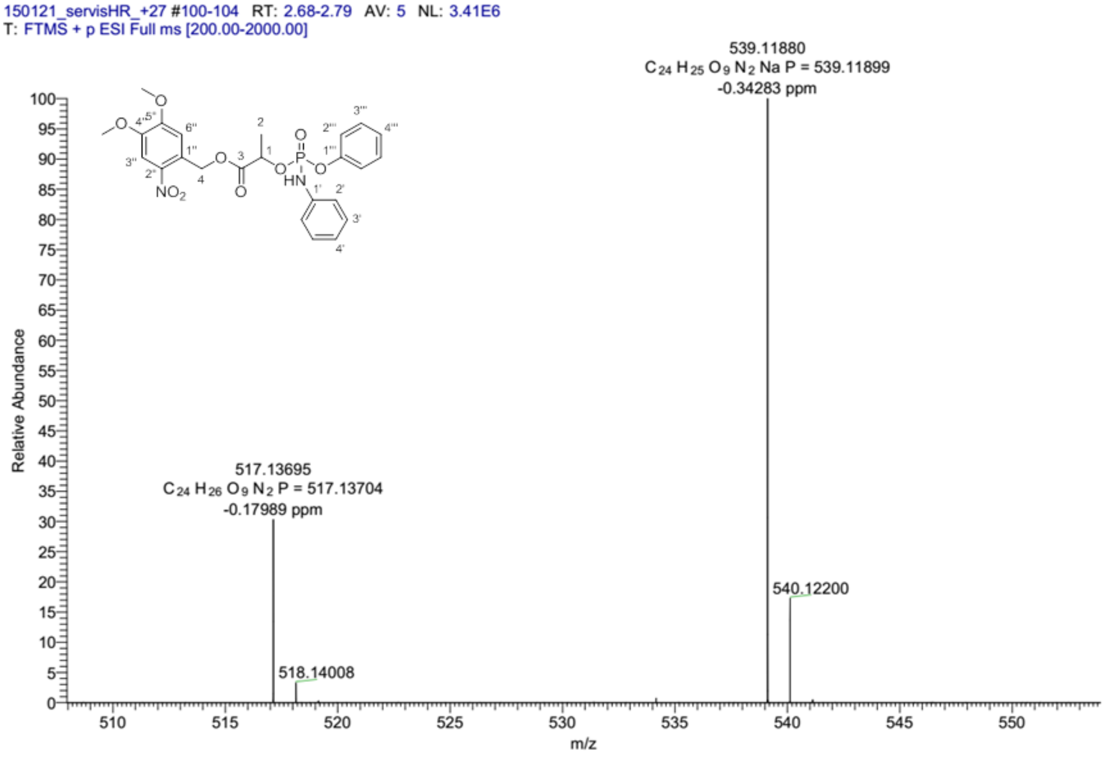

Figure S80. HR-MS spectrum of compound **16**.

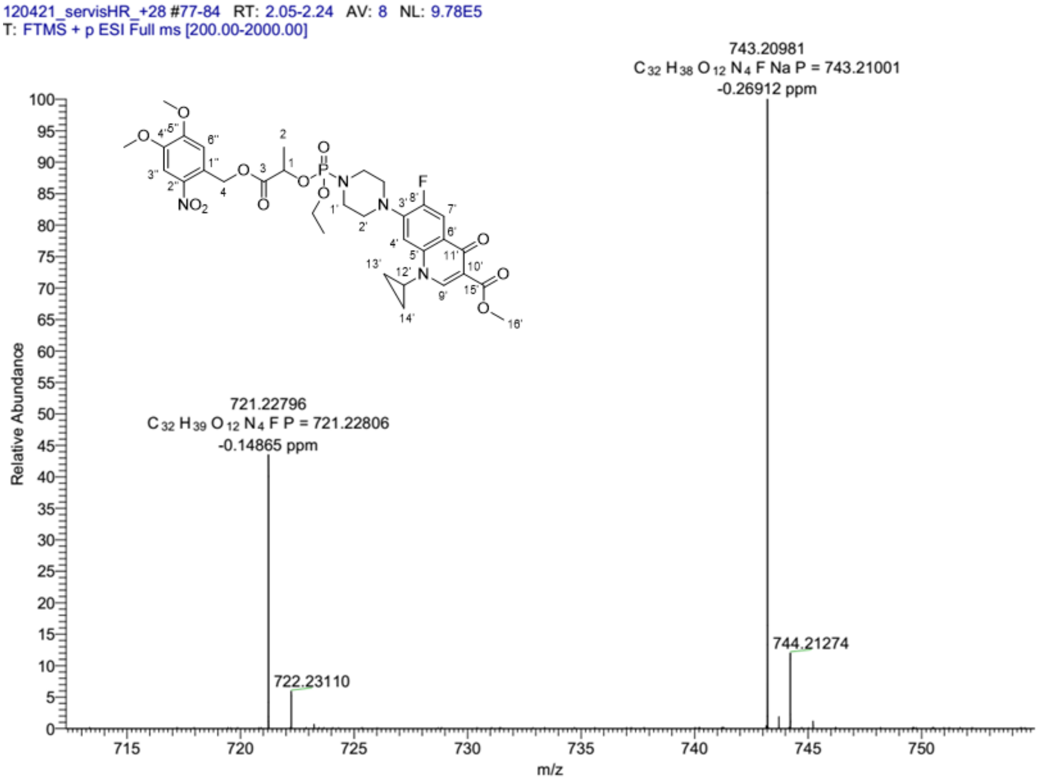

Figure S81. HR-MS spectrum of compound **17**.

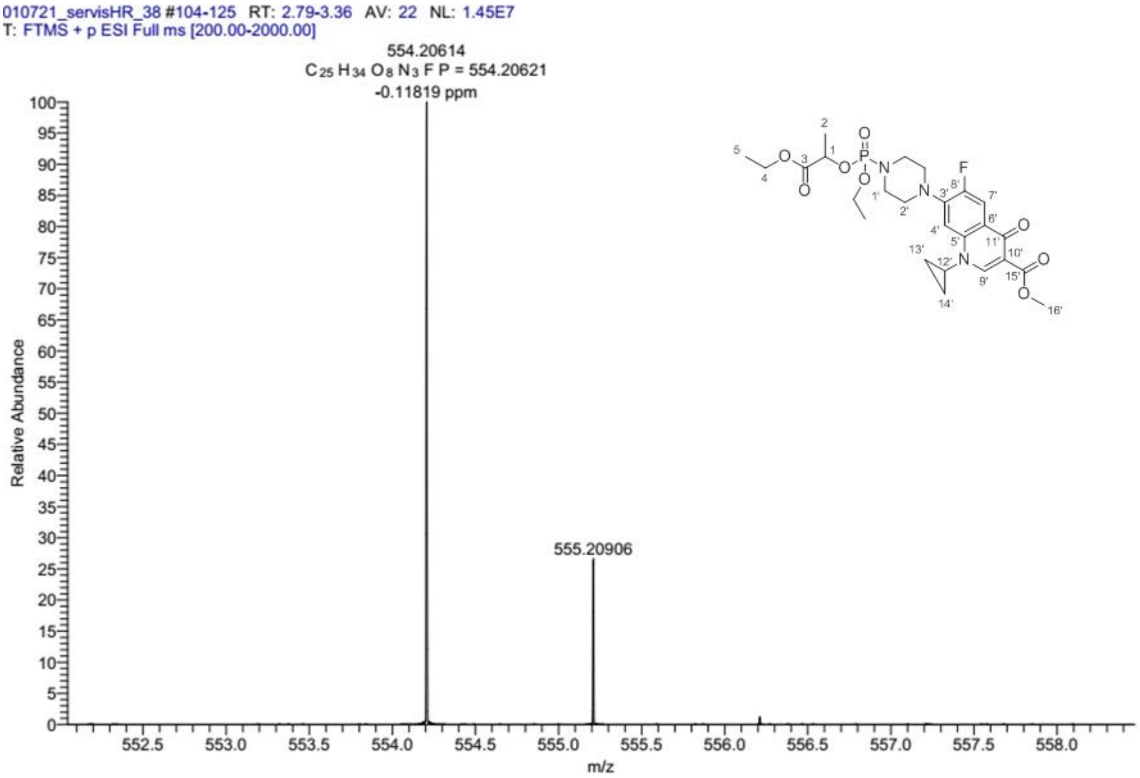

Figure S82. HR-MS spectrum of compound **18**.

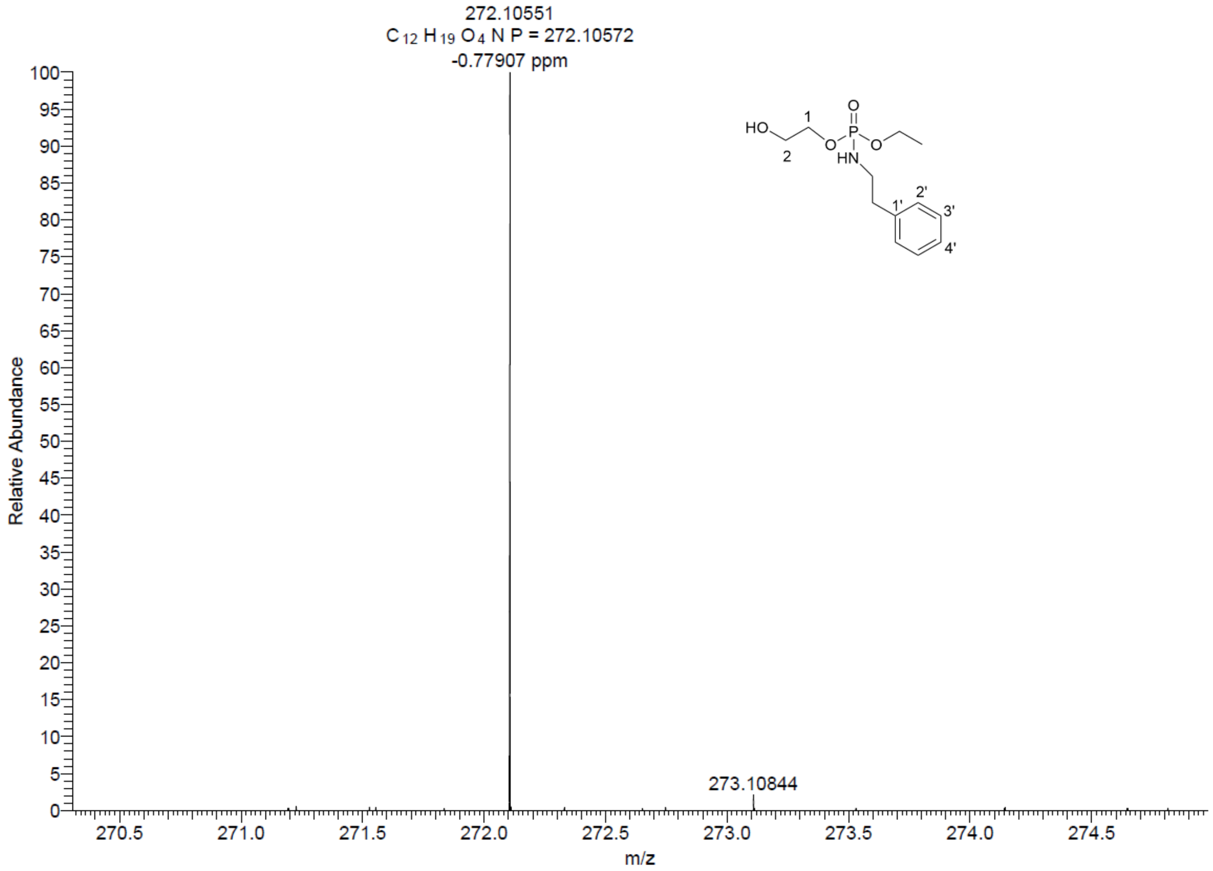

Figure S83. HR-MS spectrum of compound **1-I**.

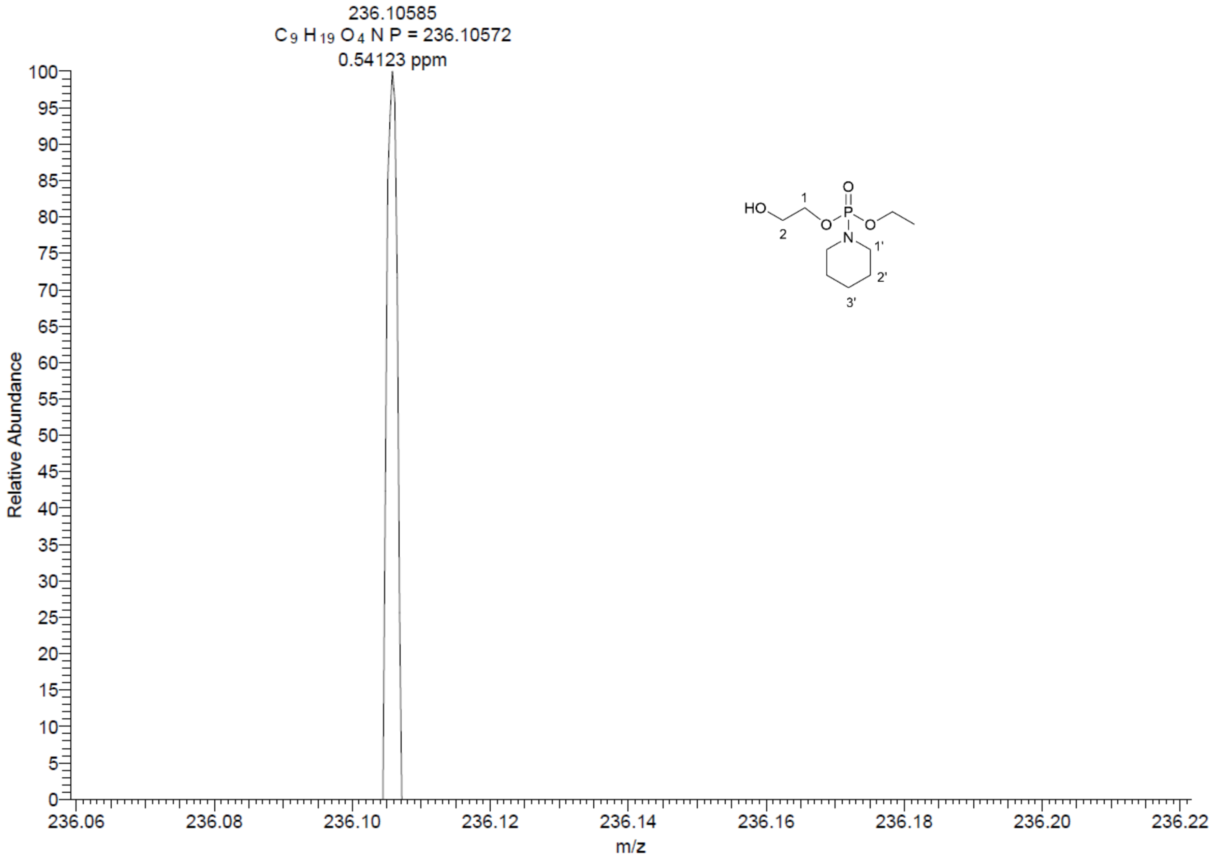

Figure S84. HR-MS spectrum of compound **2-I**.

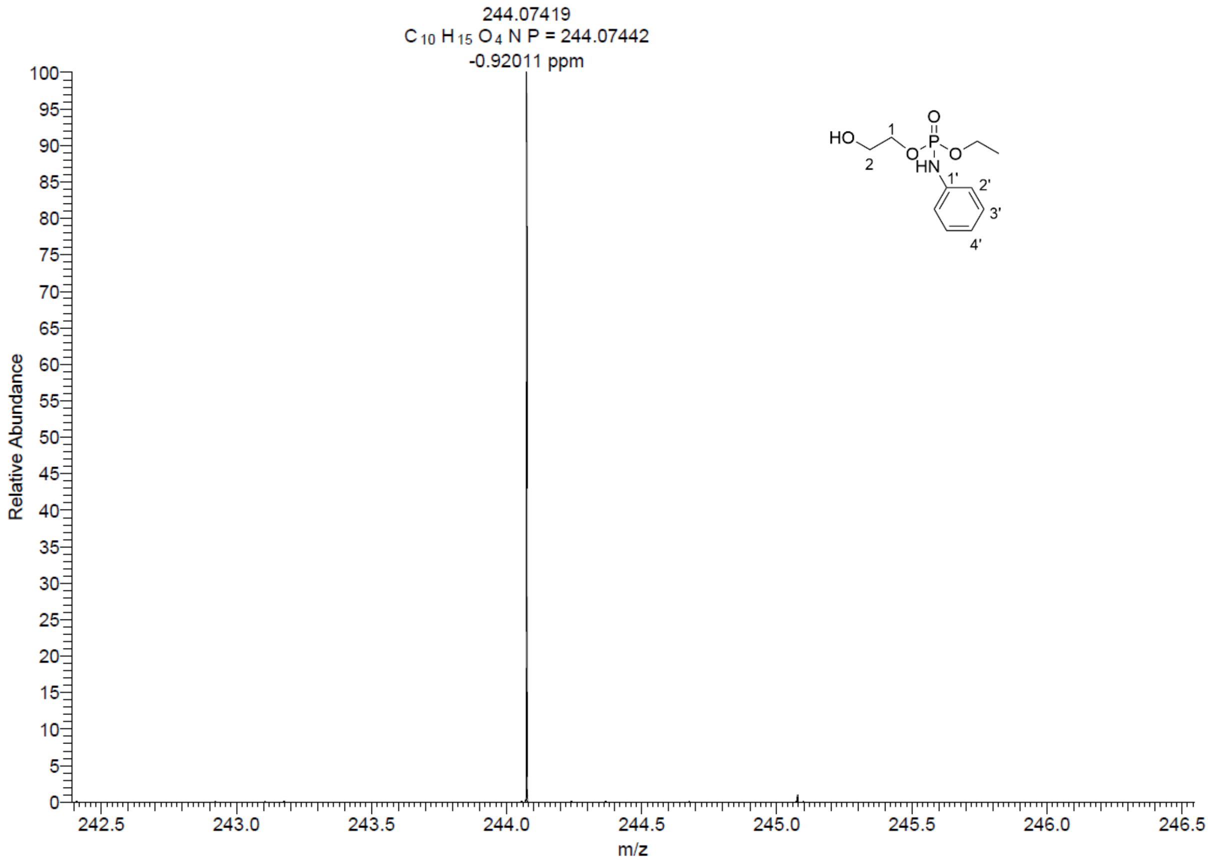

Figure S85. HR-MS spectrum of compound **3-I**.

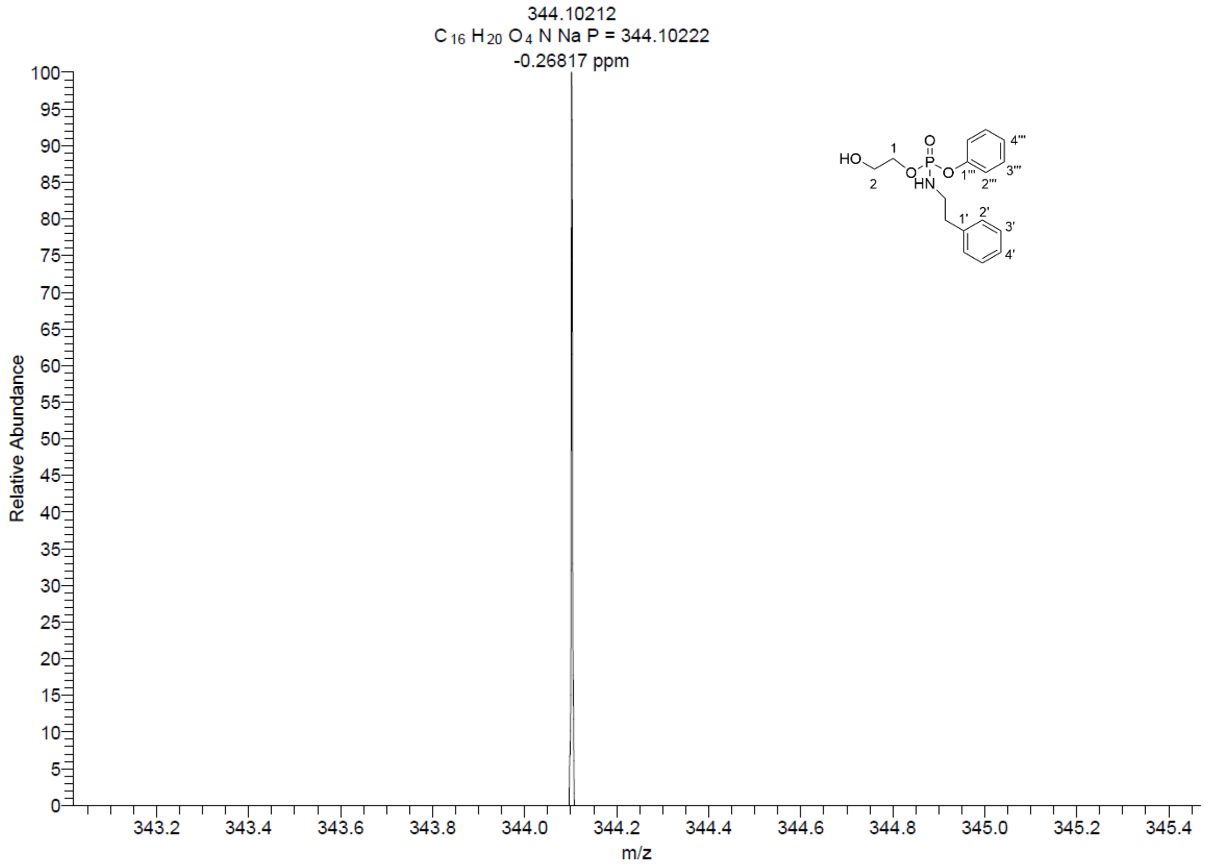

Figure S86. HR-MS spectrum of compound **4-I**.

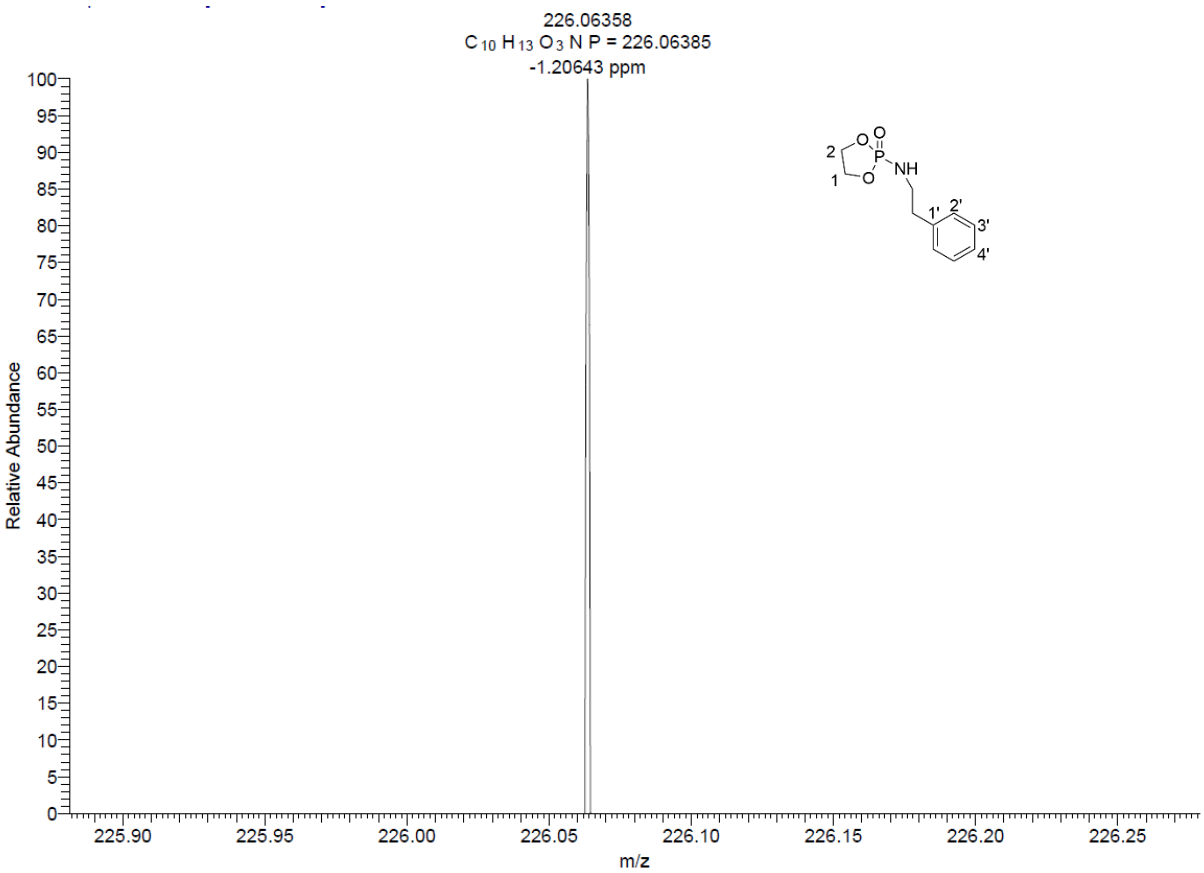

Figure S87. HR-MS spectrum of compound **4-cyc-I**.

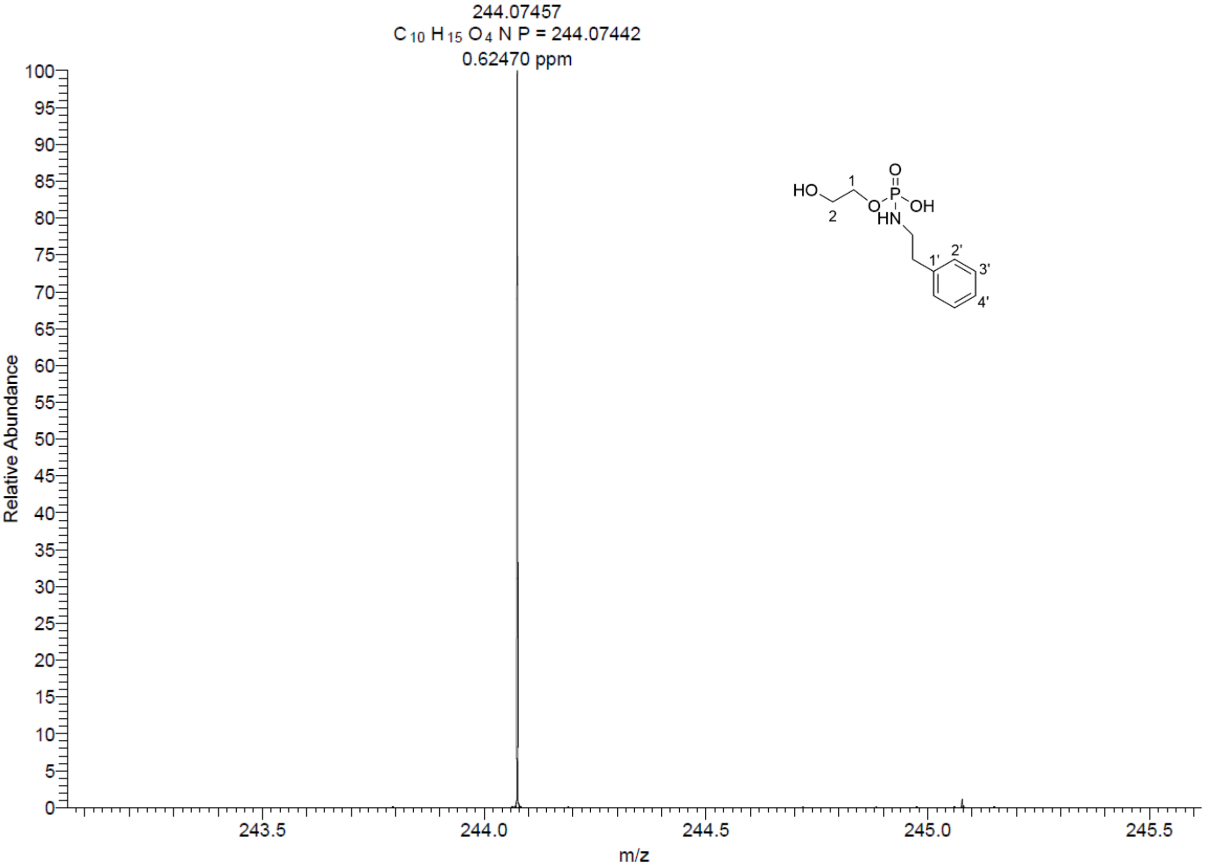

Figure S88. HR-MS spectrum of compound **4-P2**.

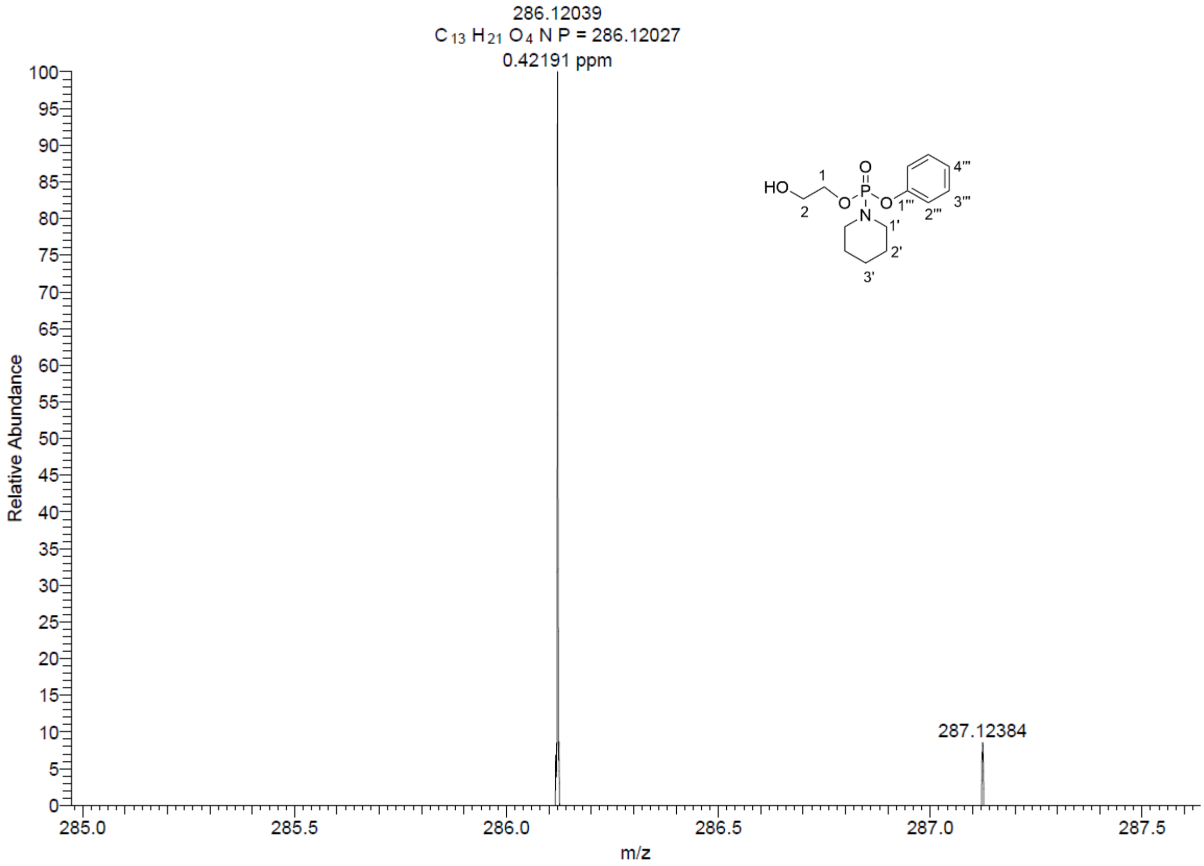

Figure S89. HR-MS spectrum of compound **5-I**.

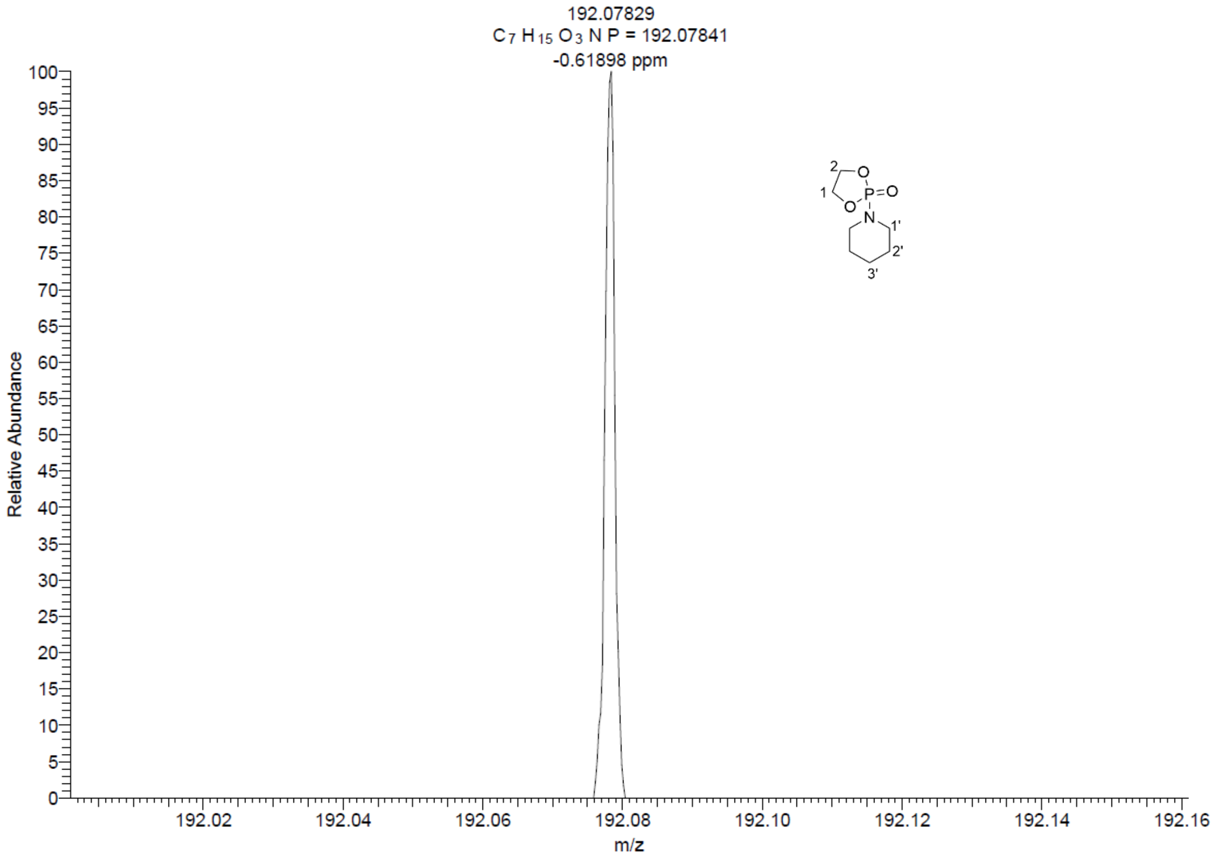

Figure S90. HR-MS spectrum of compound **5-cyc-I**.

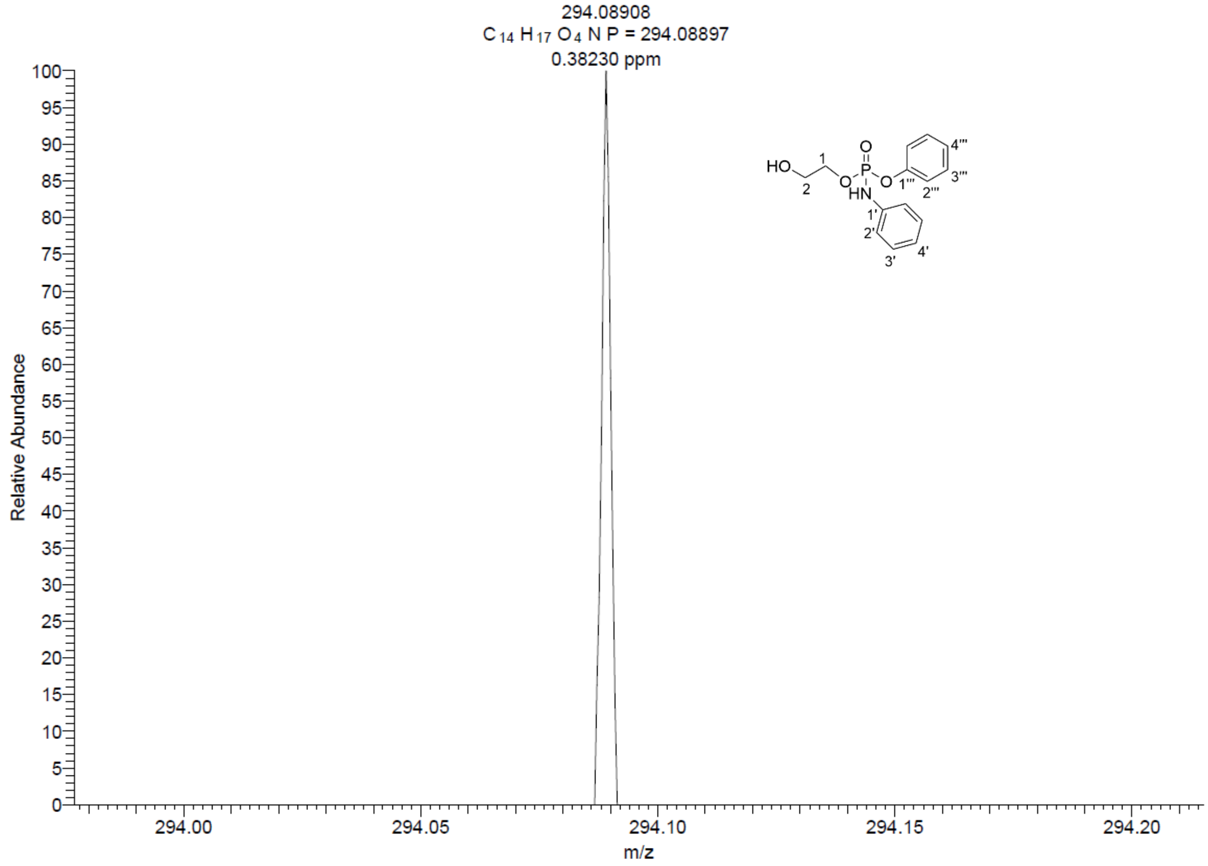

Figure S91. HR-MS spectrum of compound **6-I**.

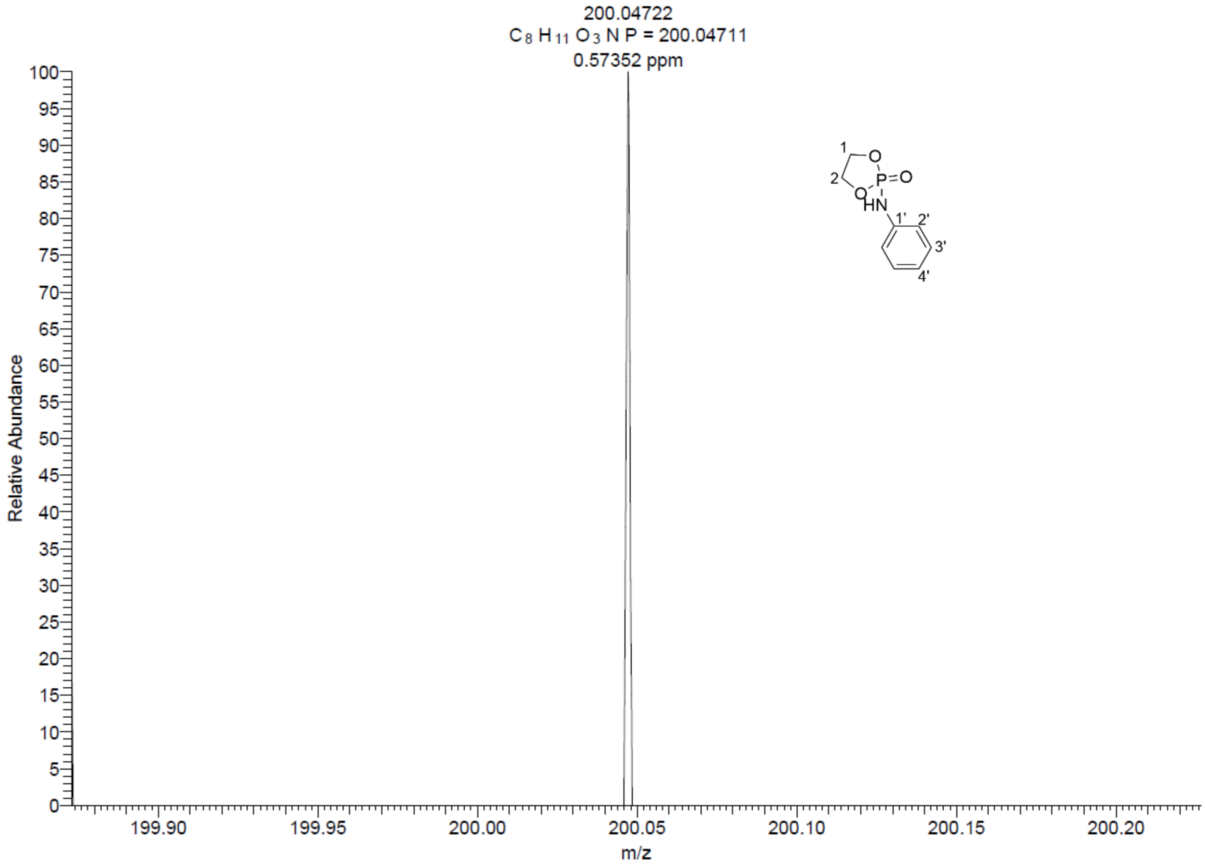

Figure S92. HR-MS spectrum of compound **6-cyc-I**.

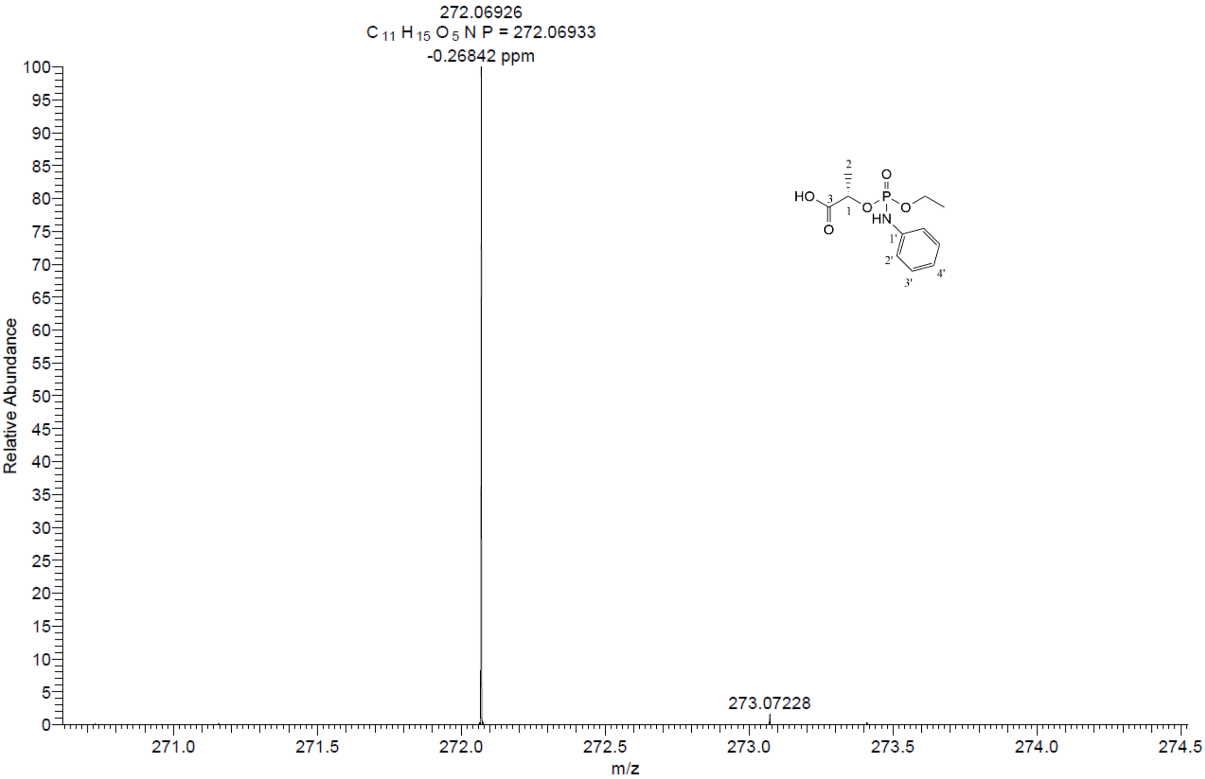

Figure S93. HR-MS spectrum of compound **9-I**.

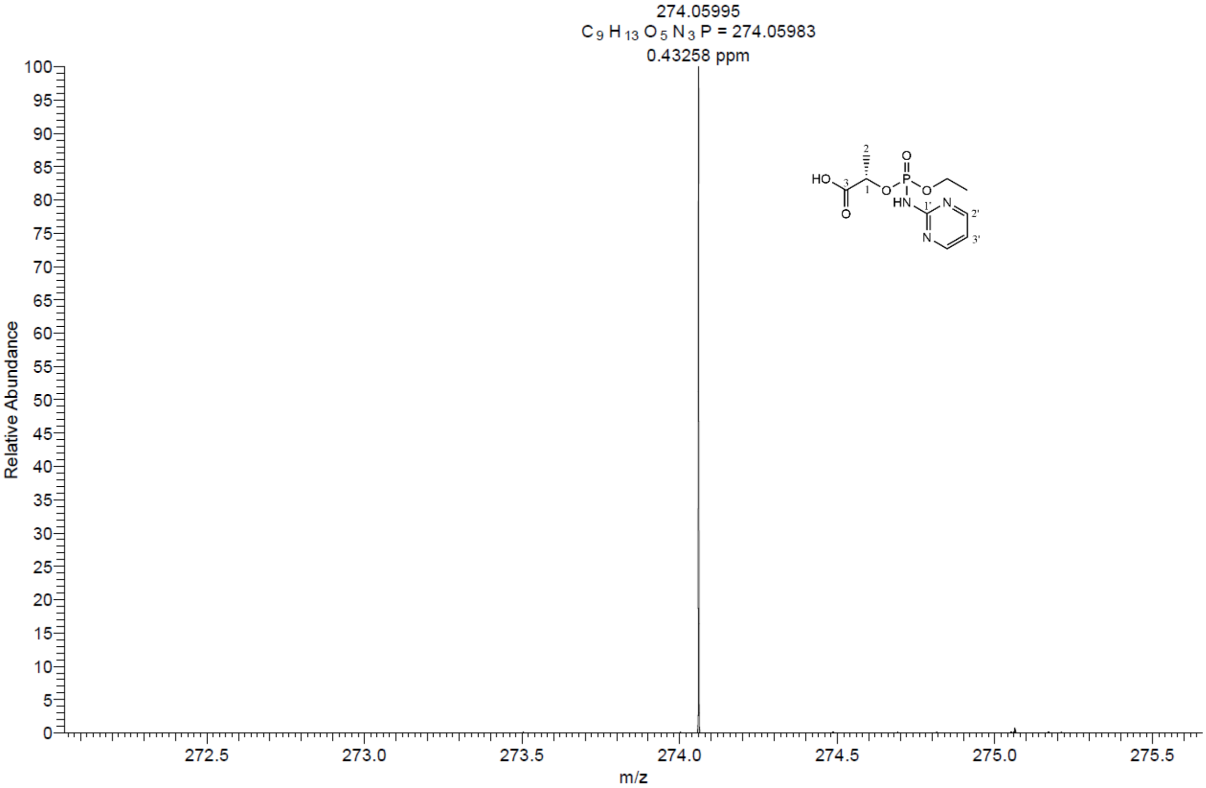

Figure S94. HR-MS spectrum of compound **13-I**.

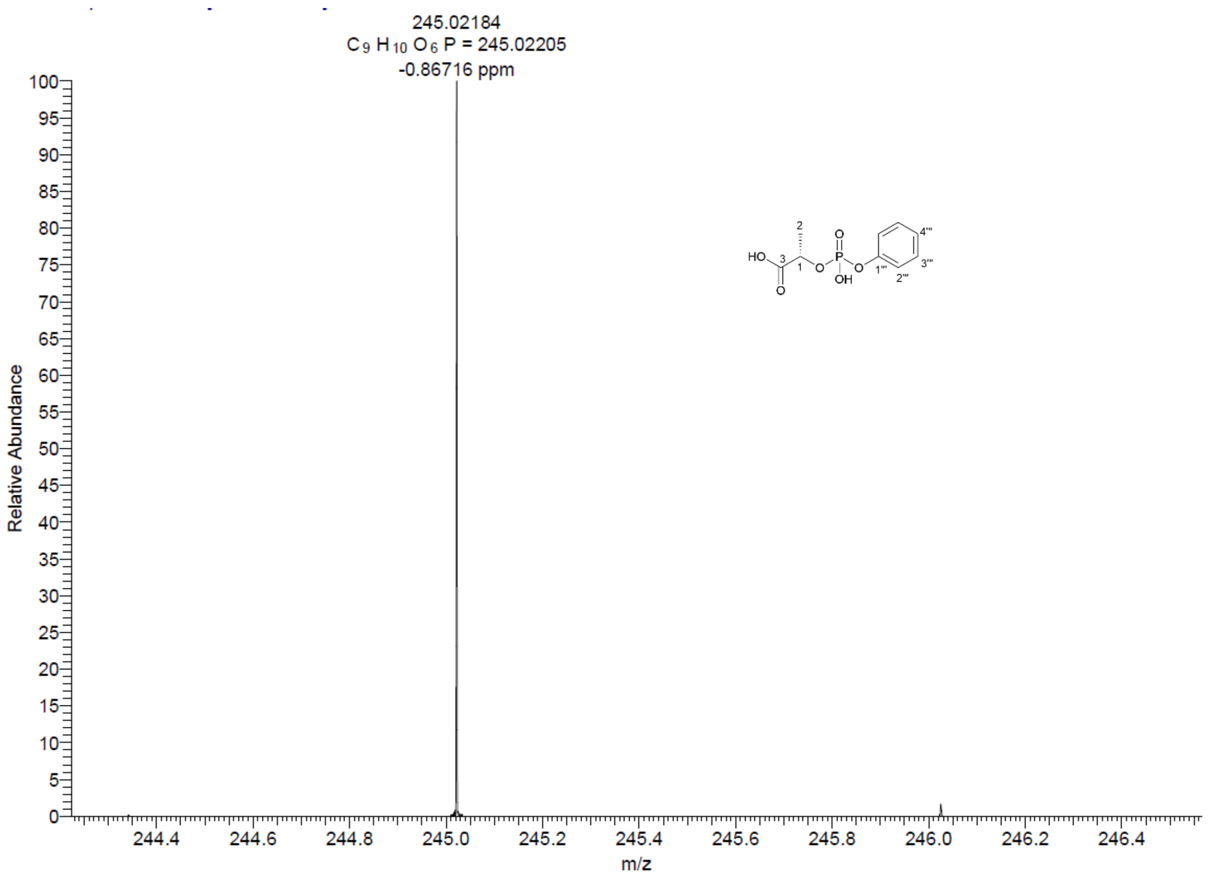

Figure S94. HR-MS spectrum of compound **14-P2**.

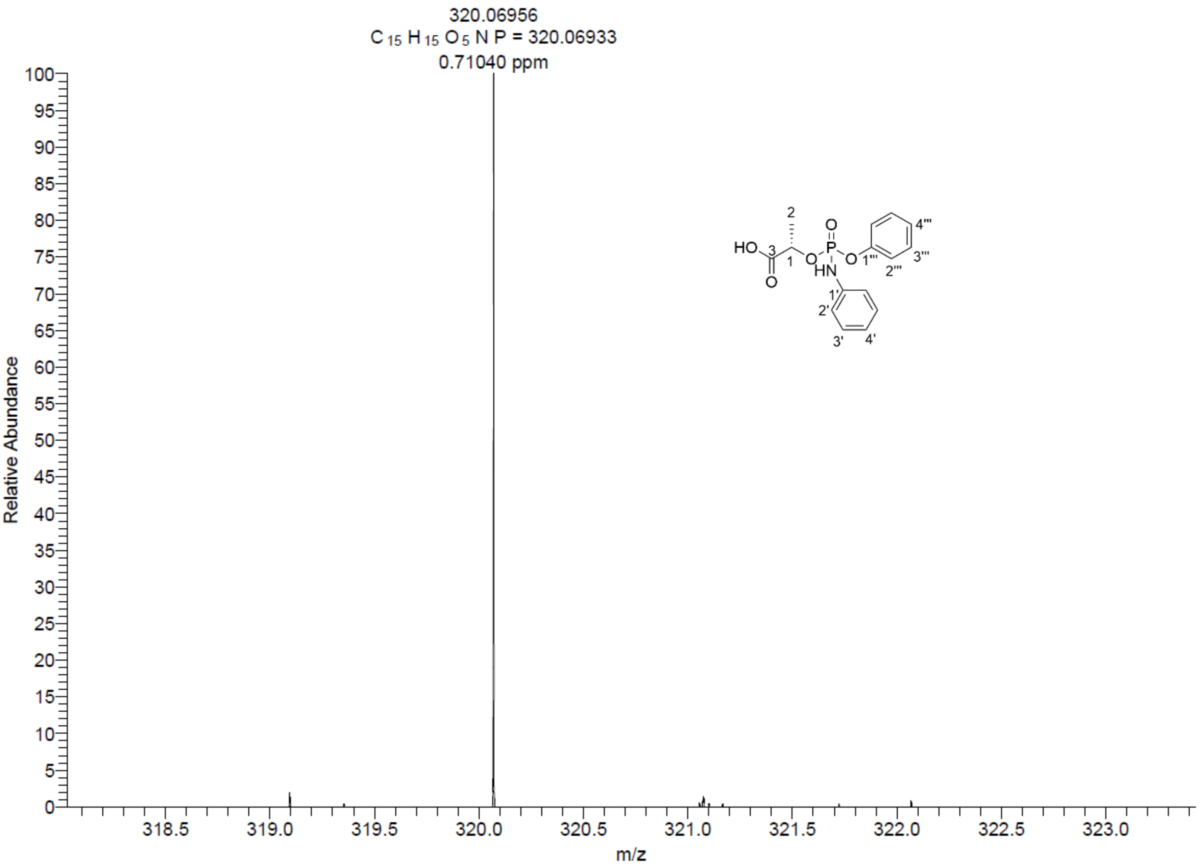

Figure S95. HR-MS spectrum of compound **16-I**.

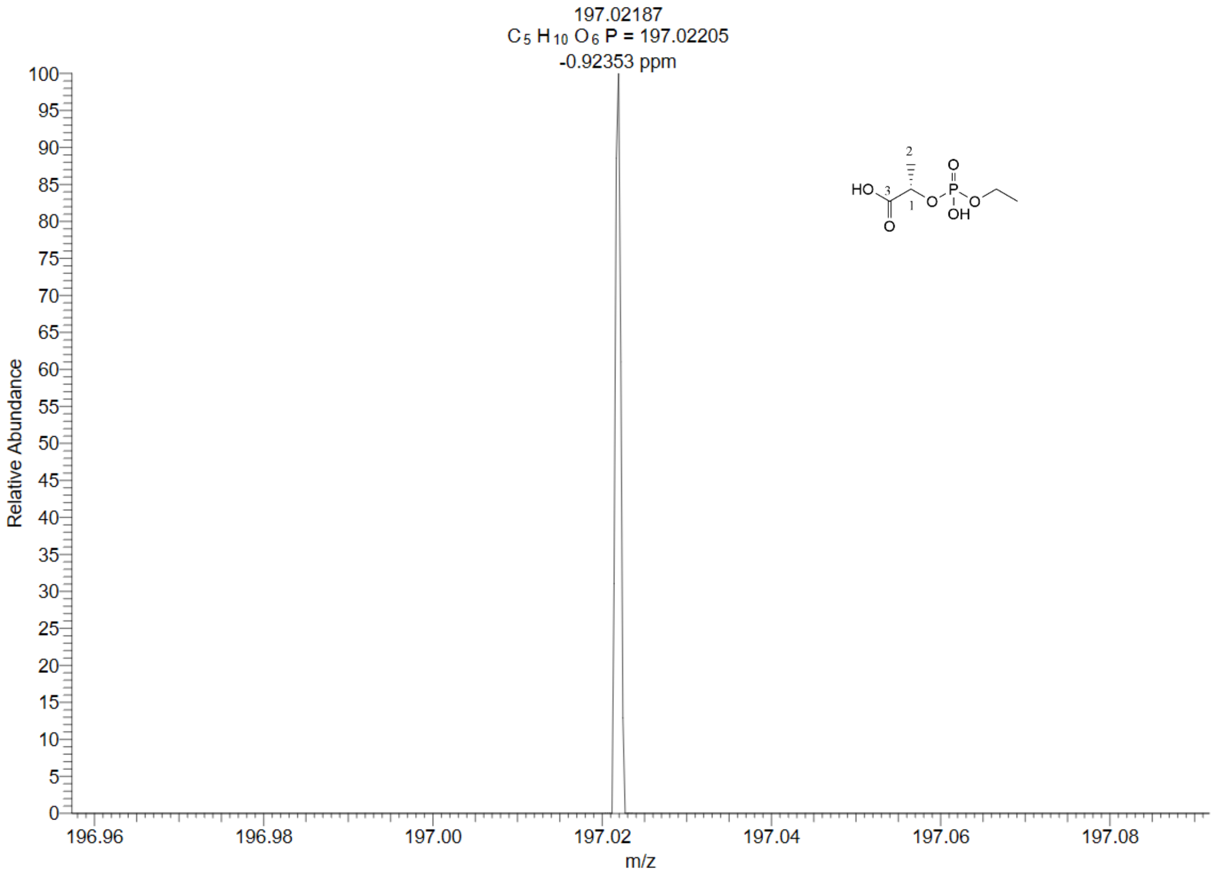

Figure S96. HR-MS spectrum of compound **9-P**.

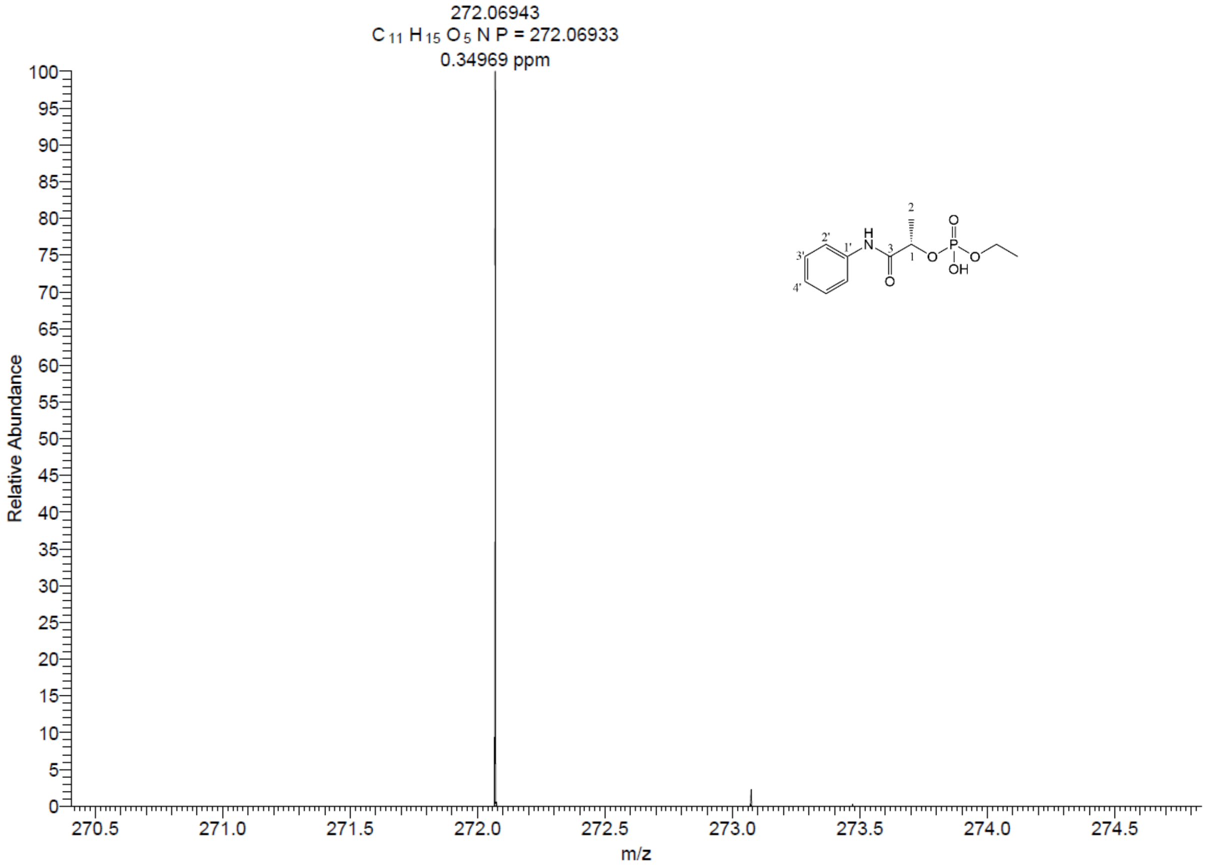

Figure S97. HR-MS spectrum of compound **9-X**.

5 Antibiotic activity of ciprofloxacin, and its derivatives 18 and 27.

Table S1. Antibiotic activity of ciprofloxacin, and its derivatives **18** and **27** against various bacterial strains and *Candida albigans*.

collection LF UP	ciprofloxacin						+ 4% BSA			**27** - ciprofloxacin-Me-ester						+ 4% BSA			gentamicin
bacterial strain or candida	MIC µg/mL			MBC µg/mL			MIC µg/mL			MIC µg/mL			MBC µg/mL			MIC µg/mL			µg/mL
experiment	1	2	3	1	2	3	1	2	3	1	2	3	1	2	3	1	2	3	
Escherichia coli ATCC 25922 = CCM 3954	0.008	0.008	0.008	0.008	0.008	0.008	0.01	0.008	0.01	4	4	4	4	4	4	8	8	2	0.5
Pseudomonas aeruginosa ATCC 27853 = CCM 3955	0.5	0.5	1	1	1	1	1	1	1	>128	>128	>128	>128	>128	>128	>128	>128	>128	1
Enterococcus faecalis ATCC 29212 = CCM 4224	2	2	2	4	4	4	2	2	2	>128	>128	>128	>128	>128	>128	>128	>128	>128	-
Staphylococcus aureus ATCC 29213 = CCM 4223	1	1	1	0.5	0.5	0.5	1	1	1	128	>128	>128	>128	>128	>128	>128	>128	>128	-
																			-
Staphylococcus epidermidis CCM 7221	32	32	32	32	32	32	-	-	-	>128	>128	>128	>128	>128	>128	-	-	-	-
Staphylococcus aureus 4591	128	128	128	128	128	>128	-	-	-	>128	>128	>128	>128	>128	>128	-	-	-	-
Enterococcus faecium 419/ANA	128	128	64	>128	>128	>128	-	-	-	>128	>128	>128	>128	>128	>128	-	-	-	-
Escherichia coli CE5556	>128	>128	>128	>128	>128	>128	-	-	-	>128	>128	>128	>128	>128	>128	-	-	-	-
Escherichia coli 20574/C	>128	>128	>128	>128	>128	>128	-	-	-	>128	>128	>128	>128	>128	>128	-	-	-	-
Pseudomonas aeruginosa R	32	16	32	64	64	16	-	-	-	>128	>128	>128	>128	>128	>128	-	-	-	-
Proteus mirabilis 16585/B	≤0,063	≤0,063	≤0,063	≤0,063	≤0,063	≤0,063	-	-	-	16	16	16	16	16	16	-	-	-	-
																			-
Candida albicans ATCC 90028 = CCM 8161	>128	>128	>128	>128	>128	>128	-	-	-	>128	>128	>128	>128	>128	>128	-	-	-	-

collection LF UP	**18** - ciprofloxacin phosphoramidate						+ 4% BSA									+ 4% BSA			gentamicin
bacterial strain or candida	MIC µg/mL			MBC µg/mL			MIC µg/mL			MIC µg/mL			MBC µg/mL			MIC µg/mL			µg/mL
experiment	1	2	3	1	2	3	1	2	3	1	2	3	1	2	3	1	2	3	
Escherichia coli ATCC 25922 = CCM 3954	32	32	32	32	32	32	64	32	64										0.5
Pseudomonas aeruginosa ATCC 27853 = CCM 3955	>128	>128	>128	>128	>128	>128	>128	>128	>128										1
Enterococcus faecalis ATCC 29212 = CCM 4224	>128	>128	>128	>128	>128	>128	>128	>128	>128										-
Staphylococcus aureus ATCC 29213 = CCM 4223	>128	>128	>128	>128	>128	>128	>128	>128	>128										-
																			-
Staphylococcus epidermidis CCM 7221	>128	>128	>128	>128	>128	>128	-	-	-										-
Staphylococcus aureus 4591	>128	>128	>128	>128	>128	>128	-	-	-										-
Enterococcus faecium 419/ANA	>128	>128	>128	>128	>128	>128	-	-	-										-
Escherichia coli CE5556	>128	>128	>128	>128	>128	>128	-	-	-										-
Escherichia coli 20574/C	>128	>128	>128	>128	>128	>128	-	-	-										-
Pseudomonas aeruginosa R	>128	>128	>128	>128	>128	>128	-	-	-										-
Proteus mirabilis 16585/B	128	128	>128	>128	128	128	-	-	-										-
